# Supplementary material for: Cobalt catalyzed practical hydroboration of terminal alkynes with time-dependent stereoselectivity
Source: Nat Commun. 2024 Mar 12;15:2208. doi: 10.1038/s41467-024-46550-y (PMC10928171; doi:10.1038/s41467-024-46550-y)
Supplement: Supplementary file 1 — Supplementary Information [file 41467_2024_46550_MOESM1_ESM.pdf]

Supplementary Information

Cobalt catalyzed practical hydroboration of terminal alkynes with time-dependent stereoselectivity

Jinglan Wen,[1,2] Yahao Huang,[1,2] Yu Zhang,[1,2] Hansjörg Grützmacher,[2,3] Peng Hu*[1,2,4]

[1]Institute of Green Chemistry and Molecular Engineering, School of Chemistry, Sun Yat-sen University, Guangzhou 510006, PR China
[2]Lehn Institute of Functional Materials, School of Chemistry, Sun Yat-sen University, Guangzhou 510006, PR China
[3]Department of Chemistry and Applied Biosciences, ETH Zürich, Zürich 8093, Switzerland
[4]State Key Laboratory of Structural Chemistry, Fujian Institute of Research on the Structure of Matter, Chinese Academy of Sciences, Fuzhou, Fujian 350002, PR China
*Correspondence to: hupeng8@mail.sysu.edu.cn

Table of Contents

1. General Method 2

1.1 Materials and methods 2

1.2 Synthesis of Ligand 2

1.3 Synthesis of substrate 4

2. General procedure for condition optimization 6

3. Substrate investigation and characterization 12

4. Time-dependent Z/E selective transformation 28

5. Large scale reaction 37

6. Mechanistic Study 38

6.1 Procedure for monitoring the hydroboration of phenylacetylene 38

6.2 Procedure for kinetic experiments of catalyst concentration 38

6.3 Deuterium labeling experiments 39

6.4 Kinetic isotope effects experiment: 44

6.5 Inhabitation experiment with inert alkyne: 46

6.6 Kinetic trace of Z-selective products: 46

6.7 HRMS of in situ formed (CNC-^{i}Pr)Co(II) complex: 47

6.8 Single crystal of (CNC-^{i}Pr)$_2$Co(III) complex 49

6.9 HRMS of in situ formed (CNC-^{i}Pr)Co(I)H complex: 52

6.10 Detection of H_2 by gas chromatography 54

7. NMR spectra 55

8. References 118

1. General Method

1.1 Materials and methods

All air- and moisture-sensitive manipulations were carried out using standard Schlenk techniques or in an M. Braun inert atmosphere glovebox containing an atmosphere of purified nitrogen. Solvents for air- and moisture sensitive manipulations were dried and deoxygenated using literature procedures. Reagents were purchased from Energy Chemical, Bidepharm, Aladdin, TCI, Adamas-Beta, and Alfa used without further purification unless otherwise stated. Commercially available terminal alkynes were used before recrystallization or distillation, and stored at -20 °C. DMF was purchased from Alfa (Stock No.: 43465, anhydrous, amine free), used without further purification and stored in dark at -20 °C. Gas chromatography-mass spectrometry analysis was performed on an Agilent 5977B and Agilent GC/MSD 8890 GC system. High resolution mass spectra were obtained from Orbitrap LC/MS (Q Exactive). ^{1}H NMR spectra were recorded at 400 MHz or 600 MHz, ^{13}C NMR spectra were recorded at 101 MHz or 151 MHz, on a Bruker AVANCE III 400 (400 MHz) or Bruker AVANCE III 600 (600 MHz). ^{1}H NMR chemical shifts were determined relative to the signal of the residual protonated solvent: $CDCl_3$ δ 7.26. ^{13}C NMR chemical shifts were determined relative to $CDCl_3$ at δ 77.16. Data for ^{1}H NMR are reported as follows: chemical shift (δ ppm), multiplicity (s = singlet, d = doublet, t = triplet, m = multiplet, q = quartet, br = broad), integration, coupling constant (Hz). ^{13}C NMR spectra was reported as chemical shifts in ppm and multiplicity where appropriate.

1.2 Synthesis of Ligand

Synthesis of CNC-^{i}Pr

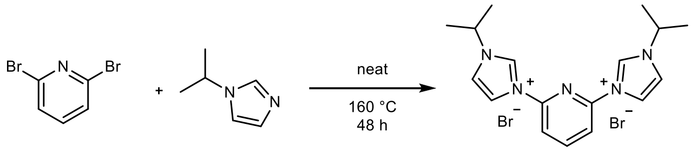

CNC-^{i}Pr
99%

The title compound was synthesized according to a reported procedure with modifications[1]. In a nitrogen atmosphere, a pressure tube with the 1-isopropyl-1H-imidazole (66 mmol, 7.3 g) and 2,6-diboromopyridine (31 mmol, 7.6 g) was sealed and heated for 48 h at 160 °C. After cooling to room temperature, the brown mixture was triturated several times with DCM and Et_2O. The product was obtained as an off-white powder in 99% yield (14.3 g). See spectrum

^{1}H NMR (600 MHz, DMSO-d6) δ 10.63 (s, 2H), 8.91 (s, 2H), 8.63 (t, J = 8.1 Hz, 1H), 8.32 (m, 4H), 4.88 (hept, J = 6.7 Hz, 2H), 1.63 (d, J = 6.7 Hz, 12H). The data is in accordance with the published results[1].

Synthesis of CNC-H

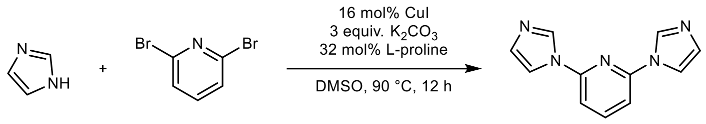

CNC-H
95%

The title compound was synthesiszed according to a reported procedure[2]. In a nitrogen atmosphere, a pressure tube with 2,6-dibromopyridine (590 mg, 2.5 mmol), imidazole (375 mg, 5.5 mmol), CuI (71 mg, 0.4 mmol), L-proline (87 mg, 0.8 mmol), K_2CO_3 (1.03 g, 7.5 mmol) and DMSO (3 mL) was sealed and heated for 12 h at 90 °C. After cooling to room temperature, 250 mL H_2O was added into the reaction mixture. The reaction mixture was extracted with dichloromethane (3×250 mL). The combined organic layers were washed with H_2O (5×300 mL) and dried with Na2SO4. The solvent was removed using reduced pressure and the residual solid was recrystallized from a mixture of dichloromethane and ether; the title compound was afforded as a white solid (500 mg; yield: 95%). See spectrum

^{1}H NMR (400 MHz, Chloroform-d) δ 8.39 (s, 2H), 7.97 (t, J = 8.0 Hz, 1H), 7.67 (s, 2H), 7.30 – 7.24 (m, 4H). The data is in accordance with the published results[2].

Synthesis of CNC-Me

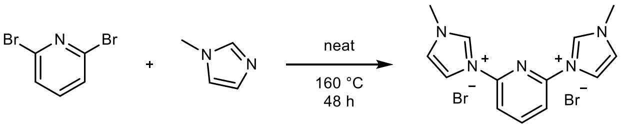

CNC-Me
84%

The title compound was synthesized using the identical methodology as **CNC-*i*Pr**. The reaction of 1-methyl-1H-imidazole (2.4 g, 30 mmol) and 2,6-dibromopyridine (2.96 g, 12.5 mmol) afforded **CNC-Me** as a white solid (4.2 g, 84%). See spectrum

^{1}H NMR (400 MHz, DMSO-d_6) δ 10.49 (s, 2H), 8.77 (s, 2H), 8.59 (t, *J* = 8.1 Hz, 1H), 8.21 (d, *J* = 8.1 Hz, 2H), 8.05 (s, 2H), 4.02 (s, 6H). The data is in accordance with the published results[3].

Synthesis of CNC-Et

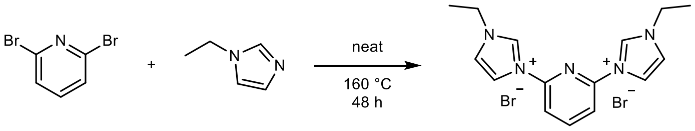

CNC-Et
87%

The title compound was synthesized using the identical methodology as **CNC-*i*Pr**. The reaction of 1-ethyl-1H-imidazole (2.9 g, 30 mmol) and 2,6-dibromopyridine (2.96 g, 12.5 mmol) afforded **CNC-Et** as a white solid (4.6 g, 87%). See spectrum

^{1}H NMR (400 MHz, DMSO-d_6) δ 10.59 (s, 2H), 8.84 (s, 2H), 8.62 (t, J = 8.0 Hz, 1H), 8.27 (d, J = 8.1 Hz, 2H), 8.20 (s, 2H), 4.40 (q, J = 7.2 Hz, 4H), 1.57 (t, J = 7.2 Hz, 6H). The data is in accordance with the published results[4].

Synthesis of CNC-^{t}Bu

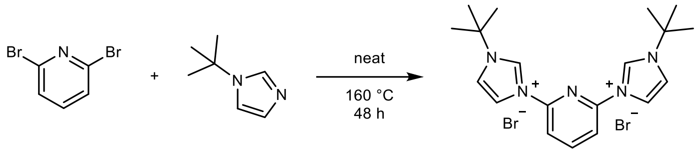

CNC-^{t}Bu
77%

The title compound was synthesized using the identical methodology as **CNC-^{i}Pr**. The reaction of 1-(tert-butyl)-1H-imidazole (3.7 g, 30 mmol) and 2,6-dibromopyridine (2.96 g, 12.5 mmol) afforded **CNC-^{t}Bu** as a pale yellow solid (4.9 g, 77%). See spectrum
^{1}H NMR (400 MHz, DMSO-d_6) δ 10.15 (s, 2H), 8.97 (s, 2H), 8.64 (t, J = 8.1 Hz, 1H), 8.44-8.41 (m, 4H), 1.73 (s, 18H). The data is in accordance with the published results[1].

Synthesis of CNC-Mes

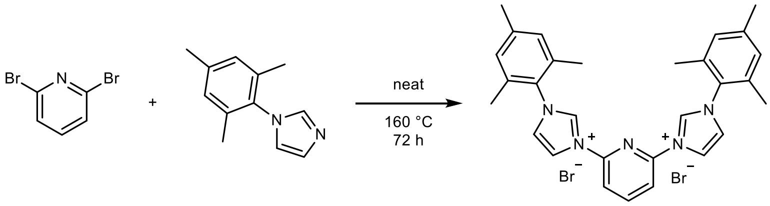

CNC-Mes
8%

The title compound was synthesized using the identical methodology as **CNC-^{i}Pr**. The reaction of 1-mesityl-1H-imidazole (1.86 g, 10 mmol) and 2,6-dibromopyridine (0.95 g, 4 mmol) afforded **CNC-Mes** as a pale yellow solid (0.2 g, 8%). See spectrum

^{1}H NMR (400 MHz, Chloroform-d) δ 11.89 (s, 2H), 9.83 (s, 2H), 9.10 (d, J = 8.1 Hz, 2H), 8.29 – 8.25 (m, 1H), 7.32 (s, 2H), 7.01 (s, 4H), 2.32 (s, 6H), 2.17 (s, 12H). The data is in accordance with the published results[5].

1.3 Synthesis of substrate

synthesis of N-(4-ethynylphenyl)acetamide 5a

4-Ethynylaniline (1.1 mL, 10 mmol) and acetic anhydride (1.2 mL, 13 mmol) were dissolved in dichloromethane (20 mL) and stirred at rt for 12 h. Flash chromatography on silica gel (eluent: petroleumether/ethyl acetate =100/1→5/1) afforded the corresponding alkyne (1.4 g, 90%). See spectrum

^{1}H NMR (400 MHz, Chloroform-d) δ 7.51-7.45 (m, 4H), 7.41 (br, 1H), 3.06 (s, 1H), 2.20 (s, 3H). The data is in accordance with the published results[6].

General procedure for synthesis of alkyne 6a, 16a, 22a, 23a, 24a, 26a[7]

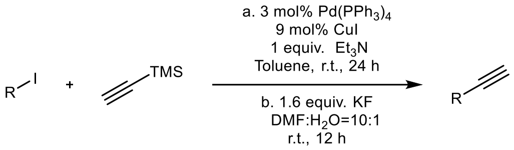

$Pd(PPh_3)_4$ (347 mg, 0.3 mmol), CuI (171 mg, 0.9 mmol), iodide (10 mmol), toluene (25 mL), Et_3N (1.4 mL, 10 mmol) and ethynyltrimethylsilane (2.1 mL, 15 mmol) were added into a flask under nitrogen atmosphere. The resulting solution was stirred for 24 h at rt. Flash chromatography on silica gel (eluent: petroleumether/ethyl acetate =100/1→5/1) afforded the trimethylsilyl alkyne. The obtained alkyne was transfer into a flask with KF (926 mg, 16 mmol), DMF (100 mL), water (10 mL). The reaction mixture was stirred for 12 h at rt. and water (50 mL) was added. The resulting mixture was extracted with ethyl acetate (3×50 mL), the combined organic phase was washed with saturated solution of NaCl and dried with Na2SO4. Flash chromatography on silica gel (eluent: petroleumether/ethyl acetate =100/1→5/1) afforded the corresponding alkyne.

synthesis of 4-ethynylphenyl acetate 6a

The reaction of $Pd(PPh_3)_4$ (140 mg, 0.12 mmol), CuI (69 mg, 0.36 mmol), 4-iodophenyl acetate (1.0 g ,4 mmol), toluene (10 mL), Et_3N (0.6 mL, 4 mmol), ethynyltrimethylsilane (0.8 mL, 6 mmol) and KF (370 mg, 6.4 mmol) afforded **6a** as a white solid (0.6 g, 94%). See spectrum

^{1}H NMR (600 MHz, Chloroform-*d*) δ 7.53 (d, *J* = 8.6 Hz, 2H), 7.08 (d, *J* = 8.6 Hz, 2H), 3.08 (s, 1H), 2.33 (s, 3H). The data is in accordance with the published results[7].

synthesis of methyl 5-ethynyl-2-methoxybenzoate 16a

The reaction of $Pd(PPh_3)_4$ (525 mg, 0.45 mmol), CuI (259 mg, 1.35 mmol), methyl 5-iodo-2-methoxybenzoate (4.4 g, 15 mmol), toluene (38 mL), Et_3N (2.1 mL, 15 mmol), ethynyltrimethylsilane (3.2 mL, 22.5 mmol) and KF (1.4 g, 24 mmol) afforded **16a** as a white solid (1.9 g, 72%). See spectrum

^{1}H NMR (400 MHz, Chloroform-*d*) δ 7.96 (s, 1H), 7.60 (d, *J* = 8.4 Hz, 1H), 6.95 (d, *J* = 8.5 Hz, 1H), 3.94 (s, 3H), 3.91 (s, 3H), 3.04 (s, 1H). The data is in accordance with the published results[8].

synthesis of 1-ethynyl-2-isopropylbenzene 22a

The reaction of $Pd(PPh_3)_4$ (3.5 g, 3 mmol), CuI (1.7 g, 9 mmol), 1-iodo-2-isopropylbenzen (24.6 g, 100 mmol), toluene (250 mL), Et_3N (14 mL, 100 mmol), ethynyltrimethylsilane (21 mL, 150 mmol) and KF (9.3 g, 160 mmol) afforded **22a** as a colorless liquid (10.1 g, 71%). See spectrum

^{1}H NMR (400 MHz, Chloroform-*d*) δ 7.51 (d, *J* = 7.6 Hz, 1H), 7.38 – 7.31 (m, 2H), 7.20 – 7.15 (m, 1H), 3.54 (hept, *J* = 6.9 Hz, 1H), 3.29 (s, 1H), 1.31 (d, *J* = 6.9 Hz, 6H). The data is in accordance with the published results[9].

synthesis of 2-ethynyl-1-fluoro-3-methoxybenzene 23a

The reaction of $Pd(PPh_3)_4$ (266 mg, 0.23 mmol), CuI (129 mg, 0.68 mmol), 1-fluoro-2-iodo-3-methoxybenzene (1.9 g, 7.5 mmol), toluene (20 mL), Et_3N (1.1 mL, 7.5 mmol), ethynyltrimethylsilane (1.5 mL, 11 mmol) and KF (698 mg, 12 mmol) afforded **23a** as a colorless liquid (0.7 g, 62%). See spectrum

^{1}H NMR (600 MHz, Chloroform-*d*) δ 7.31 – 7.27 (m, 1H), 6.75 (t, *J* = 8.5 Hz, 1H), 6.71 (d, *J* = 8.5 Hz, 1H), 3.94 (s, 3H), 3.55 (s, 1H). The data is in accordance with the published results[10].

synthesis of 2-ethynyl-1,3-dimethoxybenzene 24a

The reaction of $Pd(PPh_3)_4$ (150 mg, 0.13 mmol), CuI (72 mg, 0.38 mmol), 2-iodo-1,3-dimethoxybenzene (1.1 g, 4.2 mmol), toluene (10 mL), Et_3N (0.6 mL, 4.2 mmol),

ethynyltrimethylsilane (0.9 mL, 6.3 mmol) and KF (390 mg, 6.7 mmol) afforded **24a** as a white solid (0.3 g, 31%). See spectrum

^{1}H NMR (400 MHz, Chloroform-*d*) δ 7.25 (t, *J* = 8.4 Hz, 1H), 6.54 (d, *J* = 8.4 Hz, 2H), 3.89 (s, 6H), 3.56 (s, 1H). The data is in accordance with the published results[7].

synthesis of 2-ethynyl-1,3-diisopropylbenzene 26a

The reaction of $Pd(PPh_3)_4$ (229 mg, 0.2 mmol), CuI (112 mg, 0.59 mmol), 2-iodo-1,3-diisopropylbenzene (1.9 g, 6.6 mmol), Et_3N (16 mL, 115 mmol), ethynyltrimethylsilane (1.4 mL, 10 mmol) was heated at 90 °C and then react with KF (636 mg, 11 mmol) afforded **23a** as a colorless liquid (460 mg, 38%). See spectrum

^{1}H NMR (400 MHz, Chloroform-*d*) δ 7.33 (t, *J* = 7.8 Hz, 1H), 7.17 (d, *J* = 7.8 Hz, 2H), 3.61 (hept, *J* = 6.9 Hz, 2H), 3.51 (s, 1H), 1.31 (d, *J* = 6.9 Hz, 12H). The data is in accordance with the published results[11].

synthesis of (E)-but-1-en-3-yn-1-ylbenzene 41a

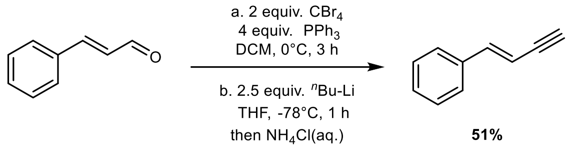

Under a nitrogen atmosphere, carbon tetrabromide (3.8 g, 11.6 mmol) and dichloromethane (10 mL) was added to a solution of triphenylphosphine (6.1 g, 23.2 mmol) in dichloromethane (40 mL) at 0 °C. After 15 min of stirring, a solution of (E)-cinnamaldehyde (0.73 mL, 0.77 g, 5.8 mmol) in dichloromethane (10 mL) was added dropwise and the resulting mixture was stirred for 3 h. After adding petroleum ether (300 mL), the mixture was filtered through a pad of silica. The solvent was evaporated and the obtained crude dibromo compound was used without further purification.

The obtained dibromo compound was dissolved in dry THF (15 mL), and n-butyllithium (5.8 mL, 14.5 mmol, 2.5 M in THF) was added and stirred for 1 h at -78 °C. Saturated solution of NH_4Cl (30 mL) was added and the resulting mixture was extracted with ethyl acetate (3×50 mL). The combined organic layer was evaporated and purified by flash chromatography on silica gel (eluent: petroleumether) afforded **41a** as colorless liquid (0.38 g, 51%). See spectrum

^{1}H NMR (400 MHz, Chloroform-*d*) δ 7.40-7.38 (m, 2H), 7.36 – 7.30 (m, 3H), 7.05 (d, *J* = 16.3 Hz, 1H), 6.14 (dd, *J* = 16.3, 2.3 Hz, 1H), 3.05 (s, 1H). The data is in accordance with the published results[12].

synthesis of (E)-1-(but-1-en-3-yn-1-yl)-4-methylbenzene 42a

The title compound was synthesized using the identical methodology as **41a**. 4-methylcinnamaldehyde (0.8 g, 5.5 mmol) was treated with carbon tetrabromide (3.6 g, 11 mmol) and triphenylphosphine (5.8 g, 22 mmol) in dichloromethane (60 mL) at 0 ℃. The diboromo compound was dissolved in dry THF (15 mL) and n-butyllithium (5.6 mL, 14 mmol, 2.5 M in THF) was added at -78 ℃. Purification by flash chromatography on silica gel (eluent: petroleumether) afforded **42a** as colorless liquid (0.4 g, 53%).

2. General procedure for condition optimization

In a nitrogen atmosphere, a vail was charged with cobalt salt, ligand, and base. The mixture was dissolved in solvent and stirred for 5 min, followed by adding HBpin. The mixture was further stirred for 5 min and phenylacetylene (0.2/0.4 mmol) was added rapidly. The resulting mixture was stirred for a certain time. The reaction was then quenched by adding water. Yield was obtained by

GC-Ms using mesitylene as internal standard or by ^{1}H-NMR using methylene bromide as internal standard.

Supplementary Table 1 Initial screening of ligand

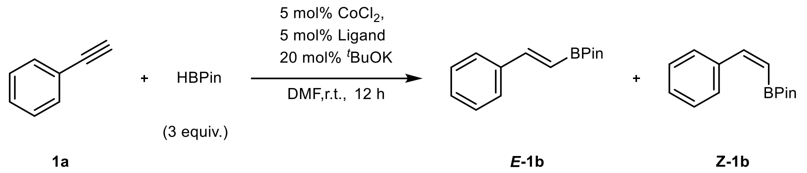

Entry	Ligand	Yield (***E*-1b**: ***Z*-1b**)
1	CNC-H	Trace
2	CNC-Me	71% (97:3)
3	CNC-Et	78% (64:36)
4	CNC-^{i}Pr	99% (98:2)
5	CNC-^{t}Bu	7%
6	CNC-Mes	Trace

Conditions: 5 mol% $CoCl_2$, 5 mol% Ligand, 20 mol% tBuOK, Solvent: DMF, [**1a**] =0.2 M, 3 eq. HBpin, r.t.

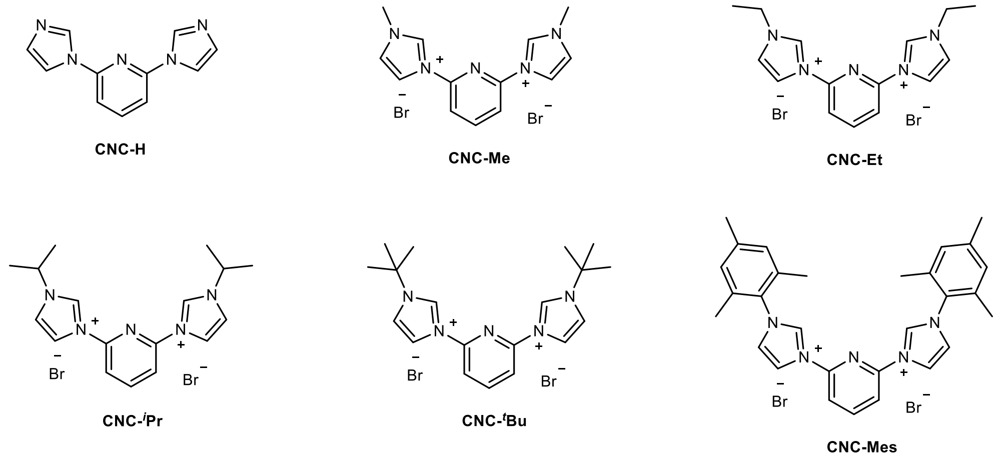

Supplementary Table 2 Initial screening of base

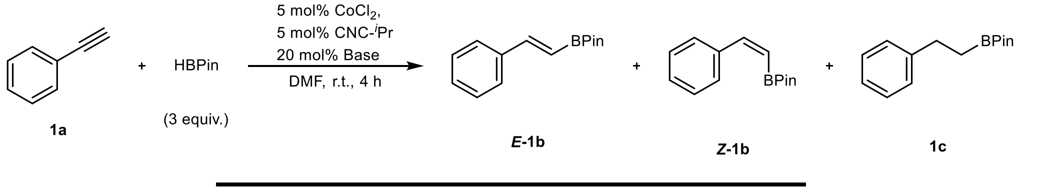

Entry	Base	Yield (**1c**: ***E*-1b**: ***Z*-1b**)
1	^{t}BuOK	>99% (3:62:35)
2	K_2CO_3	0
3	^{t}BuONa	96% (3:88:9)
4	EtONa	Trace
5	MeONa	0
6	NaH	0

Conditions: 5 mol% $CoCl_2$, 5 mol% CNC-^{i}Pr, 20 mol% Base, Solvent: DMF, [**1a**] =0.2 M, 3 eq.

HBpin, r.t.

Supplementary Table 3 Initial screening of time

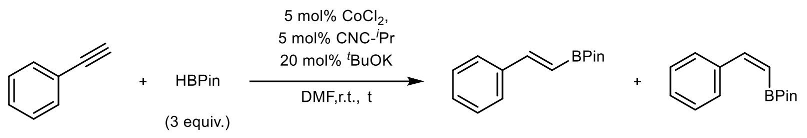

1a E-1b Z-1b

Entry	t	Yield (***E*-1b**: ***Z*-1b**)
1	8 h	>99% (95:5)
2	6 h	99% (95:5)
3	30 min	>99% (51:49)
4	10 min	>99% (4:96)

Condition: 5 mol% $CoCl_2$, 5 mol% CNC-iPr, 20 mol% ^{t}BuOK, Solvent: DMF, [**1a**]=0.2 M, 3 eq. HBpin, r.t.

Supplementary Table 4 Initial screening of equivalent of HBpin

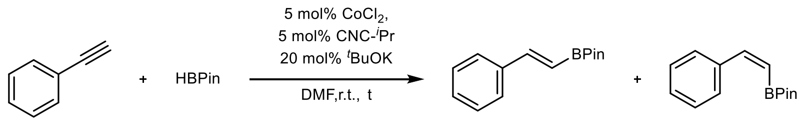

1a E-1b Z-1b

Entry	HBPin	t	Yield (***E*-1b**: ***Z*-1b**)
1	1.5 eq.	10 min	65% (<1:99)
2	1.5 eq.	24 h	70% (<1:99)
3	2 eq.	10 min	87% (<1:99)
4	2 eq.	24 h	95% (66:34)
5	3 eq.	10 min	>99% (4:96)
6	3 eq.	12 h	99% (98:2)

Conditions: 5 mol% $CoCl_2$, 5 mol% CNC-^{i}Pr, 20 mol% ^{t}BuOK, Solvent: DMF, [**1a**] =0.2 M, HBpin, r.t.

Supplementary Table 5 Screening of different ligand in low catalyst loading.

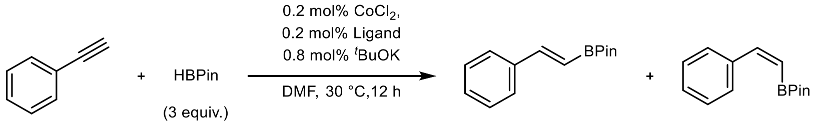

1a E-1b Z-1b

Entry	Ligand	***E*-1b**	***Z*-1b**
1	CNC-H	Trace	Trace
2	CNC-Me	Trace	Trace
3	CNC-Et	Trace	5%
4	CNC-^{i}Pr	Trace	30%
5	CNC-^{t}Bu	Trace	Trace

6	CNC-Mes	Trace	Trace

Conditions: 0.2 mol% $CoCl_2$, 0.2 mol% ligand, 0.8 mol% base, solvent: DMF, [**1a**] =0.2 M, 3 eq. HBpin, t = 12 h, 30 ℃.

Supplementary Table 6 Screening of reaction concentration

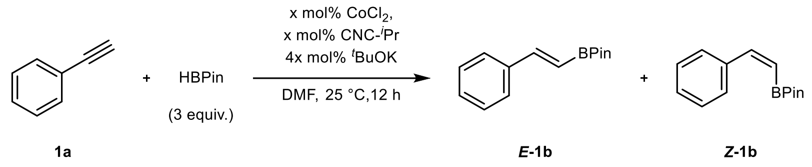

Entry	Metal	[**1a**]	***E*-1b**	***Z*-1b**
1	0.4 mol% $CoCl_2$	0.05 M	Trace	7%
2	0.4 mol% $CoCl_2$	0.1 M	4%	96%
3	0.4 mol% $CoCl_2$	0.2 M	76%	24%
4	0.4 mol% $CoCl_2$	0.4 M	97%	4%
5	0.2 mol% $CoCl_2$	0.05 M	Trace	Trace
6	0.2 mol% $CoCl_2$	0.1 M	Trace	Trace
7	0.2 mol% $CoCl_2$	0.2 M	Trace	8%
8	0.2 mol% $CoCl_2$	0.3 M	Trace	22%
9	0.2 mol% $CoCl_2$	0.4 M	Trace	44%
10[a]	0.5mol% $CoCl_2$	neat	Trace	Trace
11[b]	0.5 mol% $CoCl_2$	neat	Trace	Trace

Conditions: x mol% $CoCl_2$, x mol% **CNC-^{i}Pr**, 4x mol% **^{t}BuOK**, Solvent: DMF, 3 eq. HBpin, t= 12 h, 25 ℃. a: 200 µl of DMF to dissolve the Cat., 4 mmol scale; b: 200 µl of DMF to dissolve the Cat., 10 mmol scale.

Supplementary Table 7 Screening of solvent

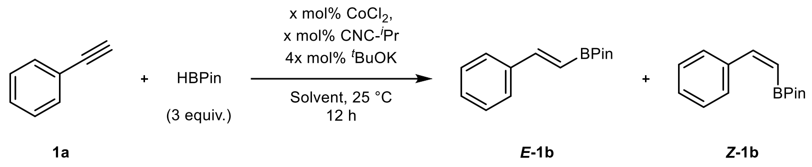

Entry	Metal	Solvent	***E*-1b**	***Z*-1b**
1	0.2 mol% $CoCl_2$	DMA	Trace	9%
2	0.2 mol% $CoCl_2$	NMP	Trace	6%
3	0.2 mol% $CoCl_2$	DMSO	Trace	Trace
4	0.2 mol% $CoCl_2$	DMF	Trace	44%
5	0.3 mol% $CoCl_2$	DMA	Trace	17%
6	0.3 mol% $CoCl_2$	NMP	Trace	13%
7	0.3 mol% $CoCl_2$	DMSO	Trace	Trace
8	0.3 mol% $CoCl_2$	DMF	Trace	58%

Conditions: x mol% $CoCl_2$, x mol% **CNC-^{i}Pr**, 4x mol% **^{t}BuOK**, Solvent, [**1a**] =0.4 M, 3 eq. HBpin, t = 12 h, 25 ℃.

Supplementary Table 8 Screening of Metal Precursor

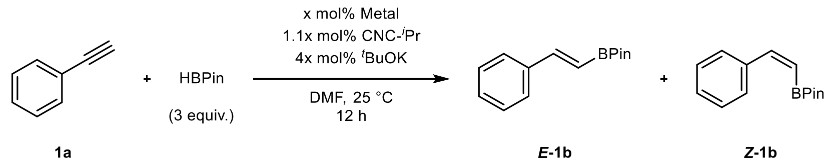

Entry	Metal	***E*-1b**	***Z*-1b**
1	0.3 mol% $CoCl_2$	Trace	91%
2	0.2 mol% $CoCl_2$	Trace	27%
3	0.4 mol% $CoBr_2$	Trace	48%
4	0.3 mol% $CoBr_2$	Trace	35%
5	0.3 mol% $Co(acac)_2$	52%	46%
6	0.2 mol% $Co(acac)_2$	48%	50%
7	0.1 mol% $Co(acac)_2$	Trace	50%
8	0.05 mol% $Co(acac)_2$	Trace	Trace
9	0.3 mol% $Co(OAc)_2$	50%	52%
10	0.2 mol% $Co(OAc)_2$	14%	83%
11	0.1 mol% $Co(OAc)_2$	Trace	45%
12	0.05 mol% $Co(OAc)_2$	Trace	12%

Conditions: x mol% Metal, 1.1x mol% **CNC-^{i}Pr**, 4x mol% **^{t}BuOK**, Solvent: DMF, [**1a**] =0.4 M, 3 eq. HBpin, t= 12 h, 25 °C.

Supplementary Table 9 Screening of equivalent of Ligand

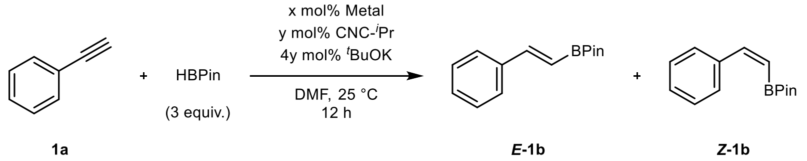

Entry	Metal	Ligand (eq. of Metal)	***E*-1b**	***Z*-1b**
1	0.2 mol% $Co(acac)_2$	0.5 eq.	Trace	22%
2	0.2 mol% $Co(acac)_2$	1 eq.	Trace	45%
3	0.2 mol% $Co(acac)_2$	1.3 eq.	54%	46%
4	0.2 mol% $Co(acac)_2$	1.4 eq.	86%	8%
5	0.2 mol% $Co(acac)_2$	1.5 eq.	71%	26%
6	0.2 mol% $Co(acac)_2$	1.6 eq.	54%	38%
7	0.2 mol% $Co(acac)_2$	2 eq.	43%	56%
8	0.1 mol% $Co(acac)_2$	0.5 eq.	Trace	6%
9	0.1 mol% $Co(acac)_2$	1 eq.	Trace	8%
10	0.1 mol% $Co(acac)_2$	1.3 eq.	8%	78%
11	0.1 mol% $Co(acac)_2$	1.4 eq.	5%	89%
12	0.1 mol% $Co(acac)_2$	1.5 eq.	4%	48%
13	0.1 mol% $Co(acac)_2$	1.6 eq.	6%	35%

14	0.1 mol% $Co(acac)_2$	2 eq.	Trace	26%

Conditions: x mol% $Co(acac)_2$, y mol% **CNC-iPr**, 4y mol% ***t*BuOK**, Solvent: DMF, [**1a**] =0.4 M, 3 eq. HBpin, t = 12 h, 25 ℃.

Supplementary Table 10 Screening of concentration using $Co(acac)_2$

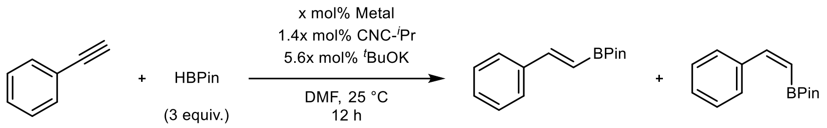

1a E-1b Z-1b

Entry	Metal	[**1a**]	***E*-1b**	***Z*-1b**
1	0.1 mol% $Co(acac)_2$	0.8 M	5%	95%
2	0.1 mol% $Co(acac)_2$	1.0 M	Trace	93%
3	0.1 mol% $Co(acac)_2$	1.6 M	Trace	64%
4	0.05 mol% $Co(acac)_2$	0.8 M	Trace	16%
5	0.05 mol% $Co(acac)_2$	1.6 M	Trace	14%

Conditions: x mol% $Co(acac)_2$, 1.4x mol% **CNC-*i*Pr**, 5.6x mol% ***t*BuOK**, Solvent: DMF, 3 eq. HBpin, t = 12 h, 25 ℃.

Supplementary Table 11 Screening of equivalent of Base

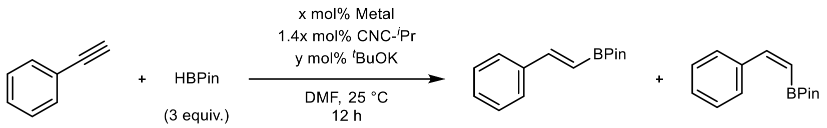

1a E-1b Z-1b

Entry	Metal	*t*BuOK (eq. of Ligand)	***E*-1b**	***Z*-1b**
1	0.05 mol% $Co(acac)_2$	4 eq.	Trace	16%
2	0.05 mol% $Co(acac)_2$	3 eq.	Trace	Trace
3	0.05 mol% $Co(acac)_2$	2.5 eq.	Trace	Trace
4	0.05 mol% $Co(acac)_2$	2 eq.	Trace	Trace
5	0.08 mol% $Co(acac)_2$	4 eq.	Trace	48%
6	0.08 mol% $Co(acac)_2$	3 eq.	Trace	36%
7	0.08 mol% $Co(acac)_2$	2.5 eq.	Trace	15%
8	0.08 mol% $Co(acac)_2$	2 eq.	Trace	Trace

Conditions: x mol% $Co(acac)_2$, 1.4x mol% **CNC-iPr**, y mol% ***t*BuOK**, Solvent: DMF, [**1a**] =0.8 M, 3 eq. HBpin, t = 12 h, 25 ℃.

Supplementary Table 12 Screening of other boranes

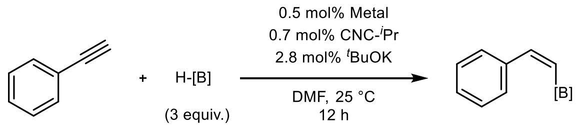

1a

Entry	H-[B]	Yield
1	HBCat	0

2	HBdan	0
3	9-BBN	0

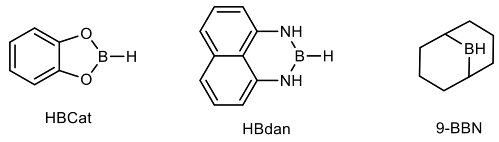

3. Substrate investigation and characterization

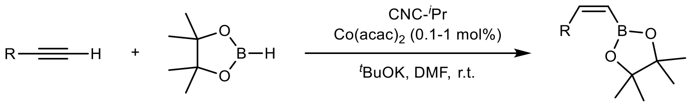

General procedure A: In a nitrogen atmosphere, a vail was charged with Co(acac)$_2$ (4.1 mg, 0.016 mmol), CNC-^{i}Pr (10.2 mg, 0.022 mmol), ^{t}BuOK (10.1 mg, 0.09 mmol) in dry DMF (2 mL) and was stirred for 5 mins. The freshly prepared stock solution of the *in situ* prepared active catalyst ([Co] = 8.0 mM in DMF) was added via a micro-syringe (50-500 µL, 0.0004-0.004 mmol, 0.1-1 mol%, as noted) to a vial charged with HBpin (75µL, 0.52 mmol, 1.3 equiv., unless otherwise noted) and DMF([alkyne]=0.8 M). The mixture was stirred for 5 mins. Alkyne (0.4 mmol, unless otherwise noted) was added rapidly and the resulting mixture was stirred for 12 hours. The reaction was then quenched by adding water. 20 mL EtOAc was added and the organic phase was washed with 10 mL brine twice to remove most of DMF. Pure product was isolated by column chromatography over silica gel deactivated with 2% NEt$_3$ in petroleum ether using petroleum/EtOAc as the eluent.

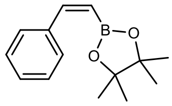
1b

(Z)-4,4,5,5-tetramethyl-2-styryl-1,3,2-dioxaborolane (**1b**): The title compound was prepared following general procedure A (0.1 mol% of [Co], 0.8 mmol scale). Dibromomethane (DBM) was added as internal standard and NMR yield (>99%) was obtained. Purification by flash column chromatography on silica gel (eluent: petroleumether→ petroleumether/EtOAc =20/1) afforded the product as a colorless oil in 90% yield (166.5 mg, Z:E = 98:2). See spectrum
The title compound can also be synthesized according to general procedure A (0.5 mol% of [Co]), resulting in a >99% NMR yield (Z:E=98:2) when dibromomethane was used as the internal standard.
^{1}H NMR (400 MHz, Chloroform-*d*) δ 7.53 (d, *J* = 6.7 Hz, 2H), 7.33 – 7.26 (m, 3H), 7.22 (d, *J* = 15.0 Hz, 1H), 5.59 (d, *J* = 14.9 Hz, 1H), 1.29 (s, 12H).
^{11}B NMR (128 MHz, CDCl$_3$) δ 29.4.
All recorded spectroscopic data matched those previously reported in the literature[13].

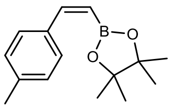
2b

(Z)-4,4,5,5-tetramethyl-2-(4-methylstyryl)-1,3,2-dioxaborolane (**2b**): The title compound was prepared following general procedure A (1 mol% of [Co]). DBM was added as internal standard and NMR yield (93%) was obtained. Purification by flash column chromatography on silica gel (eluent:

petroleumether→ petroleumether/EtOAc =20/1) afforded the product as a pale-yellow oil in 90% yield (87.8 mg, Z:E = 99:1). See spectrum

^{1}H NMR (400 MHz, Chloroform-*d*) δ 7.46 (d, *J* = 7.6 Hz, 2H), 7.19 (d, *J* = 14.9 Hz, 1H), 7.12 (d, *J* = 7.7 Hz, 2H), 5.53 (d, *J* = 14.9 Hz, 1H), 2.35 (s, 3H), 1.31 (s, 12H).

All recorded spectroscopic data matched those previously reported in the literature[13].

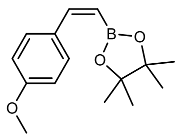

3b

(Z)-2-(4-methoxystyryl)-4,4,5,5-tetramethyl-1,3,2-dioxaborolane (**3b**): The title compound was prepared following general procedure A (0.1 mol% of [Co]), the reaction mixture was stirred for 3 hours. Purification by flash column chromatography on silica gel (eluent: petroleumether→ petroleumether/EtOAc =20/1) afforded the product as a colorless oil in 99% yield (99.9 mg, Z:E=98:2)). See spectrum

The title compound can also be synthesized according to general procedure A (0.5 mol% of [Co]), resulting in a >99% NMR yield (Z:E=97:3) when dibromomethane was used as the internal standard.

Reaction with low catalyst loading: In a nitrogen atmosphere, a vail was charged with $Co(acac)_2$ (4.1 mg, 0.016 mmol), CNC-^{i}Pr (10.2 mg, 0.022 mmol), ^{t}BuOK (10.1 mg, 0.09 mmol) in dry DMF (2 mL) and was stirred for 5 mins. The freshly prepared stock solution of the *in situ* prepared active catalyst ([Co] = 8.0 mM in DMF) was added via a micro-syringe (0.05 mol%) to a vial charged with HBpin (1.3 equiv.) and DMF (0.5 mL, [alkyne]=3.2 M). The mixture was stirred for 5 mins. Alkyne (1.6 mmol) was added rapidly and the resulting mixture was stirred for 24 or 48 hours. The resulting mixture was diluted with EtOAc and sent for GC-MS analysis, leading to the title compound with a 77% GC-MS yield (Z:E>99:1, 24 h) and 84% GC-MS yield (Z:E>99:1, 48 h), respectively.

^{1}H NMR (400 MHz, Chloroform-*d*) δ 7.57 (d, *J* = 8.7 Hz, 2H), 7.18 (d, *J* = 14.9 Hz, 1H), 6.86 (d, *J* = 8.8 Hz, 2H), 5.48 (d, *J* = 14.9 Hz, 1H), 3.84 (s, 3H), 1.33 (s, 12H).

^{11}B NMR (193 MHz, $CDCl_3$) δ 30.4.

All recorded spectroscopic data matched those previously reported in the literature[13].

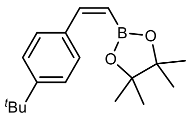

4b

(Z)-2-(4-(tert-butyl)styryl)-4,4,5,5-tetramethyl-1,3,2-dioxaborolane (**4b**): The title compound was prepared following general procedure A (1 mol% of [Co]). Purification by flash column chromatography on silica gel (eluent: petroleumether→ petroleumether/EtOAc =20/1) afforded the product as a pale-yellow oil in 82% yield (91.8 mg, Z:E=99:1)). See spectrum

The title compound can also be synthesized according to general procedure A (0.5 mol% of [Co],), resulting in a 86% NMR yield, Z:E=98:2) when dibromomethane was used as the internal standard.

^{1}H NMR (400 MHz, Chloroform-*d*) δ 7.54 (d, *J* = 8.1 Hz, 2H), 7.36 (d, *J* = 8.2 Hz, 2H), 7.20 (d, *J* = 14.9 Hz, 1H), 5.55 (d, *J* = 14.9 Hz, 1H), 1.34 (s, 9H), 1.33 (s, 12H).

All recorded spectroscopic data matched those previously reported in the literature[13].

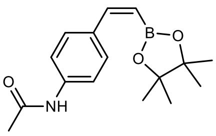

5b

(Z)-N-(4-(2-(4,4,5,5-tetramethyl-1,3,2-dioxaborolan-2-yl)vinyl)phenyl)acetamide (**5b**): The title compound was prepared following general procedure A (0.5 mol% of [Co]). **5a** (63.7 mg, 0.4 mmol) was added into a vail with Co(acac)$_2$ (0.5 mg, 0.002 mmol), CNC-^{i}Pr (1.3 mg, 0.0028 mmol), ^{t}BuOK (1.3 mg, 0.0112 mmol), HBpin (75μL, 0.52 mmol, 1.3 equiv.) and DMF (0.5 mL). The reaction mixture was stirred for 10 minutes. DBM was added as internal standard and NMR yield (>99%) was obtained. Purification by flash column chromatography on silica gel (eluent: petroleumether→ petroleumether/EtOAc =10/1) afforded the product as a white solid in 87% yield (99.7 mg, Z:E=97:3)). See spectrum

^{1}H NMR (400 MHz, Chloroform-*d*) δ 7.53 (d, *J* = 8.6 Hz, 2H), 7.45 (d, *J* = 8.6 Hz, 2H), 7.30 (s, 1H), 7.14 (d, *J* = 14.9 Hz, 1H), 5.52 (d, *J* = 14.9 Hz, 1H), 2.17 (s, 3H), 1.29 (s, 12H).

^{13}C NMR (101 MHz, CDCl$_3$) δ 168.2, 147.7, 137.8, 134.5, 129.6, 119.0, 83.6, 24.8, 24.8.

^{11}B NMR (128 MHz, CDCl$_3$) δ 29.5.

HRMS (ESI+) *m*/*z* calculated for C16H22BNO3Na$^+$ ([M+Na]+) 310.1585; found 310.1582.

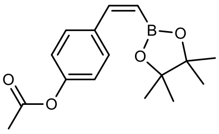

6b

(Z)-4-(2-(4,4,5,5-tetramethyl-1,3,2-dioxaborolan-2-yl)vinyl)phenyl acetate (**6b**): The title compound was prepared following general procedure A (0.5 mol% of [Co]). **6a** (64.1 mg, 0.4 mmol) was added into a vail with Co(acac)$_2$ (0.5 mg, 0.002 mmol), CNC-^{i}Pr (1.3 mg, 0.0028 mmol), ^{t}BuOK (1.3 mg, 0.0112 mmol), HBpin (75μL, 0.52 mmol, 1.3 equiv.) and DMF (0.5 mL). The reaction mixture was stirred for 10 minutes. Purification by flash column chromatography on silica gel (eluent: petroleumether→ petroleumether/EtOAc =10/1) afforded the product as a white solid in 88% yield (101.6 mg, Z:E>99:1)). See spectrum

^{1}H NMR (400 MHz, Chloroform-*d*) δ 7.58 (d, *J* = 8.5 Hz, 2H), 7.18 (d, *J* = 14.9 Hz, 1H), 7.03 (d, *J* = 8.6 Hz, 2H), 5.58 (d, *J* = 14.9 Hz, 1H), 2.29 (s, 3H), 1.28 (s, 12H).

^{13}C NMR (101 MHz, CDCl$_3$) δ 169.4, 150.4, 147.3, 136.2, 129.8, 121.0, 83.6, 24.8, 21.2.

^{11}B NMR (193 MHz, CDCl$_3$) δ 30.2.

HRMS (ESI+) *m*/*z* calculated for C16H22BO4+ ([M+H]+) 289.1606; found 289.1603.

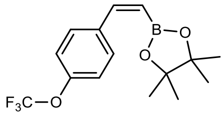

7b

(Z)-4,4,5,5-tetramethyl-2-(4-(trifluoromethoxy)styryl)-1,3,2-dioxaborolane (**7b**): The title compound was prepared following general procedure A (1 mol% of [Co]). **7a** (74.5 mg, 0.4 mmol) was added into a vail with Co(acac)$_2$ (1.0 mg, 0.004 mmol), CNC-^{i}Pr (2.6 mg, 0.0056 mmol), ^{t}BuOK (2.5 mg, 0.0224 mmol), HBpin (75μL, 0.52 mmol, 1.3 equiv.) and DMF (0.5 mL). The reaction mixture was stirred for 12 hours. Purification by flash column chromatography on silica gel (eluent: petroleumether→ petroleumether/EtOAc =20/1) afforded the product as a yellow oil in 83% yield (104.3 mg, Z:E>99:1)). See spectrum

The title compound can also be synthesized according to general procedure A (0.5 mol% of [Co]), resulting in a 70% NMR yield (Z:E=96:4) when dibromomethane was used as the internal standard.
^{1}H NMR (600 MHz, Chloroform-*d*) δ 7.58 (d, *J* = 8.5 Hz, 2H), 7.18 (d, *J* = 14.9 Hz, 1H), 7.15 (d, *J* = 8.3 Hz, 2H), 5.64 (d, *J* = 14.9 Hz, 1H), 1.29 (s, 12H).
^{19}F NMR (565 MHz, $CDCl_3$) δ -57.8.
^{13}C NMR (151 MHz, $CDCl_3$) δ 148.8, 146.7, 137.1, 130.1, 123.0, 121.3, 120.3, 119.6, 117.9, 83.6, 24.7.
^{11}B NMR (193 MHz, $CDCl_3$) δ 30.1.
HRMS (APCI+) *m*/*z* calculated for C15H19BF3O3+ ([M+H]+) 315.1374; found 315.1366.

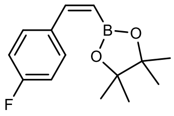
8b

(Z)-2-(4-fluorostyryl)-4,4,5,5-tetramethyl-1,3,2-dioxaborolane (**8b**): The title compound was prepared following general procedure A (1 mol% of [Co]), the reaction mixture was stirred for 10 minutes. DBM was added as internal standard and NMR yield (81%) was obtained. Purification by flash column chromatography on silica gel (eluent: petroleumether→ petroleumether/EtOAc =20/1) afforded the product as a colorless oil in 67% yield (76.6 mg, Z:E=96:4). *See spectrum*
^{1}H NMR (400 MHz, Chloroform-*d*) δ 7.60 – 7.49 (m, 2H), 7.17 (d, *J* = 14.8 Hz, 1H), 6.99 (t, *J* = 8.8 Hz, 2H), 5.56 (d, *J* = 14.9 Hz, 1H), 1.29 (s, 12H).
^{13}C NMR (101 MHz, $CDCl_3$) δ 163.8, 161.4, 147.2, 134.6, 134.6, 130.5, 130.4, 114.9, 114.7, 83.5, 24.8.
^{11}B NMR (128 MHz, $CDCl_3$) δ 30.3.
^{19}F NMR (377 MHz, $CDCl_3$) δ -113.9.
All recorded spectroscopic data matched those previously reported in the literature[13].

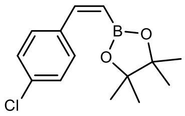
9b

(Z)-2-(4-chlorostyryl)-4,4,5,5-tetramethyl-1,3,2-dioxaborolane (**9b**): The title compound was prepared following general procedure A (1 mol% of [Co], 2.5 eq. HBpin, 0.2 mmol scale), the reaction mixture was stirred for 12 hours. DBM was added as internal standard and NMR yield (84%) was obtained. Purification by flash column chromatography on silica gel (eluent: petroleumether→ petroleumether/EtOAc =20/1) afforded the product as a colorless oil in 74% yield (40.3 mg, Z:E=97:3). *See spectrum*
The title compound can also be synthesized according to general procedure A (0.5 mol% of [Co]), resulting in a 73% NMR yield (Z:E=98:2) when dibromomethane was used as the internal standard.
^{1}H NMR (400 MHz, Chloroform-*d*) δ 7.41 (d, *J* = 8.5 Hz, 2H), 7.20 (d, *J* = 8.6 Hz, 2H), 7.07 (d, *J* = 14.9 Hz, 1H), 5.54 (d, *J* = 14.9 Hz, 1H), 1.21 (s, 12H).
All recorded spectroscopic data matched those previously reported in the literature[13].

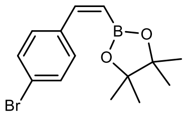
10b

(Z)-2-(4-bromostyryl)-4,4,5,5-tetramethyl-1,3,2-dioxaborolane (**10b**): The title compound was prepared following general procedure A (1 mol% of [Co], 2.5 eq. HBpin), the reaction mixture was stirred for 12hours. DBM was added as internal standard and NMR yield (77%) was obtained. Purification by flash column chromatography on silica gel (eluent: petroleumether→ petroleumether/EtOAc =20/1) afforded the product as a pale yellow oil in 66% yield (82.3 mg, Z:E>99:1). See spectrum

The title compound can also be synthesized according to general procedure A (0.5 mol% of [Co]), resulting in a 73% NMR yield (Z:E=98:2) when dibromomethane was used as the internal standard. Purification by flash column chromatography on silica gel (eluent: petroleumether→ petroleumether/EtOAc =20/1) afforded the product as a pale yellow oil in 63% yield (78.1 mg, Z:E>99:1).

^{1}H NMR (400 MHz, Chloroform-*d*) δ 7.42 (s, 4H), 7.13 (d, *J* = 14.9 Hz, 1H), 5.62 (d, *J* = 14.9 Hz, 1H), 1.29 (s, 12H).

^{13}C NMR (151 MHz, $CDCl_3$) δ 147.0, 137.3, 131.1, 130.3, 122.1, 83.6, 24.8.

^{11}B NMR (193 MHz, $CDCl_3$) δ 30.2.

All recorded spectroscopic data matched those previously reported in the literature[14].

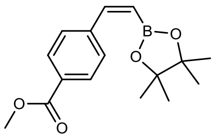

11b

Methyl (Z)-4-(2-(4,4,5,5-tetramethyl-1,3,2-dioxaborolan-2-yl)vinyl)benzoate (**11b**): The title compound was prepared following general procedure A (1 mol% of [Co], 3 eq. HBpin). DBM was added as internal standard and NMR yield (86%) was obtained. Purification by flash column chromatography on silica gel (eluent: petroleumether→ petroleumether/EtOAc =10/1) afforded the product as a white solid in 76% yield (87.6 mg, Z:E>99:1)). See spectrum

^{1}H NMR (600 MHz, Chloroform-*d*) δ 7.98 (d, *J* = 7.6 Hz, 2H), 7.58 (d, *J* = 7.7 Hz, 2H), 7.23 (d, *J* = 14.9 Hz, 1H), 5.72 (d, *J* = 14.9 Hz, 1H), 3.91 (s, 3H), 1.29 (s, 12H).

^{13}C NMR (151 MHz, $CDCl_3$) δ 167.0, 146.9, 142.9, 129.3, 129.3, 128.5, 83.7, 52.1, 24.8.

^{11}B NMR (193 MHz, $CDCl_3$) δ 30.3.

All recorded spectroscopic data matched those previously reported in the literature[13].

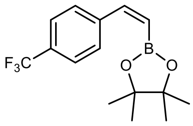

12b

(Z)-4,4,5,5-tetramethyl-2-(4-(trifluoromethyl)styryl)-1,3,2-dioxaborolane (**12b**): The title compound was prepared following general procedure A (1 mol% of [Co], 3 eq. HBpin) , the reaction mixture was stirred with 10 mol% diphenylacetylene for 12 hours. DBM was added as internal standard and NMR yield (42%) was obtained. Purification by flash column chromatography on silica gel (eluent: petroleumether→ petroleumether/EtOAc =20/1) afforded the product as a colorless oil in 36% yield (42.9 mg, Z:E=99:1)). See spectrum

The title compound can also be synthesized according to general procedure A (0.5 mol% of [Co]), resulting in a 16% NMR yield (Z:E=88:12) when dibromomethane was used as the internal standard. When conditions were modified to 1.0 mol% of [Co], an NMR yield of 50% with a Z:E ratio of

83:17 was obtained. Furthermore, by employing a condition consisting of 1.0 mol% of [Co], 3 eq. HBpin, an NMR yield of 80% with a Z:E ratio of 75:25 was achieved.

^{1}H NMR (600 MHz, Chloroform-*d*) δ 7.63 (d, *J* = 8.1 Hz, 2H), 7.58 – 7.53 (d, *J* = 8.2 Hz, 2H), 7.22 (d, *J* = 14.8 Hz, 1H), 5.73 (d, *J* = 14.9 Hz, 1H), 1.29 (s, 12H).
^{13}C NMR (151 MHz, $CDCl_3$) δ 146.6, 141.8, 129.8, 129.6, 128.8, 124.9, 124.9, 124.9, 124.8, 83.7, 24.8.
^{11}B NMR (193 MHz, $CDCl_3$) δ 30.1.
^{19}F NMR (565 MHz, $CDCl_3$) δ -62.5.
All recorded spectroscopic data matched those previously reported in the literature[14].

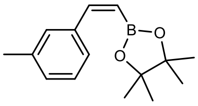

13b

(Z)-4,4,5,5-tetramethyl-2-(3-methylstyryl)-1,3,2-dioxaborolane (**13b**): The title compound was prepared following general procedure A (0.2 mol% of [Co]). DBM was added as internal standard and NMR yield (99%) was obtained. Purification by flash column chromatography on silica gel (eluent: petroleumether→ petroleumether/EtOAc =20/1) afforded the product as a colorless oil in 80% yield (78.5 mg, Z:E>99:1)). *See spectrum*
The title compound can also be synthesized according to general procedure A (0.5 mol% of [Co]), resulting in a >99% NMR yield (Z:E>99:1) when dibromomethane was used as the internal standard.
^{1}H NMR (400 MHz, Chloroform-*d*) δ 7.31 (s, 1H), 7.22 (d, *J* = 7.4 Hz, 1H), 7.15-7.06 (m, 2H), 7.04 – 6.96 (m, 1H), 5.49 (d, *J* = 14.8 Hz, 1H), 2.26 (s, 3H), 1.21 (s, 12H).
^{13}C NMR (151 MHz, $CDCl_3$) δ 148.2, 138.4, 137.4, 129.1, 128.8, 127.9, 125.9, 83.4, 24.8, 21.3.
^{11}B NMR (193 MHz, $CDCl_3$) δ 30.4.
All recorded spectroscopic data matched those previously reported in the literature[14].

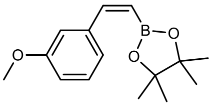

14b

(Z)-2-(3-methoxystyryl)-4,4,5,5-tetramethyl-1,3,2-dioxaborolane (**14b**): The title compound was prepared following general procedure A (1 mol% of [Co],2 eq. HBpin). Purification by flash column chromatography on silica gel (eluent: petroleumether→ petroleumether/EtOAc =20/1) afforded the product as a pale yellow oil in 91% yield (97.1 mg, Z:E=97:3)). *See spectrum*
The title compound can also be synthesized according to general procedure A (0.5 mol% of [Co]), resulting in a 56% NMR yield (Z:E=95:5) when dibromomethane was used as the internal standard.
^{1}H NMR (400 MHz, Chloroform-*d*) δ 7.25 (s, 1H), 7.14 – 7.05 (m, 2H), 6.96 (d, *J* = 7.5 Hz, 1H), 6.73 (d, *J* = 8.1 Hz, 1H), 5.50 (d, *J* = 15.0 Hz, 1H), 3.72 (s, 3H), 1.19 (s, 12H).
All recorded spectroscopic data matched those previously reported in the literature[13].

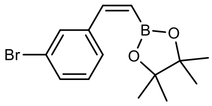

15b

(Z)-2-(3-bromostyryl)-4,4,5,5-tetramethyl-1,3,2-dioxaborolane (**15b**): The title compound was prepared following general procedure A (1 mol% of [Co],2 eq. HBpin). **15a** (72.4 mg, 0.4 mmol) was added into a vail with $Co(acac)_2$ (1.0 mg, 0.004 mmol), CNC-^{i}Pr (2.6 mg, 0.0056 mmol), ^{t}BuOK (2.5 mg, 0.0224 mmol), HBpin (115 μL, 0.8 mmol, 2 equiv.) and DMF (0.5 mL). The reaction mixture was stirred for 24 hours. Purification by flash column chromatography on silica gel (eluent: petroleumether→ petroleumether/EtOAc =20/1) afforded the product as a yellow oil in 61% yield (74.7 mg, Z:E=99:1)). See spectrum

^{1}H NMR (400 MHz, Chloroform-*d*) δ 7.82 (s, 1H), 7.37 (dd, *J* = 12.8, 7.9 Hz, 2H), 7.21 – 7.09 (m, 2H), 5.66 (d, *J* = 14.8 Hz, 1H), 1.31 (s, 12H).

^{13}C NMR (151 MHz, $CDCl_3$) δ 146.5, 140.5, 131.2, 130.8, 129.5, 127.6, 122.2, 83.7, 24.9.

^{11}B NMR (193 MHz, $CDCl_3$) δ 30.1.

HRMS (APCI+) *m*/*z* calculated for C14H19BBrO2+ ([M+H]+) 309.0656; found 309.0651.

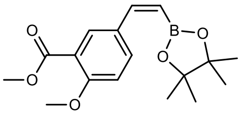

16b

methyl (Z)-2-methoxy-5-(2-(4,4,5,5-tetramethyl-1,3,2-dioxaborolan-2-yl)vinyl)benzoate (**16b**): The title compound was prepared following general procedure A (1 mol% of [Co]). **16a** (76.1 mg, 0.4 mmol) was added into a vail with $Co(acac)_2$ (1.0 mg, 0.004 mmol), CNC-^{i}Pr (2.6 mg, 0.0056 mmol), ^{t}BuOK (2.5 mg, 0.0224 mmol), HBpin (75 μL, 0.52 mmol, 1.3 equiv.) and DMF (0.5 mL). The reaction mixture was stirred for 12 hours. DBM was added as internal standard and NMR yield (99%) was obtained. Purification by flash column chromatography on silica gel (eluent: petroleumether→ petroleumether/EtOAc =10/1) eluent afforded the product as a white solid in 95% yield (120.3 mg, Z:E=99:1)). See spectrum

^{1}H NMR (600 MHz, Chloroform-*d*) δ 8.13 (d, *J* = 2.3 Hz, 1H), 7.64 (dd, *J* = 8.6, 2.3 Hz, 1H), 7.13 (d, *J* = 14.9 Hz, 1H), 6.91 (d, *J* = 8.7 Hz, 1H), 5.52 (d, *J* = 14.9 Hz, 1H), 3.90 (s, 3H), 3.87 (s, 3H), 1.29 (s, 12H).

^{13}C NMR (151 MHz, $CDCl_3$) δ 166.4, 159.0, 147.1, 134.2, 132.1, 130.7, 119.5, 111.4, 83.5, 56.1, 51.8, 24.8.

^{11}B NMR (193 MHz, $CDCl_3$) δ 30.3.

HRMS (ESI+) *m*/*z* calculated for C17H24BO5+ ([M+H]+) 319.1711; found 3191710.

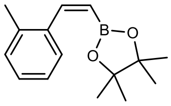

17b

(Z)-4,4,5,5-tetramethyl-2-(2-methylstyryl)-1,3,2-dioxaborolane (**17b**): The title compound was prepared following general procedure A (0.2 mol% of [Co]). DBM was added as internal standard and NMR yield (92%) was obtained. Purification by flash column chromatography on silica gel (eluent: petroleumether→ petroleumether/EtOAc =20/1) afforded the product as a pale yellow oil in 74% yield (74.7 mg, Z:E>99:1)). See spectrum

The title compound can also be synthesized according to general procedure A (0.5 mol% of [Co]), resulting in a >99% NMR yield (Z:E>99:1) when dibromomethane was used as the internal standard.

^{1}H NMR (400 MHz, Chloroform-*d*) δ 7.46 – 7.33 (m, 2H), 7.23-7.09 (m, 3H), 5.72 – 5.67 (d, *J* = 14.8, 1H), 2.33 (s, 3H), 1.24 (s, 12H).

All recorded spectroscopic data matched those previously reported in the literature[13].

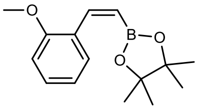

18b

(Z)-2-(2-methoxystyryl)-4,4,5,5-tetramethyl-1,3,2-dioxaborolane (**18b**): The title compound was prepared following general procedure A (0.1 mol% of [Co], 0.8 mmol scale). DBM was added as internal standard and NMR yield (95%) was obtained. Purification by flash column chromatography on silica gel (eluent: petroleumether→ petroleumether/EtOAc =20/1) afforded the product as a pale yellow oil in 89% yield (185.7 mg, Z:E=98:2)). See spectrum

The title compound can also be synthesized according to general procedure A (0.5 mol% of [Co]), resulting in a 95% NMR yield (Z:E=99:1) when dibromomethane was used as the internal standard.

^{1}H NMR (400 MHz, Chloroform-*d*) δ 7.52 (d, *J* = 7.6 Hz, 1H), 7.48 (d, *J* = 14.9 Hz, 1H), 7.25 (t, *J* = 7.7 Hz, 1H), 6.90 – 6.81 (m, 2H), 5.61 (d, *J* = 14.7 Hz, 1H), 3.81 (s, 3H), 1.25 (s, 12H).

All recorded spectroscopic data matched those previously reported in the literature[13].

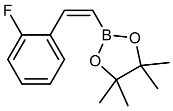

19b

(Z)-2-(2-fluorostyryl)-4,4,5,5-tetramethyl-1,3,2-dioxaborolane (**19b**): The title compound was prepared following general procedure A (1 mol% of [Co]). **19a** (48.1 mg, 0.4 mmol) was added into a vail with $Co(acac)_2$ (1.0 mg, 0.004 mmol), CNC-^{i}Pr (2.6 mg, 0.0056 mmol), ^{t}BuOK (2.5 mg, 0.0224 mmol), HBpin (75 μL, 0.52 mmol, 1.3 equiv.) and DMF (0.5 mL). The reaction mixture was stirred for 12 hours. DBM was added as internal standard and NMR yield (96%) was obtained. Purification by flash column chromatography on silica gel (eluent: petroleumether→ petroleumether/EtOAc =20/1) afforded the product as a pale yellow oil in 82% yield (93.6 mg, Z:E=97:3)). See spectrum

^{1}H NMR (400 MHz, Chloroform-*d*) δ 7.61 (td, *J* = 7.7, 1.7 Hz, 1H), 7.32 (d, *J* = 14.8 Hz, 1H), 7.27-7.21 (m, 1H), 7.09 – 7.04 (m, 1H), 7.03-6.97 (m, 1H), 5.74 (d, *J* = 14.9 Hz, 1H), 1.27 (s, 12H)

^{13}C NMR (151 MHz, $CDCl_3$) δ 161.2, 159.6, 140.3, 130.2, 130.2, 129.6, 129.6, 126.5, 126.4, 123.3, 123.3, 115.2, 115.0, 83.5, 24.8.

^{11}B NMR (193 MHz, $CDCl_3$) δ 30.0.

^{19}F NMR (565 MHz, $CDCl_3$) δ -116.4.

HRMS (APCI+) *m/z* calculated for C14H19BFO2+ ([M+H]+) 249.1457; found 249.1454.

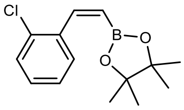
20b

(Z)-2-(2-chlorostyryl)-4,4,5,5-tetramethyl-1,3,2-dioxaborolane (**20b**): The title compound was prepared following general procedure A (1 mol% of [Co], 2 eq. HBPin, 0.5 mmol scale). **20a** (68.3 mg, 0.5 mmol) was added into a vail with Co(acac)$_2$ (1.3 mg, 0.005 mmol), CNC-^{i}Pr (3.3 mg, 0.007 mmol), ^{t}BuOK (3.1 mg, 0.028 mmol), HBpin (144 µL, 1 mmol, 2 equiv.) and DMF (0.5 mL). The reaction mixture was stirred for 12 hours. DBM was added as internal standard and NMR yield (97%) was obtained. Purification by flash column chromatography on silica gel (eluent: petroleumether→ petroleumether/EtOAc =20/1) afforded the product as a pale yellow oil in 87% yield (46.0 mg, Z:E>99:1). See spectrum

^{1}H NMR (400 MHz, Chloroform-*d*) δ 7.55 (d, *J* = 7.3 Hz, 1H), 7.44 (d, *J* = 14.6 Hz, 1H), 7.33 (d, *J* = 7.6 Hz, 1H), 7.19 (p, *J* = 7.3 Hz, 2H), 5.74 (d, *J* = 14.6 Hz, 1H), 1.24 (s, 12H).

^{13}C NMR (101 MHz, $CDCl_3$) δ 145.1, 136.9, 133.6, 130.3, 129.2, 129.1, 126.0, 83.5, 24.8.

^{11}B NMR (128 MHz, $CDCl_3$) δ 30.2.

HRMS (APCI+) *m*/*z* calculated for C14H19BClO2+ ([M+H]+) 265.1161; found 265.1159.

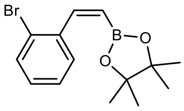
21b

(Z)-2-(2-bromostyryl)-4,4,5,5-tetramethyl-1,3,2-dioxaborolane (**21b**): The title compound was prepared following general procedure A (1 mol% of [Co], 2.5 eq. HBPin, 0.2 mmol scale). **21a** (36.2 mg, 0.2 mmol) was added into a vail with Co(acac)$_2$ (0.5 mg, 0.002 mmol), CNC-^{i}Pr (1.3 mg, 0.0028 mmol), ^{t}BuOK (1.3 mg, 0.0112 mmol), HBpin (72 µL, 0.5 mmol, 2.5 equiv.) and DMF (0.5 mL). The reaction mixture was stirred for 12 hours. DBM was added as internal standard and NMR yield (77%) was obtained. Purification by flash column chromatography on silica gel (eluent: petroleumether→ petroleumether/EtOAc =20/1) afforded the product as a pale yellow oil in 74% yield (46.2 mg, Z:E=97:3). See spectrum

The title compound can also be synthesized according to general procedure A (0.5 mol% of [Co]), resulting in a 29% NMR yield (Z:E=27:2) when dibromomethane was used as the internal standard. When conditions were modified to 1.0 mol% of [Co], 2 eq. HBPin, an NMR yield (60%) was obtained. Purification by flash column chromatography on silica gel (eluent: petroleumether→ petroleumether/EtOAc =20/1) afforded the product as a pale yellow oil in 45% yield (56.2 mg, Z:E=95:5).

^{1}H NMR (400 MHz, Chloroform-*d*) δ 7.53 (d, *J* = 7.9 Hz, 2H), 7.37 (d, *J* = 14.6 Hz, 1H), 7.22 (t, *J* = 7.5 Hz, 1H), 7.13 (td, *J* = 7.8, 1.5 Hz, 1H), 5.71 (d, *J* = 14.6 Hz, 1H), 1.23 (s, 12H).

^{13}C NMR (151 MHz, $CDCl_3$) δ 147.4, 138.6, 132.3, 130.5, 129.3, 126.6, 123.8, 83.5, 24.8.

^{11}B NMR (193 MHz, $CDCl_3$) δ 30.1.

HRMS (APCI+) *m*/*z* calculated for C14H19BBr1O2+ ([M+H]+) 309.0656; found 309.0649.

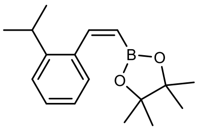
22b

(Z)-2-(2-isopropylstyryl)-4,4,5,5-tetramethyl-1,3,2-dioxaborolane (**22b**): The title compound was prepared following general procedure A (0.5 mol% of [Co]). **22a** (57.7 mg, 0.4 mmol) was added into a vail with Co(acac)$_2$ (0.5 mg, 0.002 mmol), CNC-^{i}Pr (1.3 mg, 0.0028 mmol), ^{t}BuOK (1.3 mg, 0.0112 mmol), HBpin (75µL, 0.52 mmol, 1.3 equiv.) and DMF (0.5 mL). The reaction mixture was stirred for 30 minutes. DBM was added as internal standard and NMR yield (83%) was obtained. Purification by flash column chromatography on silica gel (eluent: petroleumether→ petroleumether/EtOAc =20/1) afforded the product as a pale yellow oil in 78% yield (84.9 mg, Z:E>99:1)). See spectrum

^{1}H NMR (400 MHz, Chloroform-*d*) δ 7.49 (d, *J* = 14.4 Hz, 1H), 7.30 (d, *J* = 7.9 Hz, 1H), 7.26 – 7.21 (m, 2H), 7.11-7.04 (m, 1H), 5.66 (d, *J* = 14.4 Hz, 1H), 3.15 (hept, *J* = 6.9 Hz, 1H), 1.21 (d, *J* = 6.9 Hz, 6H), 1.17 (s, 12H).

^{13}C NMR (101 MHz, CDCl$_3$) δ 147.4, 146.2, 137.4, 129.3, 128.1, 124.9, 124.2, 83.2, 29.7, 24.7, 23.3.

^{11}B NMR (128 MHz, CDCl$_3$) δ 30.6.

HRMS (ESI+) *m/z* calculated for C17H26BO2+ ([M+H]+) 273.2020; found 273.2017.

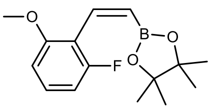

23b

(Z)-2-(2-fluoro-6-methoxystyryl)-4,4,5,5-tetramethyl-1,3,2-dioxaborolane (**23b**): The title compound was prepared following general procedure A (0.5 mol% of [Co]). **23a** (60.1 mg, 0.4 mmol) was added into a vail with Co(acac)$_2$ (0.5 mg, 0.002 mmol), CNC-^{i}Pr (1.3 mg, 0.0028 mmol), ^{t}BuOK (1.3 mg, 0.0112 mmol), HBpin (75µL, 0.52 mmol, 1.3 equiv.) and DMF (0.5 mL). The reaction mixture was stirred for 12 hours. DBM was added as internal standard and NMR yield (99%) was obtained. Purification by flash column chromatography on silica gel (eluent: petroleumether→ petroleumether/EtOAc =20/1) afforded the product as a white solid in 88% yield (98.2 mg, Z:E=99:1). See spectrum

^{1}H NMR (600 MHz, Chloroform-*d*) δ 7.16 (q, *J* = 8.0 Hz, 1H), 7.04 (d, *J* = 14.8 Hz, 1H), 6.68 – 6.61 (m, 2H), 5.88 (d, *J* = 14.8 Hz, 1H), 3.81 (s, 3H), 1.21 (s, 12H).

^{13}C NMR (151 MHz, CDCl$_3$) δ 161.1, 159.5, 158.2, 136.3, 128.7, 128.6, 116.6, 116.5, 107.9, 107.8, 106.0, 106.0, 83.1, 56.0, 24.8.

^{11}B NMR (193 MHz, CDCl$_3$) δ 29.6.

^{19}F NMR (565 MHz, CDCl$_3$) δ -113.4.

HRMS (ESI+) *m/z* calculated for C15H21BFO3+ ([M+H]+) 279.1562; found 279.1565.

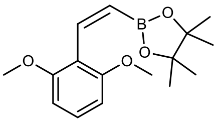

24b

(Z)-2-(2,6-dimethoxystyryl)-4,4,5,5-tetramethyl-1,3,2-dioxaborolane (**24b**): The title compound was prepared following general procedure A (0.5 mol% of [Co], 0.2 mmol scale, 3 eq. HBpin). **24a** (32.4 mg, 0.2 mmol) was added into a vail with Co(acac)$_2$ (0.25 mg, 0.001 mmol), CNC-^{i}Pr (0.65 mg, 0.0014 mmol), ^{t}BuOK (0.6 mg, 0.0056 mmol), HBpin (87 µL, 0.6 mmol, 3 equiv.) and DMF (0.5 mL). The reaction mixture was stirred for 1 hours. DBM was added as internal standard and

NMR yield (>99%) was obtained. Purification by flash column chromatography on silica gel (eluent: petroleumether→ petroleumether/EtOAc =20/1) afforded the product as a white solid in 90% yield (52.3 mg, Z:E>99:1). See spectrum

^{1}H NMR (400 MHz, Chloroform-*d*) δ 7.16 (t, *J* = 8.3 Hz, 1H), 7.06 (d, *J* = 14.8 Hz, 1H), 6.53 (d, *J* = 8.3 Hz, 2H), 5.84 (d, *J* = 14.8 Hz, 1H), 3.77 (s, 6H), 1.18 (s, 12H).

^{13}C NMR (101 MHz, $CDCl_3$) δ 157.7, 138.2, 128.4, 117.7, 104.2, 82.6, 56.0, 24.9.

^{11}B NMR (128 MHz, $CDCl_3$) δ 29.0.

HRMS (ESI+) *m/z* calculated for C16H24BO1+ ([M+H]+) 291.1762; found 291.1759.

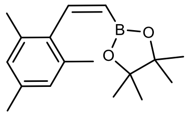

25b

(Z)-4,4,5,5-tetramethyl-2-(2,4,6-trimethylstyryl)-1,3,2-dioxaborolane (**25b**): The title compound was prepared following general procedure A (0.5 mol% of [Co]). **25a** (57.7 mg, 0.4 mmol) was added into a vail with $Co(acac)_2$ (0.5 mg, 0.002 mmol), CNC-^{i}Pr (1.3 mg, 0.0028 mmol), ^{t}BuOK (1.3 mg, 0.0112 mmol), HBpin (75µL, 0.52 mmol, 1.3 equiv.) and DMF (0.5 mL). The reaction mixture was stirred for 12 hours. DBM was added as internal standard and NMR yield (98%) was obtained. Purification by flash column chromatography on silica gel (eluent: petroleumether→ petroleumether/EtOAc =20/1) afforded the product as a white solid in 90% yield (98.3 mg, Z:E=98:2). See spectrum

^{1}H NMR (600 MHz, Chloroform-*d*) δ 7.17 (d, *J* = 14.5 Hz, 1H), 6.81 (s, 2H), 5.84 (d, *J* = 14.5 Hz, 1H), 2.28 (s, 3H), 2.20 (s, 6H), 1.08 (s, 12H).

^{13}C NMR (151 MHz, $CDCl_3$) δ 148.4, 136.4, 135.7, 135.1, 127.4, 82.8, 24.6, 21.0, 20.5.

^{11}B NMR (128 MHz, $CDCl_3$) δ 28.8.

HRMS (ESI+) *m/z* calculated for C17H26B1O2+ ([M+H]+) 273.2020; found 273.2018.

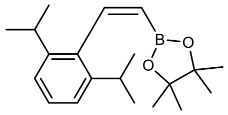

26b

(Z)-2-(2,6-diisopropylstyryl)-4,4,5,5-tetramethyl-1,3,2-dioxaborolane (**26b**): The title compound was prepared following general procedure A (0.5 mol% of [Co]). **26a** (74.5 mg, 0.4 mmol) was added into a vail with $Co(acac)_2$ (0.5 mg, 0.002 mmol), CNC-^{i}Pr (1.3 mg, 0.0028 mmol), ^{t}BuOK (1.3 mg, 0.0112 mmol), HBpin (75µL, 0.52 mmol, 1.3 equiv.) and DMF (0.5 mL). The reaction mixture was stirred for 12 hours. DBM was added as internal standard and NMR yield (92%) was obtained. Purification by flash column chromatography on silica gel (eluent: petroleumether→ petroleumether/EtOAc =20/1) afforded the product as a pale yellow oil in 88% yield (111.2 mg, Z:E>99:1). See spectrum

^{1}H NMR (400 MHz, Chloroform-*d*) δ 7.36 (d, *J* = 14.5 Hz, 1H), 7.25 – 7.18 (m, 1H), 7.09 (d, *J* = 7.7 Hz, 2H), 5.91 (d, *J* = 14.5 Hz, 1H), 3.19 (hept, *J* = 6.9 Hz, 2H), 1.17 (d, *J* = 6.5 Hz, 12H), 1.05 (s, 12H).

^{13}C NMR (101 MHz, $CDCl_3$) δ 148.6, 145.6, 137.1, 126.9, 121.6, 82.8, 30.0, 24.6, 23.4.

^{11}B NMR (128 MHz, $CDCl_3$) δ 29.6.

HRMS (ESI+) *m/z* calculated for C20H32BO2+ ([M+H]+) 315.2490; found 315.2487.

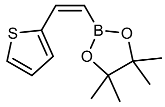

27b

(Z)-4,4,5,5-tetramethyl-2-(2-(thiophen-2-yl)vinyl)-1,3,2-dioxaborolane (**27b**): The title compound was prepared following general procedure A (0.5 mol% of [Co], 2 eq. HBPin). DBM was added as internal standard and NMR yield (89%) was obtained. Purification by flash column chromatography on silica gel (eluent: petroleumether→ petroleumether/EtOAc =20/1) afforded the product as a pale yellow oil in 81% yield (76.4 mg, Z:E=99:1). See spectrum

^{1}H NMR (400 MHz, Chloroform-*d*) δ 7.40 (d, *J* = 3.6 Hz, 1H), 7.28 (d, *J* = 5.0 Hz, 1H), 7.23 (d, *J* = 15.0 Hz, 1H), 6.99 (dd, *J* = 5.1, 3.6 Hz, 1H), 5.44 (d, *J* = 15.1 Hz, 1H), 1.34 (s, 12H).

All recorded spectroscopic data matched those previously reported in the literature[15].

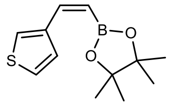

28b

(Z)-4,4,5,5-tetramethyl-2-(2-(thiophen-3-yl)vinyl)-1,3,2-dioxaborolane (**28b**): The title compound was prepared following general procedure A (0.5 mol% of [Co]). DBM was added as internal standard and NMR yield (99%) was obtained. Purification by flash column chromatography on silica gel (eluent: petroleumether→ petroleumether/EtOAc =20/1) afforded the product as a pale yellow oil in 93% yield (92.25 mg, Z:E>99:1). See spectrum

^{1}H NMR (400 MHz, Chloroform-*d*) δ 7.62 (dd, *J* = 38.7, 3.8 Hz, 2H), 7.23 (dd, *J* = 5.0, 3.0 Hz, 1H), 7.17 (d, *J* = 15.1 Hz, 1H), 5.48 (d, *J* = 15.1 Hz, 1H), 1.32 (s, 12H).

^{13}C NMR (151 MHz, $CDCl_3$) δ 141.9, 140.7, 128.6, 126.2, 124.9, 83.5, 24.9.

^{11}B NMR (193 MHz, $CDCl_3$) δ 30.0.

All recorded spectroscopic data matched those previously reported in the literature[13].

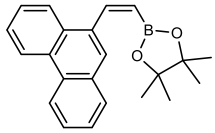

29b

(Z)-4,4,5,5-tetramethyl-2-(2-(phenanthren-9-yl)vinyl)-1,3,2-dioxaborolane (**29b**): The title compound was prepared following general procedure A (1 mol% of [Co], 2 eq. HBpin). **29a** (80.9 mg, 0.4 mmol) was added into a vail with Co(acac)$_2$ (1.0 mg, 0.004 mmol), CNC-^{i}Pr (2.6 mg, 0.0056 mmol), ^{t}BuOK (2.5 mg, 0.0224 mmol), HBpin (115 μL, 0.8 mmol, 2 equiv.) and DMF (0.5 mL). The reaction mixture was stirred for 12 hours. DBM was added as internal standard and NMR yield (83%) was obtained. Purification by flash column chromatography on silica gel (eluent: petroleumether→ petroleumether/EtOAc =20/1) afforded the product as a white solid in 82% yield (107.8 mg, Z:E=98:2). See spectrum

The title compound can also be synthesized according to general procedure A (0.5 mol% of [Co]), resulting in a 51% NMR yield (Z:E>99:1) when dibromomethane was used as the internal standard. When 1.0 mol% of [Co] was used, an NMR yield of 60% with a Z:E> 99:1 was obtained.

^{1}H NMR (600 MHz, Chloroform-*d*) δ 8.73 (d, *J* = 8.1 Hz, 1H), 8.68 (d, *J* = 8.1 Hz, 1H), 8.11 (d, *J* = 8.0 Hz, 1H), 7.91 (d, *J* = 14.2 Hz, 1H), 7.87 (d, *J* = 7.7 Hz, 1H), 7.84 (s, 1H), 7.69 – 7.60 (m, 4H), 5.98 (d, *J* = 14.2 Hz, 1H), 1.18 (s, 12H).

^{13}C NMR (151 MHz, $CDCl_3$) δ 146.5, 135.0, 131.5, 130.9, 130.5, 130.2, 128.6, 127.1, 126.6, 126.6, 126.5, 126.3, 125.4, 122.9, 122.6, 83.3, 24.7.
^{11}B NMR (193 MHz, $CDCl_3$) δ 30.6.
HRMS (ESI+) *m/z* calculated for C22H24BO2+ ([M+H]+) 338.1864; found 331.1862.

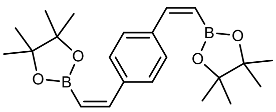
30b

1,4-bis((Z)-2-(4,4,5,5-tetramethyl-1,3,2-dioxaborolan-2-yl)vinyl)benzene (**30b**): The title compound was prepared following general procedure A (0.5 mol% of [Co], 0.2 mmol scale, 2 eq. HBpin) , the reaction mixture was stirred for 1 hours. DBM was added as internal standard and NMR yield (85%) was obtained. Purification by flash column chromatography on silica (eluent: petroleumether→ petroleumether/EtOAc =20/1) afforded the product as a white solid in 82% yield (62.2 mg, Z:E=95:5). See spectrum
^{1}H NMR (400 MHz, Chloroform-*d*) δ 7.51 (s, 4H), 7.18 (d, J = 14.9 Hz, 2H), 5.58 (d, J = 14.9 Hz, 2H), 1.30 (s, 24H).
All recorded spectroscopic data matched those previously reported in the literature[16].

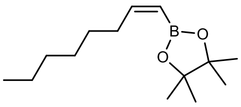
31b

(Z)-4,4,5,5-tetramethyl-2-(oct-1-en-1-yl)-1,3,2-dioxaborolane (**31b**): The title compound was prepared following general procedure A (1 mol% of [Co]), the reaction mixture was stirred for 24 hours. DBM was added as internal standard and NMR yield (76%) was obtained. Purification by flash column chromatography on silica gel (eluent: petroleumether→ petroleumether/EtOAc =20/1) afforded the product as a colorless oil in 74% yield (68.2 mg, Z:E=98:2). See spectrum
^{1}H NMR (400 MHz, Chloroform-*d*) δ 6.43 (dt, J = 14.3, 7.5 Hz, 1H), 5.32 (dt, J = 13.5, 1.3 Hz, 1H), 2.38 (m, 2H), 1.41 – 1.27 (m, 8H), 1.26 (s, 12H), 0.88 (t, J = 6.8, 3H).
^{11}B NMR (193 MHz, $CDCl_3$) δ 29.8.
All recorded spectroscopic data matched those previously reported in the literature[13].

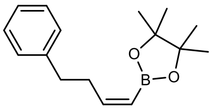
32b

(Z)-4,4,5,5-tetramethyl-2-(4-phenylbut-1-en-1-yl)-1,3,2-dioxaborolane (**32b**): The title compound was prepared following general procedure A (1 mol% of [Co]). DBM was added as internal standard and NMR yield (72%) was obtained. Purification by flash column chromatography on silica gel (eluent: petroleumether→ petroleumether/EtOAc =20/1) afforded the product as a colorless oil in 60% yield (61.8 mg, Z:E=98:2). See spectrum
^{1}H NMR (400 MHz, Chloroform-*d*) δ 7.34 – 7.16 (m, 5H), 6.49 (dt, J = 13.3, 6.0 Hz, 1H), 5.39 (d, J = 13.5 Hz, 1H), 2.81 – 2.68 (m, 4H), 1.27 (s, 12H).
All recorded spectroscopic data matched those previously reported in the literature[17].

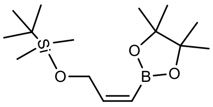

33b

(Z)-tert-butyldimethyl((3-(4,4,5,5-tetramethyl-1,3,2-dioxaborolan-2-yl)allyl)oxy)silane (**33b**): The title compound was prepared following general procedure A (1 mol% of [Co], 2 eq. HBpin). DBM was added as internal standard and NMR yield (52%) was obtained. Purification by flash column chromatography on silica gel (eluent: petroleumether→ petroleumether/EtOAc =20/1) afforded the product as a colorless oil in 48% yield (56.7 mg, Z:E=97:3). See spectrum

^{1}**H NMR** (400 MHz, Chloroform-*d*) δ 6.50 (dt, *J* = 12.9, 5.9 Hz, 1H), 5.38 (d, *J* = 13.8 Hz, 1H), 4.49 (d, *J* = 6.1 Hz, 2H), 1.25 (s, 12H), 0.90 (s, 9H), 0.07 (s, 6H).

All recorded spectroscopic data matched those previously reported in the literature[18].

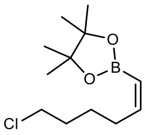

34b

(Z)-2-(6-chlorohex-1-en-1-yl)-4,4,5,5-tetramethyl-1,3,2-dioxaborolane (**34b**): The title compound was prepared following general procedure A (1 mol% of [Co]). **34a** (46.6 mg, 0.4 mmol) was added into a vail with Co(acac)$_2$ (1.0 mg, 0.004 mmol), CNC-^{i}Pr (2.6 mg, 0.0056 mmol), ^{t}BuOK (2.5 mg, 0.0224 mmol), HBpin (75 µL, 0.52 mmol, 1.3 equiv.) and DMF (0.5 mL). The reaction mixture was stirred for 12 hours. DBM was added as internal standard and NMR yield (71%) was obtained. Purification by flash column chromatography on silica gel (eluent: petroleumether→ petroleumether/EtOAc =20/1) afforded the product as a colorless oil in 63% yield (61.6 mg, Z:E=95:5). See spectrum

^{1}**H NMR** (400 MHz, Chloroform-*d*) δ 6.37 (dt, *J* = 14.3, 7.5 Hz, 1H), 5.34 (dt, *J* = 13.5, 1.2 Hz, 1H), 3.53 (t, *J* = 6.8 Hz, 2H), 2.40 (m, 2H), 1.76 (p, *J* = 6.9 Hz, 2H), 1.51 (p, *J* = 7.3 Hz, 2H), 1.24 (s, 12H).

^{13}**C NMR** (101 MHz, $CDCl_3$) δ 154.1, 82.9, 44.9, 31.8, 31.1, 26.5, 24.9.

^{11}**B NMR** (128 MHz, $CDCl_3$) δ 29.8.

HRMS (APCI+) *m/z* calculated for C12H23BClO2+ ([M+H]+) 245.1474; found 245.1470.

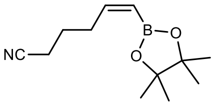

35b

(Z)-6-(4,4,5,5-tetramethyl-1,3,2-dioxaborolan-2-yl)hex-5-enenitrile (**35b**): The title compound was prepared following general procedure A (1 mol% of [Co]). **35a** (37.3 mg, 0.4 mmol) was added into a vail with Co(acac)$_2$ (1.0 mg, 0.004 mmol), CNC-^{i}Pr (2.6 mg, 0.0056 mmol), ^{t}BuOK (2.5 mg, 0.0224 mmol), HBpin (75 µL, 0.52 mmol, 1.3 equiv.) and DMF (0.5 mL). The reaction mixture was stirred for 12 hours. DBM was added as internal standard and NMR yield (99%) was obtained. Purification by flash column chromatography on silica gel (eluent: petroleumether→ petroleumether/EtOAc =10/1) afforded the product as a colorless oil in 99% yield (85.3 mg, Z:E>99:1). See spectrum

^{1}**H NMR** (400 MHz, Chloroform-*d*) δ 6.35 (dt, *J* = 14.1, 7.4 Hz, 1H), 5.45 (d, *J* = 13.4 Hz, 1H), 2.53 (m, 2H), 2.33 (t, *J* = 7.3 Hz, 2H), 1.77 (p, *J* = 7.2 Hz, 2H), 1.27 (s, 12H).

^{13}C NMR (101 MHz, $CDCl_3$) δ 151.5, 119.9, 83.2, 31.0, 25.2, 24.9, 16.4.
^{11}B NMR (193 MHz, $CDCl_3$) δ 29.5.
HRMS (ESI+) *m/z* calculated for C12H20BNO2Na+ ([M+Na]+) 244.1479; found 244.1476.

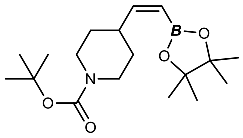

36b

tert-butyl (Z)-4-(2-(4,4,5,5-tetramethyl-1,3,2-dioxaborolan-2-yl)vinyl)piperidine-1-carboxylate (**36b**): The title compound was prepared following general procedure A (1 mol% of [Co]). **36a** (83.7 mg, 0.4 mmol) was added into a vail with Co(acac)$_2$ (1.0 mg, 0.004 mmol), CNC-^{i}Pr (2.6 mg, 0.0056 mmol), ^{t}BuOK (2.5 mg, 0.0224 mmol), HBpin (75 µL, 0.52 mmol, 1.3 equiv.) and DMF (0.5 mL). The reaction mixture was stirred for 12 hours. DBM was added as internal standard and NMR yield (99%) was obtained. Purification by flash column chromatography on silica gel (eluent: petroleumether→ petroleumether/EtOAc =20/1) afforded the product as a pale yellow oil in 95% yield (128.3 mg, Z:E>99:1). See spectrum

^{1}H NMR (600 MHz, Chloroform-*d*) δ 6.22 (dd, *J* = 13.4, 9.2 Hz, 1H), 5.31 (d, *J* = 13.5 Hz, 1H), 4.21 – 3.92 (m, 2H), 2.93 – 2.82 (m, 1H), 2.75 (m, 2H), 1.60 (d, *J* = 12.7 Hz, 2H), 1.45 (s, 9H), 1.26 (s, 14H).
^{13}C NMR (151 MHz, $CDCl_3$) δ 158.3, 155.1, 83.1, 79.3, 43.7, 38.9, 32.2, 28.6, 25.0.
^{11}B NMR (193 MHz, $CDCl_3$) δ 29.8.
HRMS (APCI+) *m/z* calculated for C18H32BNO4Na+ ([M+H]+) 360.2316; found 360.2315

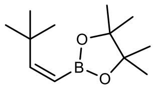

37b

(Z)-2-(3,3-dimethylbut-1-en-1-yl)-4,4,5,5-tetramethyl-1,3,2-dioxaborolane (**37b**): The title compound was prepared following general procedure A (1 mol% of [Co]). DBM was added as internal standard and NMR yield (46%) was obtained. Isolated yield was unobtainable due to its low boiling point (Z:E=97:3). See spectrum

^{1}H NMR (400 MHz, Chloroform-*d*) δ 6.24 (d, *J* = 15.0 Hz, 1H), 5.16 (d, *J* = 15.0 Hz, 1H), 1.25 (s, 12H), 1.08 (s, 9H).
All recorded spectroscopic data matched those previously reported in the literature[18].

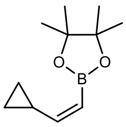

38b

(Z)-2-(2-cyclopropylvinyl)-4,4,5,5-tetramethyl-1,3,2-dioxaborolane (**38b**): The title compound was prepared following general procedure A (1 mol% of [Co]). DBM was added as internal standard and NMR yield (87%) was obtained. Purification by flash column chromatography on silica gel (eluent: petroleumether→ petroleumether/EtOAc =20/1) afforded the product as a pale yellow oil in 81% yield (78.3 mg, Z:E=98:2). See spectrum

^{1}**H NMR** (400 MHz, Chloroform-*d*) δ 5.73 – 5.58 (m, 1H), 5.18 (d, *J* = 13.5 Hz, 1H), 2.35 – 2.23 (m, 1H), 1.27 (s, 12H), 0.87 – 0.78 (m, 2H), 0.45 – 0.36 (m, 2H).
All recorded spectroscopic data matched those previously reported in the literature[19].

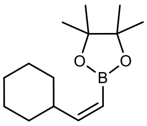

39b

(Z)-2-(2-cyclohexylvinyl)-4,4,5,5-tetramethyl-1,3,2-dioxaborolane (**39b**): The title compound was prepared following general procedure A (1 mol% of [Co]). DBM was added as internal standard and NMR yield (89%) was obtained. Purification by flash column chromatography on silica gel (eluent: petroleumether→ petroleumether/EtOAc =20/1) afforded the product as a pale yellow oil in 75% yield (66.3 mg, Z:E=97:3). See spectrum
^{1}**H NMR** (400 MHz, Chloroform-*d*) δ 6.25 (dd, *J* = 13.3, 9.4 Hz, 1H), 5.22 (dd, *J* = 13.5, 0.8 Hz, 1H), 2.78 – 2.62 (m, 1H), 1.72 – 1.62 (m, 4H), 1.36-1.27 (m, 2H), 1.26 (s, 12H), 1.23-1.01 (m, 4H).
^{1}H NMR spectra are consistent with previously reported data[17].

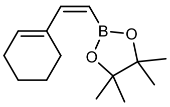

40b

(Z)-2-(2-(cyclohex-1-en-1-yl)vinyl)-4,4,5,5-tetramethyl-1,3,2-dioxaborolane (**40b**): The title compound was prepared following general procedure A (1 mol% of [Co], 2 eq. HBpin). DBM was added as internal standard and NMR yield (99%) was obtained. Purification by flash column chromatography on silica gel (eluent: petroleumether→ petroleumether/EtOAc =20/1) afforded the product as a colorless oil in 91% yield (86.7 mg, Z:E=99:1). See spectrum
^{1}**H NMR** (400 MHz, Chloroform-*d*) δ 6.64 (d, *J* = 14.9 Hz, 1H), 5.83 (s, 1H), 5.17 (d, *J* = 14.9 Hz, 1H), 2.29 – 2.21 (m, 2H), 2.17 – 2.08 (m, 2H), 1.67 – 1.55 (m, 4H), 1.28 (s, 12H).
^{13}**C NMR** (101 MHz, $CDCl_3$) δ 149.8, 138.0, 132.0, 83.3, 26.3, 26.0, 24.8, 22.5, 22.1.
^{11}**B NMR** (128 MHz, $CDCl_3$) δ 30.1.
All recorded spectroscopic data matched those previously reported in the literature[20].

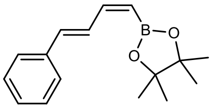

41b

4,4,5,5-tetramethyl-2-((1Z,3E)-4-phenylbuta-1,3-dien-1-yl)-1,3,2-dioxaborolane (**41b**): The title compound was prepared following general procedure A (1 mol% of [Co]) , the reaction mixture was stirred for 40 mins. DBM was added as internal standard and NMR yield (86%) was obtained. Purification by flash column chromatography on silica gel (eluent: petroleumether→ petroleumether/EtOAc =20/1) afforded the product as a colorless oil in 80% yield (82.2 mg, Z:E=99:1). See spectrum
^{1}**H NMR** (600 MHz, Chloroform-*d*) δ 7.67 (dd, *J* = 15.3, 11.4 Hz, 1H), 7.46 (d, *J* = 7.4 Hz, 2H), 7.35 (t, *J* = 7.3 Hz, 2H), 7.26 (t, *J* = 7.1 Hz, 1H), 7.03 (t, *J* = 12.1 Hz, 1H), 6.66 (d, *J* = 15.7 Hz, 1H), 5.48 (d, *J* = 13.2 Hz, 1H), 1.33 (s, 12H).

^{13}**C NMR** (151 MHz, $CDCl_3$) δ 150.4, 137.2, 136.4, 129.4, 128.6, 127.9, 126.9, 83.1, 24.9.
^{11}**B NMR** (193 MHz, $CDCl_3$) δ 29.6.
All recorded spectroscopic data matched those previously reported in the literature[20].

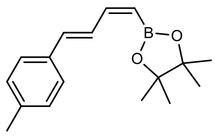

42b

4,4,5,5-tetramethyl-2-((1Z,3E)-4-(p-tolyl)buta-1,3-dien-1-yl)-1,3,2-dioxaborolane (**42b**): The title compound was prepared following general procedure A (1 mol% of [Co]), the reaction mixture was stirred for 10 mins. DBM was added as internal standard and NMR yield (99%) was obtained. Purification by flash column chromatography on silica gel (eluent: petroleumether→ petroleumether/EtOAc =20/1) afforded the product as a colorless oil in 92% yield (99.8 mg, Z:E>99:1). See spectrum
^{1}**H NMR** (600 MHz, Chloroform-*d*) δ 7.63 (dd, *J* = 15.6, 11.1 Hz, 1H), 7.35 (d, *J* = 7.9 Hz, 2H), 7.15 (d, *J* = 7.8 Hz, 2H), 7.02 (t, *J* = 12.1 Hz, 1H), 6.63 (d, *J* = 15.6 Hz, 1H), 5.45 (d, *J* = 13.3 Hz, 1H), 2.36 (s, 3H), 1.33 (s, 12H).
^{13}**C NMR** (151 MHz, $CDCl_3$) δ 150.6, 137.9, 136.4, 134.5, 129.3, 128.6, 126.8, 83.1, 24.9, 21.3.
^{11}**B NMR** (193 MHz, $CDCl_3$) δ 29.9.
All recorded spectroscopic data matched those previously reported in the literature[20].

4. Time-dependent Z/E selective transformation

General procedure B: In a nitrogen atmosphere, a vail was charged with $Co(acac)_2$ (4.1 mg, 0.016 mmol), CNC-^{i}Pr (10.2 mg, 0.022 mmol), ^{t}BuOK (10.1 mg, 0.09 mmol) dissolved in dry DMF (2 mL) and was stirred for 5 mins. The freshly prepared stock solution of the *in situ* prepared active catalyst ([Co]=8.0 mM in DMF) was added via a micro-syringe (50-500 µL, 0.0004-0.004 mmol, 0.1-1 mol%) to a vial charged with HBpin (174µL, 1.2 mmol) and DMF([alkyne]=0.8 M). The mixture was stirred for 5 mins. Alkyne (0.4 mmol) was added rapidly and the resulting mixture was stirred for 5 s -24 h. The reaction was then quenched by adding water. 20 mL EtOAc was added and the organic phase was washed with 10 mL brine twice to remove most of DMF. Pure product was isolated by column chromatography over silica gel deactivated with 2% NEt_3 in petroleum ether using petroleumether/EtOAc as the eluent.
General procedure C: In a nitrogen atmosphere, a vail was charged with $Co(acac)_2$ (5.1 mg, 0.02 mmol, 5 mol%), CNC-^{i}Pr (12.8 mg, 0.028 mmol), ^{t}BuOK (12.6 mg, 0.112 mmol) dissolved in dry DMF (1 mL). The mixture was stirred for 5 mins followed by adding HBpin (174µL, 1.2 mmol). the mixture was stirred for further 5 mins and alkynes (0.4 mmol) was added rapidly. the resulting mixture was stirred for 5 s -24 h. The reaction was then quenched by adding water. 20 mL EtOAc was added and the organic phase was washed with 10 mL brine twice to remove most of DMF. Pure product was isolated by column chromatography over silica gel deactivated with 2% NEt_3 in petroleum ether using petroleumether/EtOAc as the eluent.

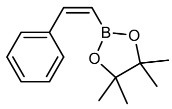

Z-**1b**

(Z)-4,4,5,5-tetramethyl-2-styryl-1,3,2-dioxaborolane (Z-**1b**): The title compound was prepared following general procedure B (0.5 mol% of [Co]), the reaction mixture was stirred for 5 min before quenched. Purification by flash column chromatography on silica gel (eluent: petroleumether→ petroleumether/EtOAc =20/1) afforded the product as a pale yellow oil in 92% yield (80.7 mg, Z:E>99:1).

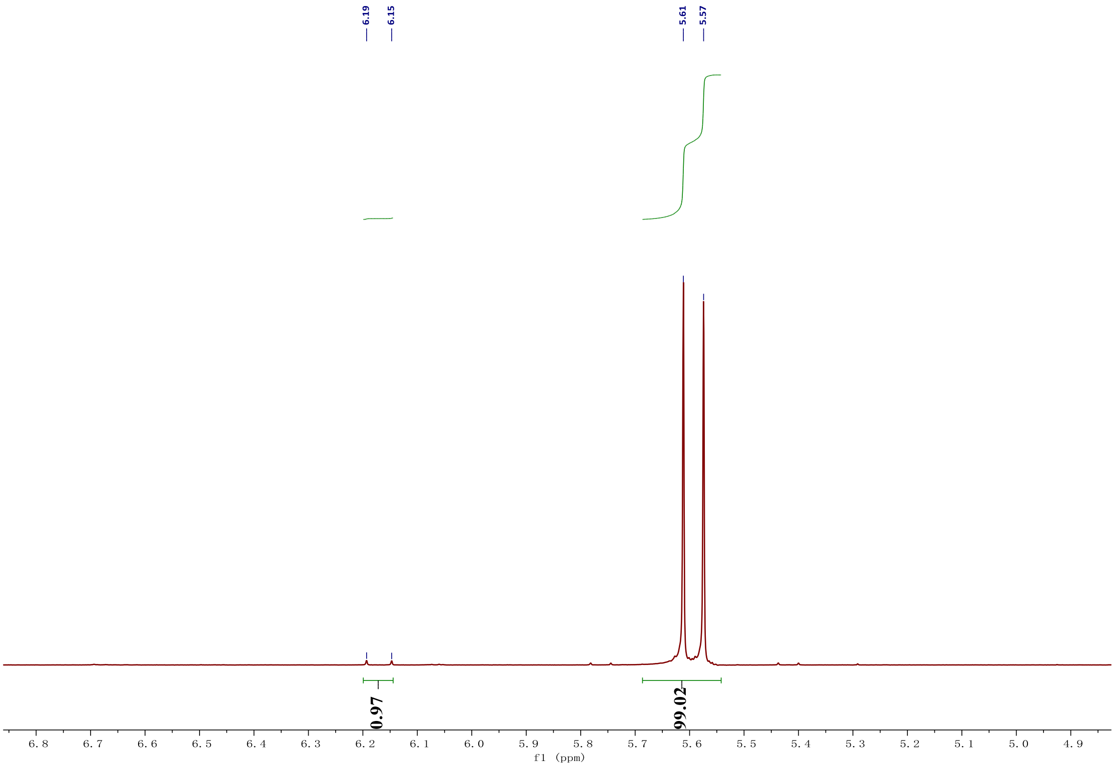

Z/E ratio of Z-**1b**

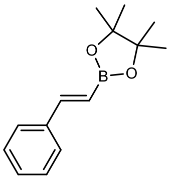

E-**1b**

(E)-4,4,5,5-tetramethyl-2-styryl-1,3,2-dioxaborolane (E-**1b**): The title compound was prepared following general procedure B (0.5 mol% of [Co]), the reaction mixture was stirred for 24 h before quenched. Purification by flash column chromatography on silica gel (eluent: petroleumether→ petroleumether/EtOAc =20/1) afforded the product as a pale yellow oil in 97% yield (89.3 mg, E:Z>99:1). See spectrum

^{1}H NMR (400 MHz, Chloroform-*d*) δ 7.49 (d, *J* = 6.9 Hz, 2H), 7.40 (d, *J* = 18.5 Hz, 1H), 7.36 – 7.26 (m, 3H), 6.17 (d, *J* = 18.4 Hz, 1H), 1.32 (s, 12H).

^{1}H NMR spectra are consistent with previously reported data[21].

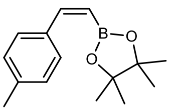

Z-**2b**

(Z)-4,4,5,5-tetramethyl-2-(4-methylstyryl)-1,3,2-dioxaborolane (Z-**2b**): The title compound was

prepared following general procedure B (1 mol% of [Co]), the reaction mixture was stirred for 5 s before quenched. DBM was added as internal standard and NMR yield (>99%) was obtained. Purification by flash column chromatography on silica gel (eluent: petroleumether→ petroleumether/EtOAc =20/1) afforded the product as a pale yellow oil in 83% yield (80.4 mg, Z:E=98:2).

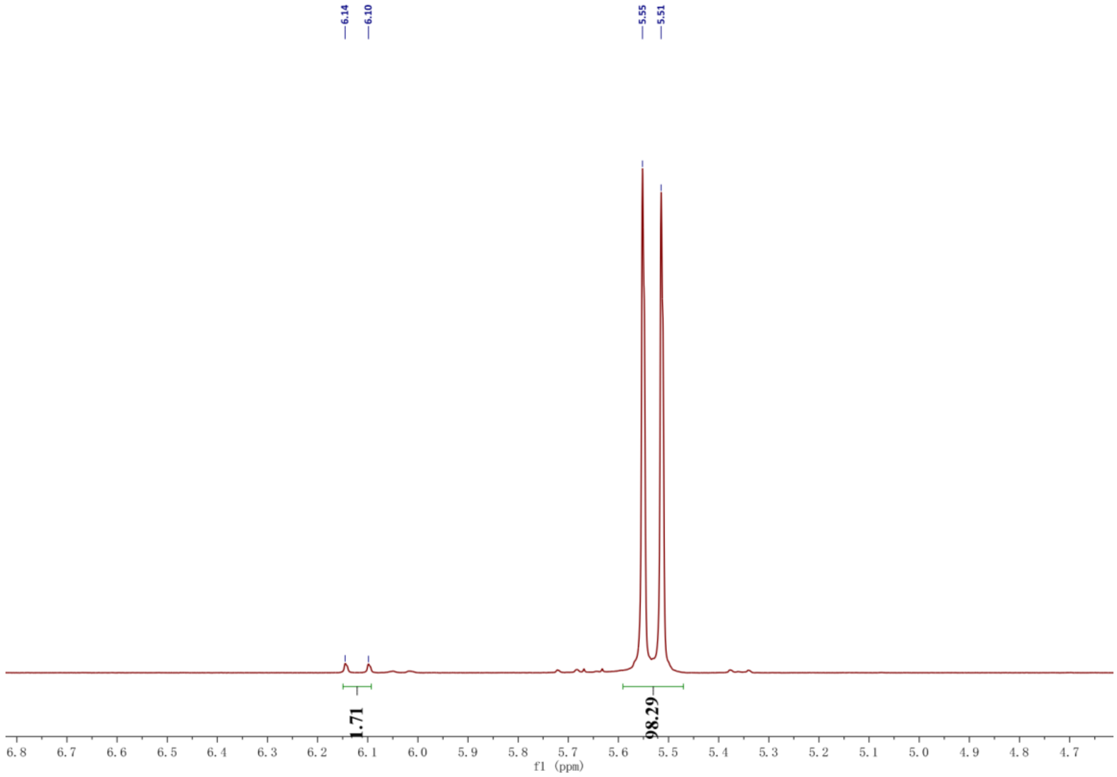

Z/E ratio of Z-**2b**

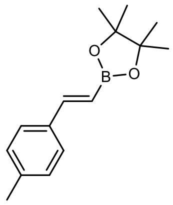

E-**2b**

(E)-4,4,5,5-tetramethyl-2-(4-methylstyryl)-1,3,2-dioxaborolane (E-**2b**): The title compound was prepared following general procedure B (1 mol% of [Co]), the reaction mixture was stirred for 24 h before quenched. Purification by flash column chromatography on silica gel (eluent: petroleumether→ petroleumether/EtOAc =20/1) afforded the product as a pale yellow oil in 93% yield (88.3 mg, E:Z>99:1). *See spectrum*

^{1}H NMR (400 MHz, Chloroform-*d*) δ 7.48 – 7.34 (m, 3H), 7.14 (d, *J* = 8.0 Hz, 2H), 6.12 (d, *J* = 18.5 Hz, 1H), 2.35 (s, 3H), 1.32 (s, 12H).

^{1}H NMR spectra are consistent with previously reported data[21].

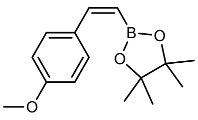

Z-**3b**

(Z)-2-(4-methoxystyryl)-4,4,5,5-tetramethyl-1,3,2-dioxaborolane (Z-**3b**): The title compound was prepared following general procedure B (0.5 mol% of [Co]), the reaction mixture was stirred for 5 s before quenched. DBM was added as internal standard and NMR yield (>99%) was obtained. Purification by flash column chromatography on silica gel (eluent: petroleumether→ petroleumether/EtOAc =20/1) afforded the product as a pale yellow oil in 92% yield (96.0 mg, Z:E=97:3). TOF = 132480 h^{-1}

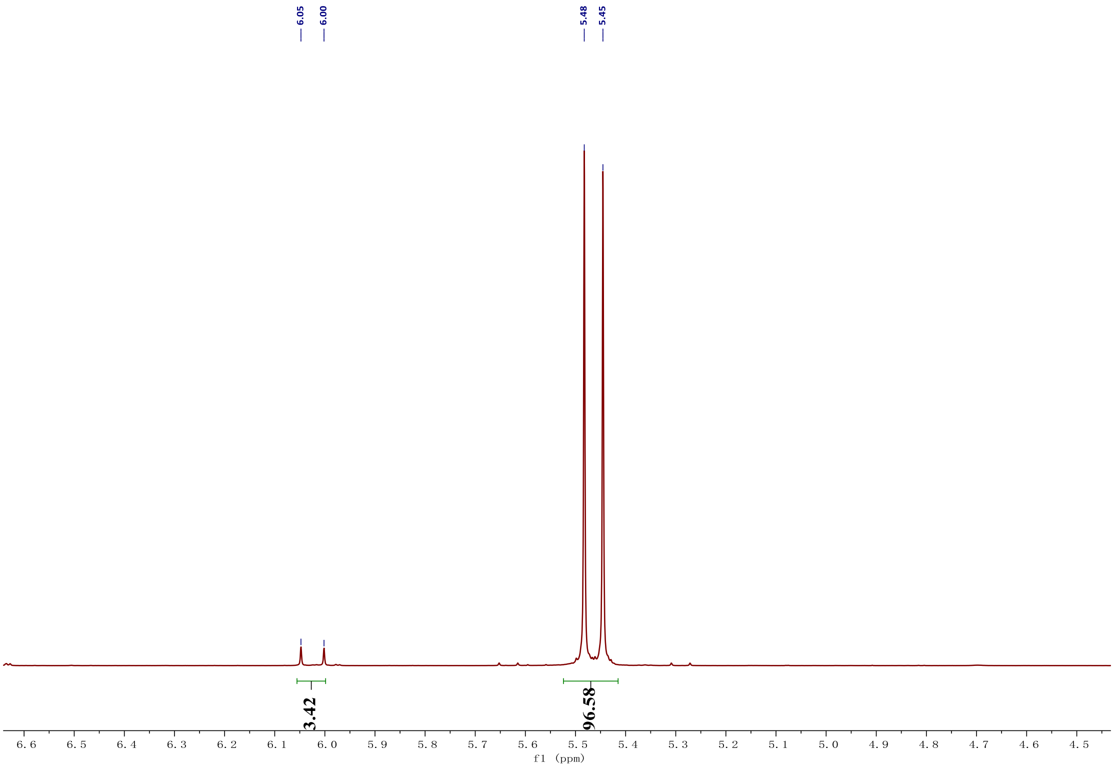

Z/E ratio of Z-**3b**

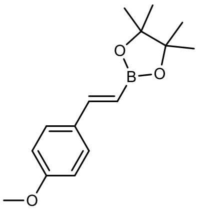

E-**3b**

(E)-4,4,5,5-tetramethyl-2-(4-methylstyryl)-1,3,2-dioxaborolane (E-**3b**): The title compound was prepared following general procedure B (0.5 mol% of [Co]), the reaction mixture was stirred for 24 h before quenched. Purification by flash column chromatography on silica gel (eluent: petroleumether→ petroleumether/EtOAc =20/1) afforded the product as a pale yellow oil in 92% yield (95.0 mg, E:Z>99:1). See spectrum

^{1}H NMR (400 MHz, Chloroform-*d*) δ 7.43 (d, *J* = 8.7 Hz, 2H), 7.35 (d, *J* = 18.4 Hz, 1H), 6.85 (d, *J* = 8.7 Hz, 2H), 6.01 (d, *J* = 18.4 Hz, 1H), 3.79 (s, 3H), 1.30 (s, 12H).

^{1}H NMR spectra are consistent with previously reported data[21].

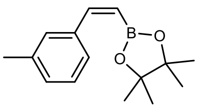

Z-**13b**

(Z)-4,4,5,5-tetramethyl-2-(3-methylstyryl)-1,3,2-dioxaborolane (Z-**13b**): The title compound was prepared following general procedure B (0.5 mol% of [Co]), the reaction mixture was stirred for 5 s before quenched. DBM was added as internal standard and NMR yield (>69%) was obtained. Purification by flash column chromatography on silica gel (eluent: petroleumether→ petroleumether/EtOAc =20/1) afforded the product as a pale yellow oil in 63% yield (80.0 mg, Z:E>99:1).

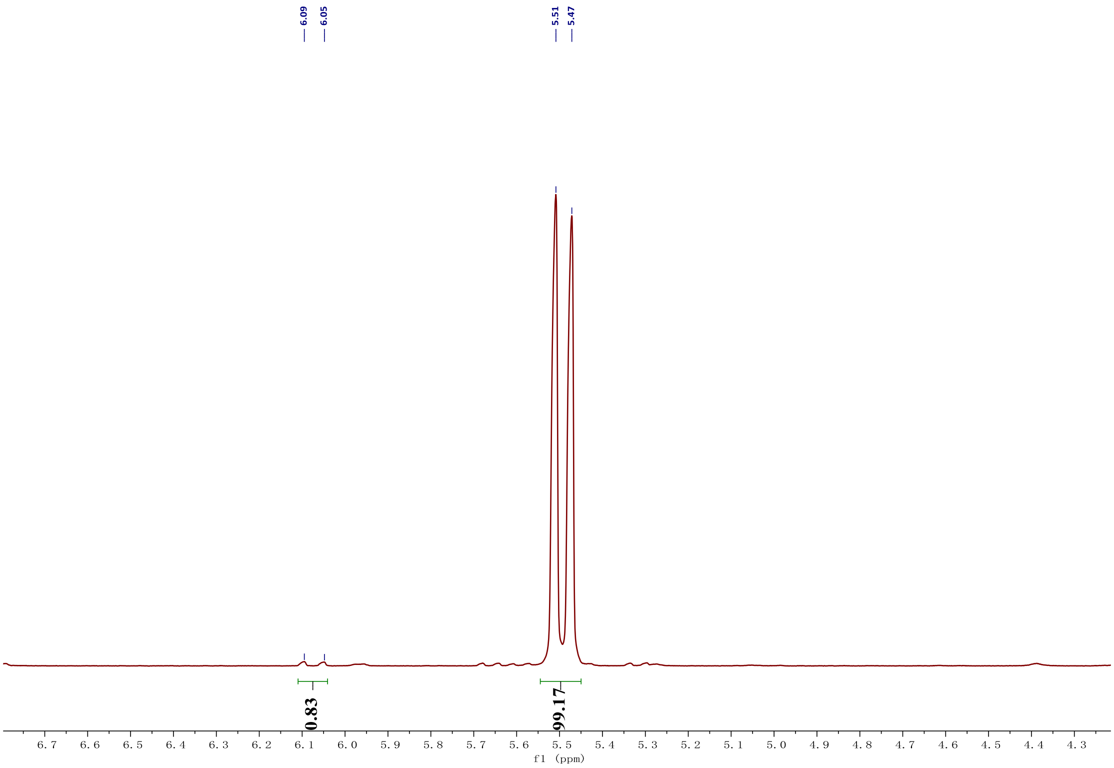

Z/E ratio of Z-**13b**

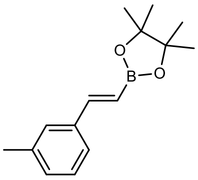

E-**13b**

(E)-4,4,5,5-tetramethyl-2-(4-methylstyryl)-1,3,2-dioxaborolane (E-**13b**): The title compound was prepared following general procedure B (0.5 mol% of [Co]), the reaction mixture was stirred for 24 h before quenched. Purification by flash column chromatography on silica gel (eluent: petroleumether→ petroleumether/EtOAc =20/1) afforded the product as a pale yellow oil in 93% yield (89.3mg, E:Z>99:1). See spectrum

^{1}H NMR (400 MHz, Chloroform-*d*) δ 7.30 (d, *J* = 18.4 Hz, 1H), 7.21 (d, *J* = 5.6 Hz, 2H), 7.14 (t, *J* = 7.8 Hz, 1H), 7.02 (d, *J* = 7.5 Hz, 1H), 6.07 (d, *J* = 18.4 Hz, 1H), 2.26 (s, 3H), 1.22 (s, 12H). ^{1}H NMR spectra are consistent with previously reported data[21].

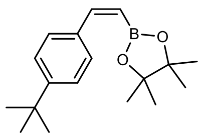

Z-**4b**

(Z)-2-(4-(tert-butyl)styryl)-4,4,5,5-tetramethyl-1,3,2-dioxaborolane (Z-**4b**): The title compound was prepared following general procedure C (5 mol% of [Co]), the reaction mixture was stirred for 3 mins before quenched. DBM was added as internal standard and NMR yield (>98%) was obtained. Purification by flash column chromatography on silica gel (eluent: petroleumether→ petroleumether/EtOAc =20/1) afforded the product as a pale yellow oil in 94% yield (107.6 mg, Z:E>99:1).

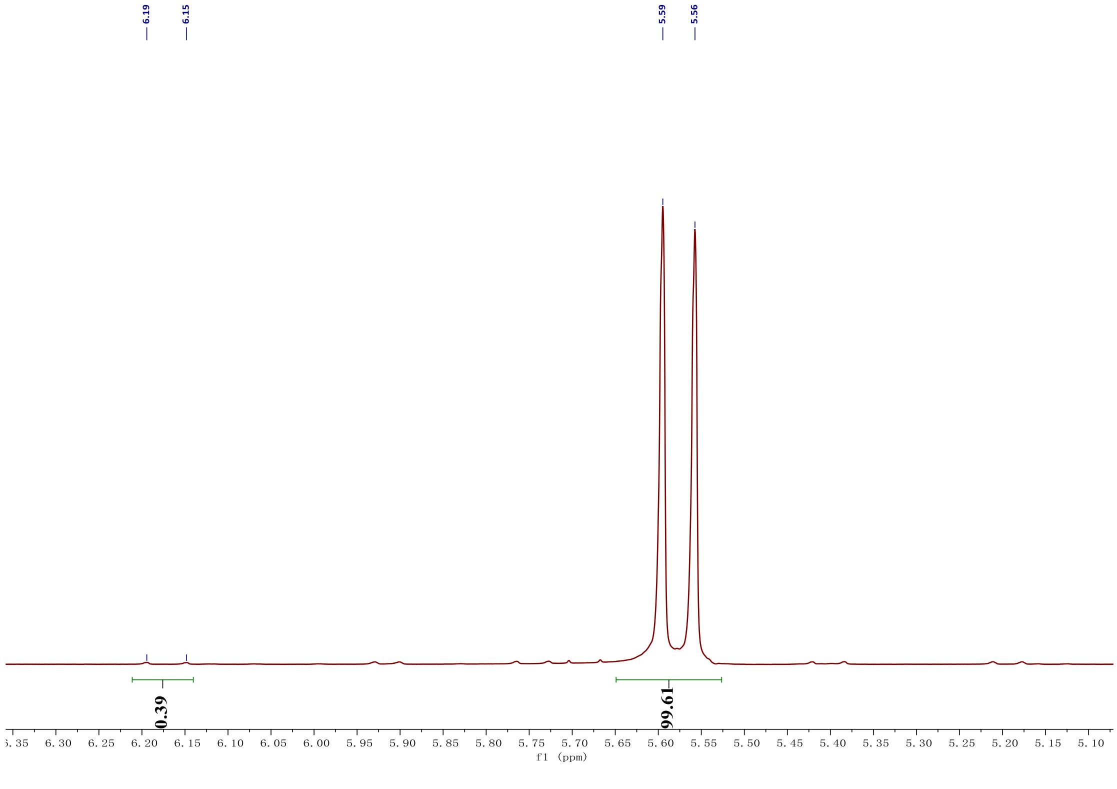

Z/E ratio of Z-**4b**

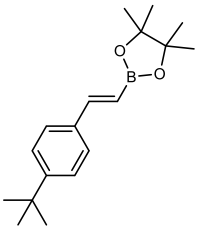

E-**4b**

(E)-2-(4-(tert-butyl)styryl)-4,4,5,5-tetramethyl-1,3,2-dioxaborolane (E-**4b**): The title compound was prepared following general procedure C (5 mol% of [Co]), the reaction mixture was stirred for 24 h before quenched. Purification by flash column chromatography on silica gel (eluent: petroleumether→ petroleumether/EtOAc =20/1) afforded the product as a pale yellow oil in 82% yield (91.6mg, E:Z>99:1). *See spectrum*

^{1}H NMR (400 MHz, Chloroform-*d*) δ 7.46 – 7.34 (m, 5H), 6.12 (d, *J* = 18.4 Hz, 1H), 1.32 (s, 21H).

^{1}H NMR spectra are consistent with previously reported data[22].

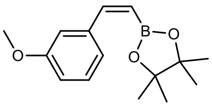

Z-**14b**

(Z)-2-(3-methoxystyryl)-4,4,5,5-tetramethyl-1,3,2-dioxaborolane (Z-**14b**): The title compound was prepared following general procedure C (5 mol% of [Co]), the reaction mixture was stirred for 5 mins before quenched. Purification by flash column chromatography on silica gel (eluent: petroleumether→ petroleumether/EtOAc =20/1) afforded the product as a pale yellow oil in 80% yield (76.7 mg, Z:E=92:8).

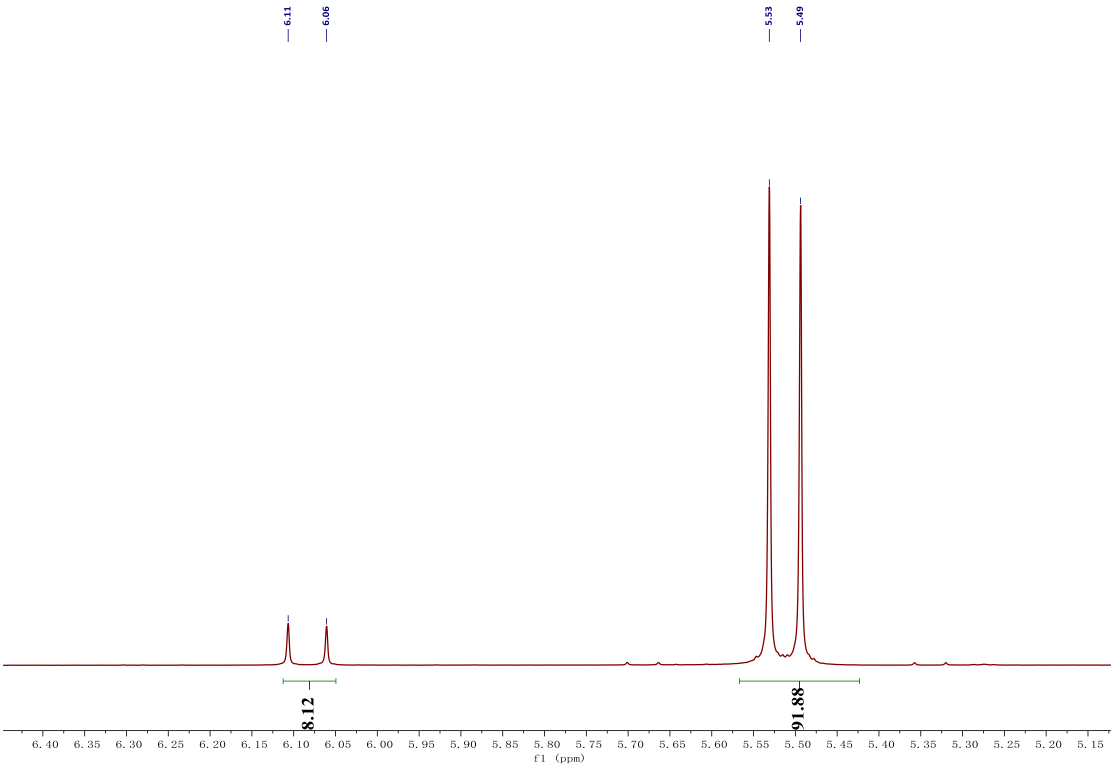

Z/E ratio of Z-**14b**

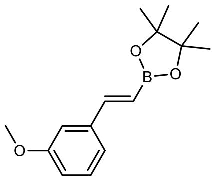

E-**14b**

(E)-2-(3-methoxystyryl)-4,4,5,5-tetramethyl-1,3,2-dioxaborolane (E-**14b**): The title compound was prepared following general procedure C (5 mol% of [Co]), the reaction mixture was stirred for 24 h before quenched. Purification by flash column chromatography on silica gel (eluent: petroleumether→ petroleumether/EtOAc =20/1) afforded the product as a pale yellow oil in 88% yield (86.9 mg, E:Z>99:1). See spectrum

^{1}H NMR (400 MHz, Chloroform-*d*) δ 7.29 (d, *J* = 18.4 Hz, 1H), 7.15 (t, *J* = 7.9 Hz, 1H), 6.99 (d, *J* = 7.7 Hz, 1H), 6.94 (s, 1H), 6.76 (d, *J* = 8.2 Hz, 1H), 6.07 (d, *J* = 18.4 Hz, 1H), 3.70 (s, 3H), 1.22 (s, 12H).

^{1}H NMR spectra are consistent with previously reported data[21].

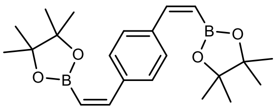

Z-**30b**

1,4-bis((Z)-2-(4,4,5,5-tetramethyl-1,3,2-dioxaborolan-2-yl)vinyl)benzene (Z-**30b**): The title compound was prepared following general procedure B (0.5 mol% of [Co], 0.2 mmol scale) , the reaction mixture was stirred for 1 hours. DBM was added as internal standard and NMR yield (85%) was obtained. Purification by flash column chromatography on silica gel (eluent: petroleumether→ petroleumether/EtOAc =20/1) afforded the product as a white solid in 82% yield (62.2 mg, Z:E=95:5).

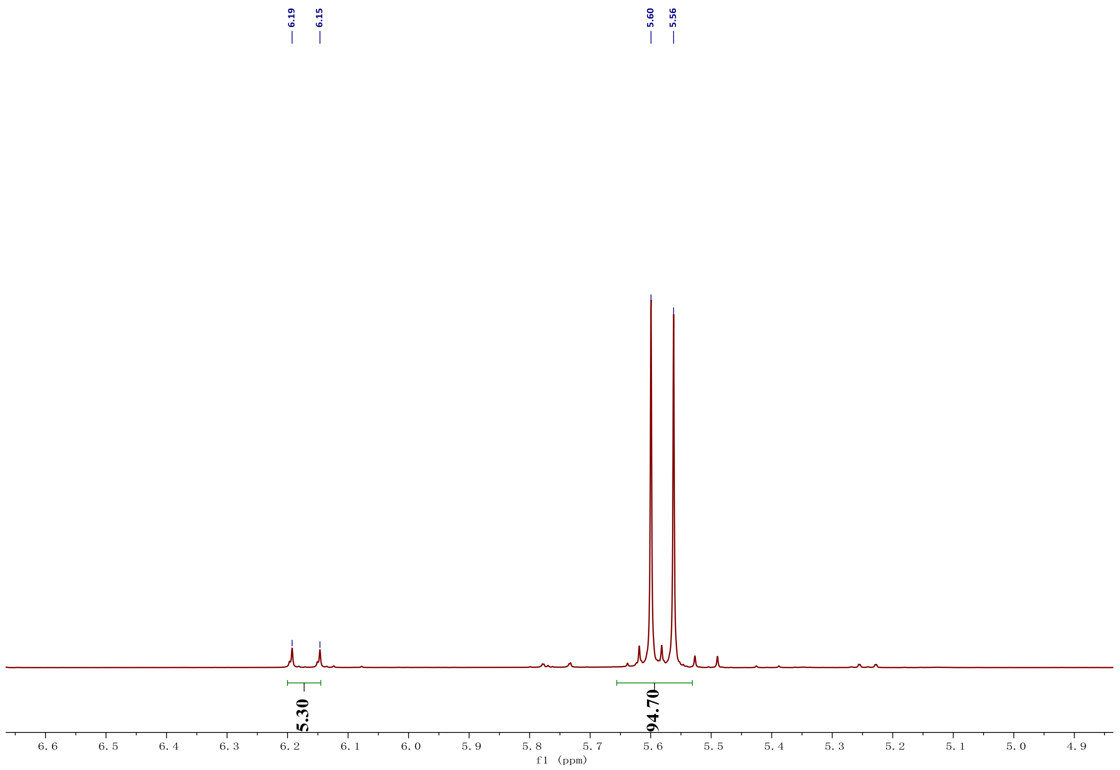

Z/E ratio of Z-**30b**

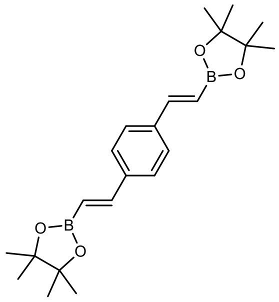

E-**30b**

1,4-bis((E)-2-(4,4,5,5-tetramethyl-1,3,2-dioxaborolan-2-yl)vinyl)benzene (E-**30b**): The title compound was prepared following general procedure B (0.5 mol% of [Co], 0.2 mmol scale), the reaction mixture was stirred for 24 h before quenched. DBM was added as internal standard and NMR yield (84%) was obtained. Purification by flash column chromatography on silica gel (eluent: petroleumether→ petroleumether/EtOAc =20/1) afforded the product as a pale yellow oil in 76% yield (58.2 mg, E:Z>99:1). *See spectrum*

^{1}H NMR (400 MHz, Chloroform-*d*) δ 7.45 (s, 4H), 7.37 (d, *J* = 18.4 Hz, 2H), 6.17 (d, *J* = 18.4 Hz, 2H), 1.31 (s, 24H).

^{1}H NMR spectra are consistent with previously reported data[22].

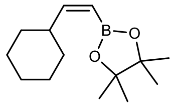

Z-**39b**

(Z)-2-(2-cyclohexylvinyl)-4,4,5,5-tetramethyl-1,3,2-dioxaborolane (Z-**39b**): The title compound

was prepared following general procedure C (5 mol% of [Co]), the reaction mixture was stirred for 1 hours. DBM was added as internal standard and NMR yield (90%) was obtained. Purification by flash column chromatography on silica gel (eluent: petroleumether→ petroleumether/EtOAc =20/1) afforded the product as a white solid in 88% yield (83.1 mg, Z:E=98:2).

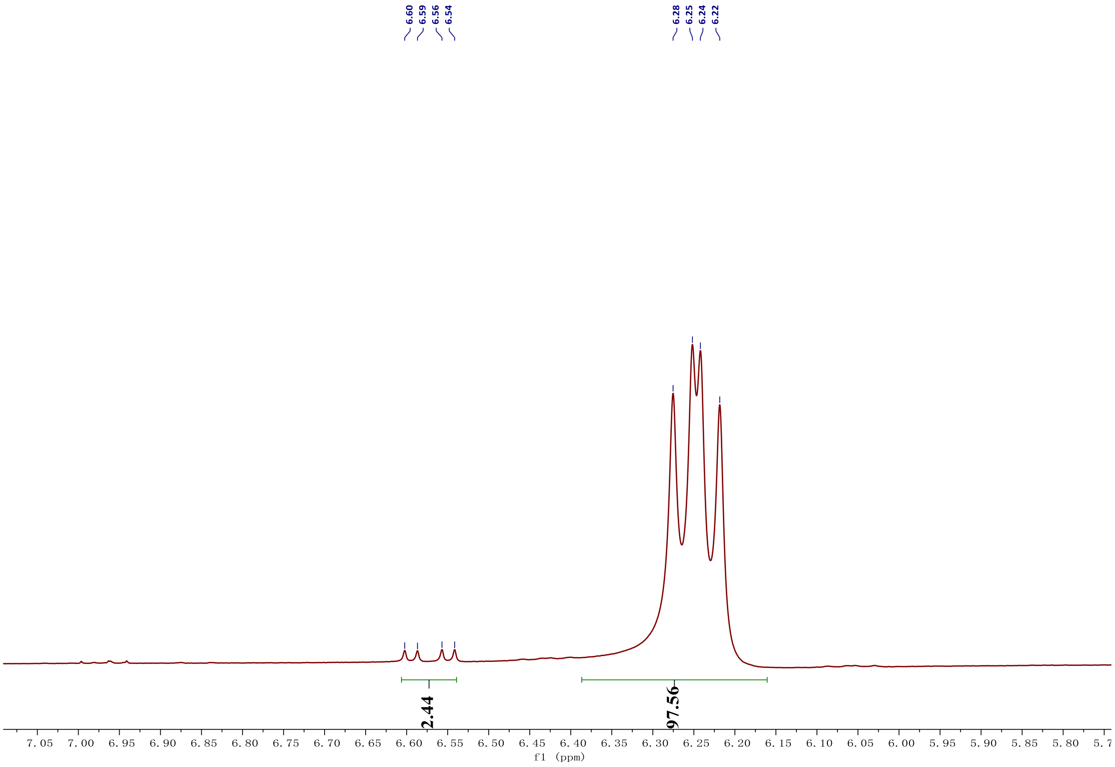

Z/E ratio of Z-**39b**

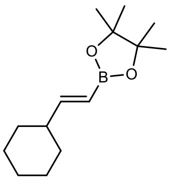

E-**39b**

(E)-2-(2-cyclohexylvinyl)-4,4,5,5-tetramethyl-1,3,2-dioxaborolane (E-**39b**): The title compound was prepared following general procedure B (0.5 mol% of [Co]), the reaction mixture was stirred for 24 h before quenched. DBM was added as internal standard and NMR yield (91%) was obtained. Purification by flash column chromatography on silica gel (eluent: petroleumether→ petroleumether/EtOAc =20/1) afforded the product as a pale yellow oil in 92% yield (58.2 mg, E:Z=97:3). *See spectrum*

^{1}H NMR (400 MHz, Chloroform-*d*) δ 6.57 (dd, *J* = 18.2, 6.2 Hz, 1H), 5.37 (dd, *J* = 18.2, 1.4 Hz, 1H), 2.07-1.96 (m, 1H), 1.77-1.68 (m, 4H), 1.67 – 1.64 (m, 1H), 1.26 (s, 12H), --1.20 – 1.03 (m, 4H), 0.99 – 0.70 (m, 1H).

^{1}H NMR spectra are consistent with previously reported data[21].

5. Large scale reaction

General procedure for large scale catalytic Z-selective hydroboration of terminal alkynes: In nitrogen atmosphere, a 100 mL Schlenk vial was charged with alkyne (30 mmol) and dry DMF (20 mL), the alkyne solution was placed in an ice path. In another vial charged with $Co(acac)_2$ (38.6 mg, 0.15 mmol), CNC-iPr (96.0 mg, 0.21 mmol), ^{t}BuOK (94.3 mg, 0.84 mmol), dry DMF (15 mL) was stirred for 5 min and HBpin (5.6 mL, 39 mmol) was added and stirred for a further 5 min. The catalyst solution was added dropwise into the alkyne solution under ice bath during 10 mins. After that, ice bath was removed and the mixture was stirred at room temperature for a certain time. The reaction was then quenched by adding water. 300 mL EtOAc was added and the organic phase was washed with 3*100 mL water and 2*100 mL brine. the organic phase was passed through a short pad of silica gel. Pure product was obtained after removing volatiles under reduced pressure.

6. Mechanistic Study

6.1 Procedure for monitoring the hydroboration of phenylacetylene

In a nitrogen atmosphere, a vail was charged with $Co(acac)_2$ (4.1 mg, 0.016 mmol), CNC-iPr (10.2 mg, 0.022 mmol), ^{t}BuOK (10.1 mg, 0.09 mmol) dissolved in dry DMF (4 mL) and was stirred for 5 min. 500 µL of the freshly prepared stock solution of the *in situ* prepared active catalyst ([Co]=4.0 mM in DMF) was added via a micro-syringe (500 µL) to a vial charged with HBpin (116µL, 0.8 mmol) and the mixture was stirred for 5 min. **1a** (0.4 mmol) was added rapidly. Periodically, an aliquot was removed from the vial and analyzed by GC-Ms.

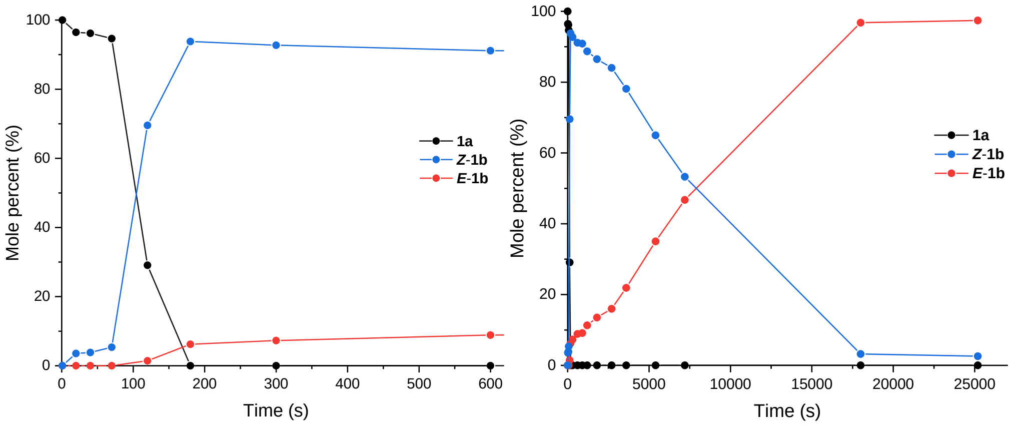

Supplementary Figure 1. Kinetic profile of the hydroboration of phenylacetylene (**1a**) with an equally distributed time axis

6.2 Procedure for kinetic experiments of catalyst concentration

In a nitrogen atmosphere, a vail was charged with $Co(acac)_2$ (4.1 mg, 0.016 mmol), CNC-^{i}Pr (10.2 mg, 0.022 mmol), ^{t}BuOK (10.1 mg, 0.09 mmol) dissolved in dry DMF (4 mL) and was stirred for 5 min. The freshly prepared stock solution of the *in situ* prepared active catalyst ([Co]=4.0 mM in DMF) was added via a micro-syringe (200 µL, 300 µL, 350 µL, 400 µL,450 µL, 500 µL) to a vial charged with HBpin (174µL, 1.2 mmol) and DMF (300 µL, 200 µL, 150 µL, 100 µL, 50 µL, 0 µL). The mixture was stirred for 5 min. Alkyne (0.4 mmol) was added rapidly. Periodically, an aliquot was removed from the vial and analyzed by GC-Ms.

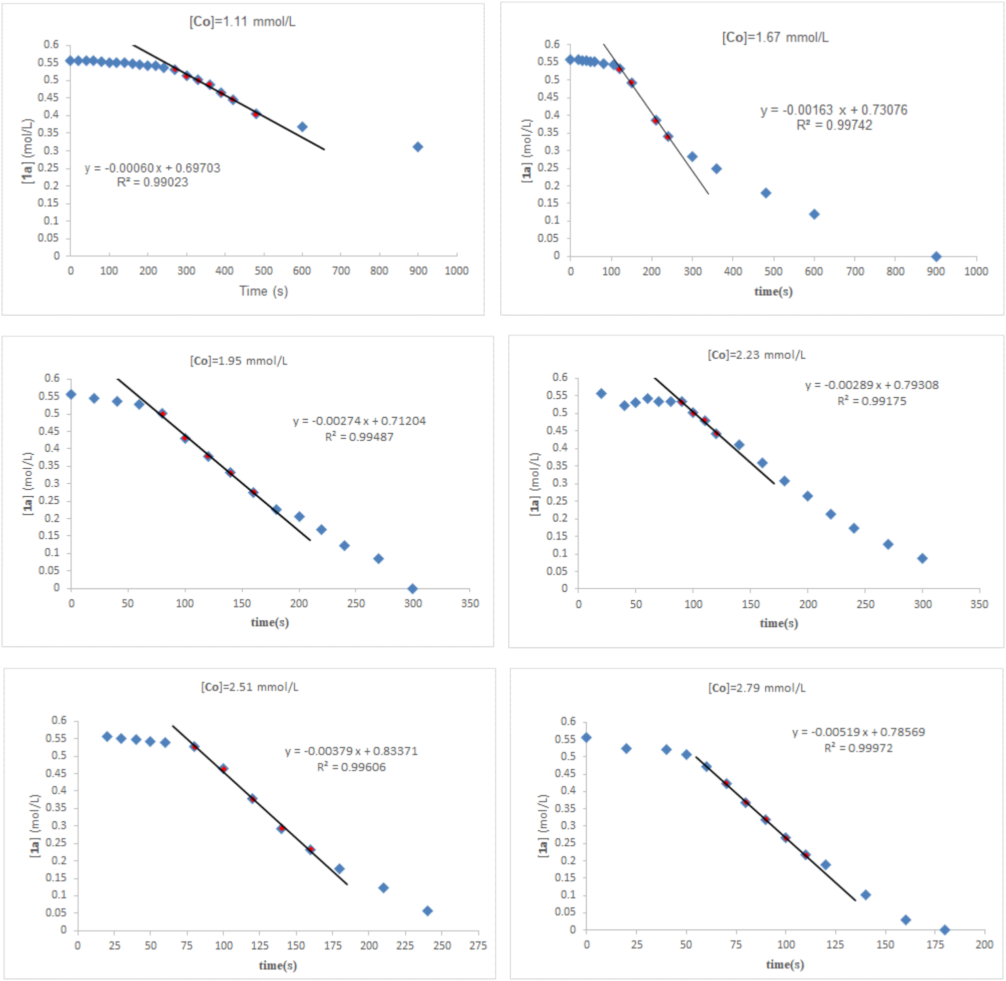

Supplementary Figure 2. The original kinetic data of **1a** with different cobalt concentrations.

6.3 Deuterium labeling experiments

Procedure for Z-selective hydroboration of 3a-d:

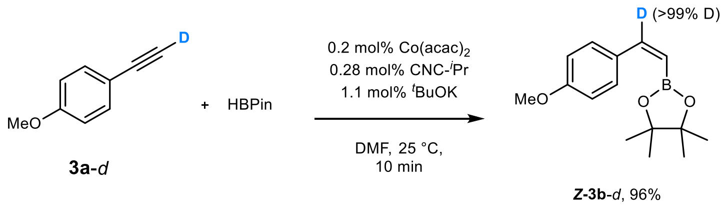

In nitrogen atmosphere, a vail was charged with Co(acac)$_2$ (4.1 mg, 0.016 mmol), CNC-iPr (10.2 mg, 0.022 mmol), ^{t}BuOK (10.1 mg, 0.09 mmol) dissolved in dry DMF (4 mL) and was stirred for 5 min. The freshly prepared stock solution of the *in situ* prepared active catalyst ([Co]=4.0 mM in DMF) was added via a micro-syringe (200 μL) to a vial charged with HBpin (75 μL, 0.52 mmol) and DMF (300 μL). The mixture was stirred for 5 mins. **3a-*d*** (0.4 mmol) was added rapidly and the resulting mixture was stirred for 10 min. The reaction was then quenched by adding water. 20 mL EtOAc was added and the organic phase was washed with 10 mL brine twice to remove most of DMF. Purification by flash column chromatography on silica gel (eluent: petroleumether→ petroleumether/EtOAc =20/1) over silica gel deactivated with 2% NEt$_3$ afforded the product as a pale yellow oil in 96% yield (100.3 mg, Z:E=98:2).

^{1}H NMR (400 MHz, $CDCl_3$) δ 7.55 (d, *J* = 8.7 Hz, 2H), 6.84 (d, *J* = 8.7 Hz, 2H), 5.45 (s, 1H), 3.82 (s, 3H), 1.30 (s, 12H).

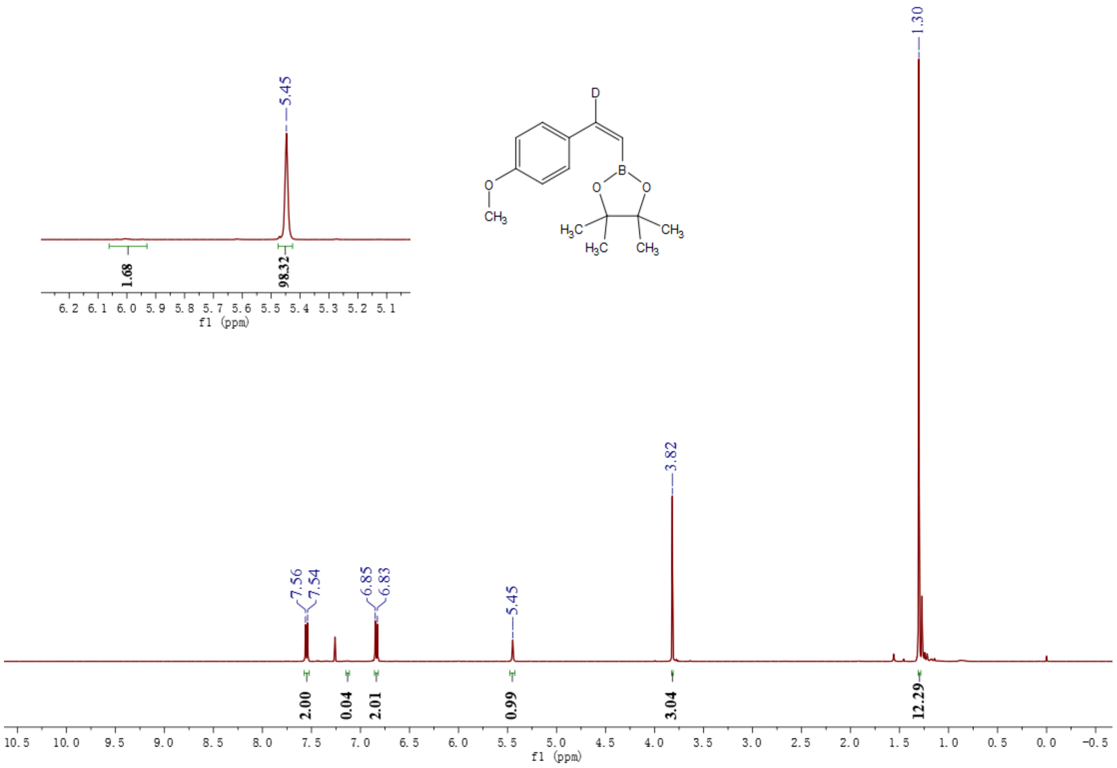

Supplementary Figure 3 ^{1}H NMR of *Z*-**3b**-*d*

Procedure for isomerization of *Z*-3b-*d*:

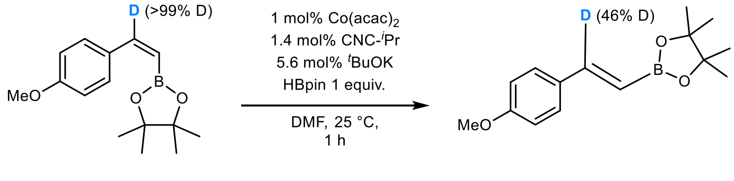

Z*-3b-*d ***E*-3b-*d***

In a nitrogen atmosphere, a vail was charged with $Co(acac)_2$ (4.1 mg, 0.016 mmol), CNC-^{i}Pr (10.2 mg, 0.022 mmol), ^{t}BuOK (10.1 mg, 0.09 mmol) dissolved in dry DMF (2 mL) and was stirred for 5 mins. The freshly prepared stock solution of the *in situ* prepared active catalyst ([Co]=8.0 mM in DMF) was added via a micro-syringe (500 μL) to a vial charged with HBpin (58 μL, 0.4 mmol). The mixture was stirred for 5 mins. *Z*-**3b-*d*** (0.4 mmol) was added rapidly and the resulting mixture was stirred for 1 h. The reaction was then quenched by adding water. 20 mL EtOAc was added and the organic phase was washed with 10 mL brine twice to remove most of DMF. Purification by flash column chromatography on silica gel (eluent: petroleumether→ petroleumether/EtOAc =20/1) over silica gel deactivated with 2% NEt_3 afforded the product as a pale yellow oil in 77% yield (80.4 mg, Z:E=98:2).

^{1}H NMR (600 MHz, Chloroform-*d*) δ 7.43 (d, *J* = 7.8 Hz, 2H), 7.35 (d, *J* = 18.2 Hz, 0.46 H), 6.86 (d, *J* = 7.5 Hz, 2H), 6.01 (d, *J* = 15.4 Hz, 1H), 3.80 (s, 3H), 1.30 (s, 12H).

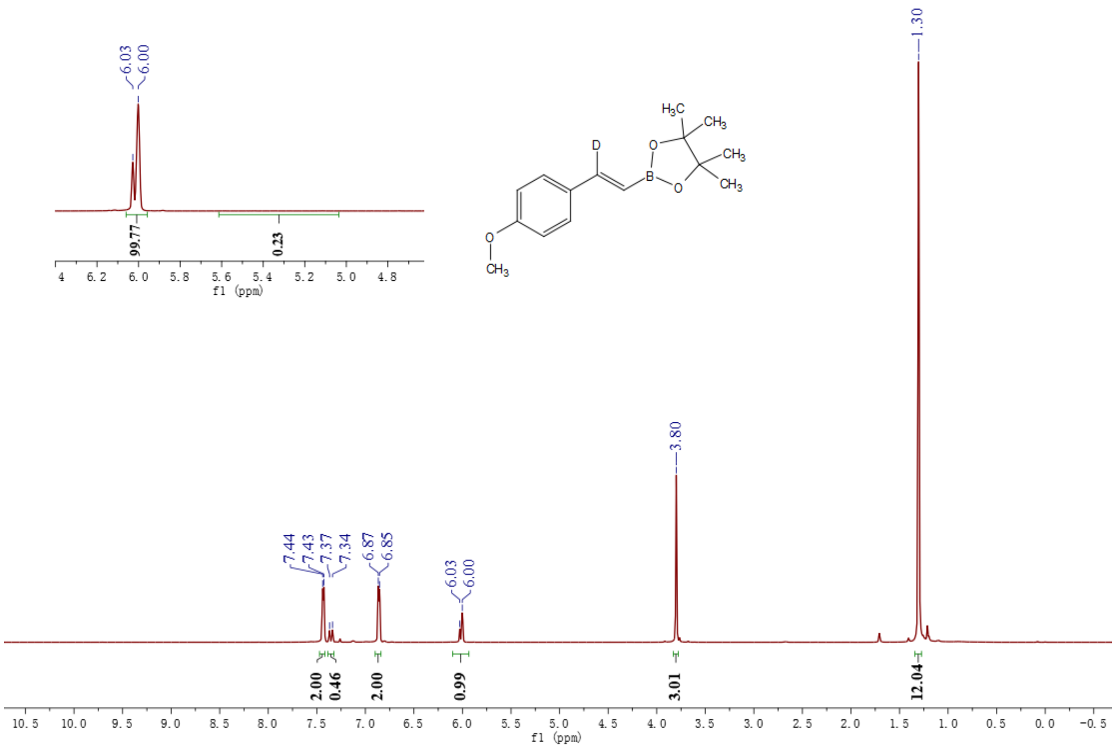

Supplementary Figure 4 ^{1}H NMR of *E*-**3b**-*d*

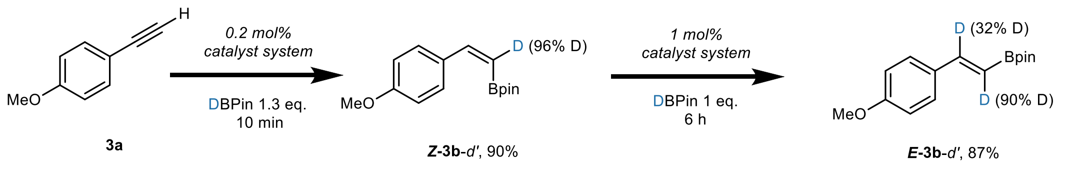

Supplementary Figure 5 Deuterium labeling experiments with DBpin

Procedure for Z-selective hydroboration of 3a with DBpin:

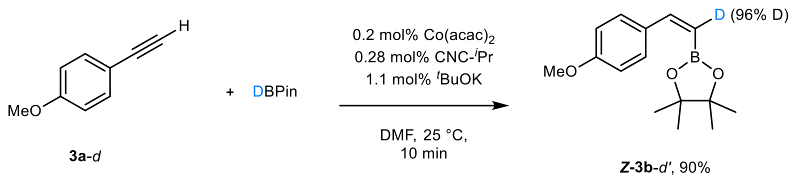

In a nitrogen atmosphere, a vail was charged with $Co(acac)_2$ (4.1 mg, 0.016 mmol), CNC-iPr (10.2 mg, 0.022 mmol), ^{t}BuOK (10.1 mg, 0.09 mmol) dissolved in dry DMF (4 mL) and was stirred for 5 min. The freshly prepared stock solution of the *in situ* prepared active catalyst ([Co]=4.0 mM in DMF) was added via a micro-syringe (200 µL) to a vial charged with DBpin (75 µL, 0.52 mmol) and DMF (300 µL). The mixture was stirred for 5 mins. **3a** (0.4 mmol) was added rapidly and the resulting mixture was stirred for 10 min. The reaction was then quenched by adding water. 20 mL EtOAc was added and the organic phase was washed with 10 mL brine twice to remove most of DMF. Purification by flash column chromatography on silica gel (eluent: petroleumether→ petroleumether/EtOAc =20/1) over silica gel deactivated with 2% NEt_3 afforded the product as a colorless oil in 90% yield (94.1 mg, Z:E>99:1).

^{1}H NMR (400 MHz, $CDCl_3$) δ 7.55 (d, *J* = 8.7 Hz, 2H), 7.15 (s, 1H), 6.84 (d, *J* = 8.8 Hz, 2H), 3.82 (s, 3H), 1.30 (s, 12H).

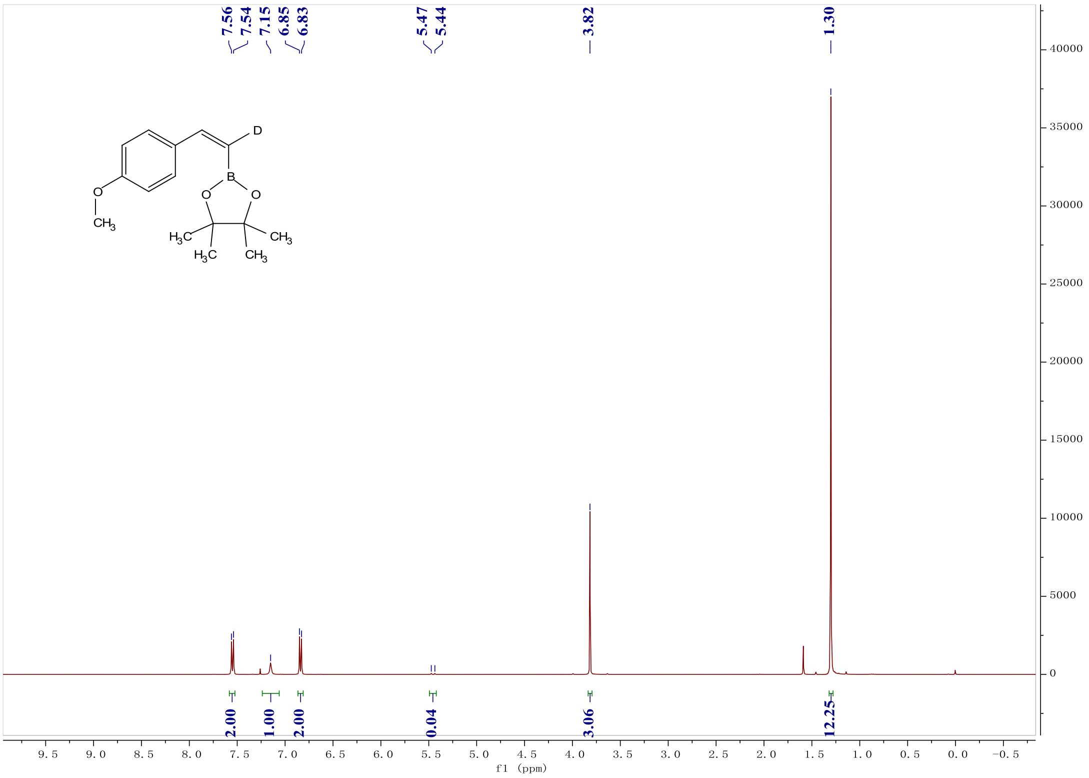

Supplementary Figure 6 ^{1}H NMR of *Z*-**3b**-*d'*

Procedure for isomerization of *Z*-3b-*d'* with DBpin:

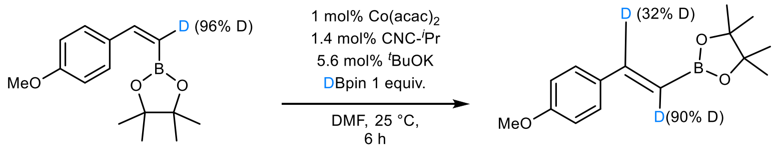

***Z*-3b**-*d'*, 90%

***E*-3b**-*d'*

In a nitrogen atmosphere, a vail was charged with $Co(acac)_2$ (4.1 mg, 0.016 mmol), CNC-^{i}Pr (10.2 mg, 0.022 mmol), ^{t}BuOK (10.1 mg, 0.09 mmol) dissolved in dry DMF (2 mL) and was stirred for 5 mins. The freshly prepared stock solution of the *in situ* prepared active catalyst ([Co]=8.0 mM in DMF) was added via a micro-syringe (500 µL) to a vial charged with DBpin (58 µL, 0.4 mmol). The mixture was stirred for 5 mins. *Z*-**3b**-***d'*** (0.4 mmol) was added rapidly and the resulting mixture was stirred for 6 h. The reaction was then quenched by adding water. 20 mL EtOAc was added and the organic phase was washed with 10 mL brine twice to remove most of DMF. Purification by flash column chromatography on silica gel (eluent: petroleumether→ petroleumether/EtOAc =20/1) over silica gel deactivated with 2% NEt_3 afforded the product as a pale yellow oil in 87% yield (91.3 mg, Z:E>99:1).

^{1}H NMR (400 MHz, Chloroform-*d*) δ 7.46 (d, *J* = 8.7 Hz, 2H), 7.37 (s, 1H), 6.89 (d, *J* = 8.7 Hz, 2H), 3.83 (s, 3H), 1.33 (s, 12H).

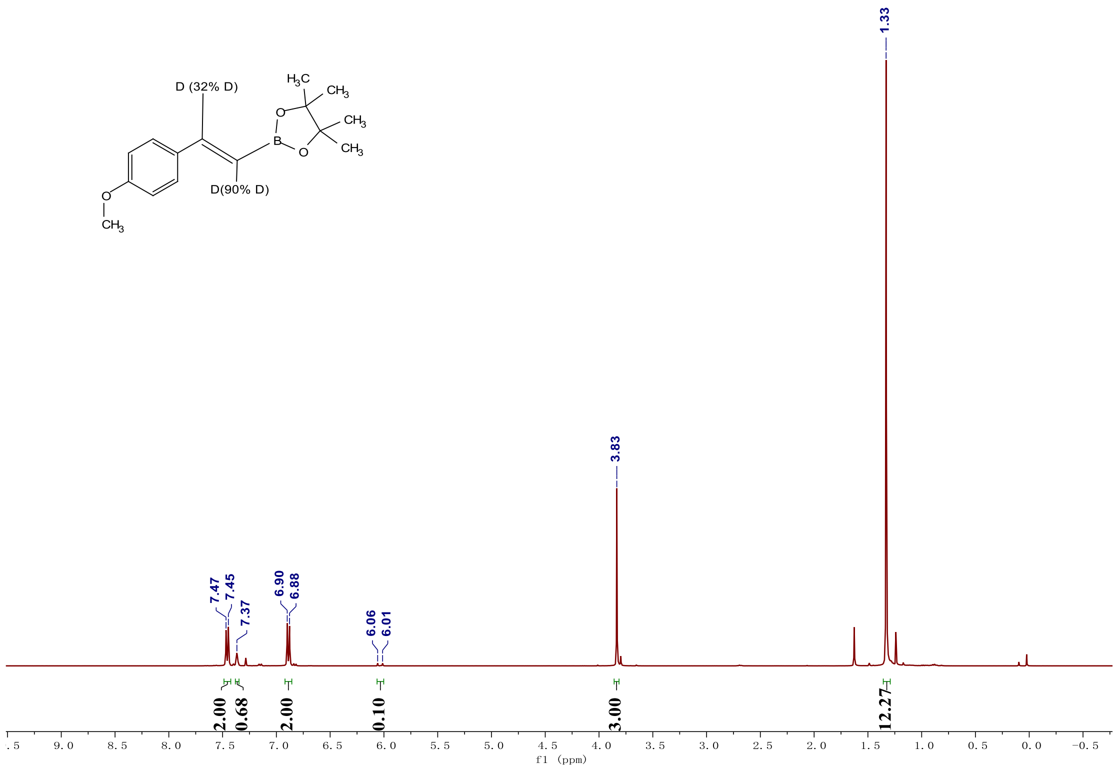

Supplementary Figure 7 ^{1}H NMR of *E*-**3b**-*d'*

Procedure for competition experiment:

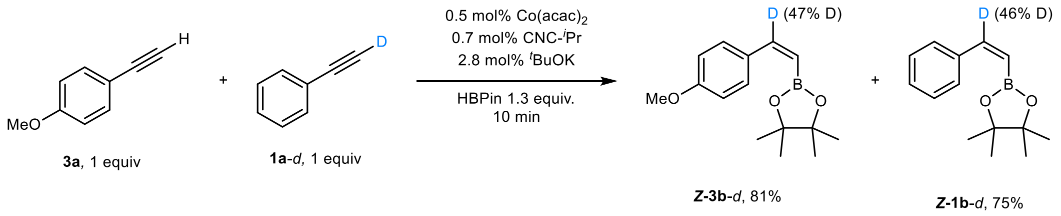

In a nitrogen atmosphere, a vail was charged with Co(acac)$_2$ (4.1 mg, 0.016 mmol), CNC-iPr (10.2 mg, 0.022 mmol), ^{t}BuOK (10.1 mg, 0.09 mmol) dissolved in dry DMF (4 mL) and was stirred for 5 mins. The freshly prepared stock solution of the *in situ* prepared active catalyst ([Co]=4.0 mM in DMF) was added via a micro-syringe (500 μL) to a vial charged with HBpin (75 μL, 0.52 mmol). The mixture was stirred for 5 mins. A mixture of **1a-*d*** (0.2 mmol) and **3a** (0.2 mmol) was added rapidly and the resulting mixture was stirred for 10 min. The reaction was then quenched by adding water. 20 mL EtOAc was added and the organic phase was washed with 10 mL brine twice to remove most of DMF. Purification by flash column chromatography on silica gel (eluent: petroleumether→ petroleumether/EtOAc =20/1) over silica gel deactivated with 2% NEt$_3$ afforded the ***Z*-3b-*d*** in 81% yield and ***Z*-1b-*d*** in 75% yield.

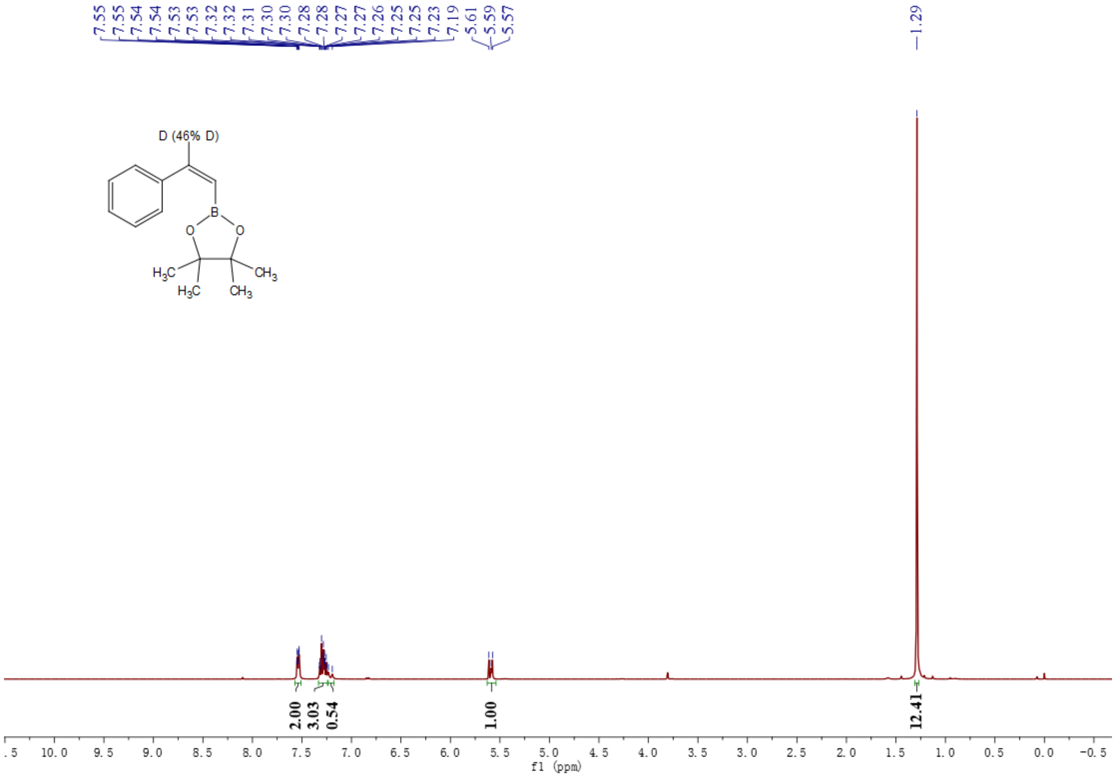

Supplementary Figure 8 ^{1}H NMR of *Z*-**1b**-*d*

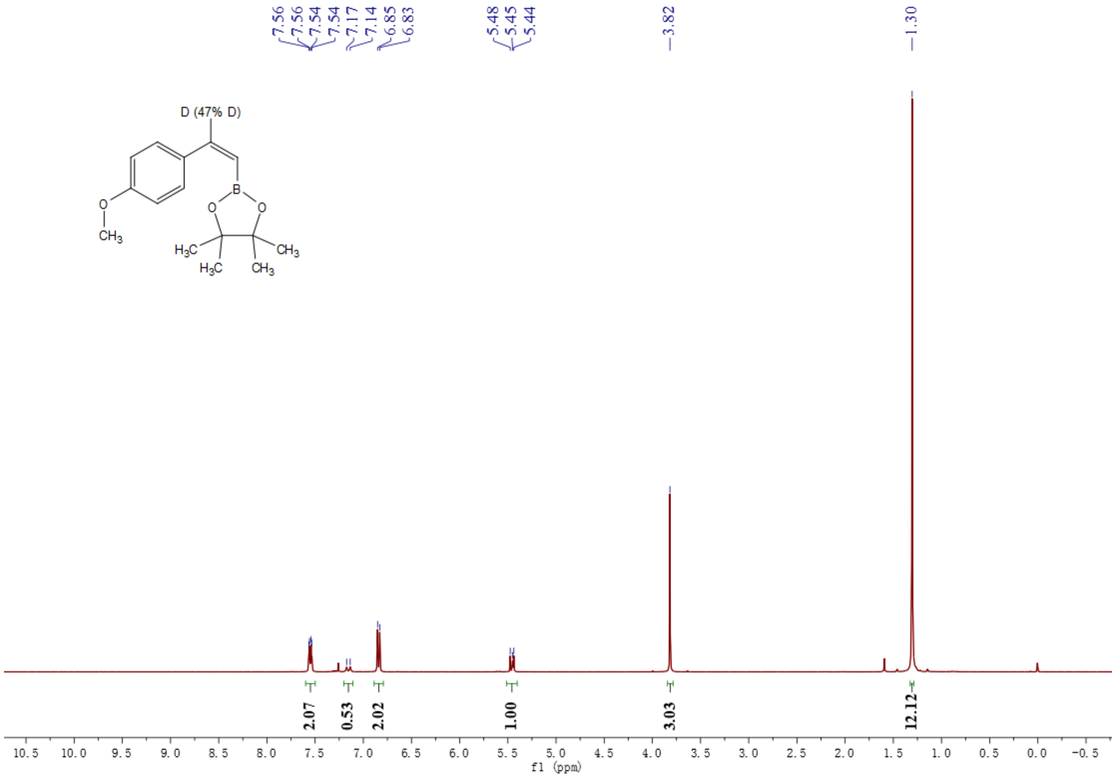

Supplementary Figure 9 ^{1}H NMR of *Z*-**3b**-*d*

6.4 Kinetic isotope effects experiment:

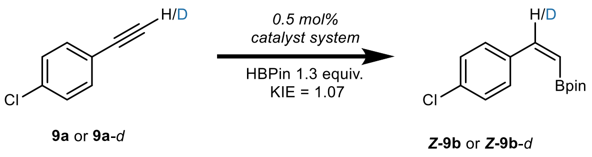

In a nitrogen atmosphere, a vail was charged with $Co(acac)_2$ (4.1 mg, 0.016 mmol), CNC-^{i}Pr (10.2 mg, 0.022 mmol), ^{t}BuOK (10.1 mg, 0.09 mmol) dissolved in dry DMF (4 mL) and was stirred for 5 min. The freshly prepared stock solution of the *in situ* prepared active catalyst ([Co]=4.0 mM in DMF) was added via a micro-syringe (500 μL) to a vial charged with HBpin (0.52 mmol). The mixture was stirred for 5 min. **9a** or **9a-*d*** (0.4 mmol) was added rapidly. Periodically, an aliquot was removed from the vial and analyzed by GC-Ms. The amount of the alkynes was traced (**Supplementary Figure 10**).

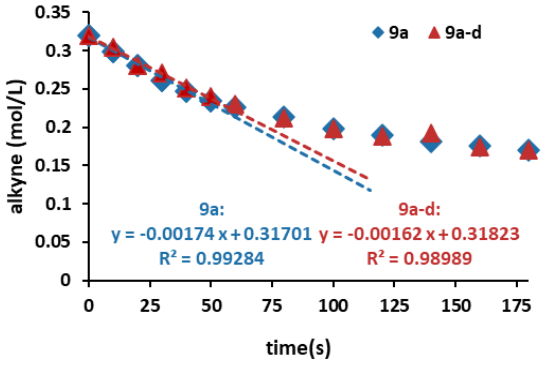

Supplementary Figure 10 Kinetic profile of the hydroboration of **9a** and **9a-d**

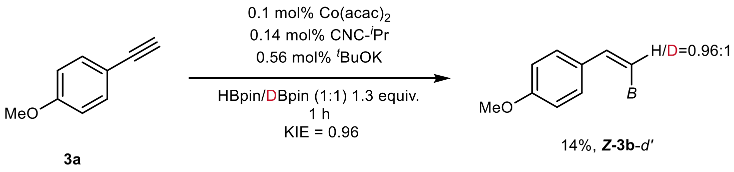

In a nitrogen atmosphere, a vail was charged with $Co(acac)_2$ (4.1 mg, 0.016 mmol), CNC-iPr (10.2 mg, 0.022 mmol), ^{t}BuOK (10.1 mg, 0.09 mmol) dissolved in dry DMF (4 mL) and was stirred for 5 mins. The freshly prepared stock solution of the *in situ* prepared active catalyst ([Co]=4.0 mM in DMF) was added via a micro-syringe (100 μL) to a vial charged with H/D-Bpin (H:D=1:1, 0.52 mmol) and DMF (400 μL). The mixture was stirred for 5 mins. **3a** (0.4 mmol) was added rapidly and the resulting mixture was stirred for 1 h. The reaction was then quenched by adding water. 20 mL EtOAc was added and the organic phase was washed with 10 mL brine twice to remove most of DMF. Purification by flash column chromatography on silica gel (eluent: petroleumether→ petroleumether/EtOAc =20/1) over silica gel deactivated with 2% NEt_3 afforded the product as a pale yellow oil in 22% yield (22.6 mg, Z:E=98:2).

^{1}H NMR (600 MHz, Chloroform-*d*) δ 7.54 (d, *J* = 8.5 Hz, 2H), 7.15 (t, *J* = 7.0 Hz, 1H), 6.84 (d, *J* = 8.0 Hz, 2H), 5.45 (d, *J* = 14.9 Hz, 0.49H), 3.82 (s, 3H), 1.30 (s, 12H).

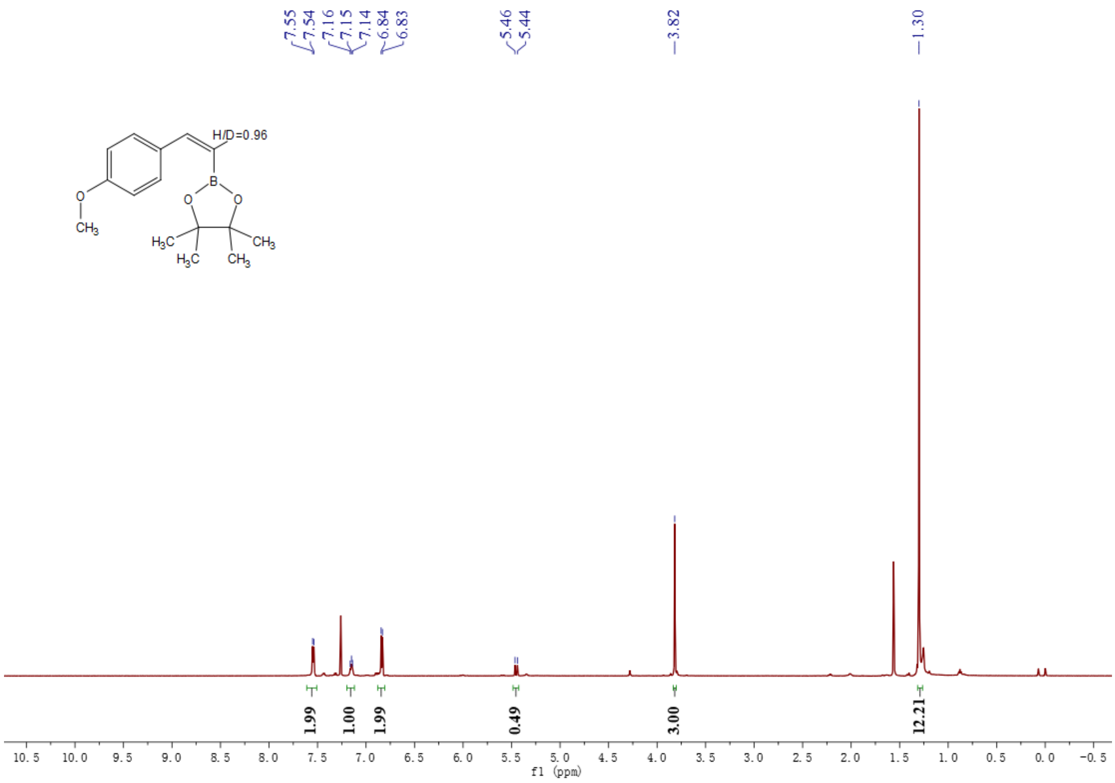

Supplementary Figure 11 ^{1}H NMR of Z-**3b**-*d'*

6.5 Inhabitation experiment with inert alkyne:

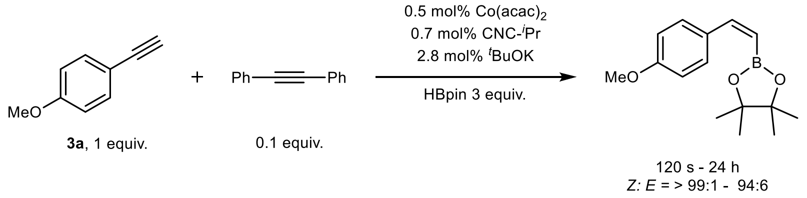

In a nitrogen atmosphere, a vail was charged with Co(acac)$_2$ (4.1 mg, 0.016 mmol), CNC-^{i}Pr (10.2 mg, 0.022 mmol), ^{t}BuOK (10.1 mg, 0.09 mmol) dissolved in dry DMF (4 mL) and was stirred for 5 mins. The freshly prepared stock solution of the *in situ* prepared active catalyst ([Co]=4.0 mM in DMF) was added via a micro-syringe (500 µL) to a vial charged with HBpin (174 µL, 1.2 mmol) and diphenylacetylene (7.1 mg, 0.04 mmol). The mixture was stirred for 5 mins. **3a** (0.4 mmol) was added rapidly. Periodically, an aliquot was removed from the vial and analyzed by GC-Ms.

6.6 Kinetic trace of Z-selective products:

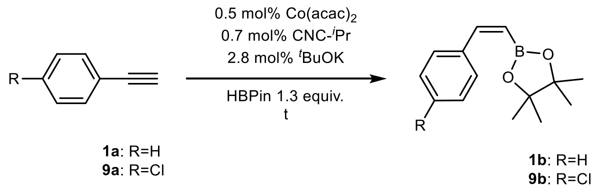

In a nitrogen atmosphere, a vail was charged with Co(acac)$_2$ (4.1 mg, 0.016 mmol), CNC-^{i}Pr (10.2 mg, 0.022 mmol), ^{t}BuOK (10.1 mg, 0.09 mmol) dissolved in dry DMF (4 mL) and was stirred for 5 mins. The freshly prepared stock solution of the *in situ* prepared active catalyst ([Co]=4.0 mM in DMF) was added via a micro-syringe (500 µL) to a vial charged with HBpin (174 µL, 1.2 mmol).

The mixture was stirred for 5 mins. **1a** (0.4 mmol) or **9a** (0.4 mmol) or a mixture of **1a** (0.2 mmol) and **9a** (0.2 mmol) was added rapidly. Periodically, an aliquot was removed from the vial and analyzed by GC-Ms.

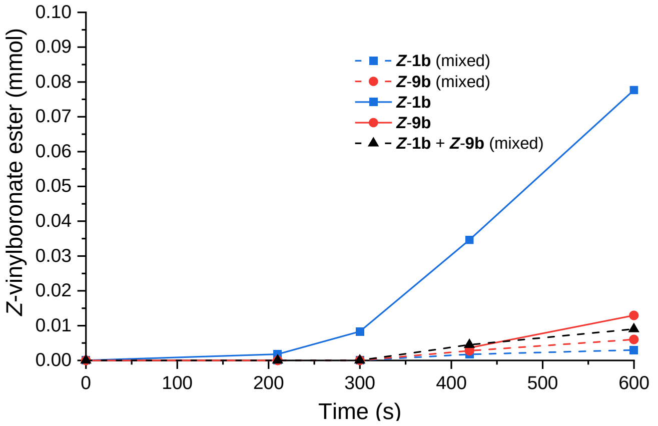

Supplementary Figure 12 The first 600 seconds of Fig. 5c

6.7 HRMS of in situ formed (CNC-^{i}Pr)Co(II) complex:

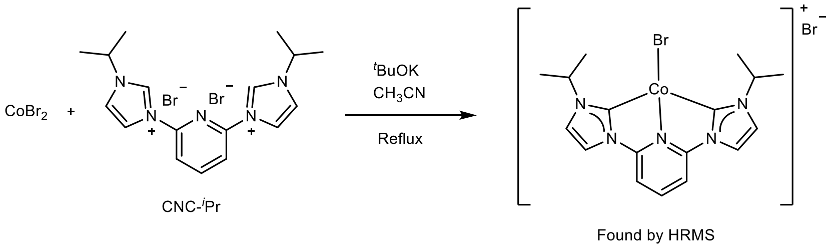

In a nitrogen atmosphere, a vail was charged with $CoBr_2$ (4.37 mg, 0.02 mmol), CNC-^{i}Pr (10.2 mg, 0.022 mmol), ^{t}BuOK (10.1 mg, 0.09 mmol) dissolved in dry acetonitrile and was stirred under 80 ℃. After cooling down, the mixture was filtrated and the residue was washed with acetonitrile, pentane. A grass green solid was obtained after dried in vacuo. The solid was dissolved in DMF and HRMS(APCI+) showed a (CNC-^{i}Pr)Co(II) complex was formed.

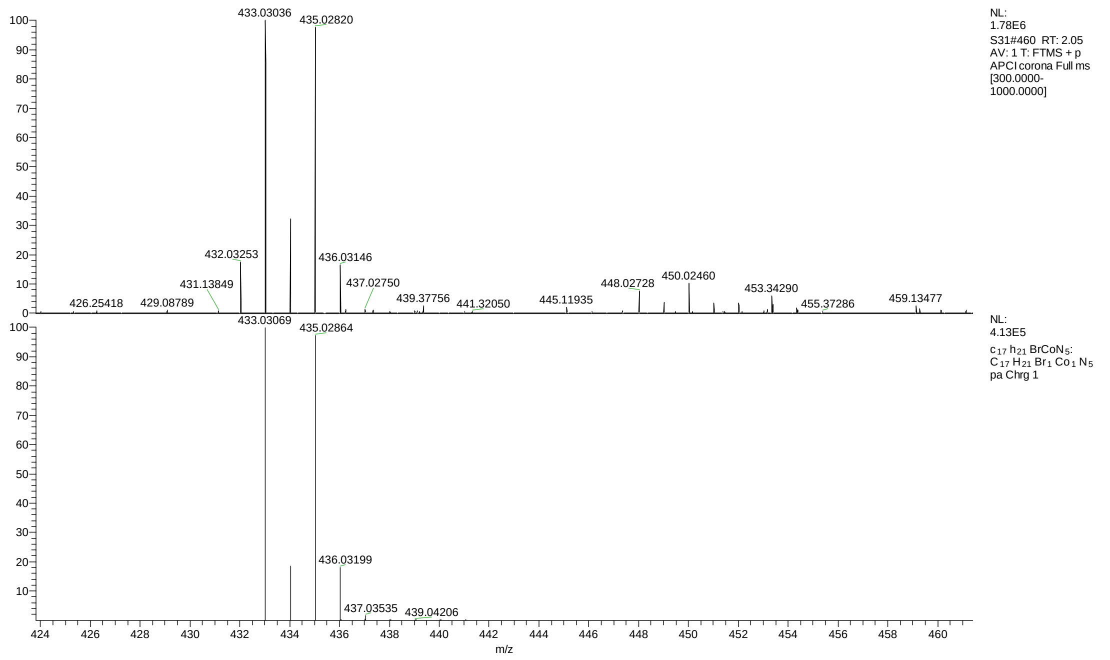

Supplementary Figure 13 HRMS(APCI+) of (CNC-iPr)Co(II) complex founded(up) and calculated(down)

6.8 Single crystal of (CNC-^{i}Pr)$_2$Co(III) complex

Method A: Single-crystals of (CNC-^{i}Pr)$_2$Co(III)Br$_3$ suitable for X-ray diffraction were obtained by slow vapor diffusion of diethyl ether into a 200 µL MeOH solution of the aforementioned grass green solid for two weeks*. The X-ray diffraction structure is shown in Supplementary Figure 14. See also the data in Supplementary Table 13-15. The X-ray crystallographic coordinate for the structure reported has been deposited at the Cambridge Crystallographic Data Centre (CCDC), under deposition number CCDC 2288260. No CheckCif file A- or B-level alerts was found.

* Note: the Co(III) complex crystal can also be obtained using alternative solvent systems such as EtOAc/DMF or EtOAc/DMSO.

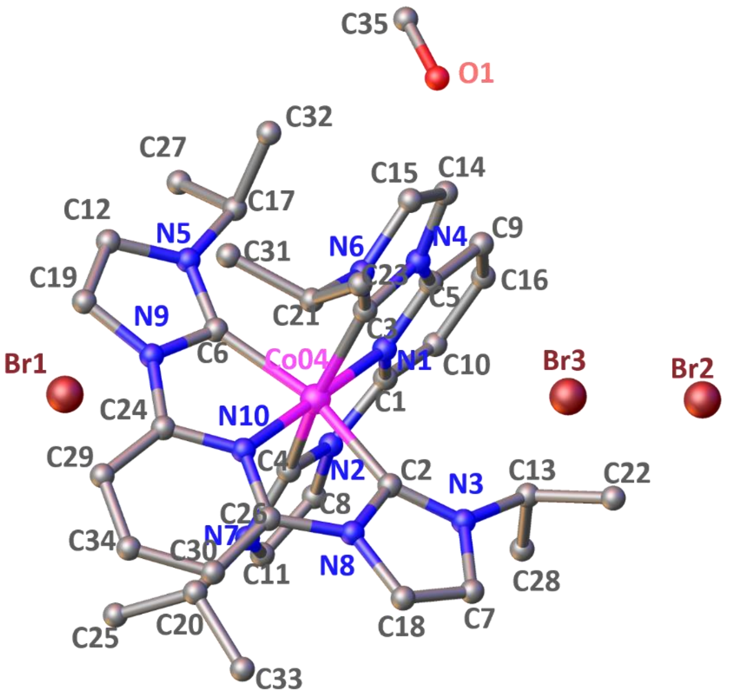

Supplementary Figure 14 X-ray structure of (CNC-^{i}Pr)$_2$CoBr$_3$ complex. Hydrogen atoms are omitted for clarity.

Supplementary Table 13 Crystal data and structure refinement for (CNC-^{i}Pr)$_2$Co(III)Br$_3$

Identification code	(CNC-^{i}Pr)$_2$Co(III)Br$_3$
Empirical formula	$C_{35}H_{46}Br_3CoN_{10}O$
Formula weight	921.48
Temperature/K	250.00(10)
Crystal system	monoclinic
Space group	$P2_1/n$
a/Å	10.34180(10)
b/Å	22.2032(2)
c/Å	16.6291(2)
α/°	90
β/°	96.1060(10)
γ/°	90
Volume/Å^3	3796.73(7)
Z	4

ρ_{calc}g/cm^3	1.612
μ/mm^{-1}	7.587
F(000)	1864
Crystal size/mm^3	0.2 × 0.15 × 0.15
Radiation	CuKα (λ = 1.54184)
2Θ range for data collection/ °	6.666 to 144.846
Index ranges	-12 ≤ h ≤ 12, -21 ≤ k ≤ 27, -20 ≤ l ≤ 20
Reflections collected	21911
Independent reflections	7394 [R_{int} = 0.0669, R_{sigma} = 0.0469]
Data/restraints/parameters	7394/6/461
Goodness-of-fit on F^2	1.065
Final R indexes [I>=2σ (I)]	R_1 = 0.0683, Wr_2 = 0.1858
Final R indexes [all data]	R_1 = 0.0742, Wr_2 = 0.1934
Largest diff. peak/hole / e Å^{-3}	1.86/-1.33

Supplementary Table 14 Bond Lengths for (CNC-^{i}Pr)$_2$Co(III)Br$_3$

Atom	Atom	Length/Å	Atom	Atom	Length/Å
Co04	N1	1.893(3)	N8	C26	1.389(7)
Co04	N10	1.895(4)	N9	C6	1.363(6)
Co04	C2	1.961(4)	N9	C19	1.391(6)
Co04	C3	1.968(5)	N9	C24	1.402(7)
Co04	C4	1.977(5)	N10	C24	1.339(7)
Co04	C6	1.965(4)	N10	C26	1.347(7)
N1	C1	1.335(5)	C1	C10	1.382(6)
N1	C5	1.340(5)	C5	C9	1.376(6)
N2	C1	1.390(5)	C7	C18	1.326(7)
N2	C4	1.377(5)	C8	C11	1.333(7)
N2	C8	1.395(6)	C9	C16	1.360(7)
N3	C2	1.331(5)	C10	C16	1.392(7)
N3	C7	1.408(6)	C12	C19	1.350(8)
N3	C13	1.489(6)	C13	C22	1.519(8)
N4	C3	1.376(6)	C13	C28	1.517(8)
N4	C5	1.398(5)	C14	C15	1.339(7)
N4	C14	1.383(6)	C17	C27	1.516(8)
N5	C6	1.337(6)	C17	C32	1.504(8)
N5	C12	1.390(6)	C20	C25	1.507(7)
N5	C17	1.477(6)	C20	C33	1.504(8)
N6	C3	1.340(6)	C21	C23	1.514(9)
N6	C15	1.387(7)	C21	C31	1.518(9)
N6	C21	1.500(6)	C24	C29	1.395(8)
N7	C4	1.335(6)	C26	C30	1.401(8)
N7	C11	1.398(7)	C29	C34	1.387(10)
N7	C20	1.482(6)	C30	C34	1.404(10)
N8	C2	1.372(5)	O1	C35	1.314(17)
N8	C18	1.389(6)			

Supplementary Table 15 Bond Angles for (CNC-^{i}Pr)$_2$Co(III)Br$_3$

Atom	Atom	Atom	Angle/°	Atom	Atom	Atom	Angle/°
N1	Co04	N10	177.98(18)	N3	C2	Co04	143.1(3)
N1	Co04	C2	100.80(16)	N3	C2	N8	104.5(4)
N1	Co04	C3	80.47(16)	N8	C2	Co04	112.1(3)
N1	Co04	C4	80.38(16)	N4	C3	Co04	111.8(3)
N1	Co04	C6	98.69(16)	N6	C3	Co04	144.2(3)
N10	Co04	C2	80.19(17)	N6	C3	N4	103.6(4)
N10	Co04	C3	101.25(19)	N2	C4	Co04	111.2(3)
N10	Co04	C4	97.89(19)	N7	C4	Co04	144.5(3)
N10	Co04	C6	80.34(17)	N7	C4	N2	104.1(4)
C2	Co04	C3	94.08(18)	N1	C5	N4	110.1(3)
C2	Co04	C4	89.88(18)	N1	C5	C9	121.8(4)
C2	Co04	C6	160.52(18)	C9	C5	N4	128.1(4)
C3	Co04	C4	160.85(17)	N5	C6	Co04	143.2(3)
C6	Co04	C3	89.30(18)	N5	C6	N9	104.7(4)
C6	Co04	C4	93.19(18)	N9	C6	Co04	111.7(3)
C1	N1	Co04	120.1(3)	C18	C7	N3	107.9(4)
C1	N1	C5	119.7(3)	C11	C8	N2	105.4(4)
C5	N1	Co04	120.1(3)	C16	C9	C5	117.4(4)
C1	N2	C8	130.6(4)	C1	C10	C16	115.8(4)
C4	N2	C1	117.9(4)	C8	C11	N7	108.2(4)
C4	N2	C8	111.4(4)	C19	C12	N5	108.3(4)
C2	N3	C7	110.4(4)	N3	C13	C22	109.6(4)
C2	N3	C13	126.0(4)	N3	C13	C28	110.1(4)
C7	N3	C13	123.5(4)	C28	C13	C22	112.6(5)
C3	N4	C5	117.5(4)	C15	C14	N4	105.2(4)
C3	N4	C14	111.9(4)	C14	C15	N6	108.2(4)
C14	N4	C5	130.4(4)	C9	C16	C10	122.6(4)
C6	N5	C12	110.4(4)	N5	C17	C27	110.6(4)
C6	N5	C17	125.7(4)	N5	C17	C32	109.8(5)
C12	N5	C17	123.9(4)	C32	C17	C27	109.9(5)
C3	N6	C15	111.0(4)	C7	C18	N8	105.7(4)
C3	N6	C21	125.4(4)	C12	C19	N9	104.6(4)
C15	N6	C21	123.6(4)	N7	C20	C25	111.0(4)
C4	N7	C11	110.9(4)	N7	C20	C33	110.7(5)
C4	N7	C20	126.4(4)	C33	C20	C25	111.0(5)
C11	N7	C20	122.7(4)	N6	C21	C23	109.3(5)
C2	N8	C18	111.4(4)	N6	C21	C31	109.8(5)
C2	N8	C26	117.7(4)	C23	C21	C31	111.8(5)
C26	N8	C18	129.9(4)	N10	C24	N9	109.2(4)
C6	N9	C19	111.9(4)	N10	C24	C29	123.1(6)
C6	N9	C24	118.0(4)	C29	C24	N9	127.5(5)
C19	N9	C24	130.0(4)	N8	C26	C30	127.1(6)
C24	N10	Co04	119.8(3)	N10	C26	N8	109.5(4)
C24	N10	C26	119.2(5)	N10	C26	C30	122.5(6)
C26	N10	Co04	119.9(3)	C34	C29	C24	116.6(6)
N1	C1	N2	110.3(3)	C26	C30	C34	116.3(6)
N1	C1	C10	122.6(4)	C29	C34	C30	122.0(6)
C10	C1	N2	127.0(4)				

Method B: In a nitrogen atmosphere, a vail was charged with $Co(acac)_2$ (4.37 mg, 0.02 mmol), CNC-^{i}Pr (10.2 mg, 0.022 mmol), ^{t}BuOK (10.1 mg, 0.09 mmol) dissolved in dry DMF. Single-crystals of (CNC-^{i}Pr)$_2$Co(III)Br$_3$ suitable for X-ray diffraction were obtained by slow vapor diffusion of toluene into the DMF solution. HRMS of (CNC-^{i}Pr)$_2$Co(III) was also collected as shown in Supplementary Figure 15. The collected (CNC-^{i}Pr)$_2$Co(III)Br$_3$ crystals, totaling less than 0.5 mg, were utilized for catalyzing the transformation of **1a**, showing promising catalytic activity similar to the model reaction (Supplementary Figure 16).

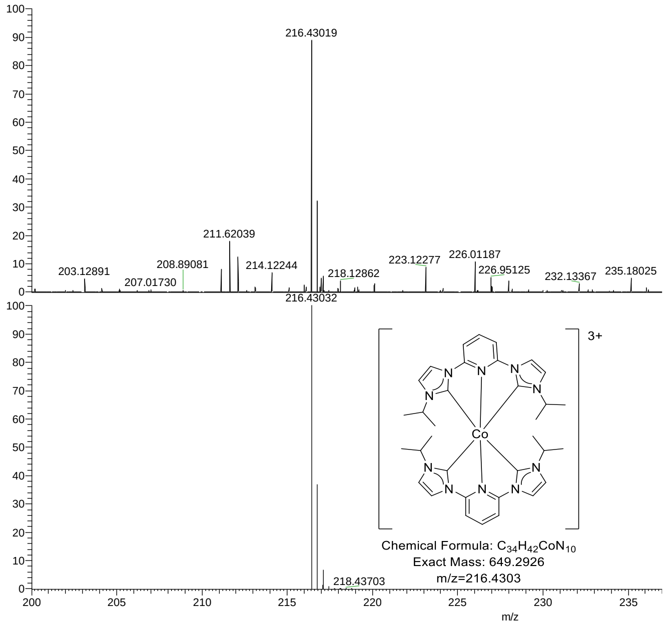

Supplementary Figure 15 HRMS of (CNC-^{i}Pr)$_2$Co(III)

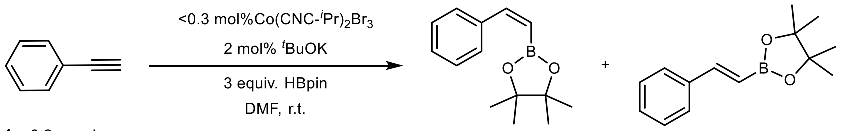

1a, 0.2 mmol

Z-**1b** *E*-**1b**

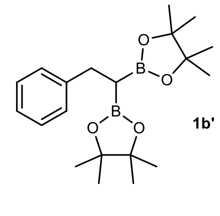

1b'

t	*Z*-**1b**: *E*-**1b**[a]	Yield (%)[b]
30 min	95:5	>99
24 h	0:100	80[c]

[a]*Z/E* ratio based on GC-Ms analysis. [b]GC-Ms yield using mesitylene as internal standard. [c]20% of the byproduct **1b'** was observed.

Supplementary Figure 16 Catalytic performance of (CNC-^{i}Pr)$_2$CoBr$_3$

6.9 HRMS of in situ formed (CNC-^{i}Pr)Co(I)H complex:

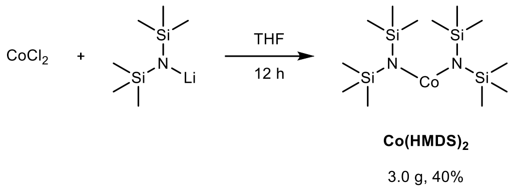

Preparation of Co(HMDS)$_2$: The title compound was synthesized according to a reported procedure with modifications[23]. LiHMDS (1 M in THF, 40 mmol) was added to a vigorously stirred suspension of $CoCl_2$ (2.6 g, 20 mmol) in THF (40 mL) while being cooled in an ice bath. After 1 hour, the ice bath was removed and stirring was continued for an additional 12 hours, resulting in the formation of a green solution. The THF was then evaporated under reduced pressure, and the remaining residue was extracted with pentane (50 mL) and subsequently filtered. The pentane was evaporated under reduced pressure, leaving behind a green, oily solid that was further purified by sublimation at 70 ℃ under reduced pressure. This purification step yielded bright green crystals of the product, with a total weight of 3.0 g and a yield of 40%.

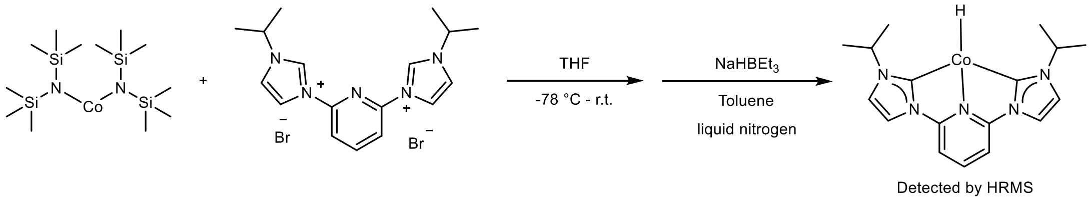

Co(HMDS)$_2$ (986 mg, 2.6 mmol) and CNC-^{i}Pr (1.2 g, 2.26 mmol) were combined in a Schlenk tube. The tube was then placed in a -78℃ environment, and precooled THF (100 mL) was slowly added via syringe. As the suspension gradually warmed up, it transitioned from red-orange to eventually forming a green solution at room temperature. The reaction mixture was stirred at room temperature for 8 hours, during which dark green precipitates began to form. After removing the volatile components under vacuum, a green residue remained and was washed with ether and subsequently dried under vacuum.

Next, a 20 mL Schlenk tube was filled with a solution of 0.154 g of the green residue in approximately 3 mL of toluene. The mixture was cooled in a liquid nitrogen-cooled cold well for approximately 5 minutes, and with vigorous stirring, 315 μL (0.315 mmol) of a 1 M solution of $NaHBEt_3$ in THF were added dropwise. The mixture was then stirred at room temperature for 12 hours before being filtered. The residue was washed twice with toluene and three times with pentane (in 5 mL portions each time), resulting in the acquisition of 58 mg of a dark green powder. This powder was then subjected to HRMS testing, leading to the detection of (CNC-^{i}Pr)Co(I)H (as shown in Figure S17).

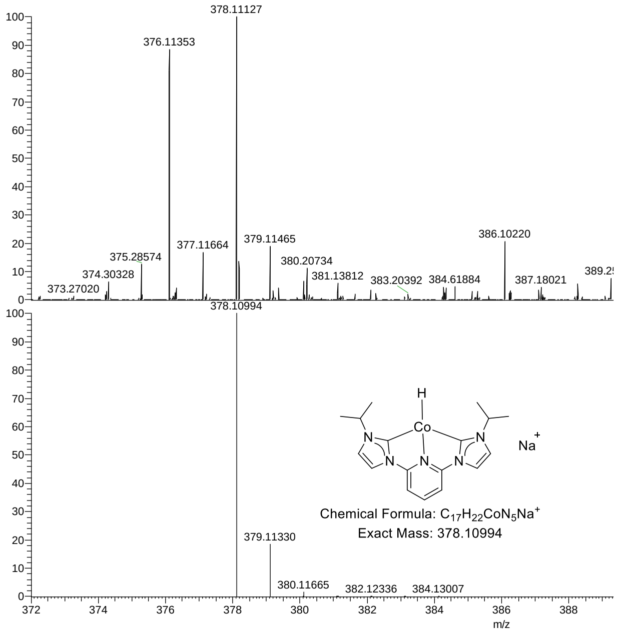

Supplementary Figure 17 HRMS of (CNC-^{i}Pr)$_2$Co(I)H

6.10 Detection of H_2 by gas chromatography

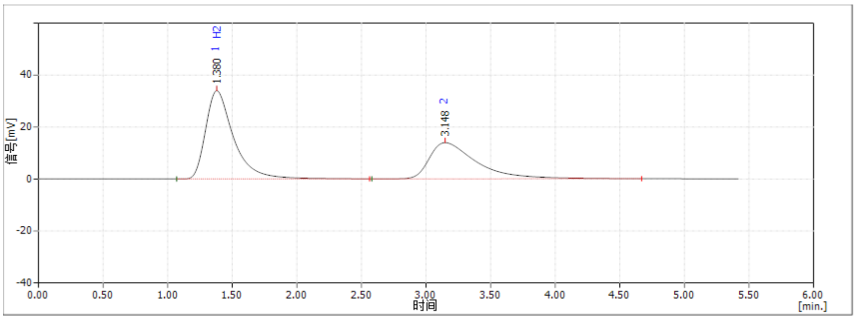

Supplementary Figure 18 GC of the gas from the headspace of the model reaction

7. NMR spectra

^{1}H NMR (600 MHz, DMSO-*d6*) of **CNC-^{i}Pr** (*See procedure*)

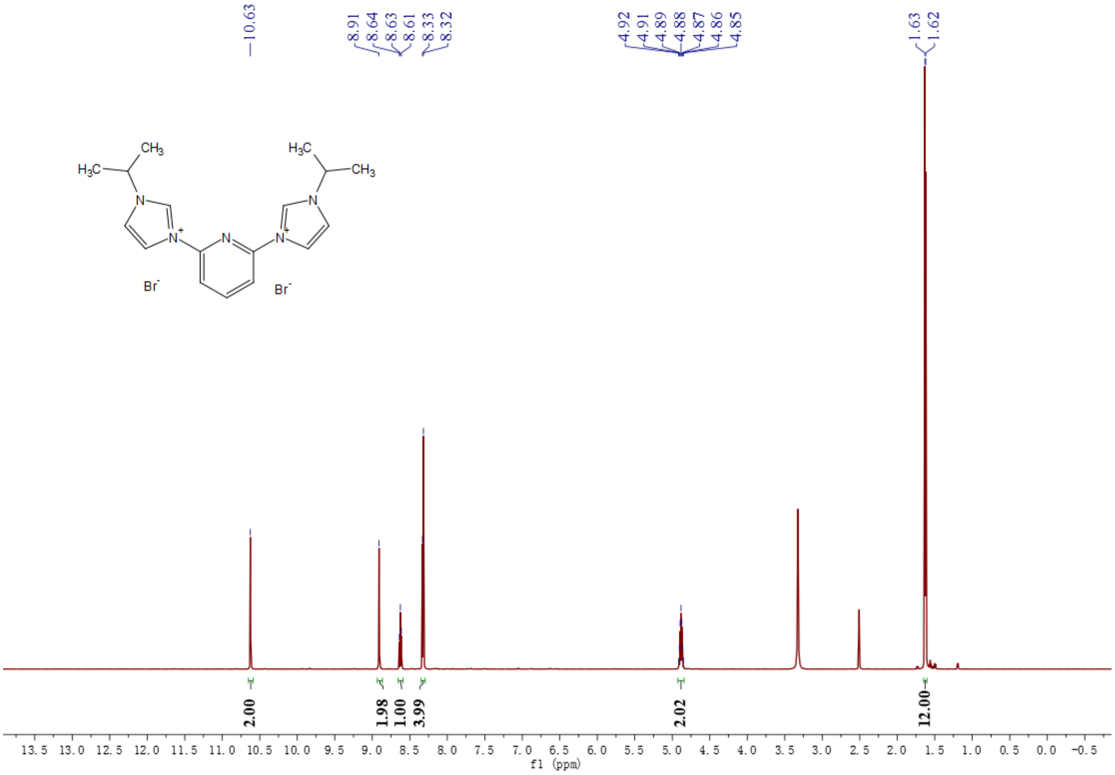

^{1}H NMR(400 MHz, Chloroform-*d*) of **CNC-H** (*See procedure*)

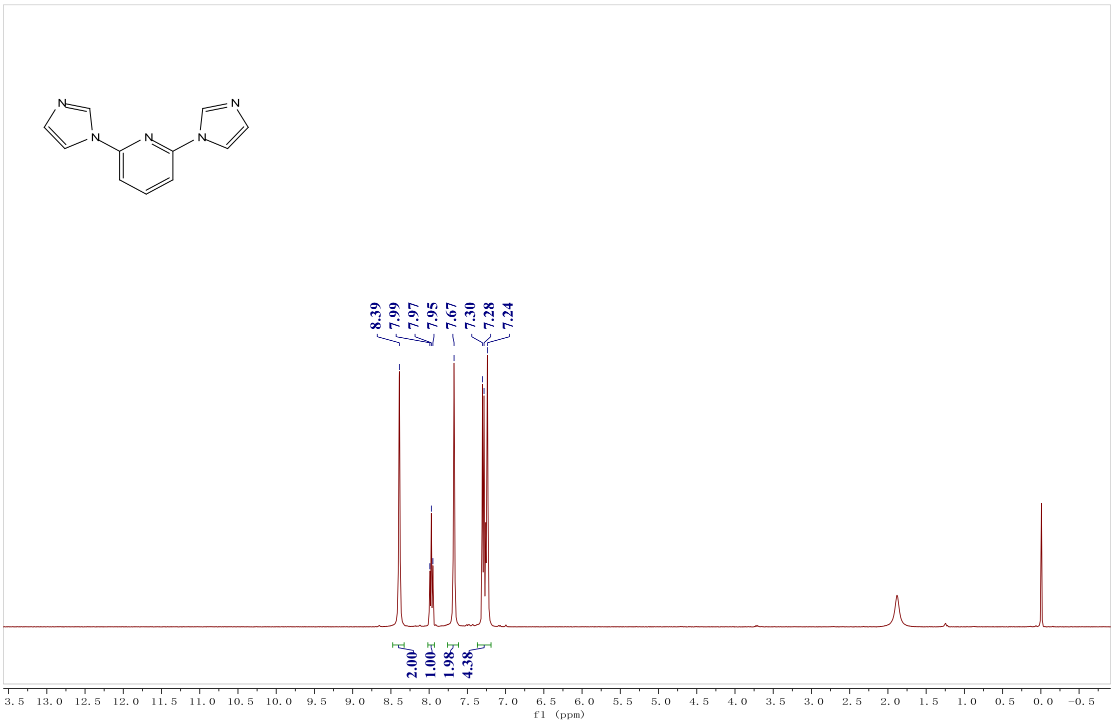

^{1}H NMR (400 MHz, DMSO-*d6*) of **CNC-Me** (*See procedure*)

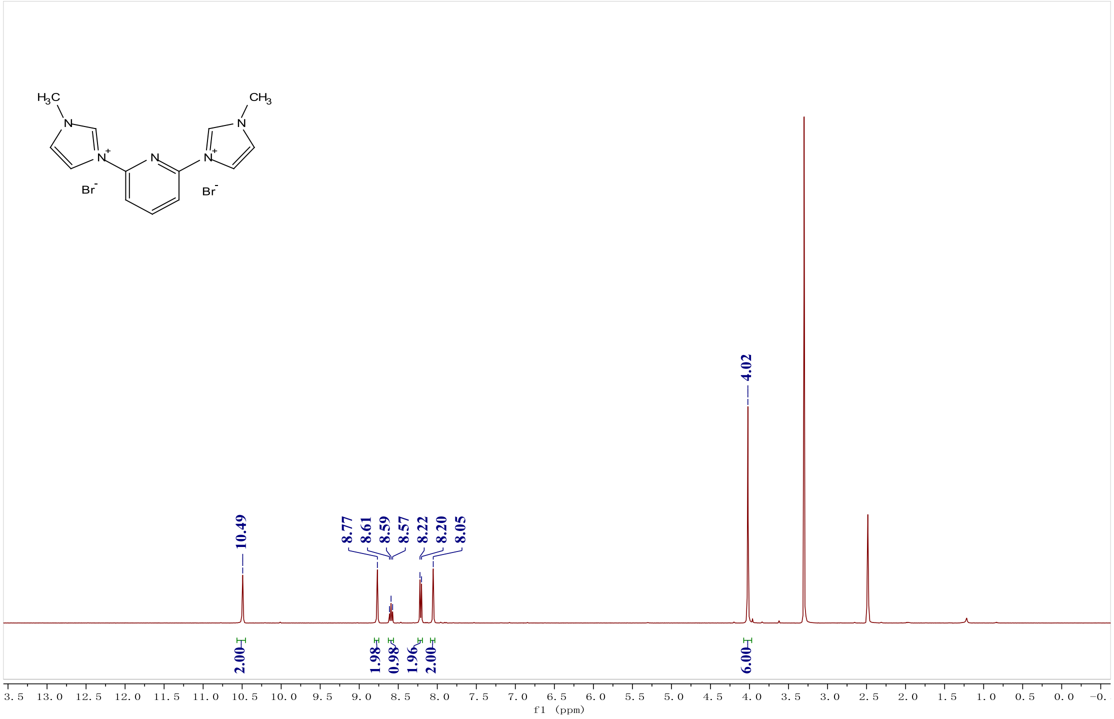

^{1}H NMR (400 MHz, DMSO-*d6*) of **CNC-Et** (*See procedure*)

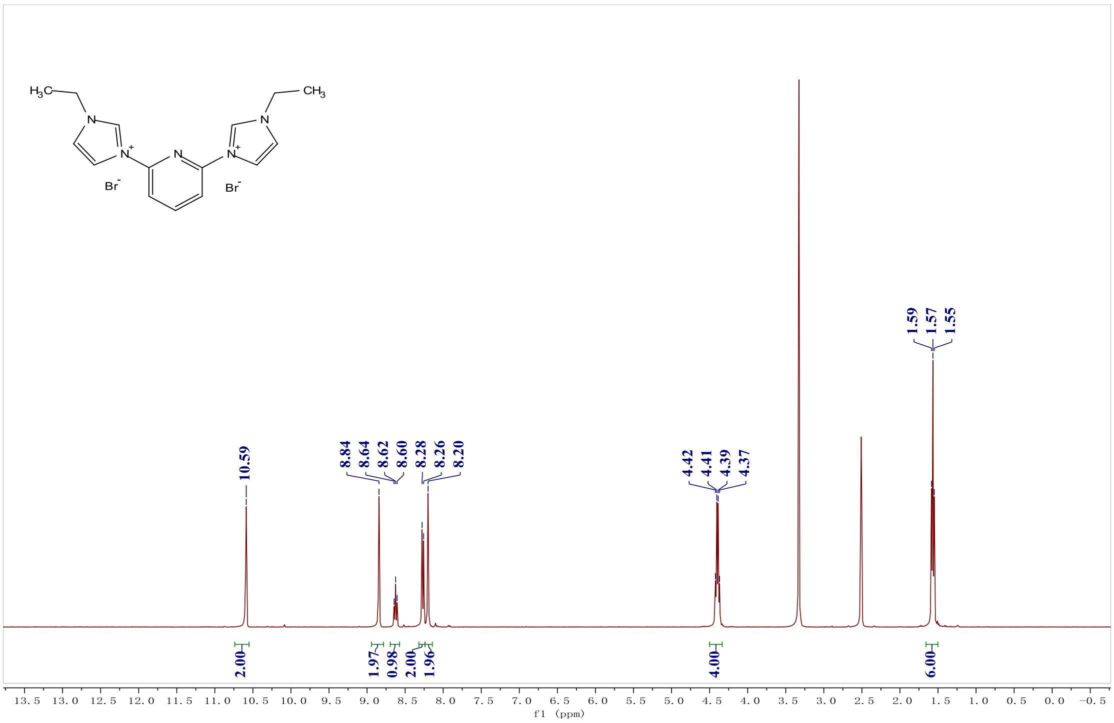

^{1}H NMR (400 MHz, DMSO-*d6*) of **CNC-*ᵗ*Bu** (*See procedure*)

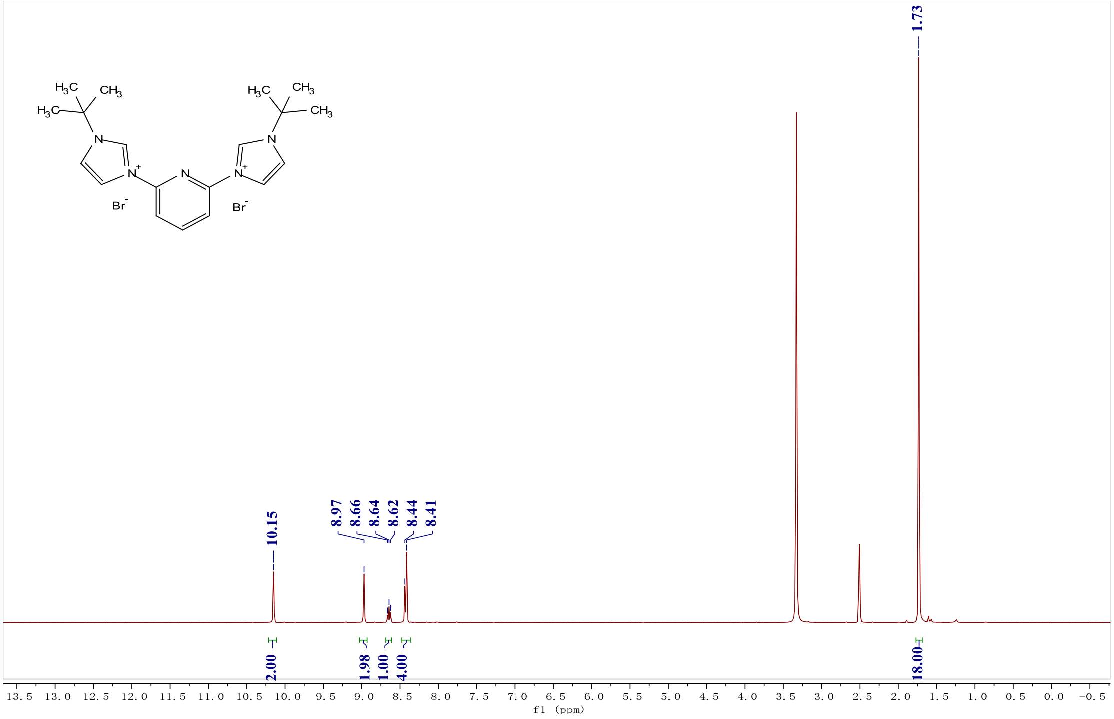

^{1}H NMR (400 MHz, Chloroform-*d*) of **CNC-Mes** (*See procedure*)

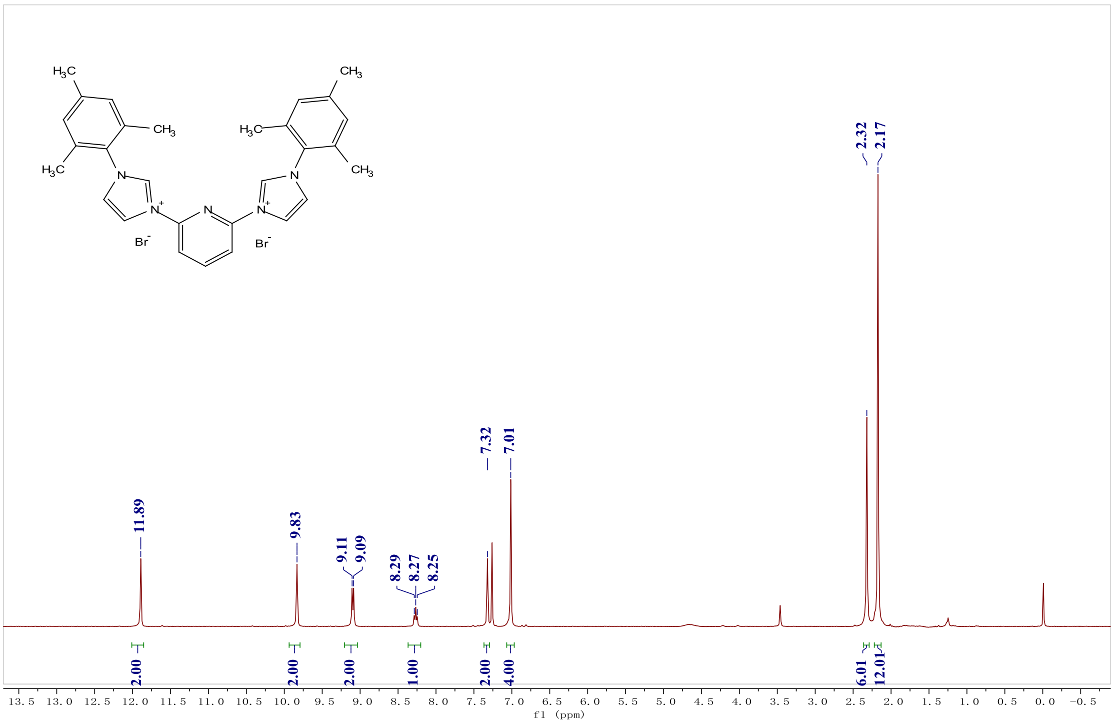

^{1}H NMR (400 MHz, Chloroform-*d*) of **5a** (*See procedure*)

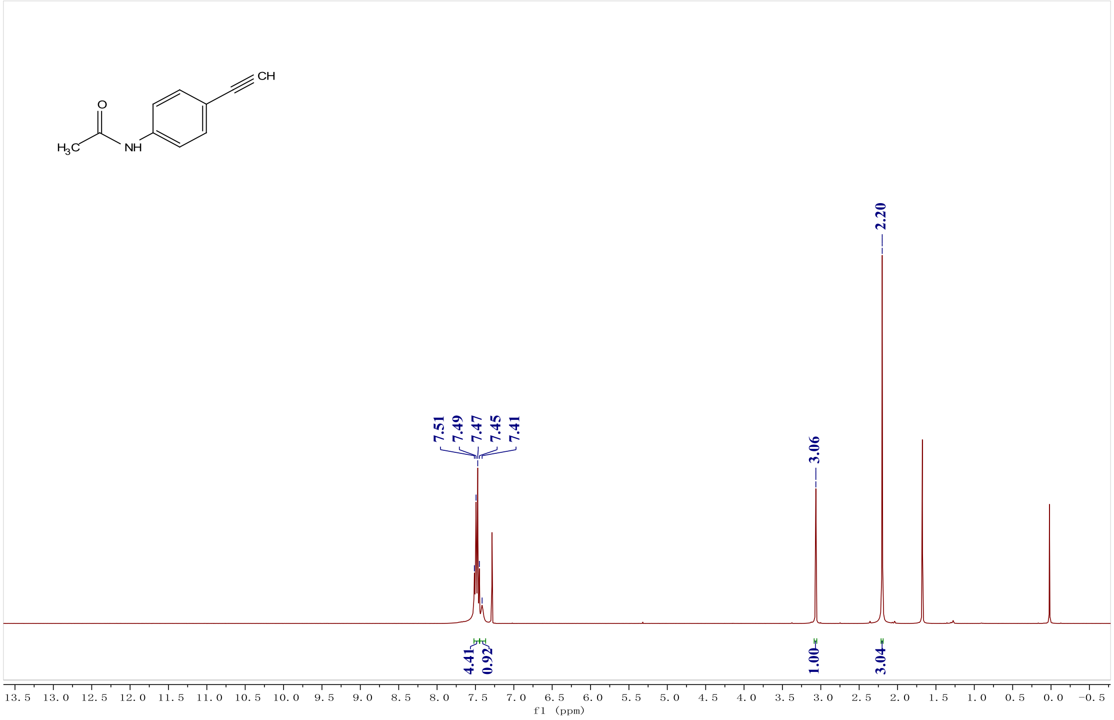

^{1}H NMR (600 MHz, Chloroform-*d*) of **6a** (*See procedure*)

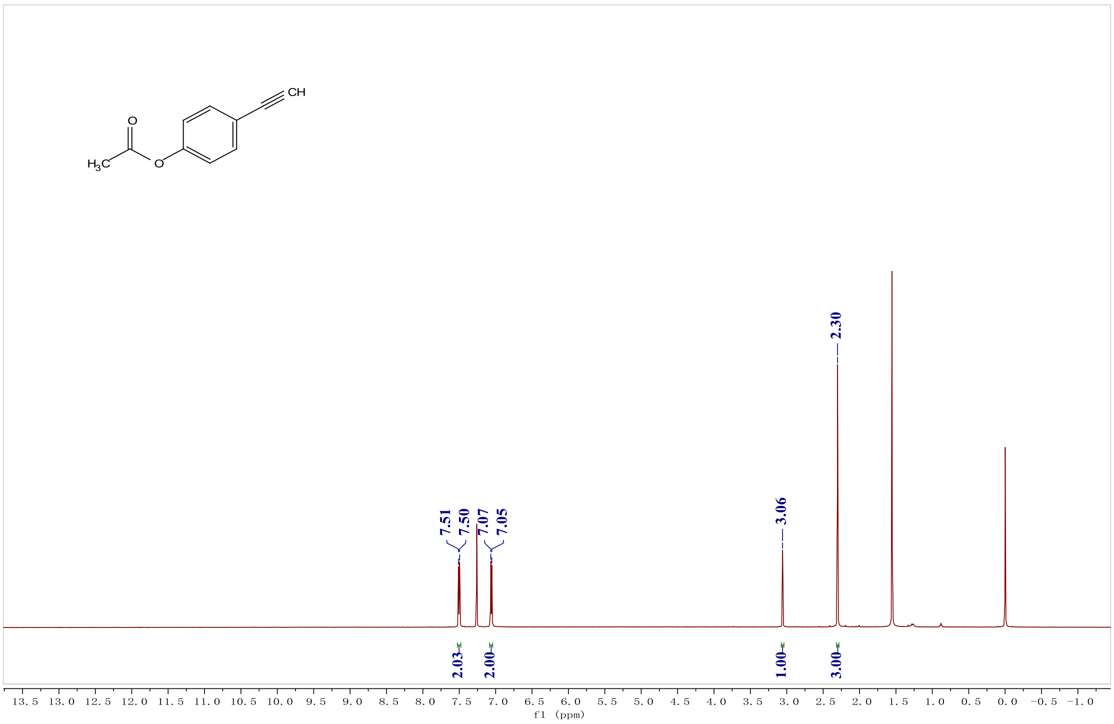

^{1}H NMR (400 MHz, Chloroform-*d*) of **16a** (*See procedure*)

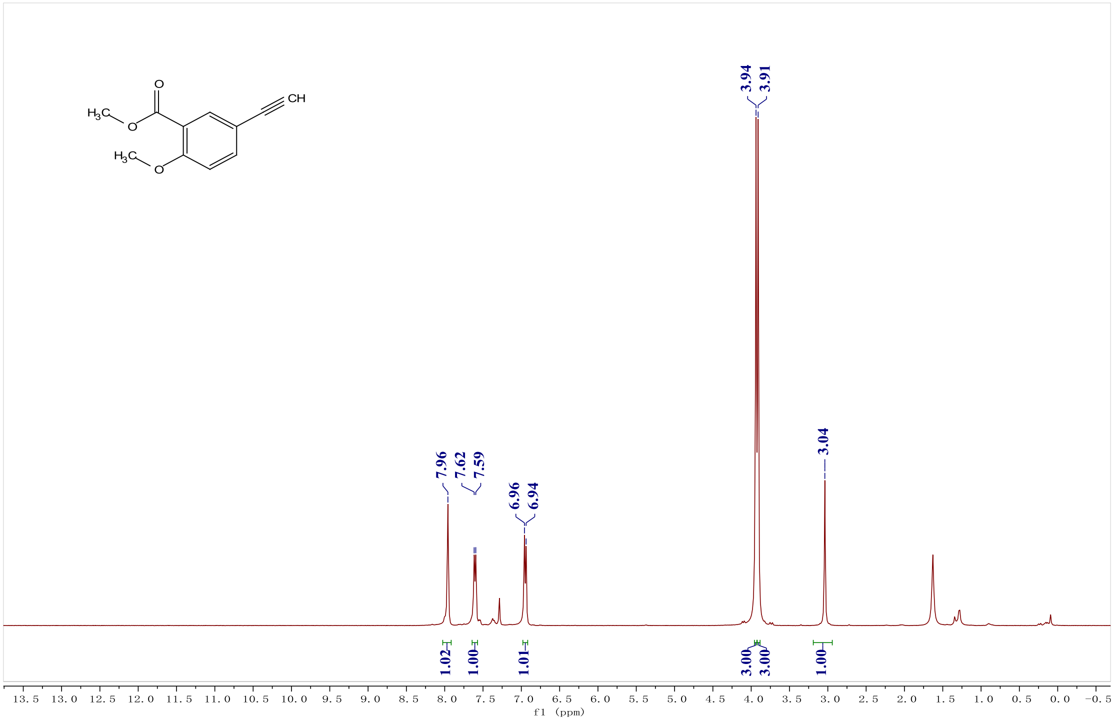

^{1}H NMR (400 MHz, Chloroform-*d*) of **22a** (See procedure)

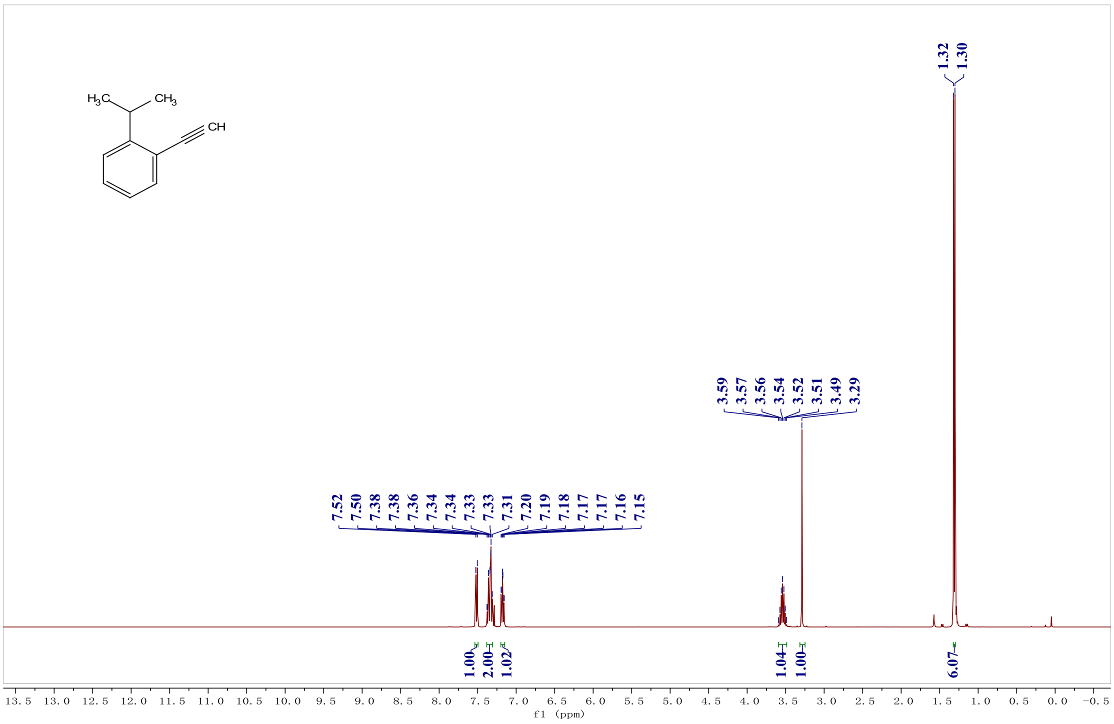

^{1}H NMR (600 MHz, Chloroform-*d*) of **23a** (See procedure)

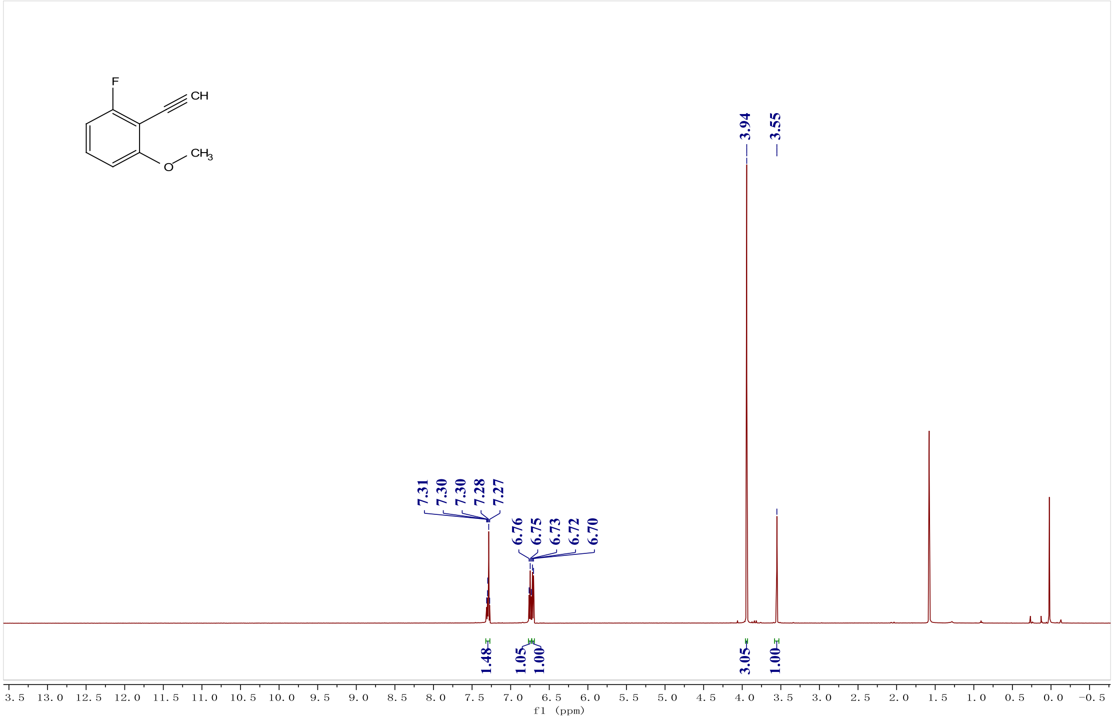

^{1}H NMR (400 MHz, Chloroform-*d*) of **24a** (*See procedure*)

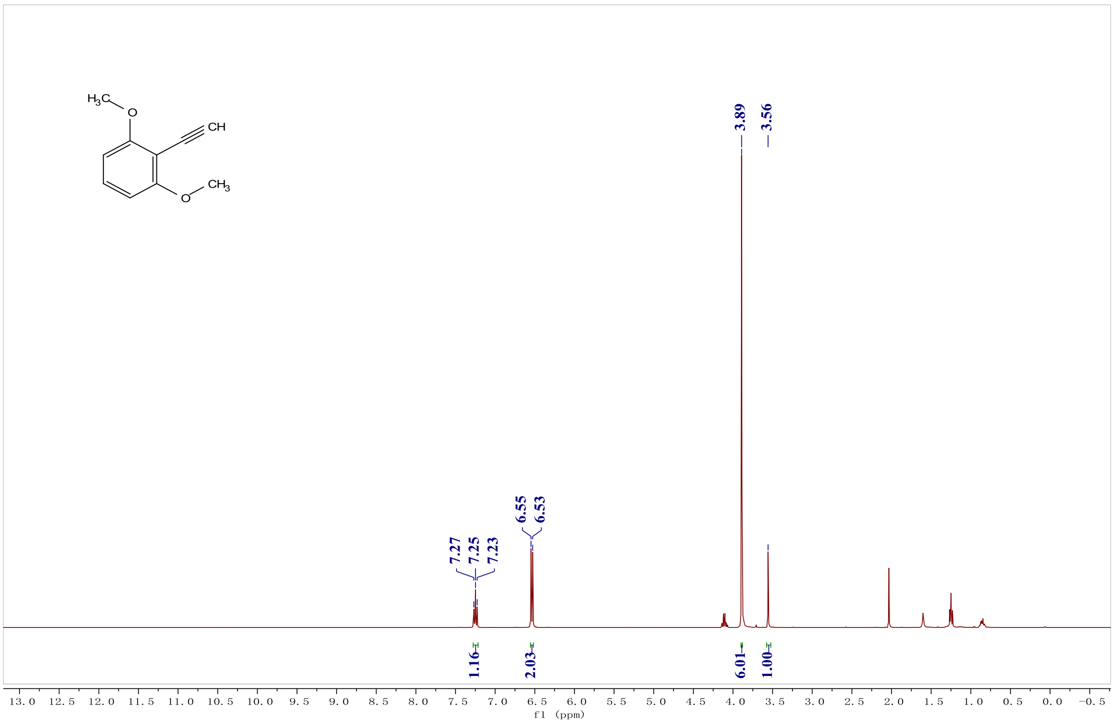

^{1}H NMR (400 MHz, Chloroform-*d*) of **26a** (*See procedure*)

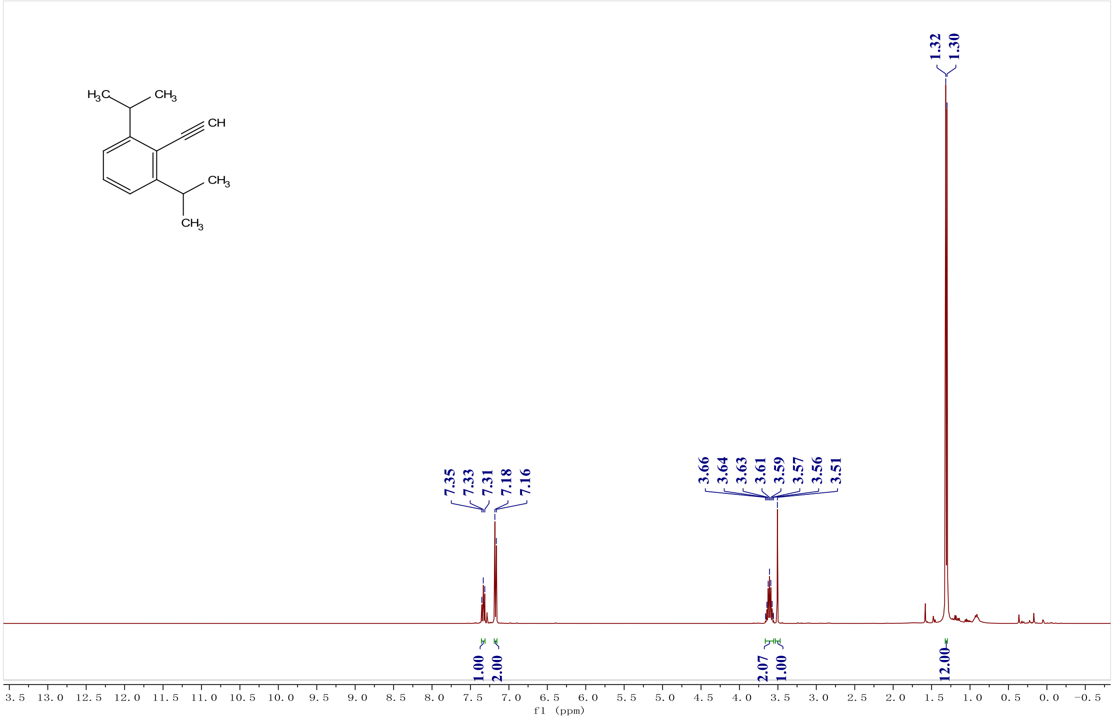

^{1}H NMR (400 MHz, Chloroform-*d*) of **41a** (See procedure)

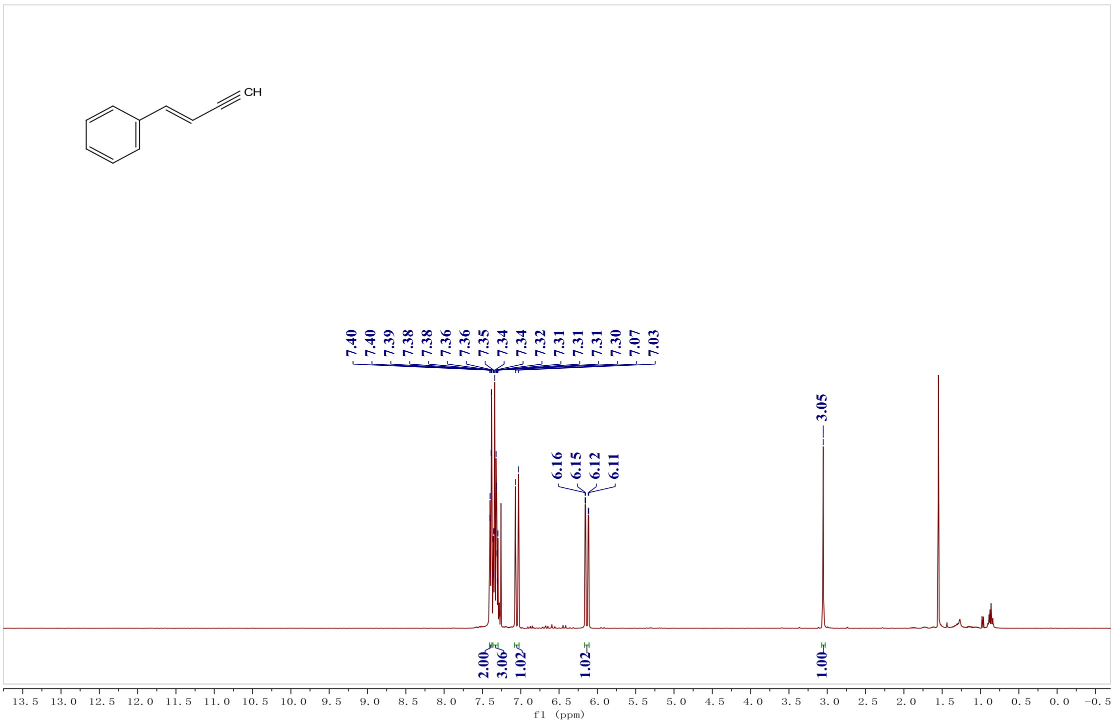

^{1}H NMR (400 MHz, Chloroform-*d*) of **1b** (See procedure)

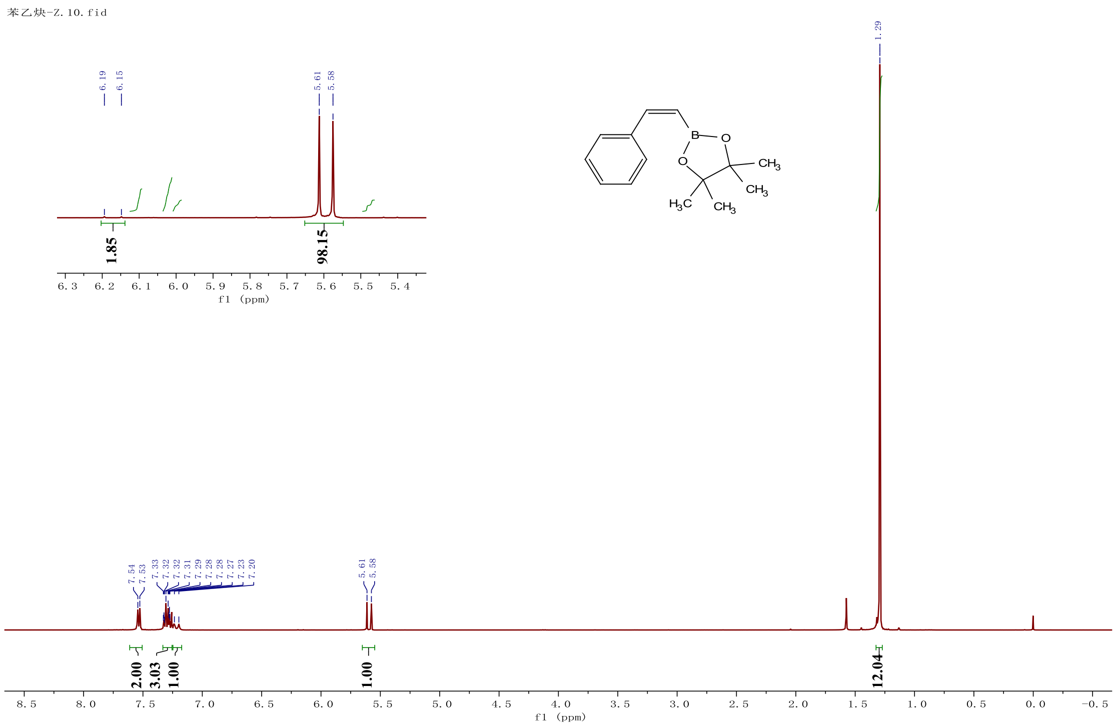

^{11}B NMR (128 MHz, Chloroform-*d*) of **1b** (*See procedure*)

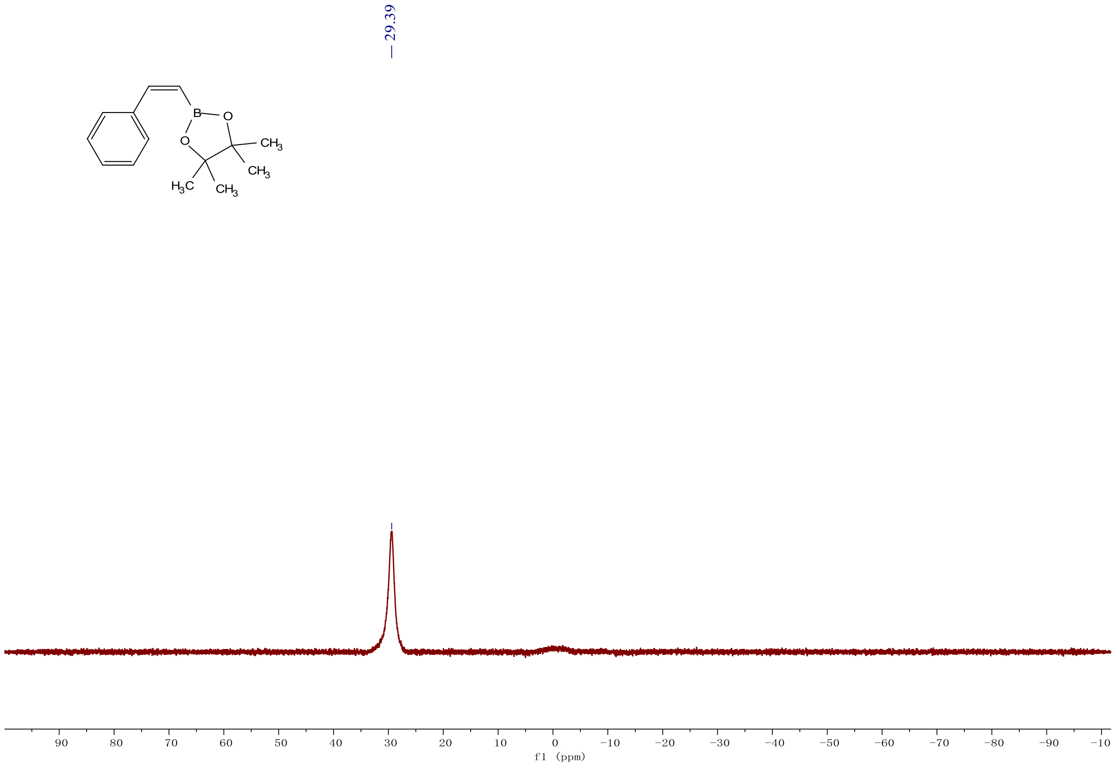

^{1}H NMR (400 MHz, Chloroform-*d*) of **2b** (See procedure)

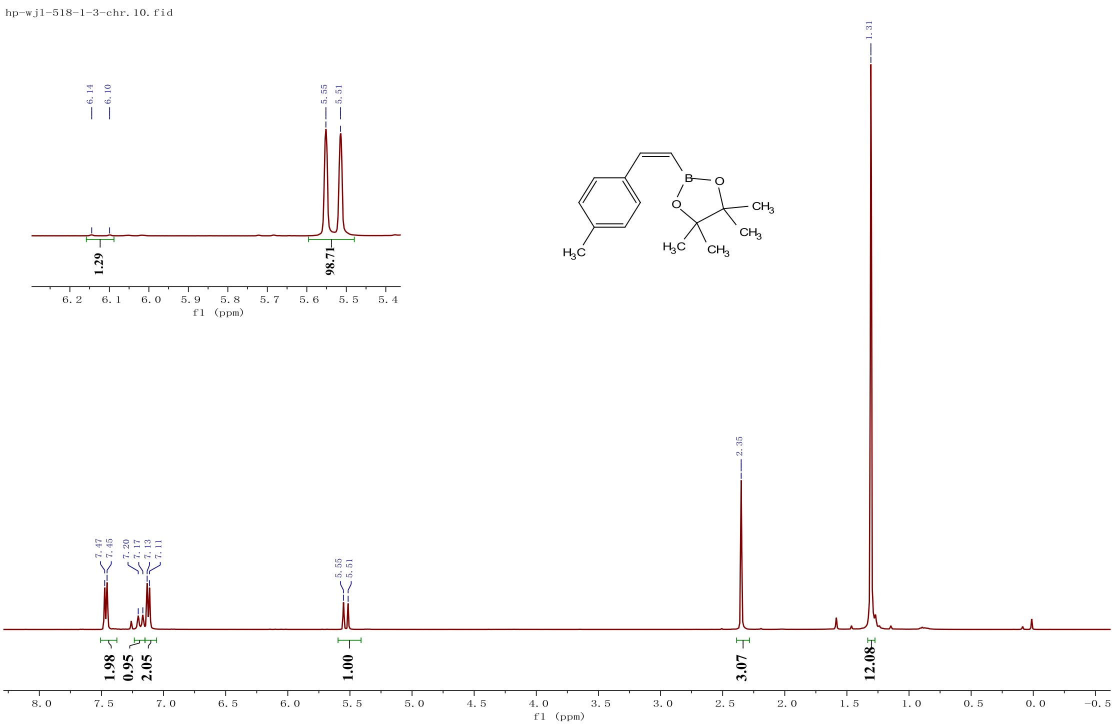

^{1}H NMR (400 MHz, Chloroform-*d*) of **3b** (See procedure)

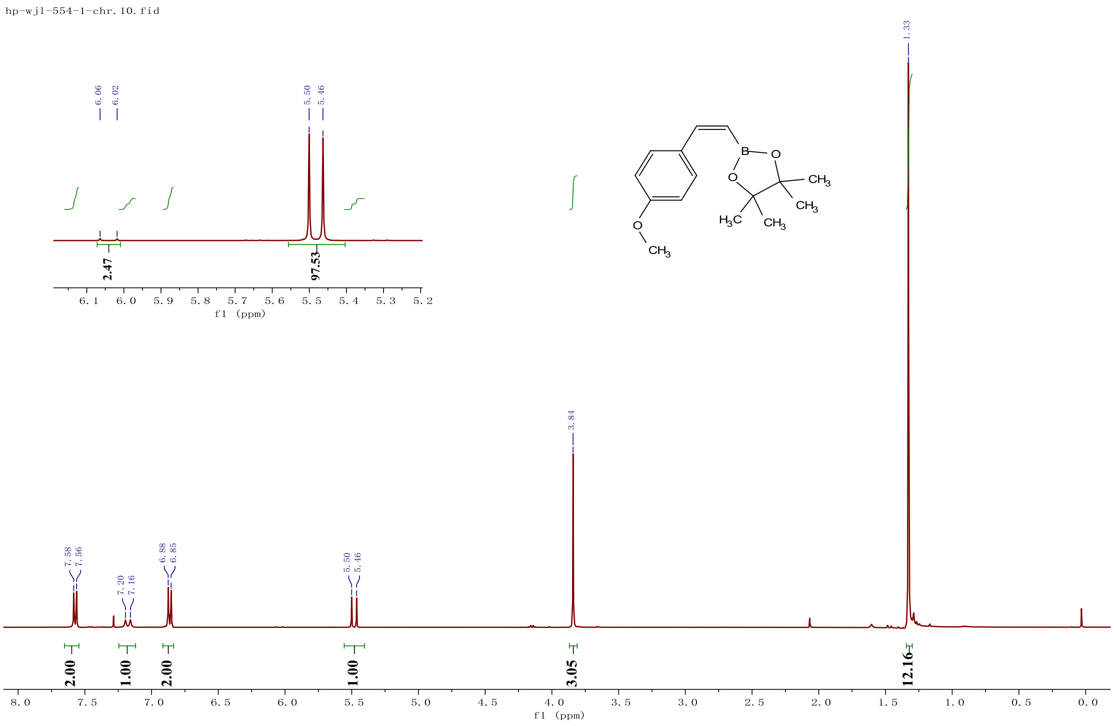

^{11}B NMR (193 MHz, Chloroform-*d*) of **3b** (*See procedure*)

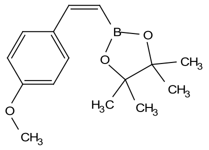

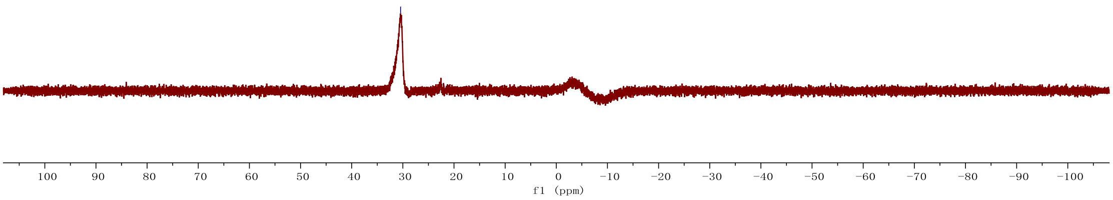

^{1}H NMR (400 MHz, Chloroform-*d*) of **4b** (*See procedure*)

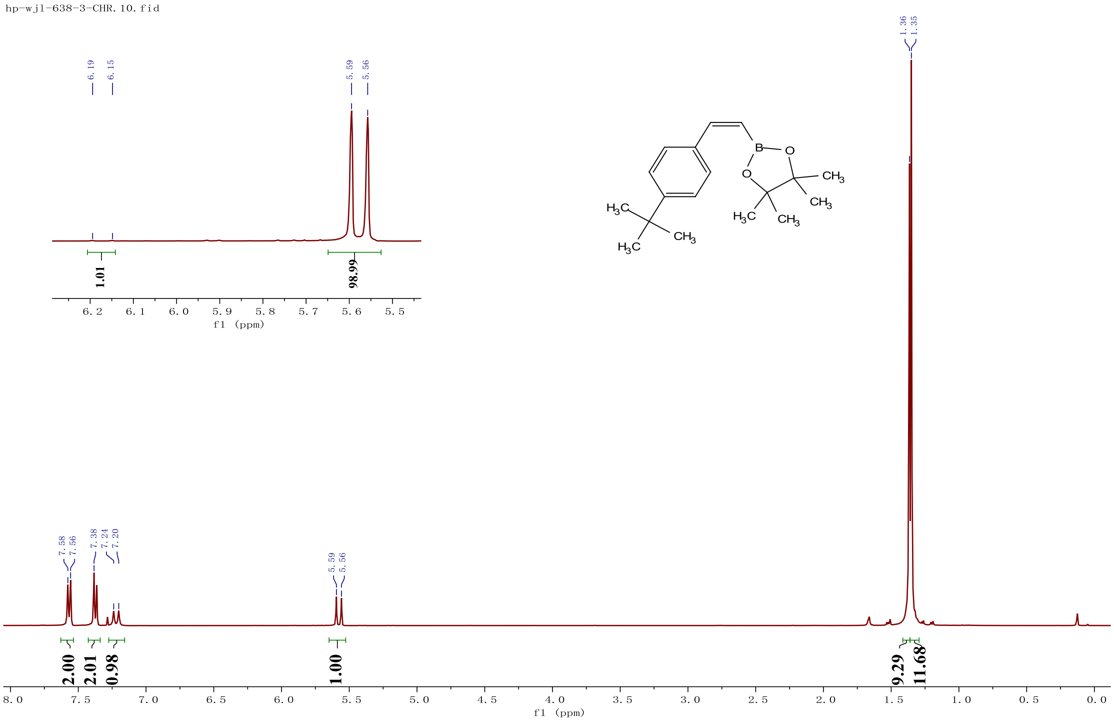

^{1}H NMR (400 MHz, Chloroform-*d*) of **5b** (*See procedure*)

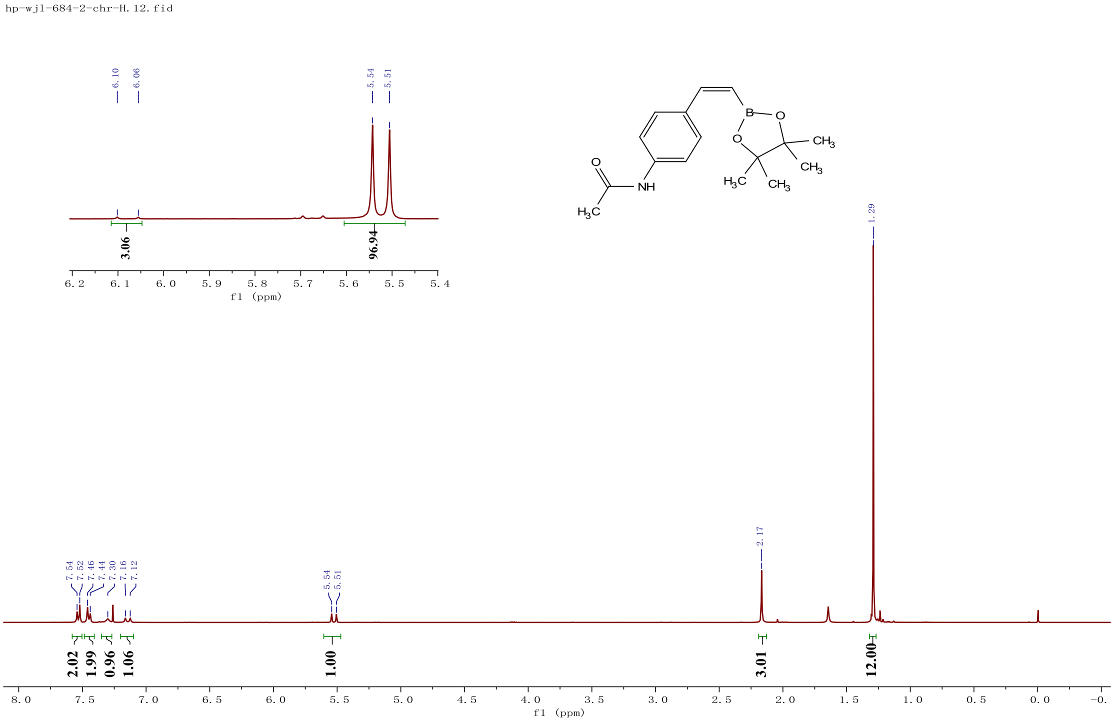

^{13}C NMR (101 MHz, Chloroform-*d*) of **5b** (*See procedure*)

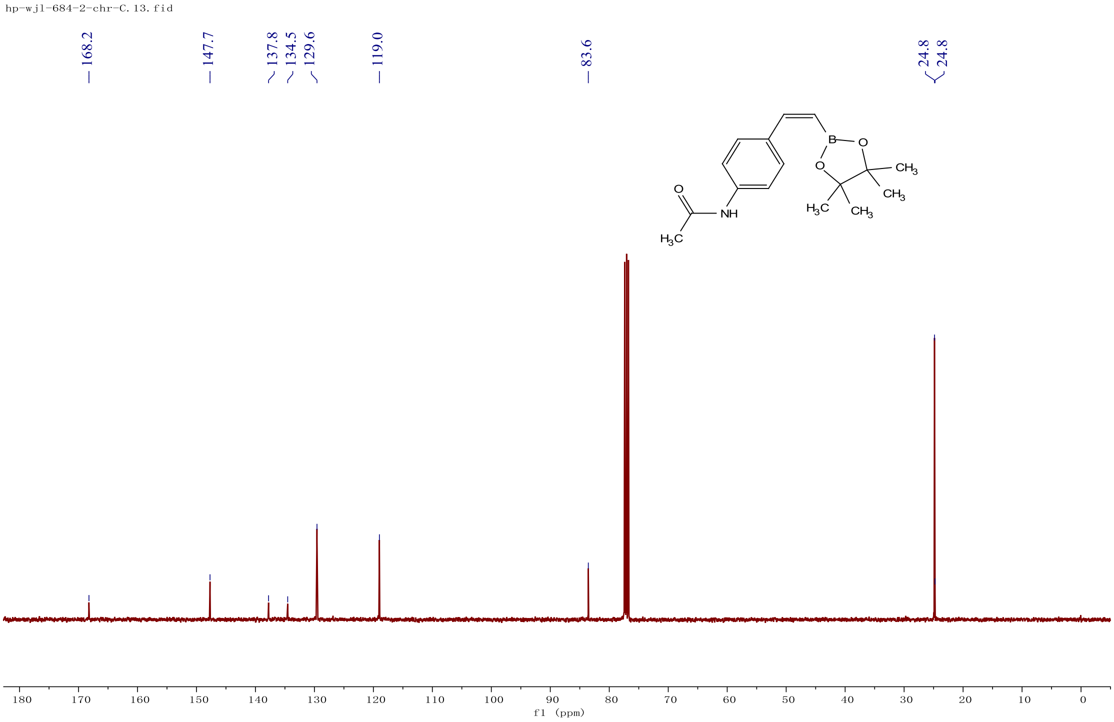

^{11}B NMR (193 MHz, Chloroform-*d*) of 5**b** (*See procedure*)

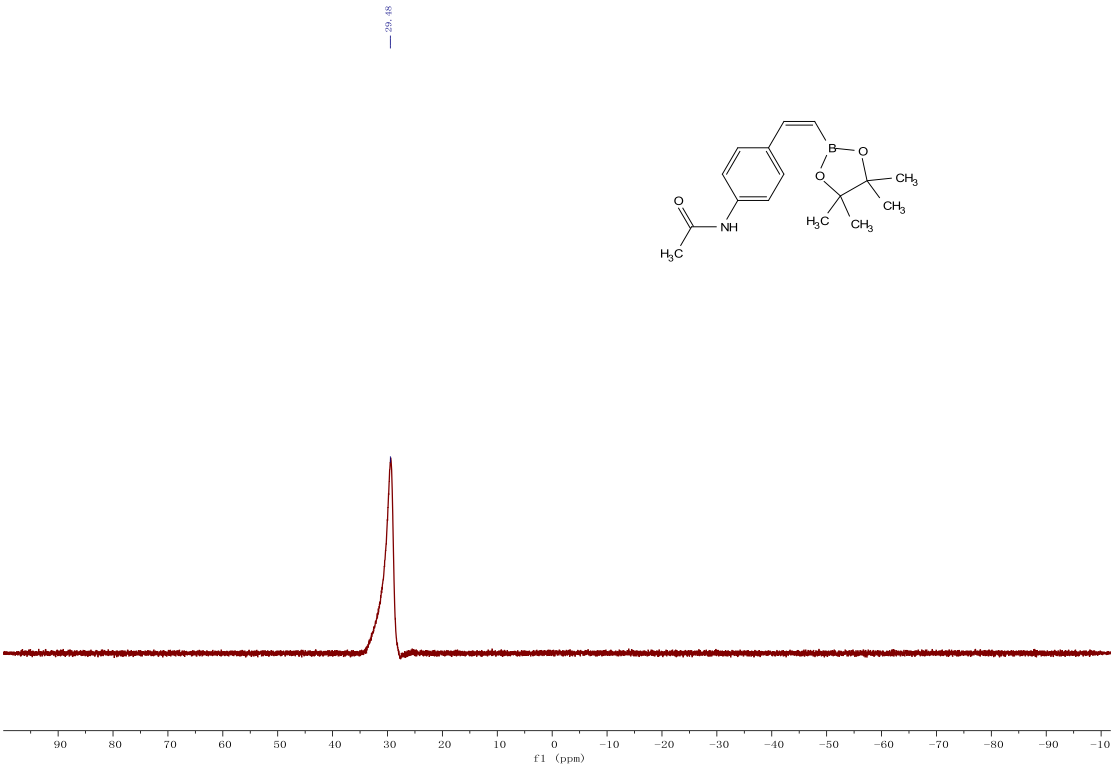

^{1}H NMR (400 MHz, Chloroform-*d*) of 6**b** (See procedure)

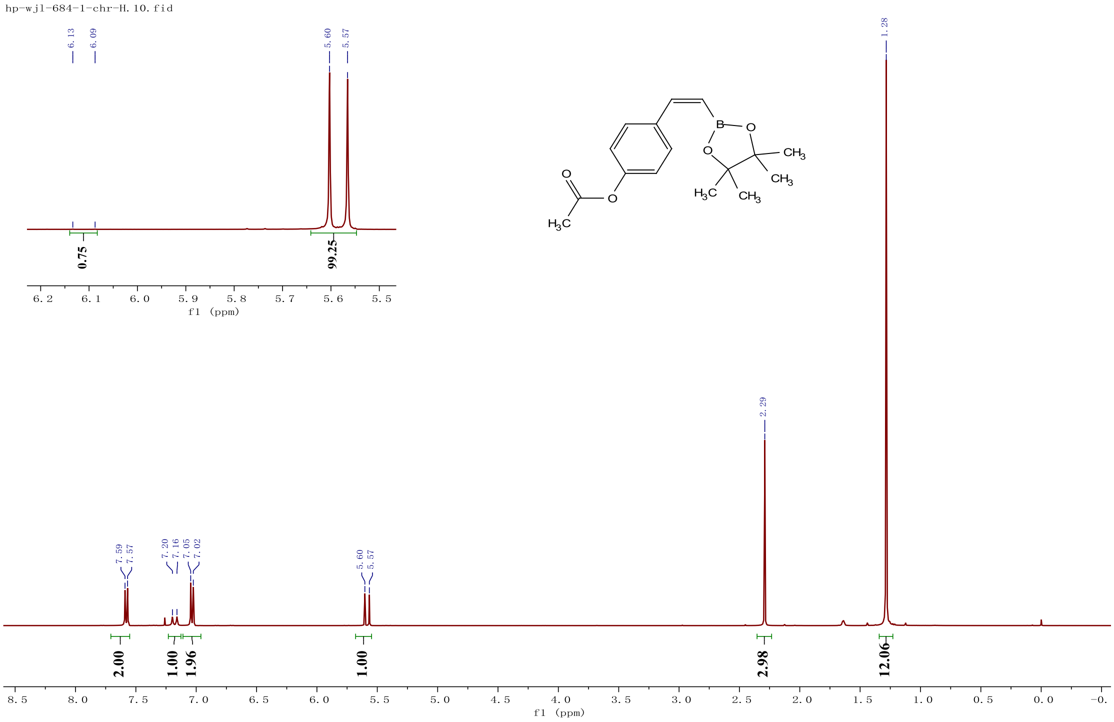

^{13}C NMR (101 MHz, Chloroform-*d*) of 6**b** (See procedure)

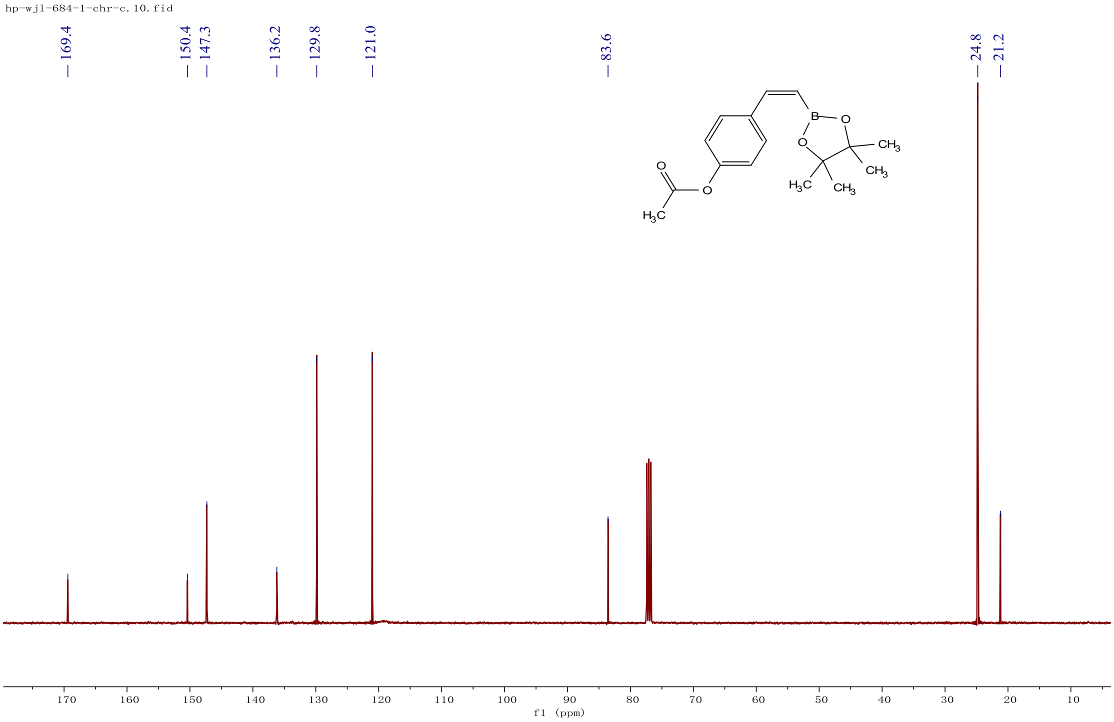

^{11}B NMR (193 MHz, Chloroform-d) of 6**b** (*See procedure*)

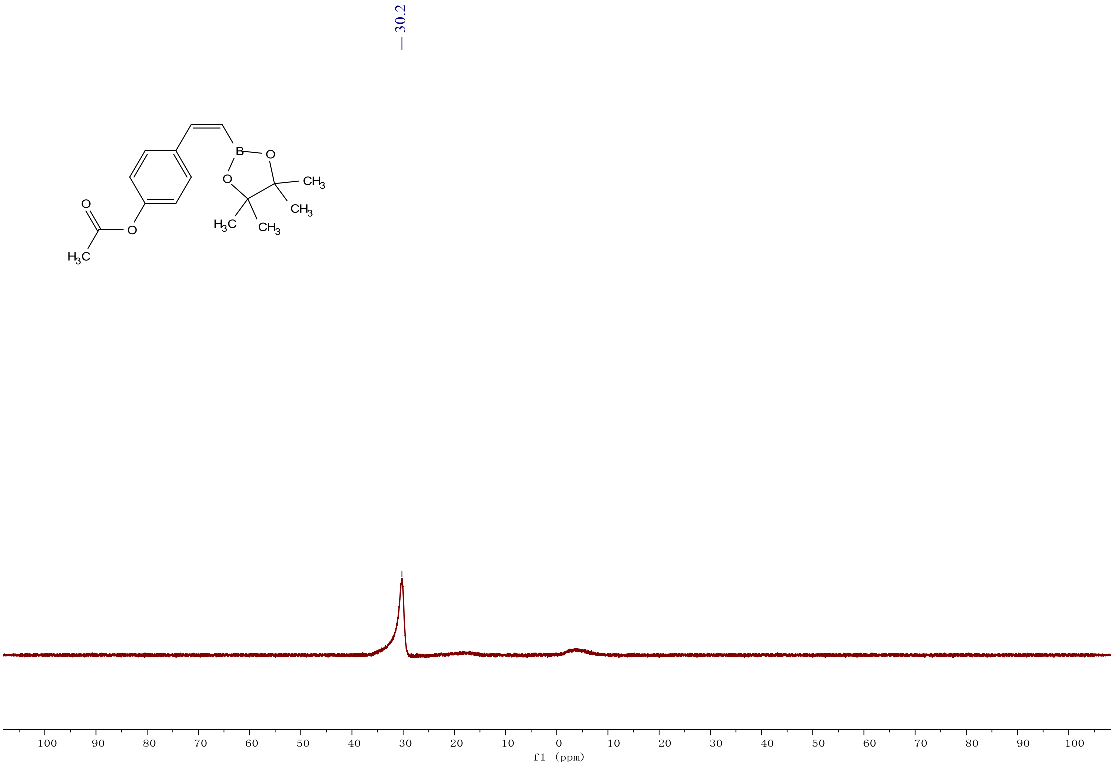

^{1}H NMR (600 MHz, Chloroform-d) of 7**b** (*See procedure*)

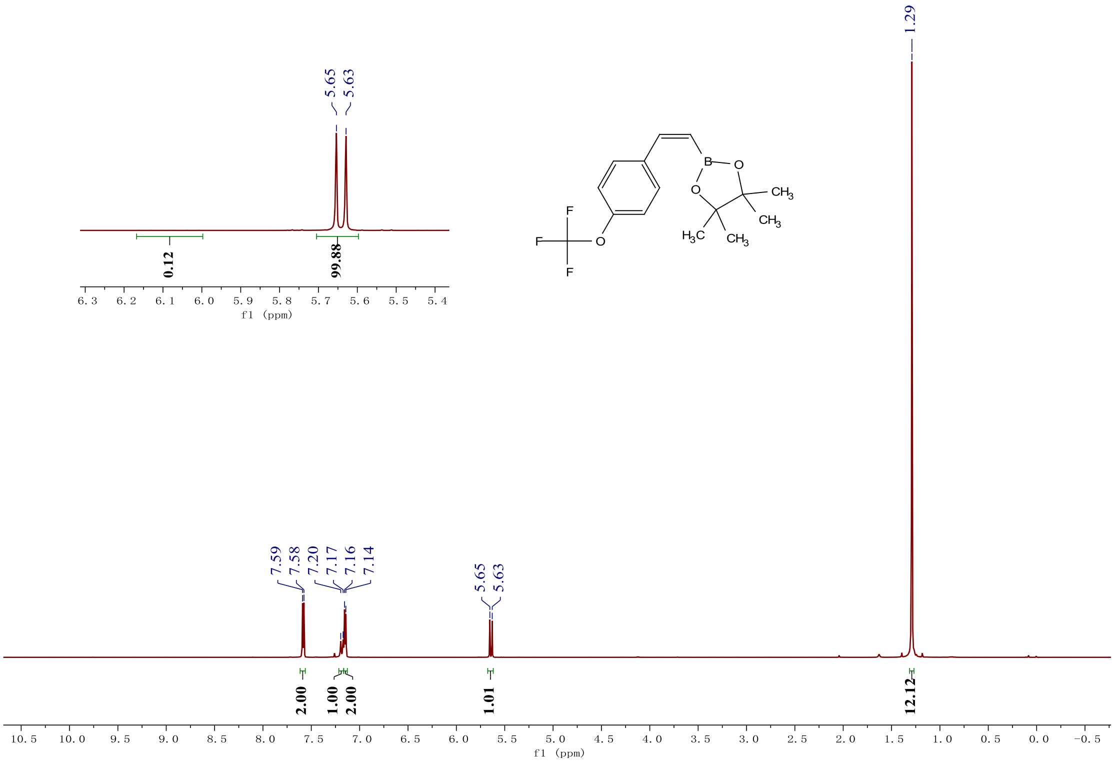

^{19}F NMR (565 MHz, **Chloroform-*d***) of **7b** (<u>*See procedure*</u>)

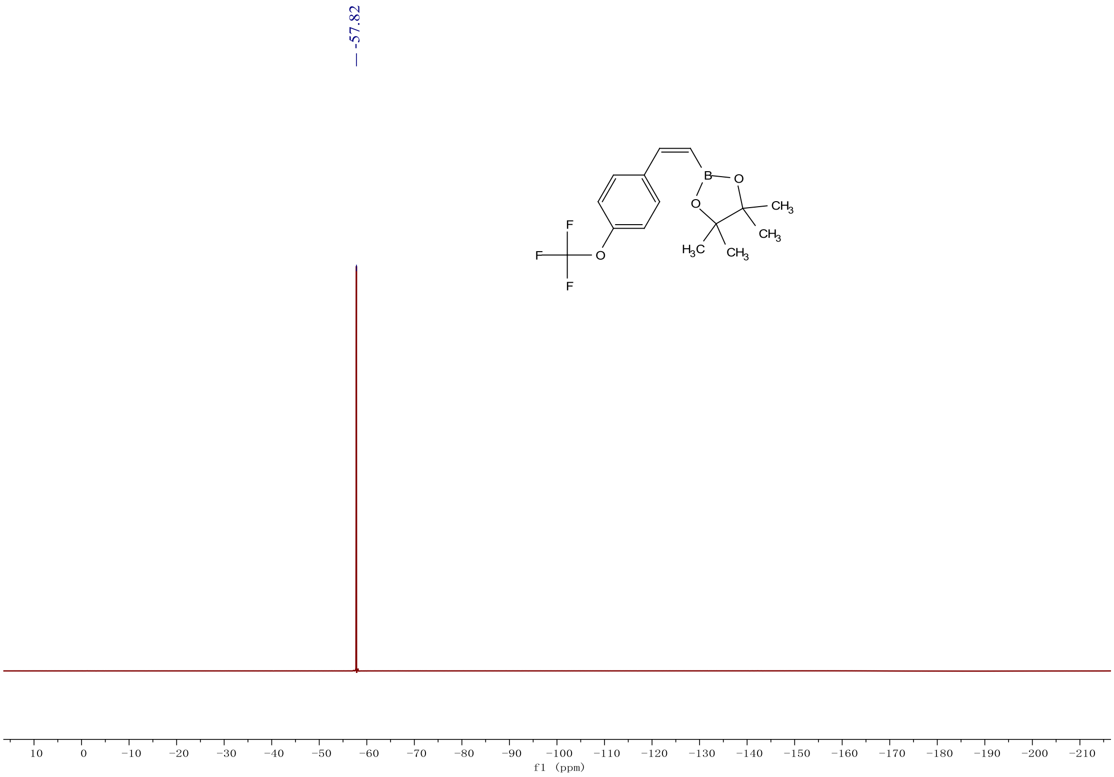

^{12}C NMR (151 MHz, **Chloroform-*d***) of **7b** (<u>*See procedure*</u>)

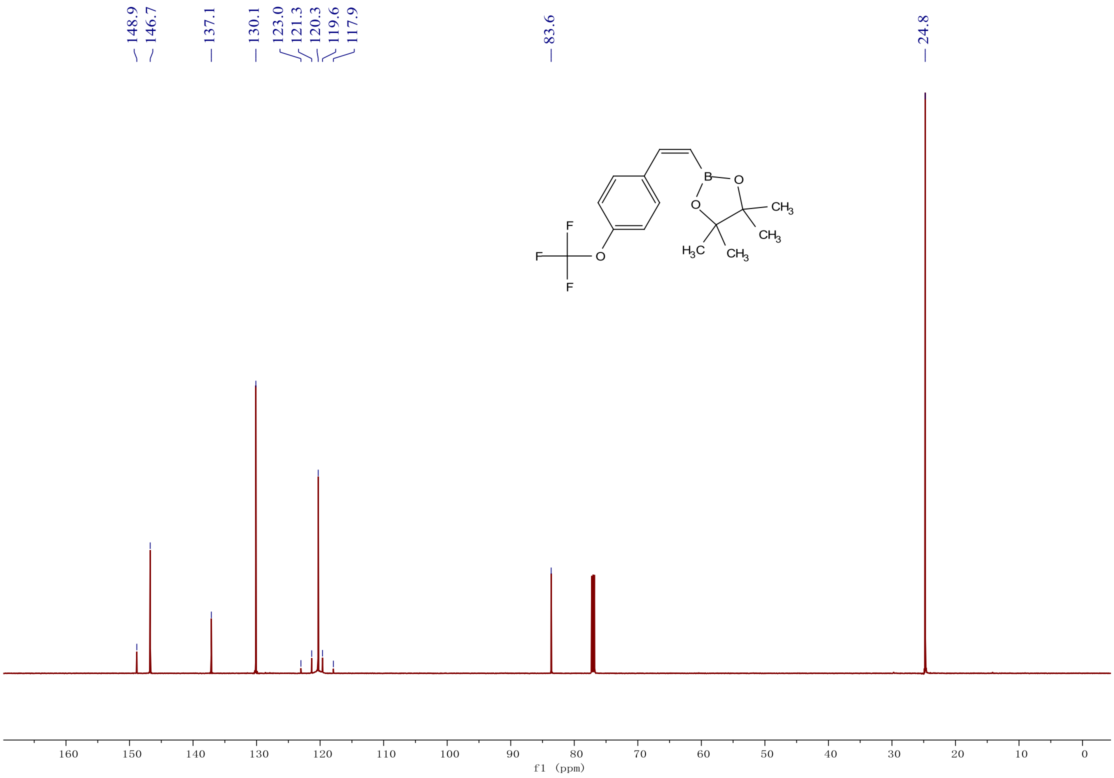

^{11}B NMR (193 MHz, Chloroform-*d*) of 7**b** (See procedure)

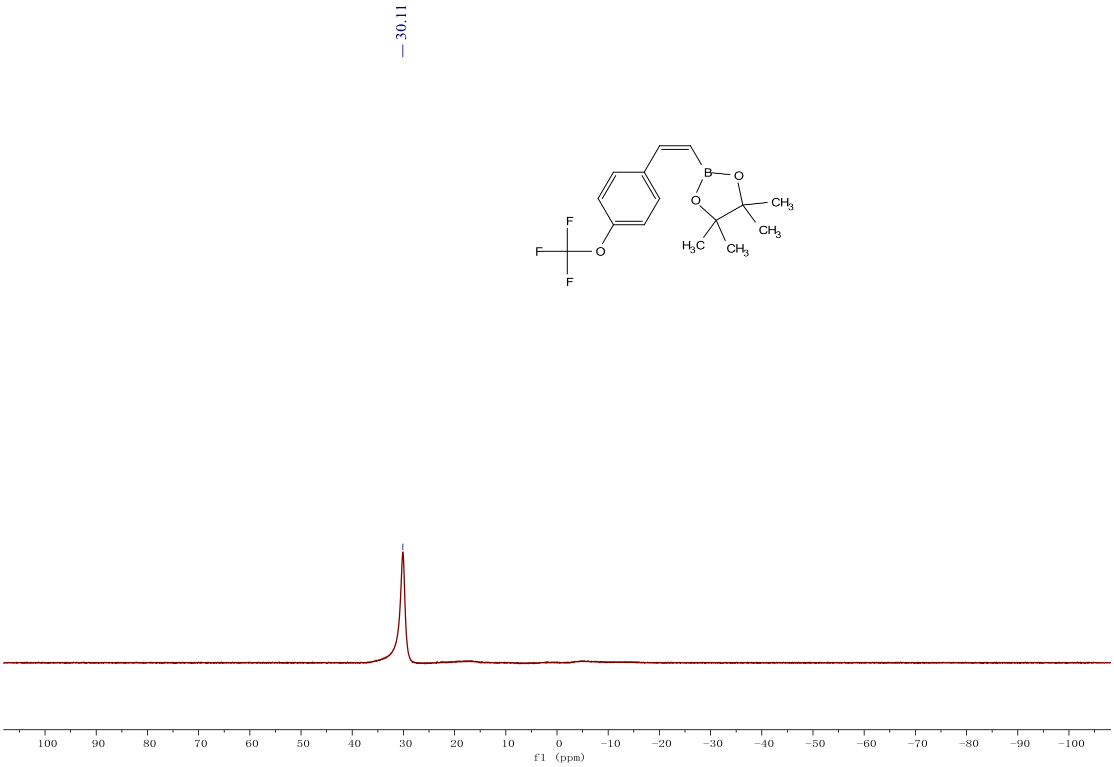

^{1}H NMR (400 MHz, Chloroform-*d*) of 8**b** (See procedure)

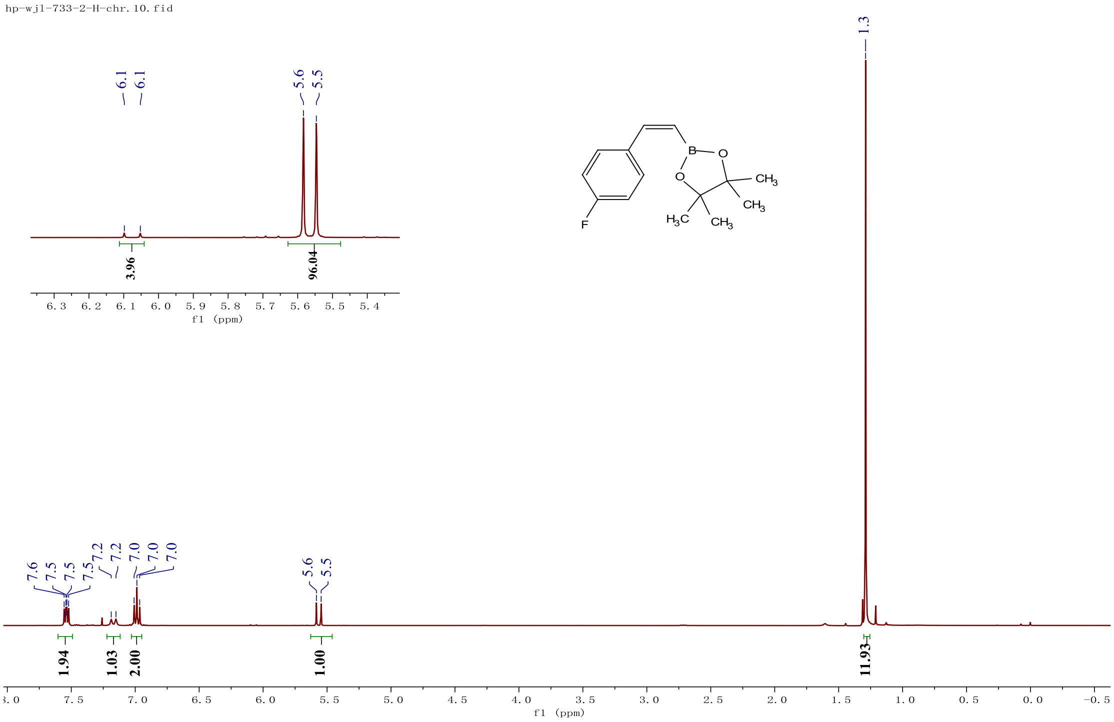

^{13}C NMR (101 MHz, Chloroform-*d*) of 8**b** (*See procedure*)

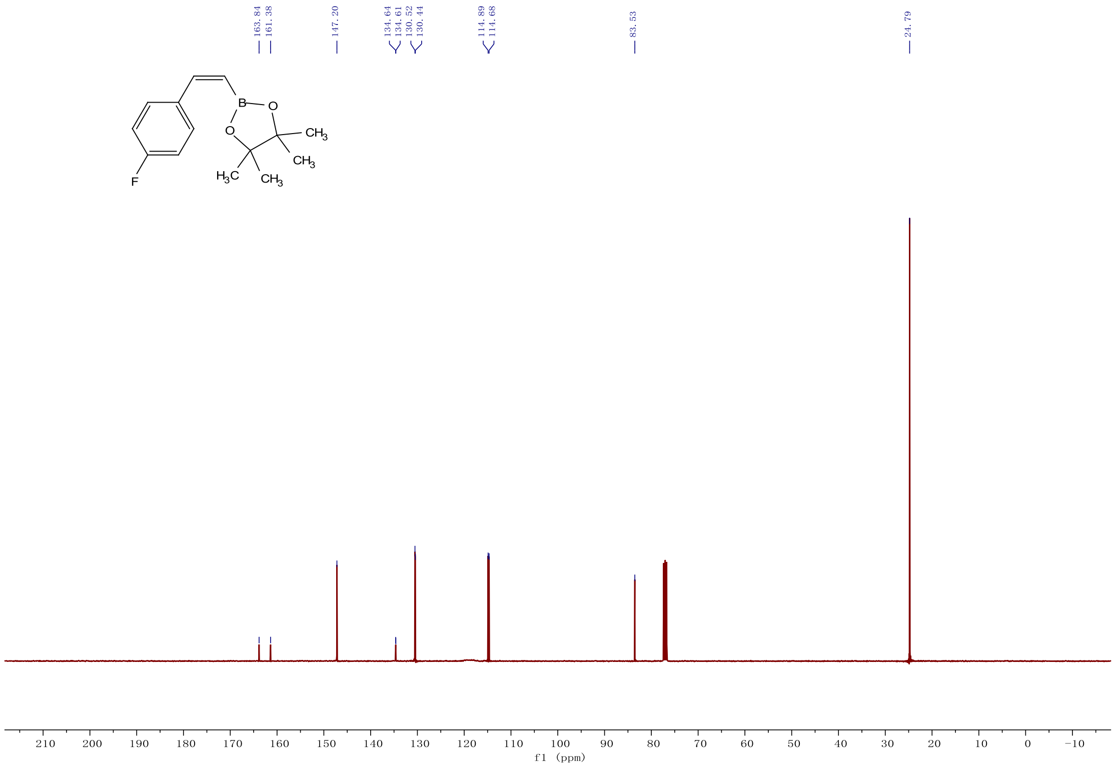

^{11}B NMR (128 MHz, Chloroform-*d*) of 8**b** (*See procedure*)

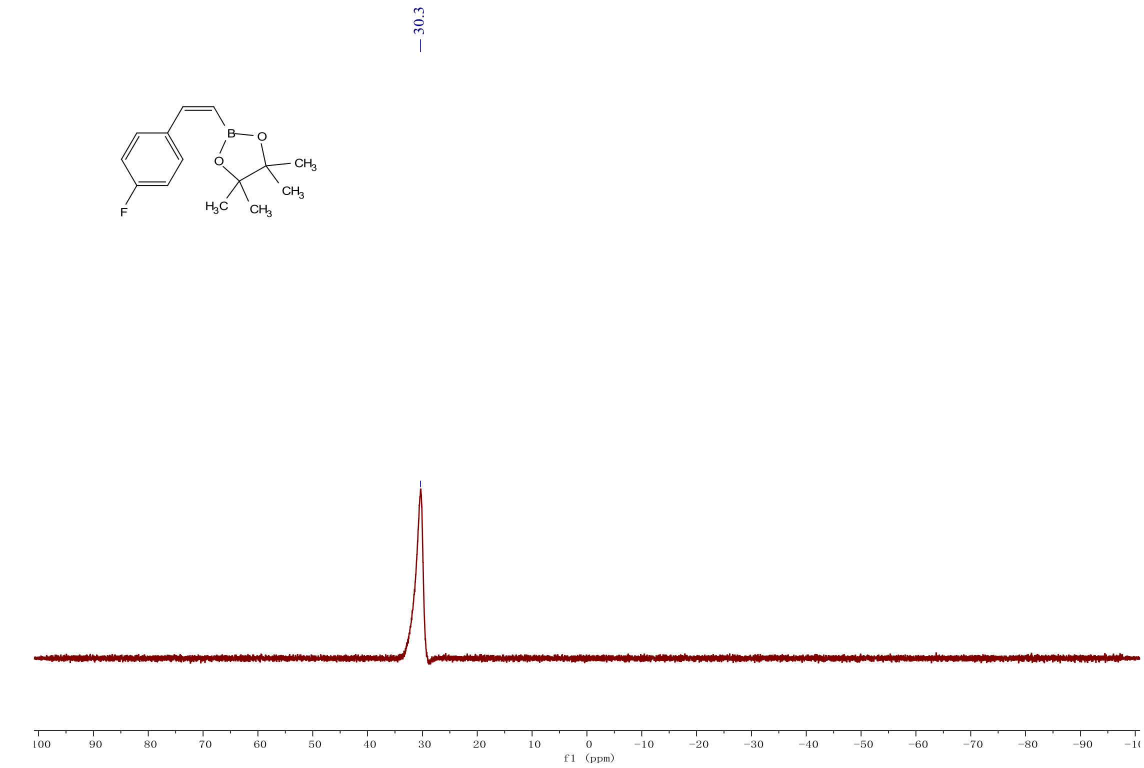

^{19}F NMR (377 MHz, Chloroform-*d*) of 8**b** (*See procedure*)

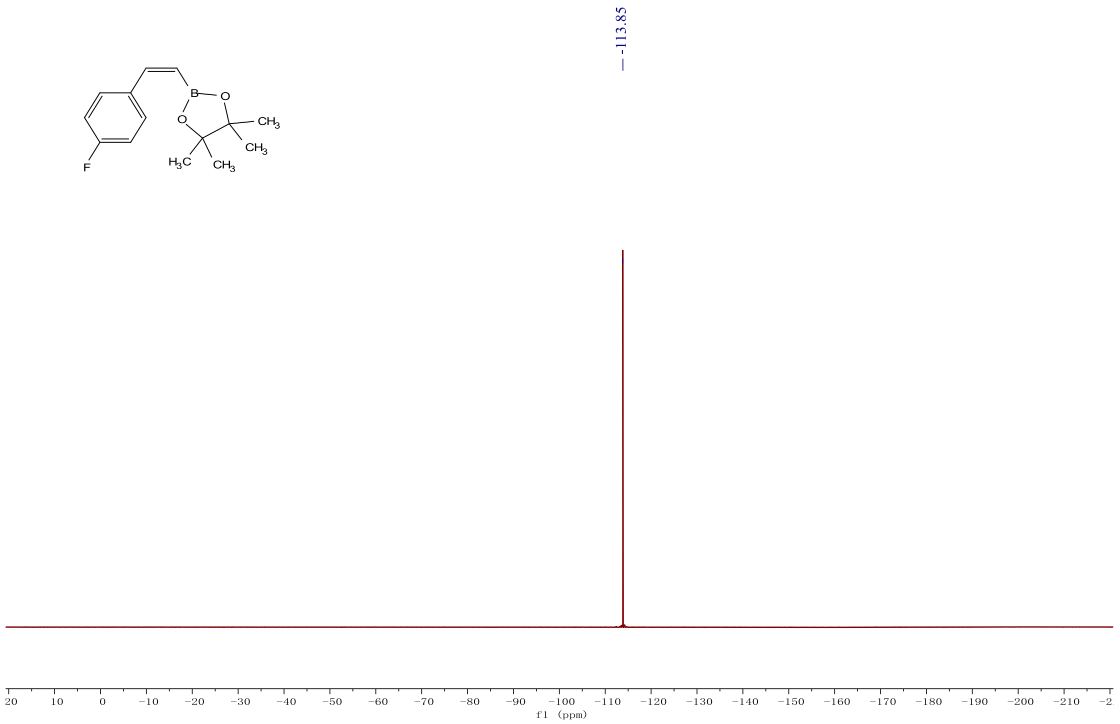

^{1}H NMR (400 MHz, Chloroform-*d*) of 9**b** (*See procedure*)

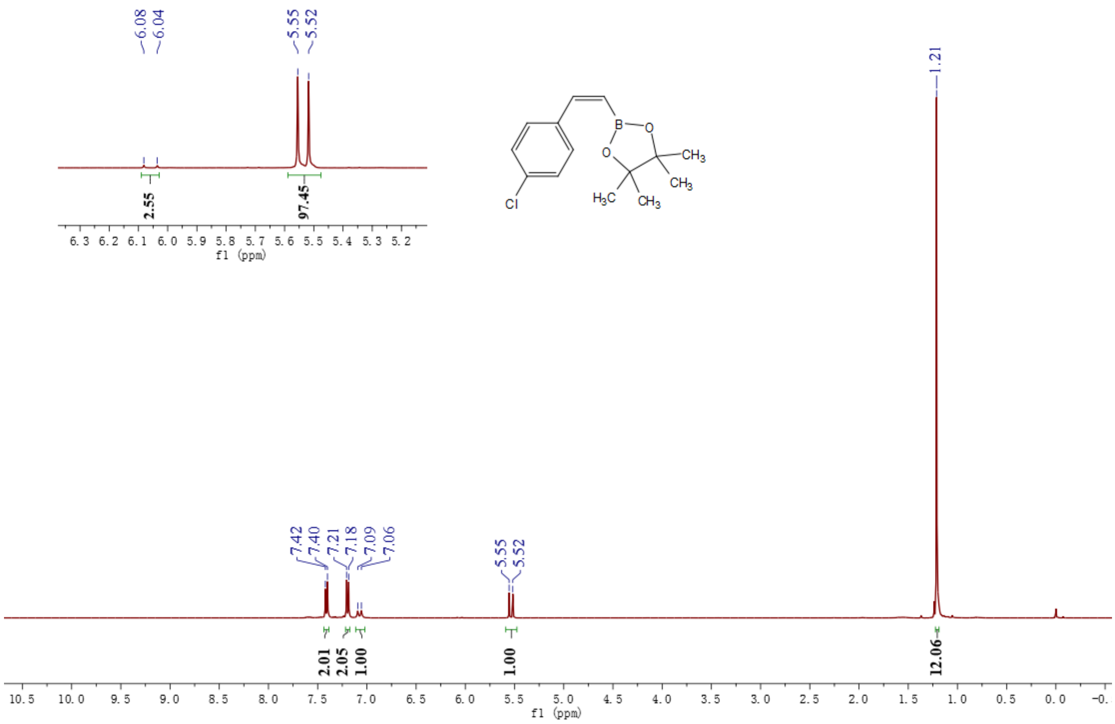

^{1}H NMR (400 MHz, Chloroform-*d*) of 10**b** (See procedure)

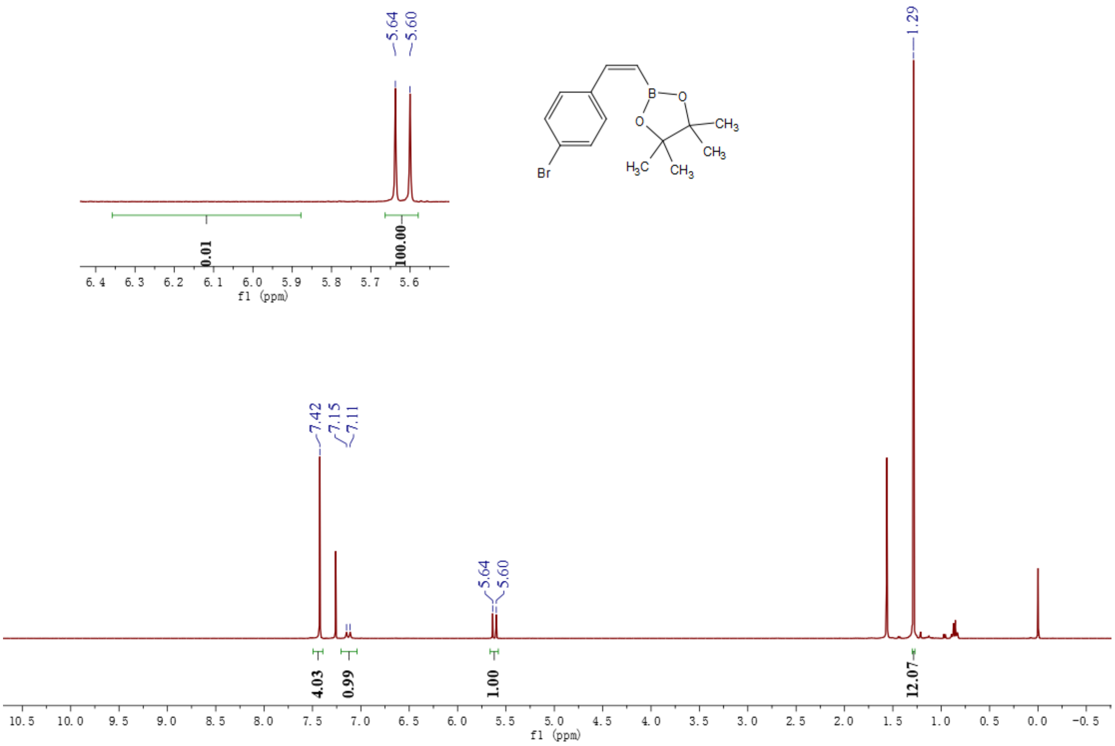

^{13}C NMR (151 MHz, Chloroform-*d*) of 10**b** (See procedure)

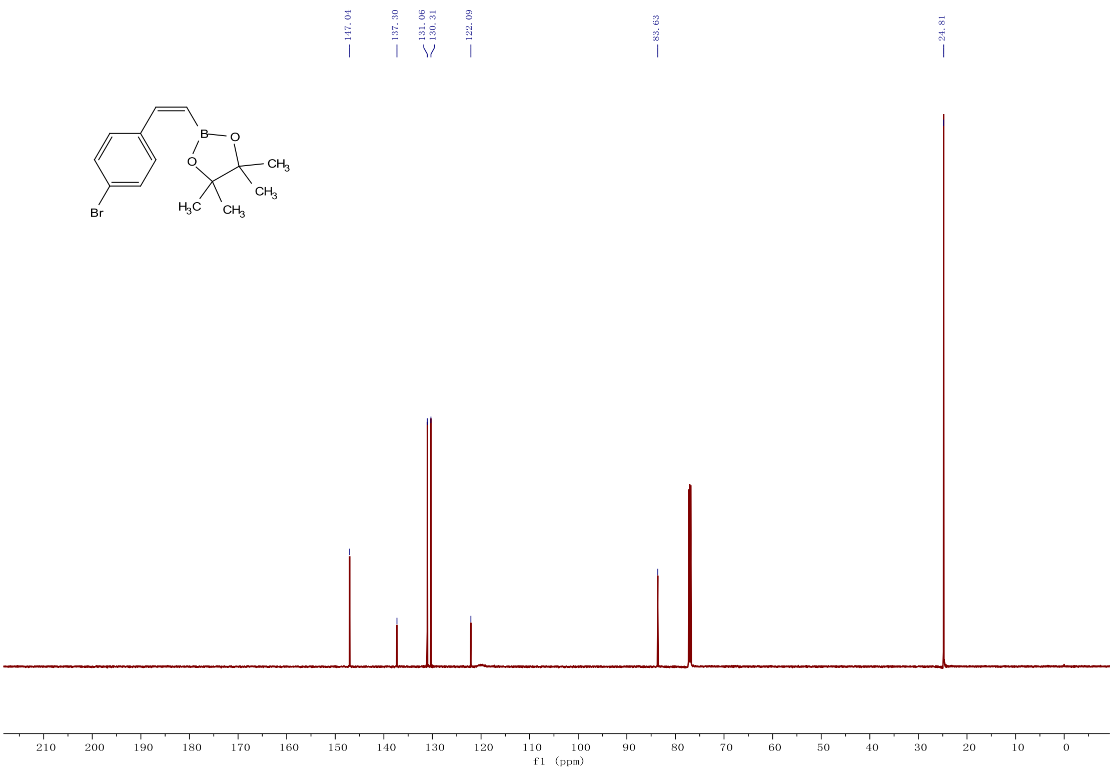

^{11}B NMR (193 MHz, Chloroform-*d*) of 10**b** (*See procedure*)

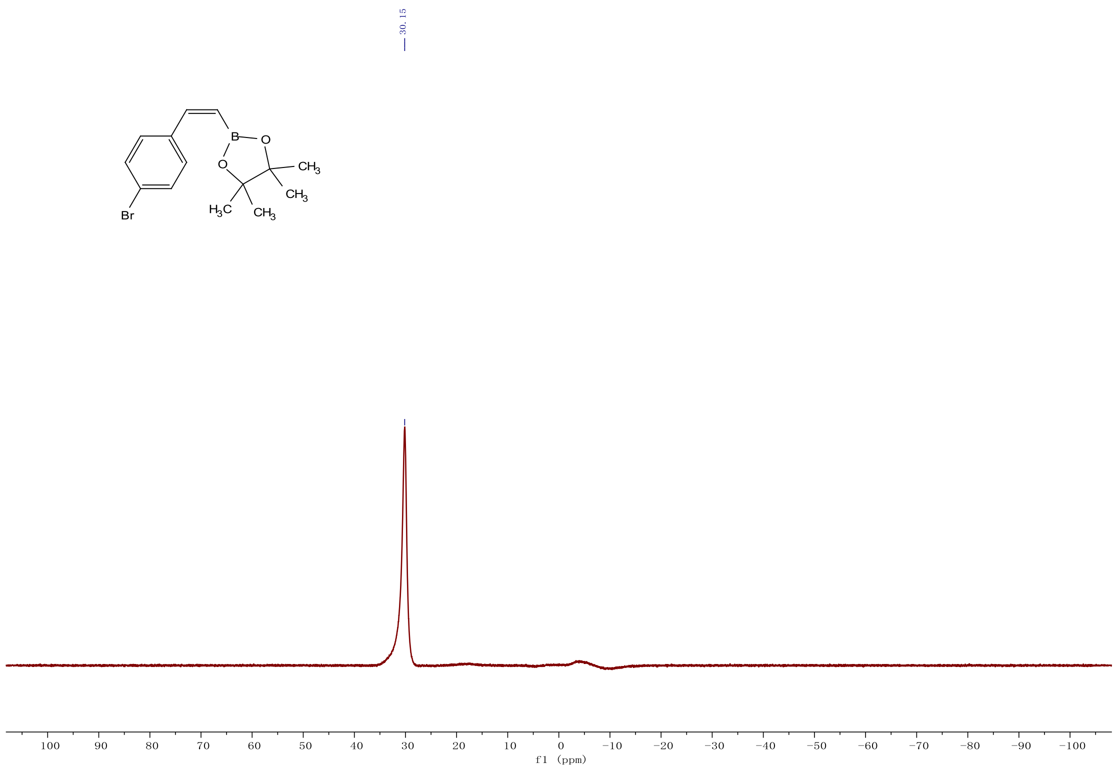

^{1}H NMR (400 MHz, Chloroform-*d*) of 11**b** (*See procedure*)

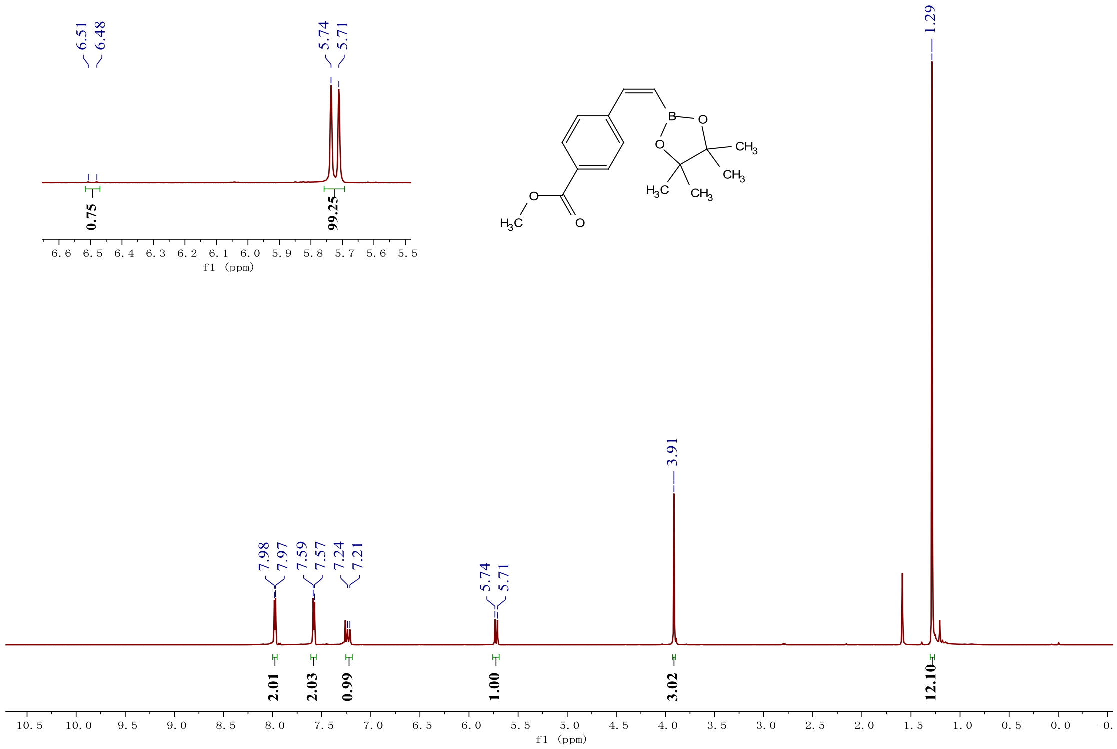

^{13}C NMR (151 MHz, Chloroform-*d*) of 11**b** (*See procedure*)

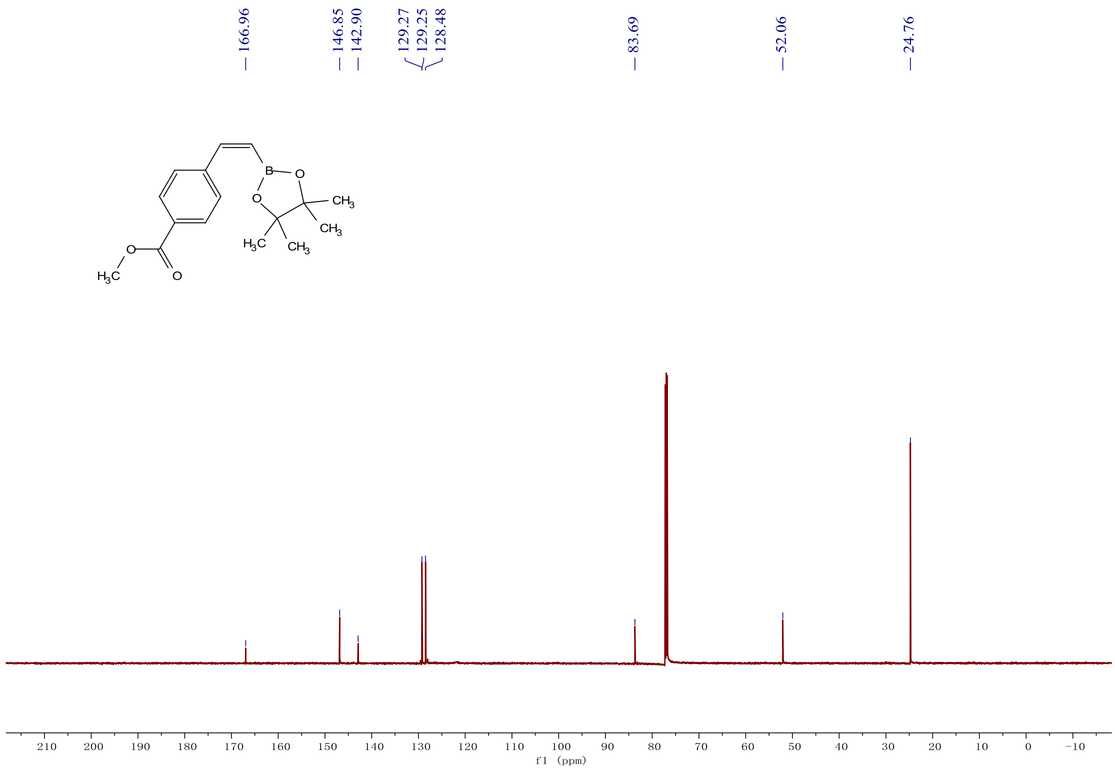

^{11}B NMR (193 MHz, Chloroform-*d*) of 11**b** (*See procedure*)

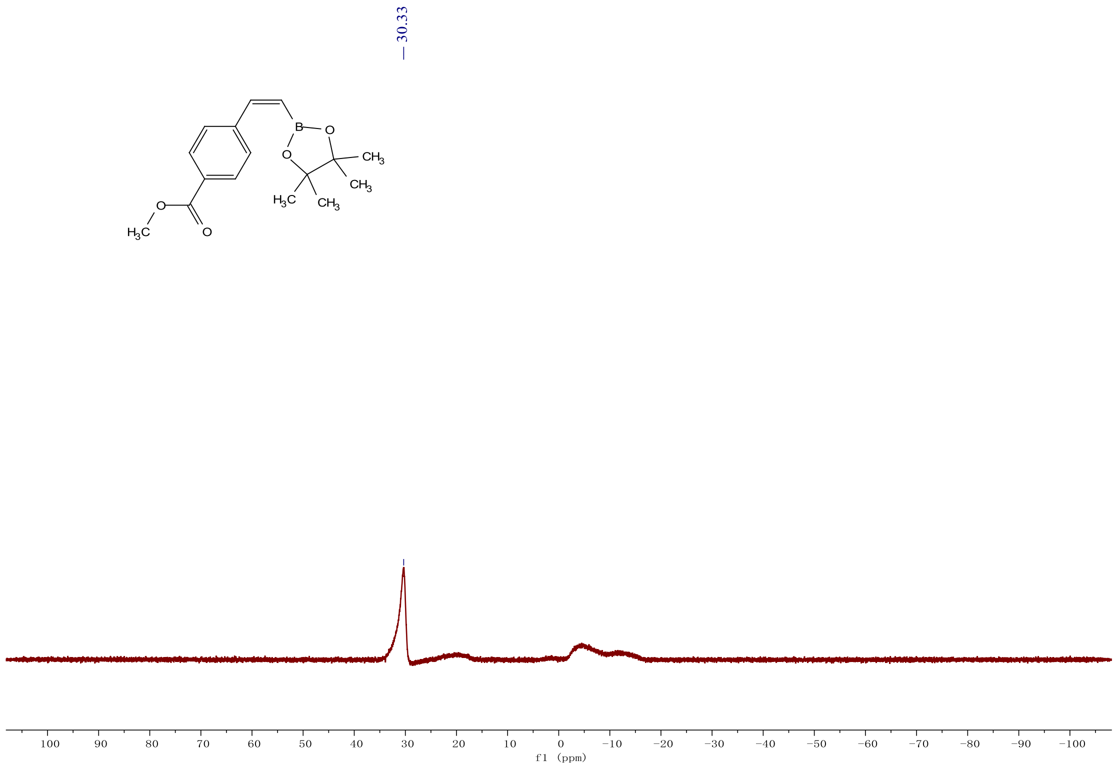

^{1}H NMR (400 MHz, Chloroform-*d*) of 12**b** (*See procedure*)

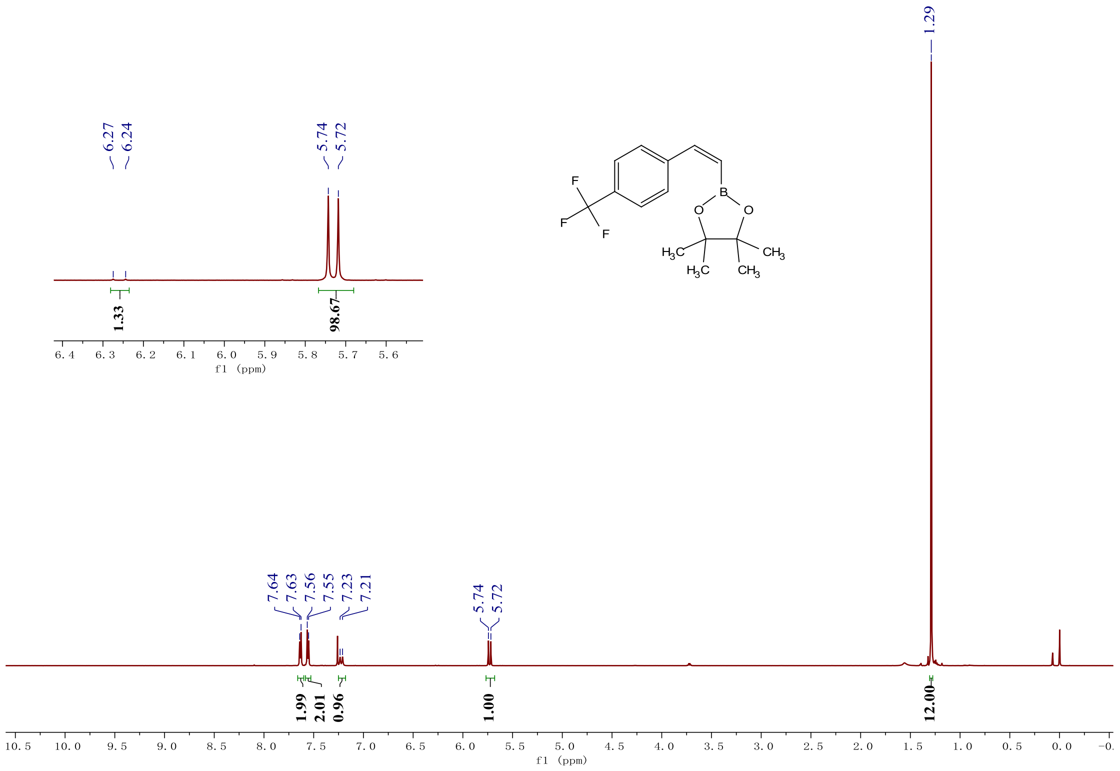

^{13}C NMR (151 MHz, Chloroform-*d*) of 12**b** (*See procedure*)

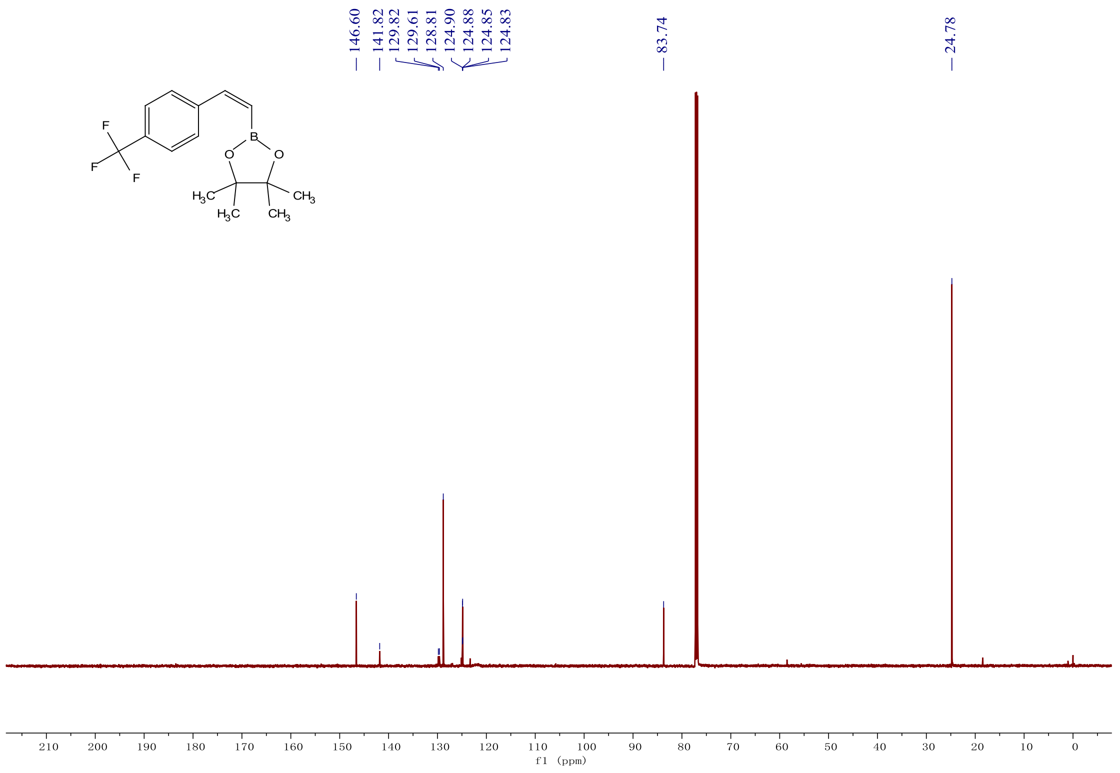

^{11}B NMR (193 MHz, Chloroform-*d*) of 12**b** (*See procedure*)

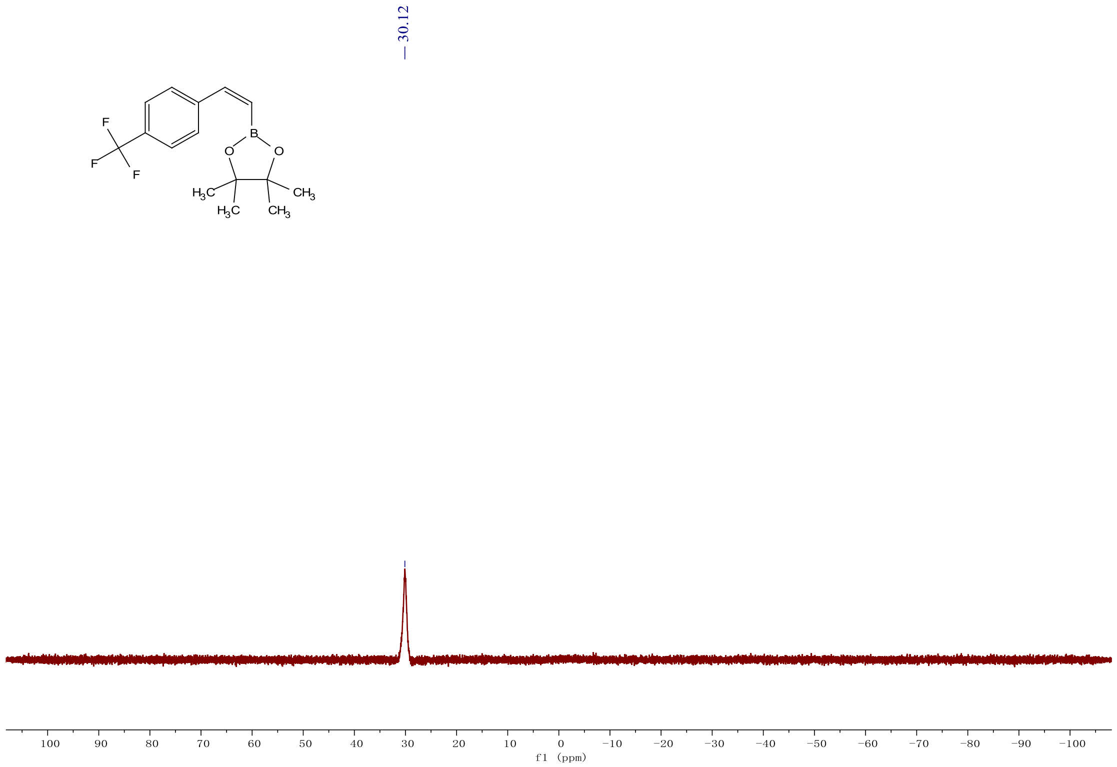

^{19}F NMR (565 MHz, Chloroform-*d*) of 12**b** (*See procedure*)

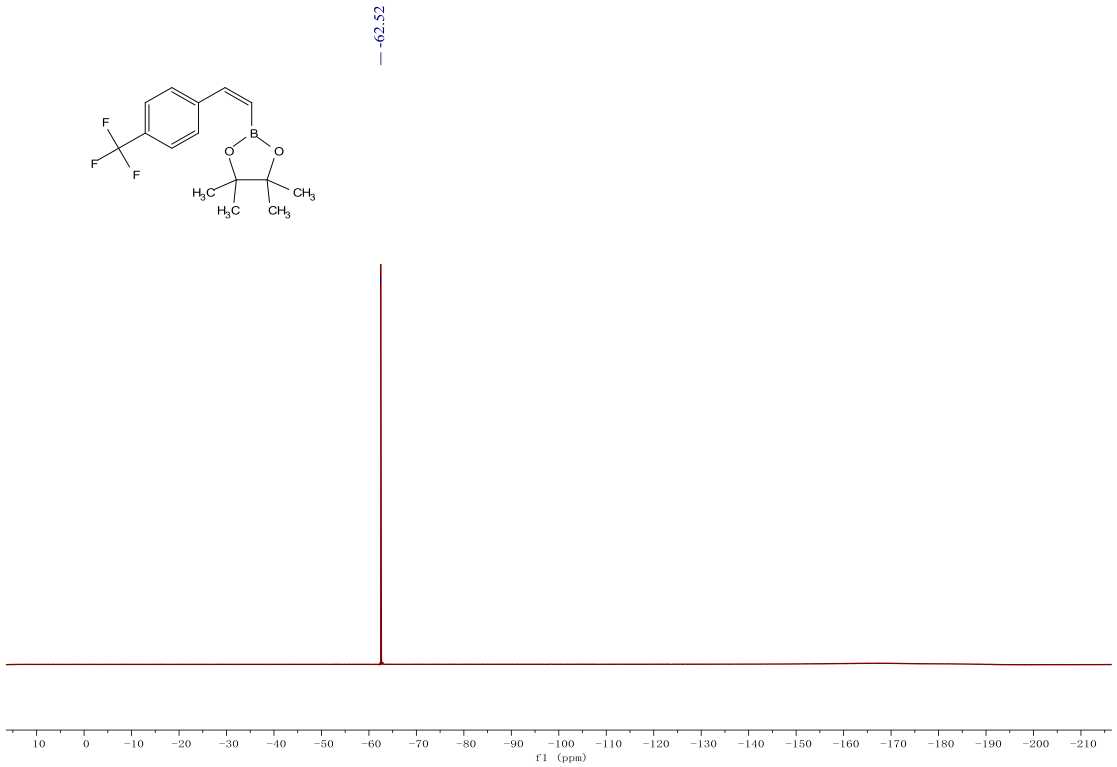

^{1}H NMR (400 MHz, Chloroform-*d*) of 13**b** (*See procedure*)

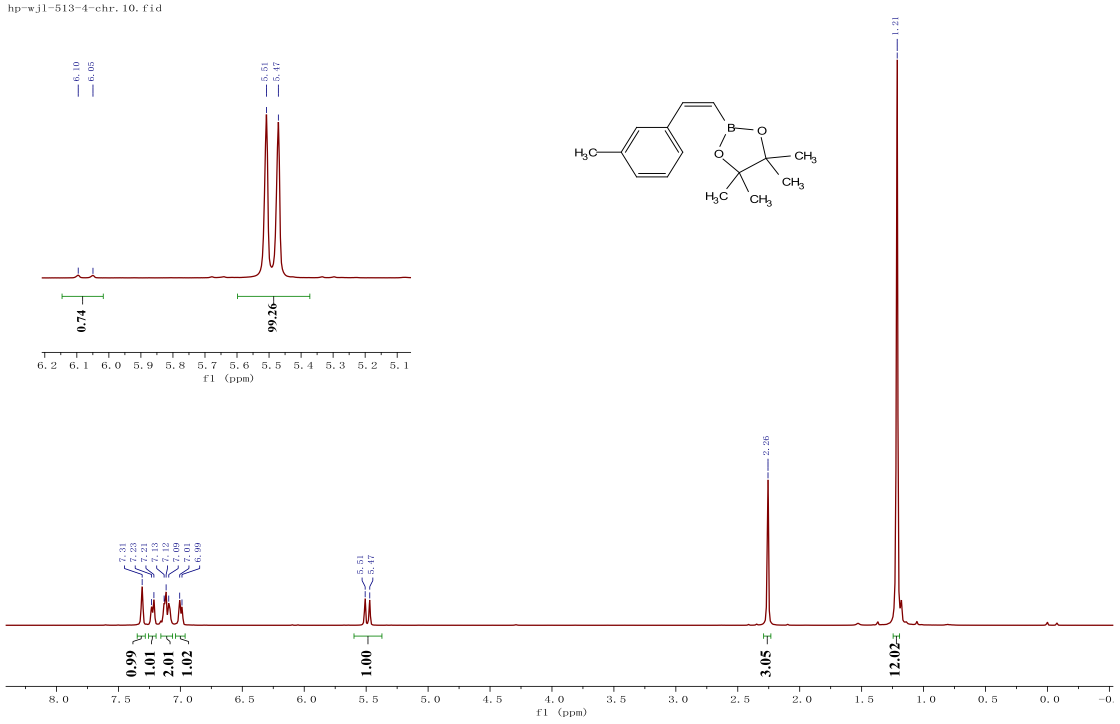

^{13}C NMR (151 MHz, Chloroform-*d*) of 13**b** (*See procedure*)

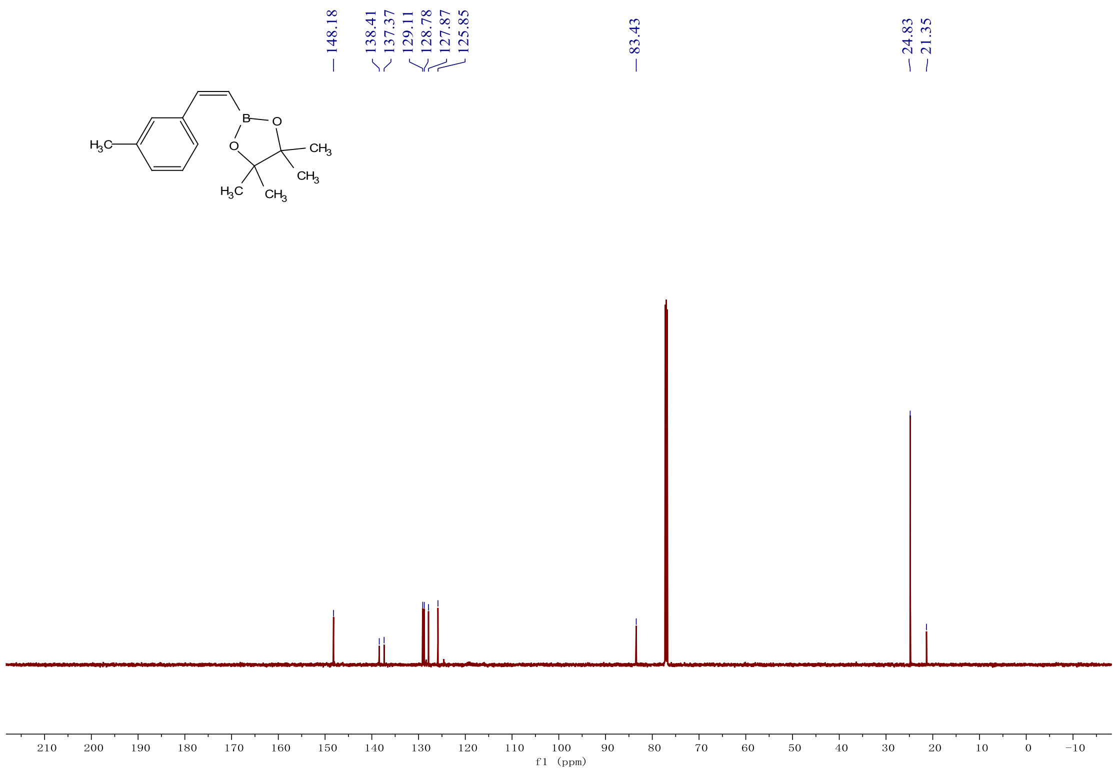

^{11}B NMR (193 MHz, Chloroform-*d*) of 13**b** (See procedure)

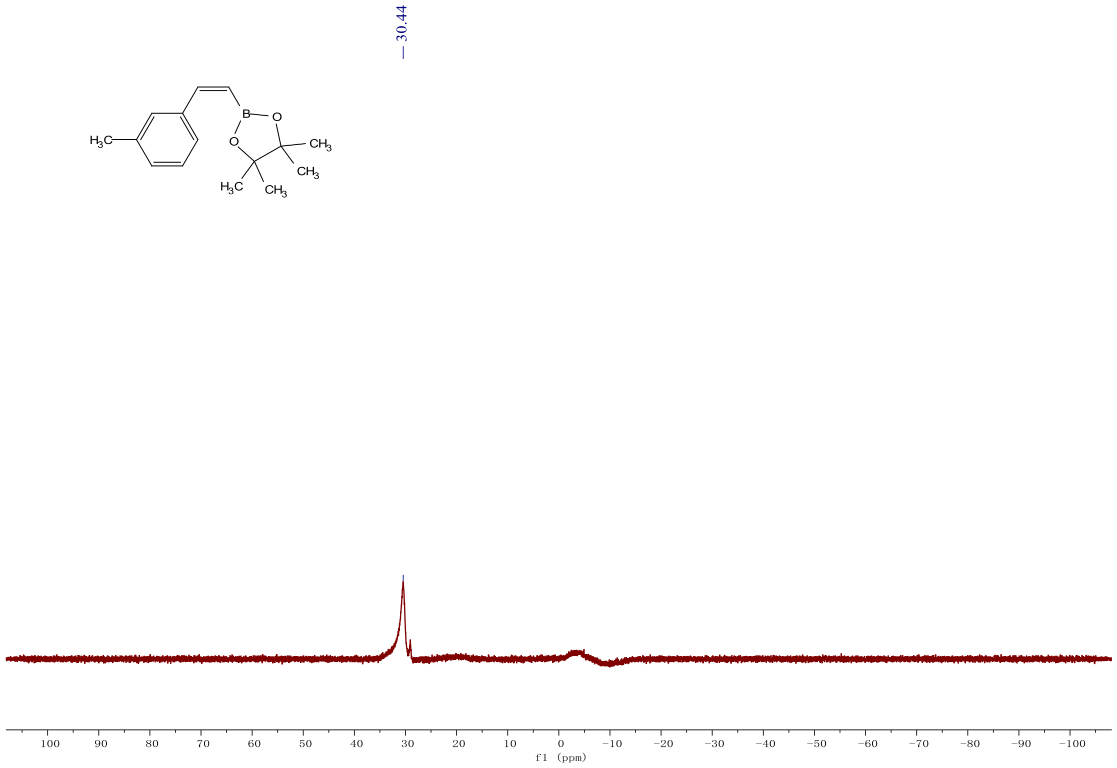

^{1}H NMR (400 MHz, Chloroform-*d*) of 14**b** (See procedure)

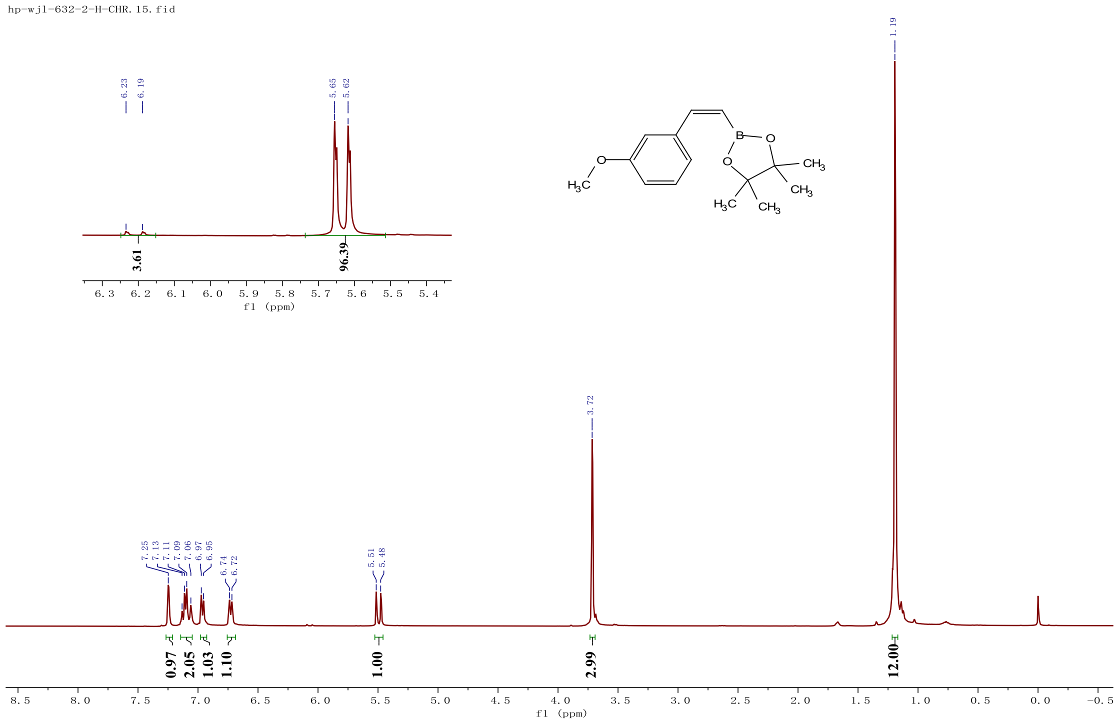

^{1}H NMR (400 MHz, Chloroform-*d*) of 15**b** (<u>*See procedure*</u>)

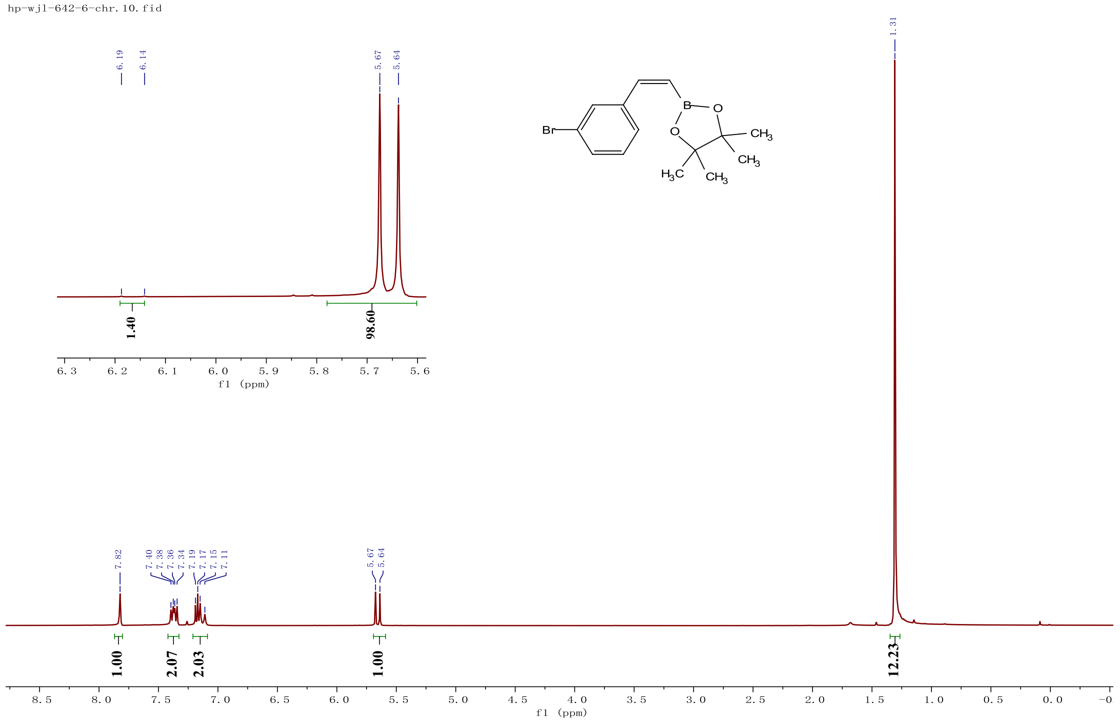

^{13}C NMR (151 MHz, Chloroform-*d*) of 15**b** (<u>*See procedure*</u>)

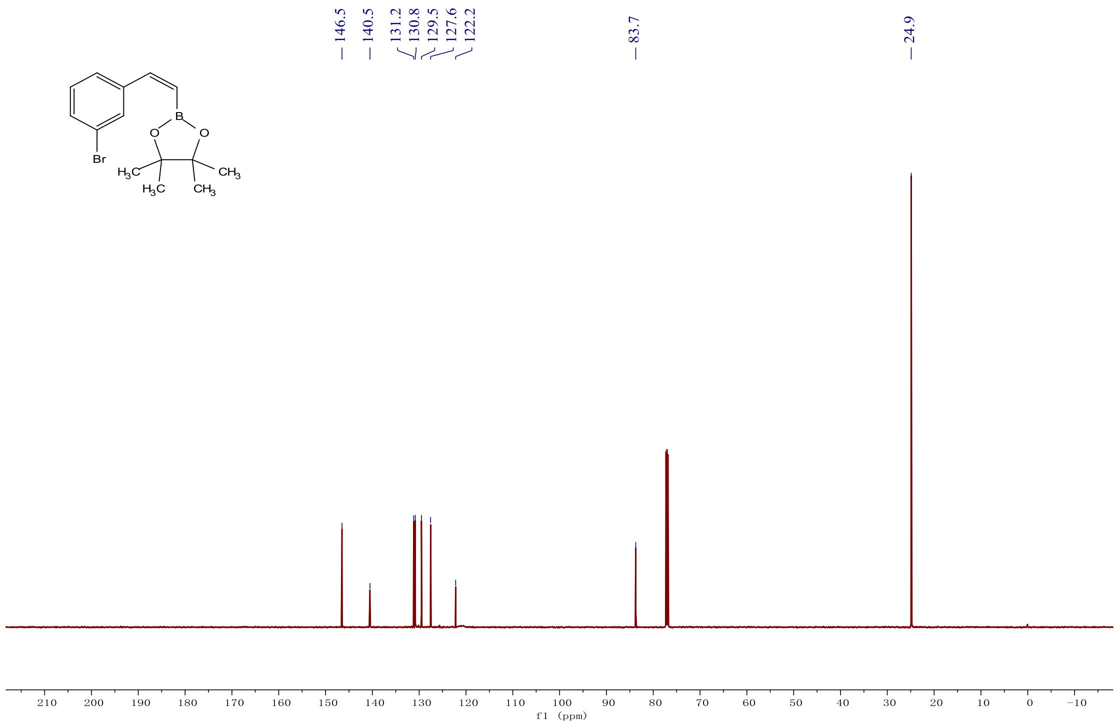

^{11}B NMR (193 MHz, Chloroform-*d*) of 15**b** (*See procedure*)

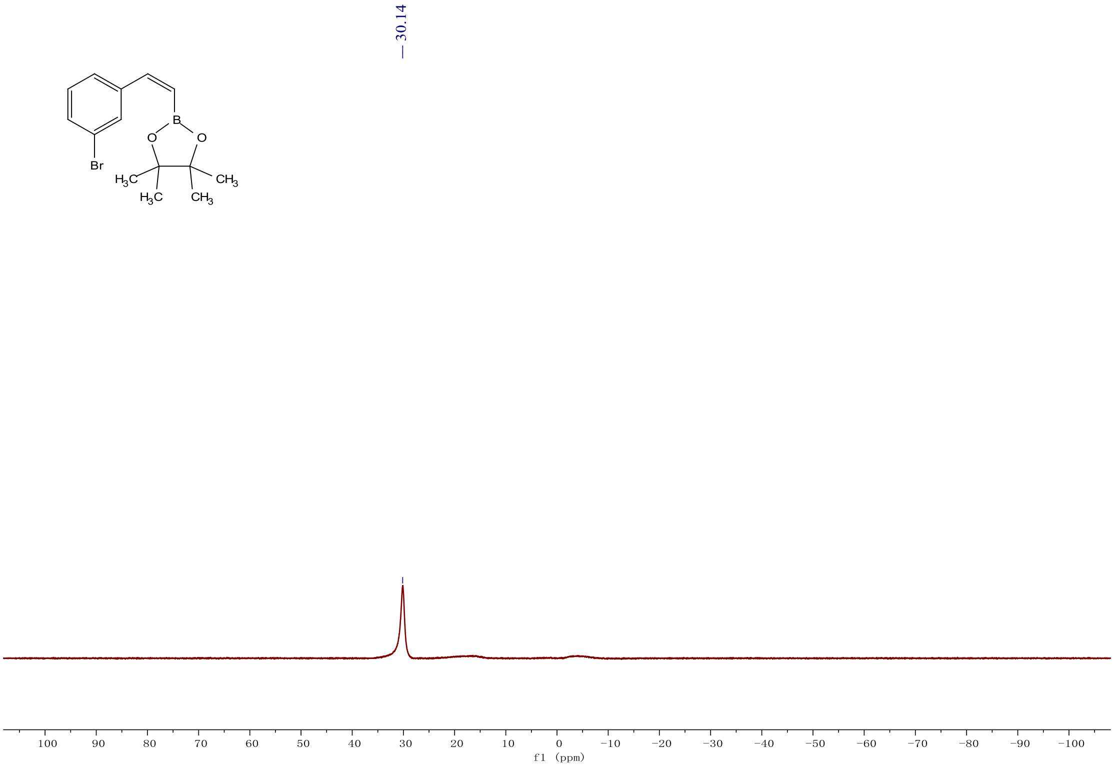

^{1}H NMR (400 MHz, Chloroform-*d*) of 16**b** (*See procedure*)

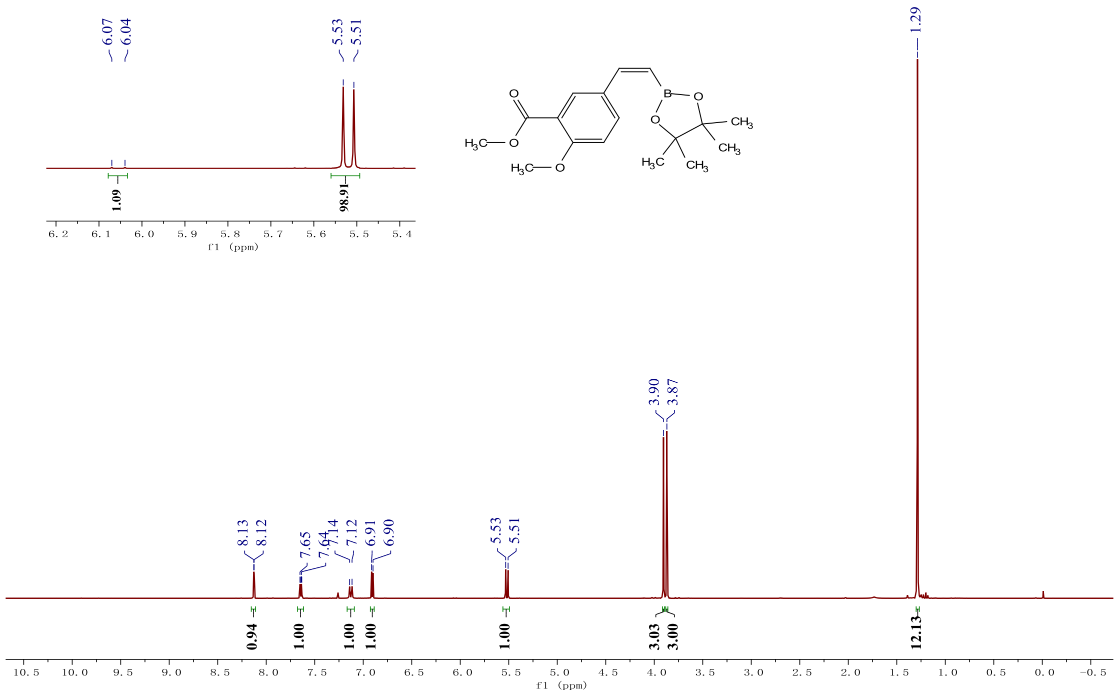

^{13}C NMR (151 MHz,Chloroform-*d*) of 16**b** (*See procedure*)

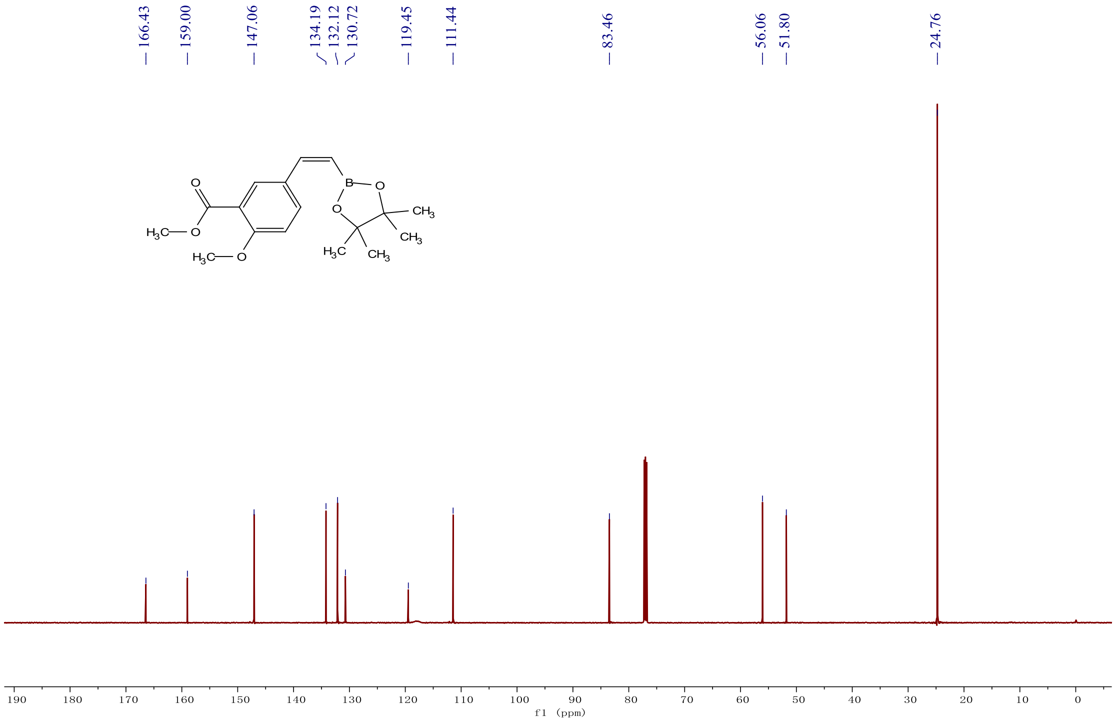

^{11}B NMR (193 MHz, Chloroform-*d*) of 16**b** (See procedure)

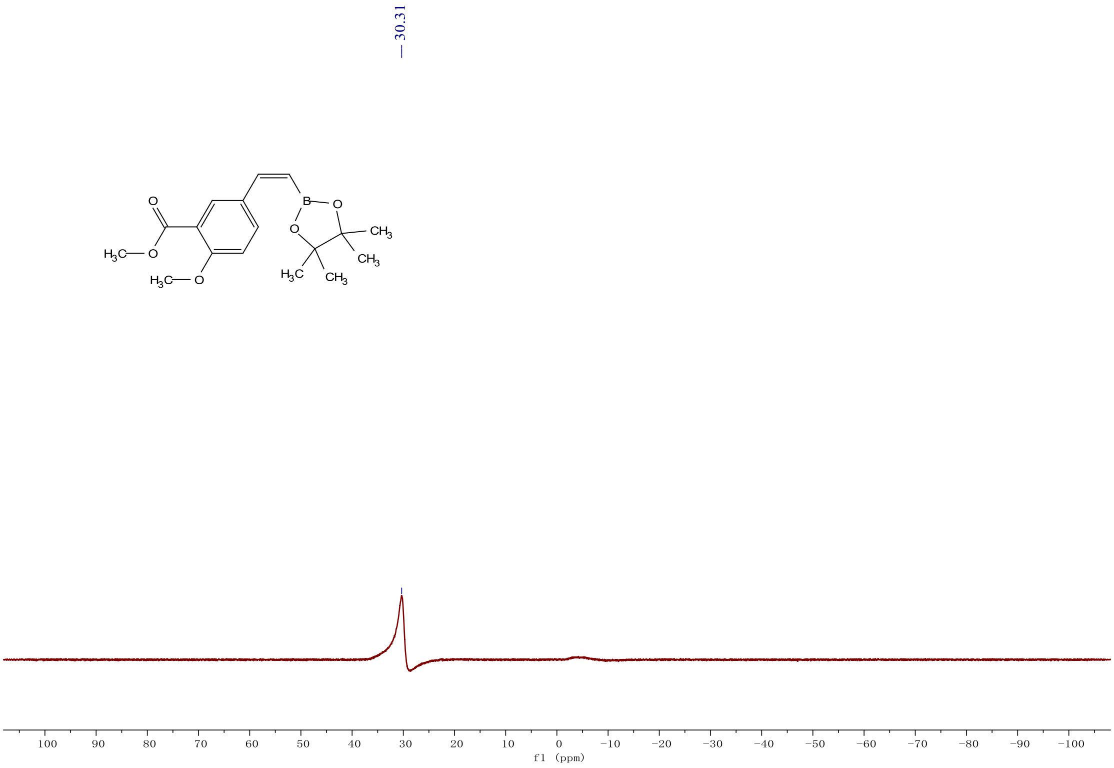

^{1}H NMR (400 MHz, Chloroform-*d*) of 17**b** (See procedure)

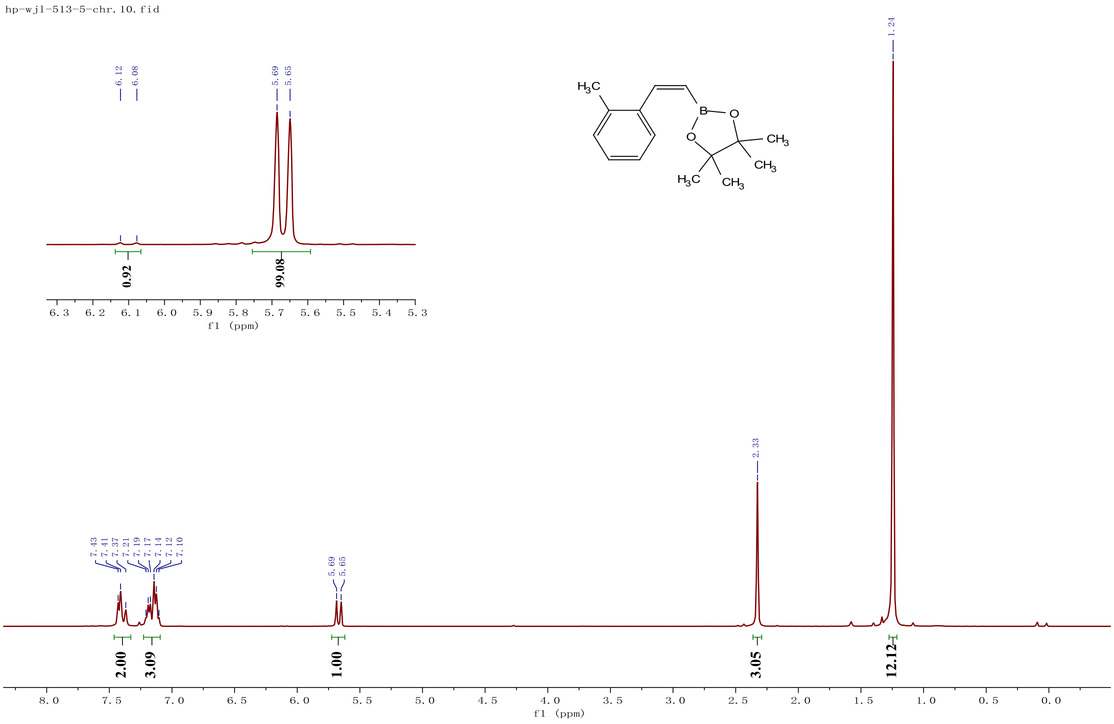

^{1}H NMR (400 MHz, Chloroform-*d*) of 18**b** (See procedure)

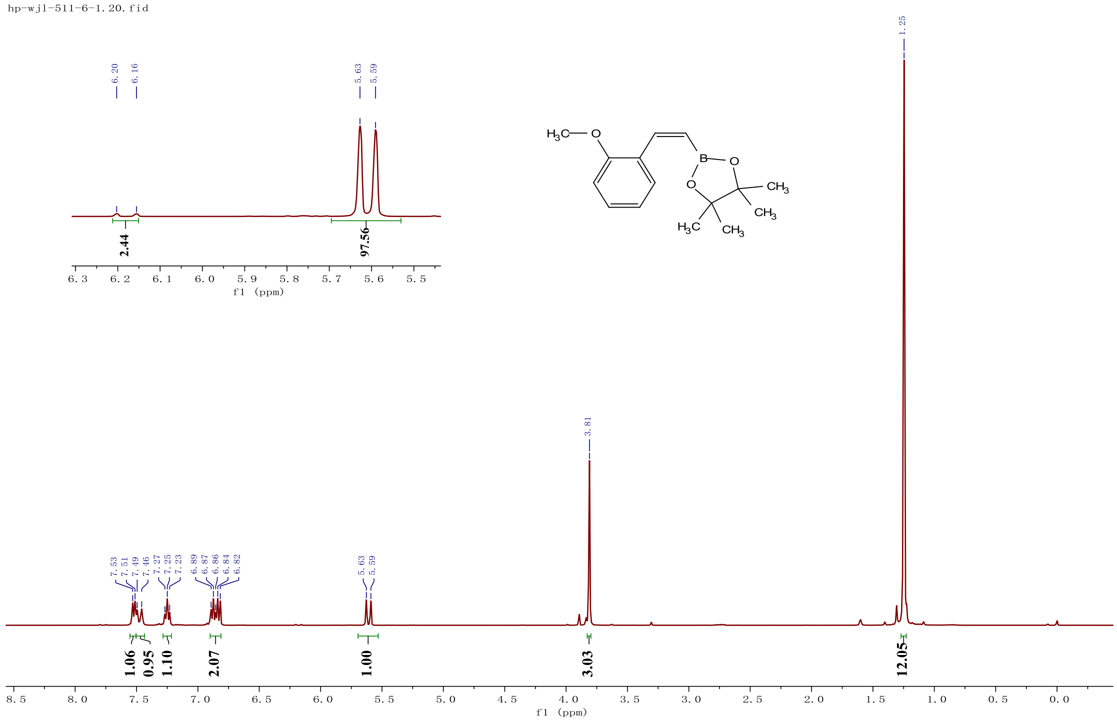

^{1}H NMR (400 MHz, Chloroform-*d*) of 19**b** (See procedure)

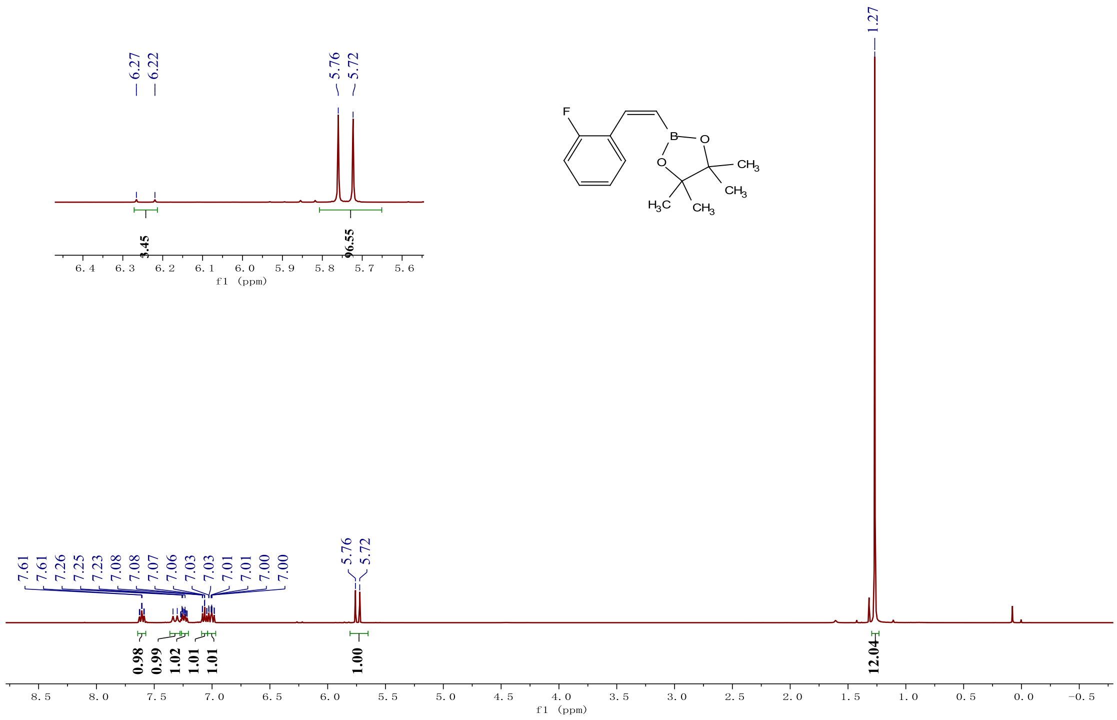

^{13}C NMR (151 MHz, Chloroform-*d*) of 19**b** (*See procedure*)

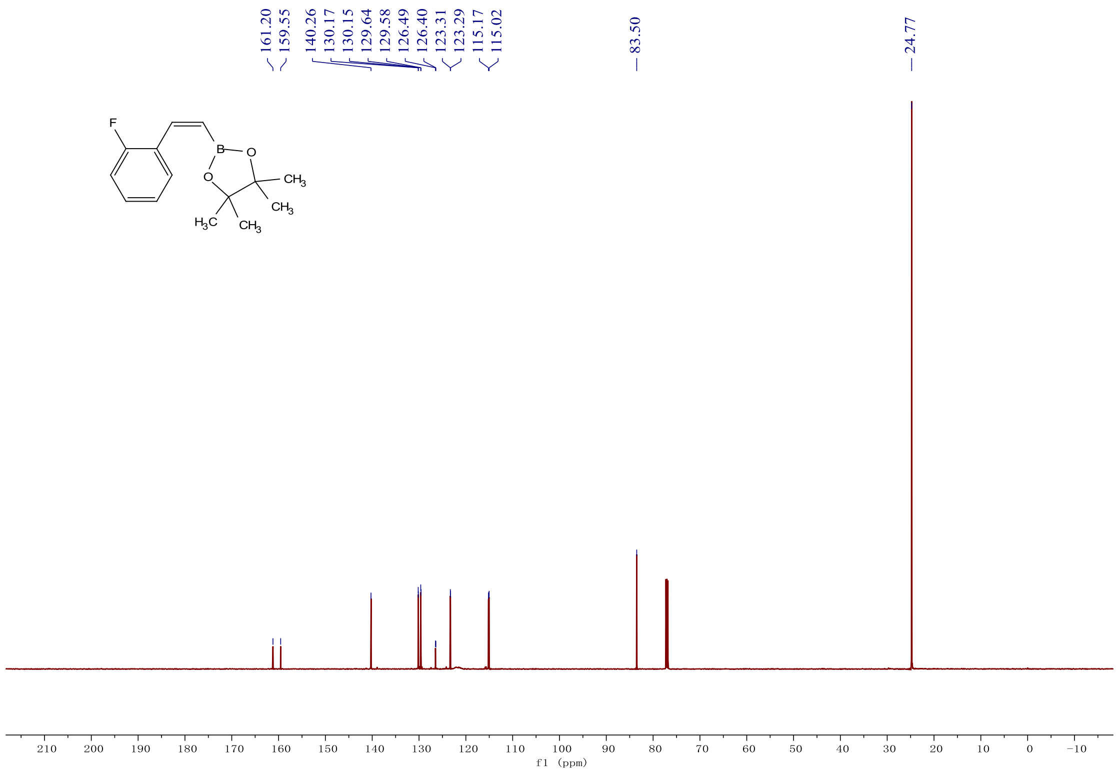

^{11}B NMR (193 MHz, Chloroform-*d*) of 19**b** (*See procedure*)

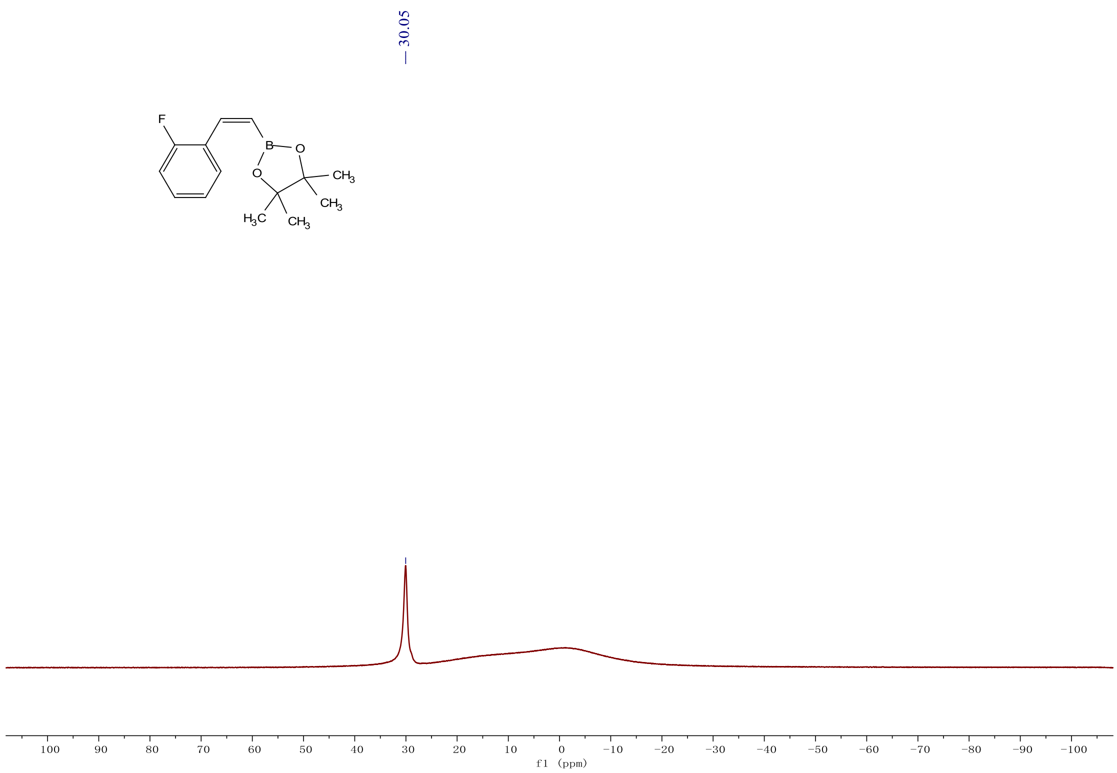

^{19}F NMR (565 MHz, Chloroform-*d*) of 19**b** (See procedure)

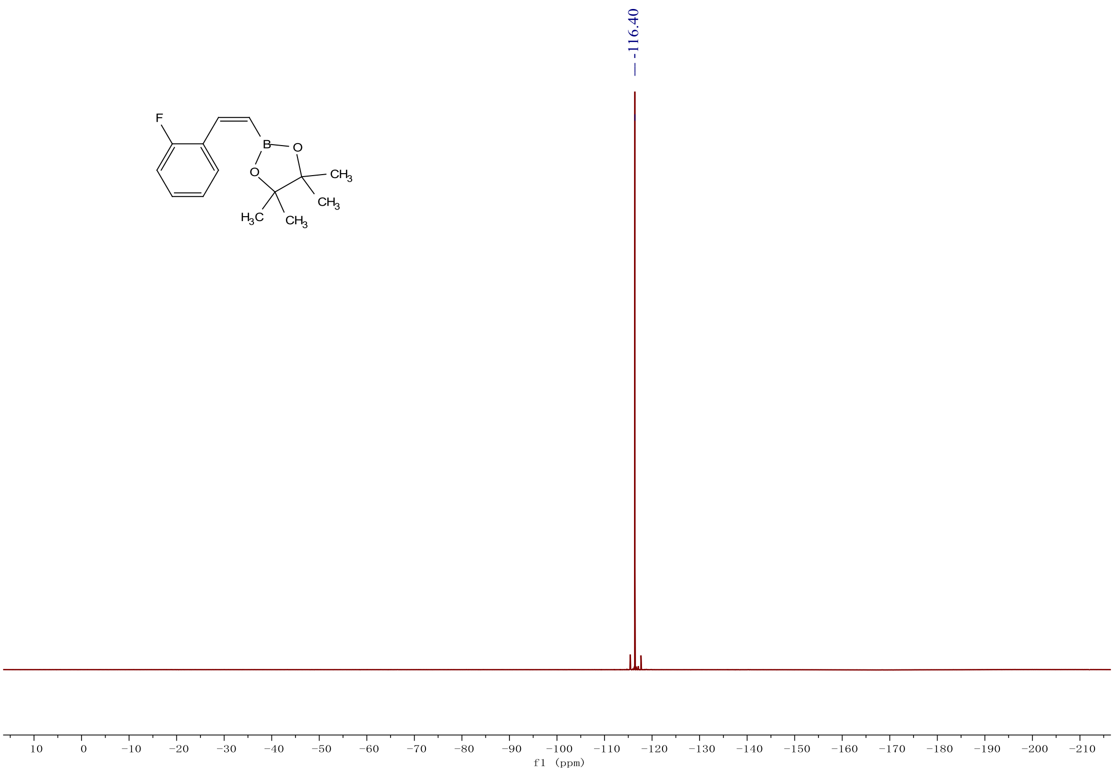

^{1}H NMR (400 MHz, Chloroform-*d*) of 20**b** (See procedure)

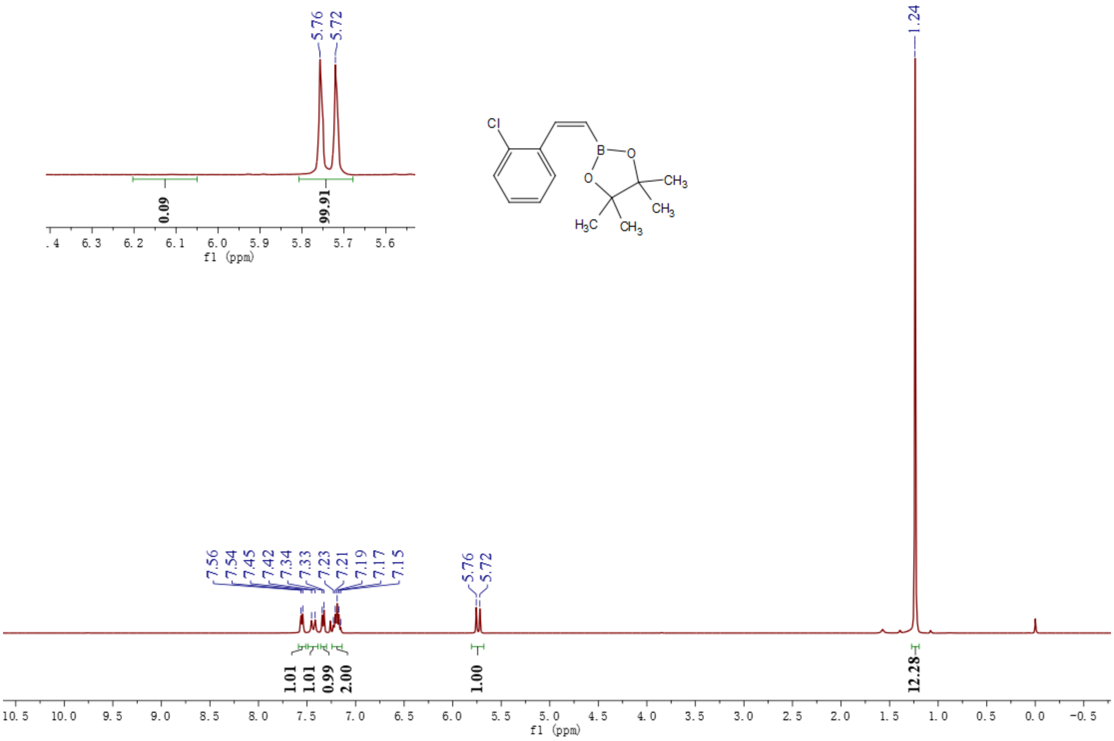

^{13}C NMR (101 MHz, Chloroform-*d*) of 20**b** (*See procedure*)

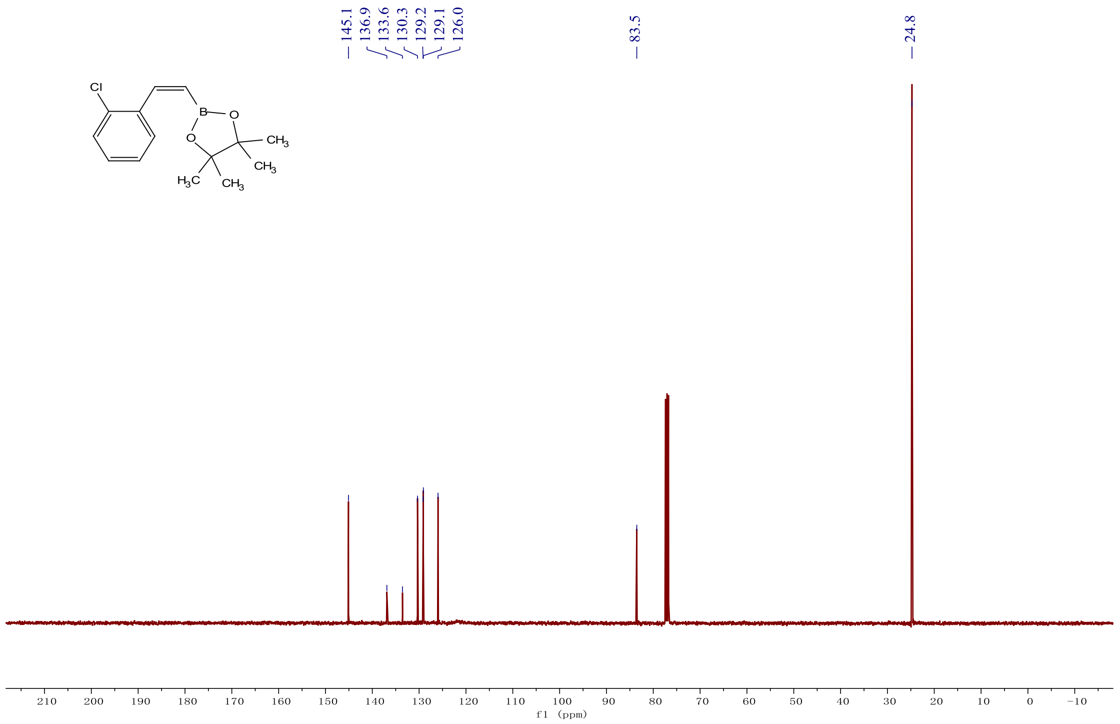

^{11}B NMR (128 MHz, Chloroform-*d*) of 20**b** (*See procedure*)

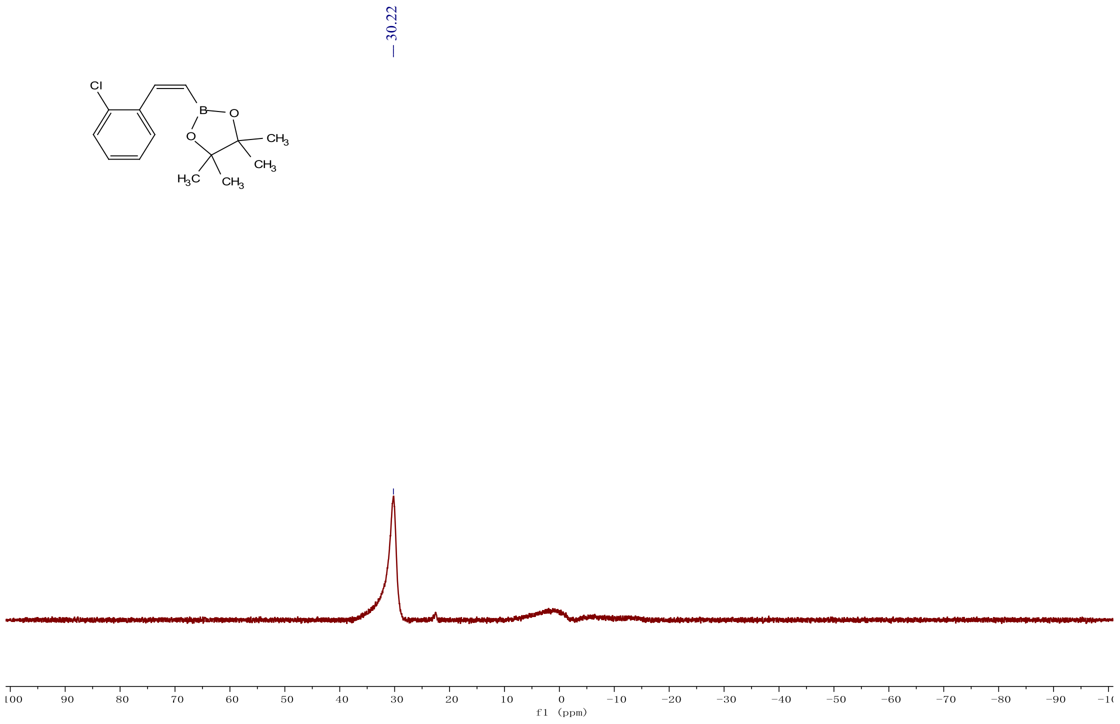

^{1}H NMR (400 MHz, Chloroform-*d*) of 21**b** (See procedure)

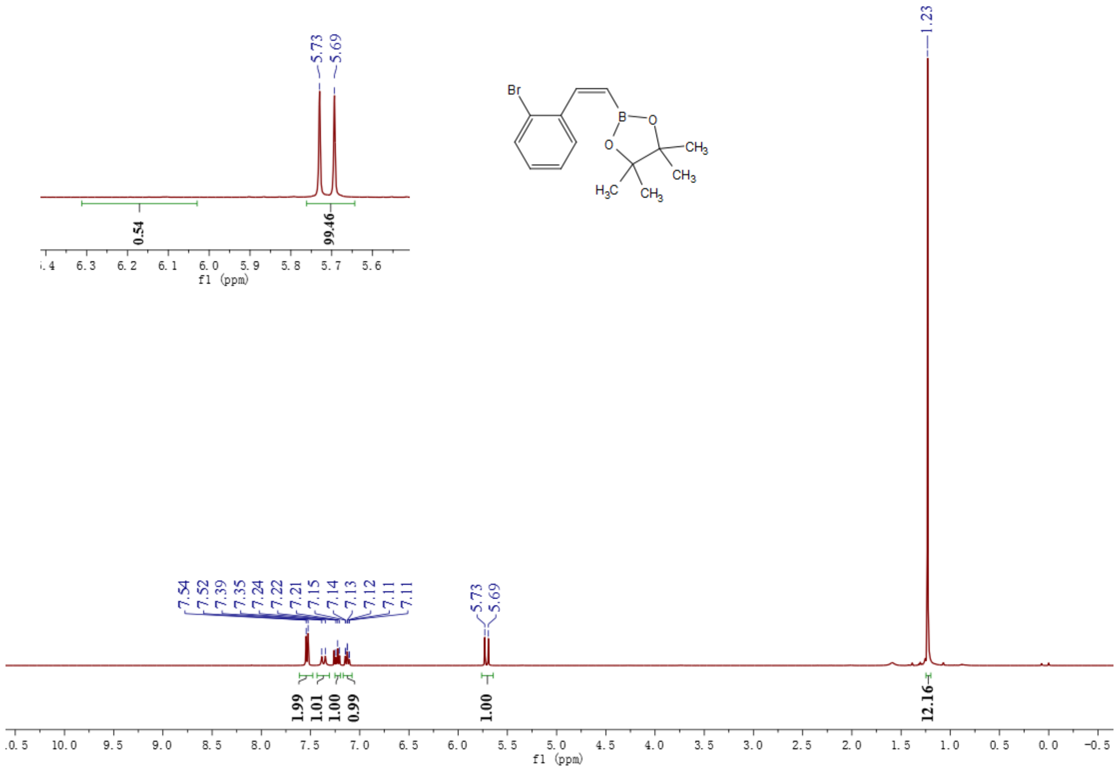

^{13}C NMR (151 MHz, Chloroform-*d*) of 21**b** (See procedure)

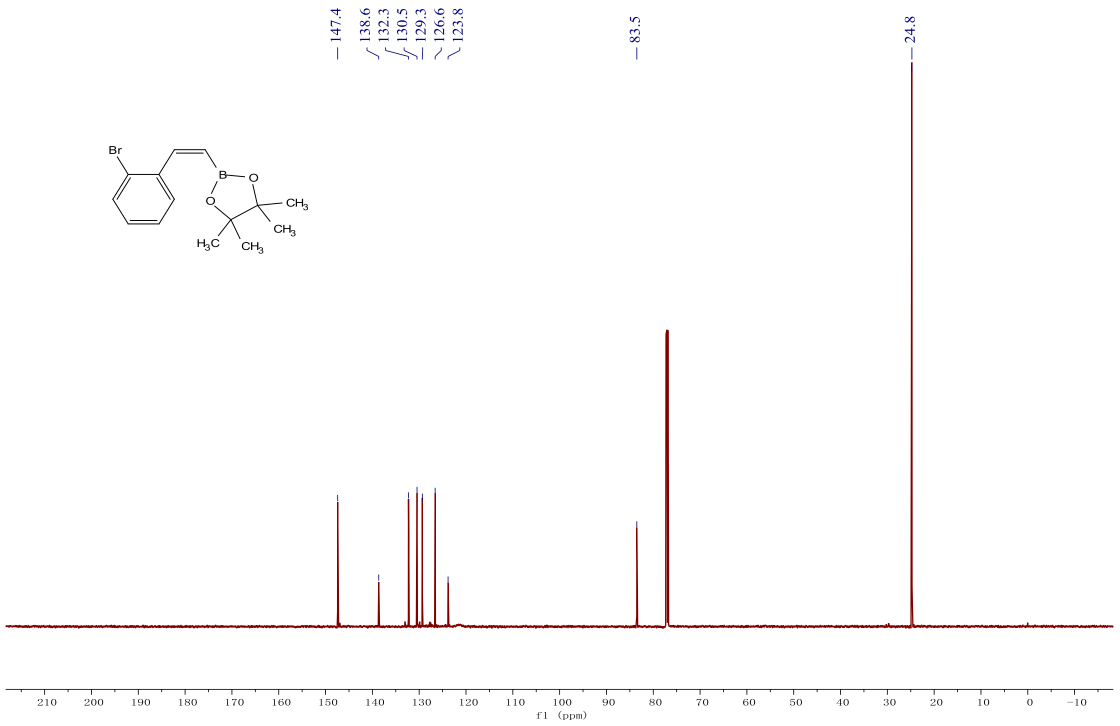

^{11}B NMR (193 MHz, Chloroform-*d*) of 21**b** (*See procedure*)

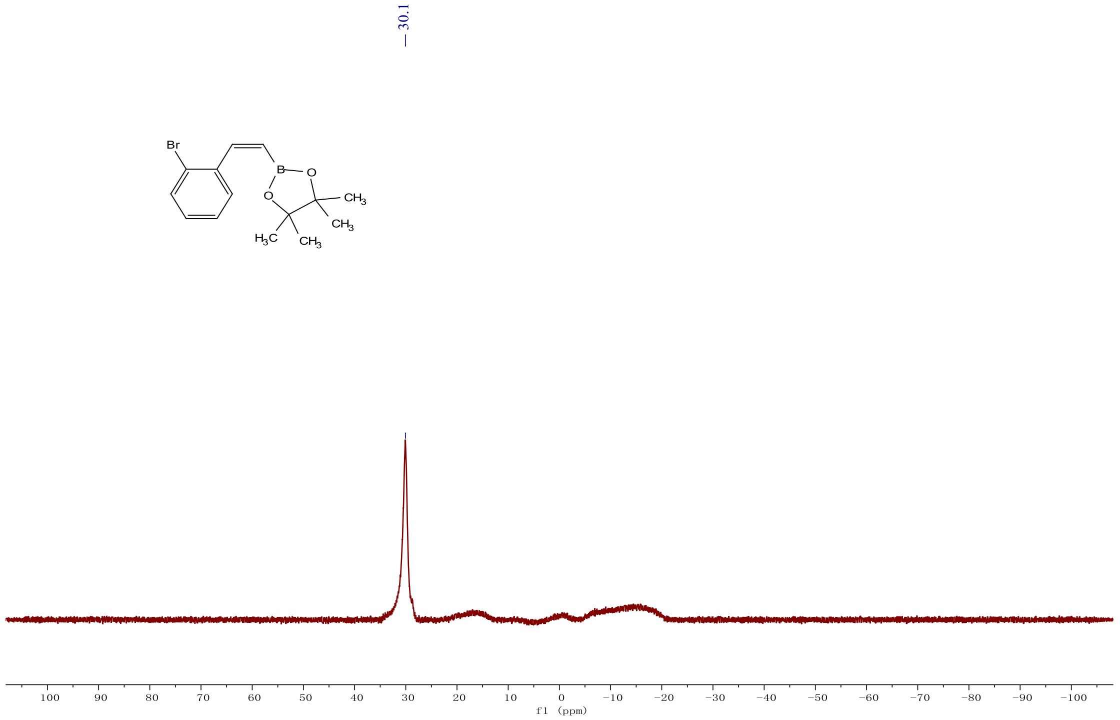

^{1}H NMR (400 MHz, Chloroform-*d*) of 22**b** (*See procedure*)

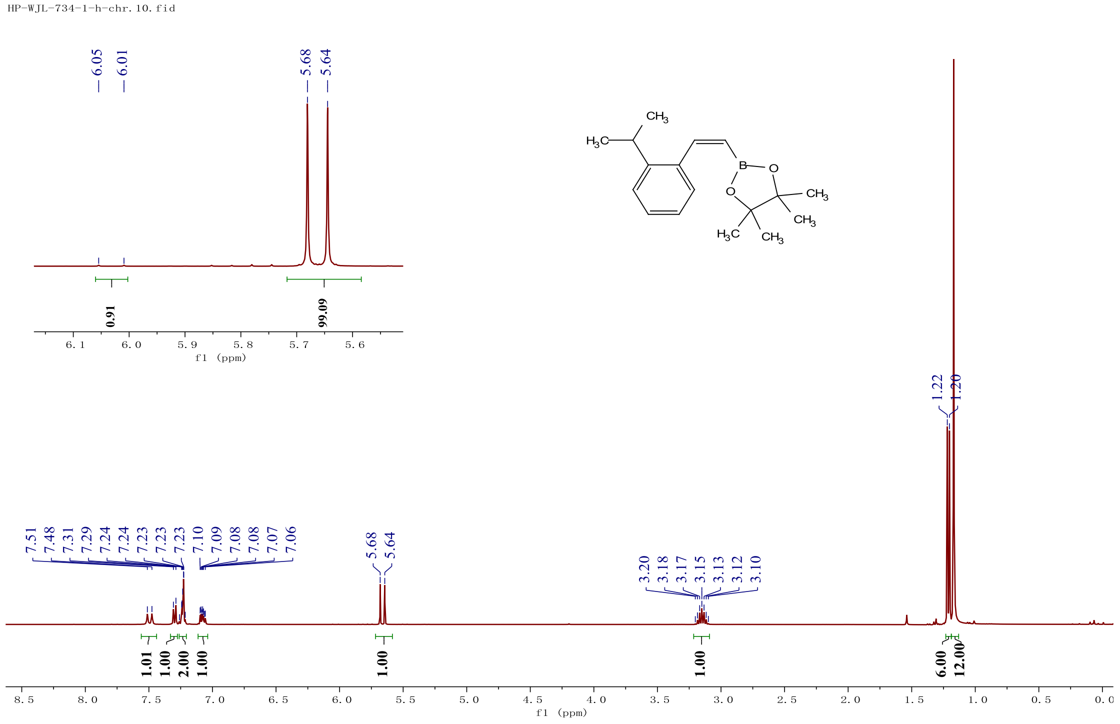

^{13}C NMR (101 MHz, Chloroform-*d*) of 22**b** (See procedure)

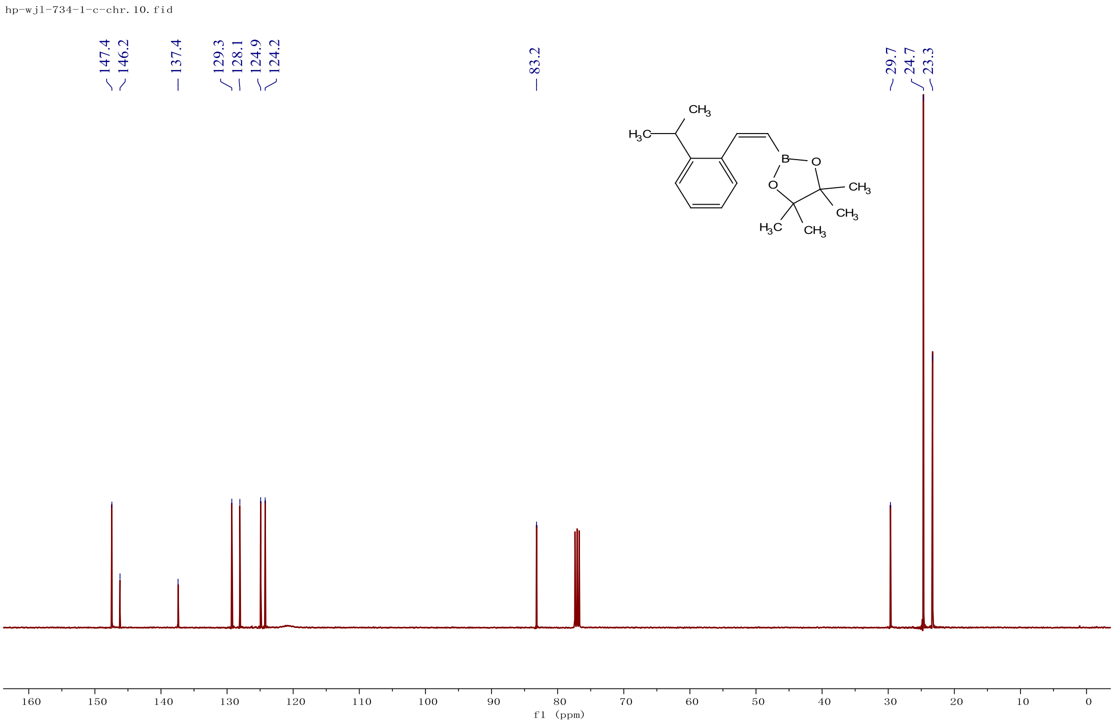

^{11}B NMR (128 MHz, Chloroform-*d*) of 22**b** (See procedure)

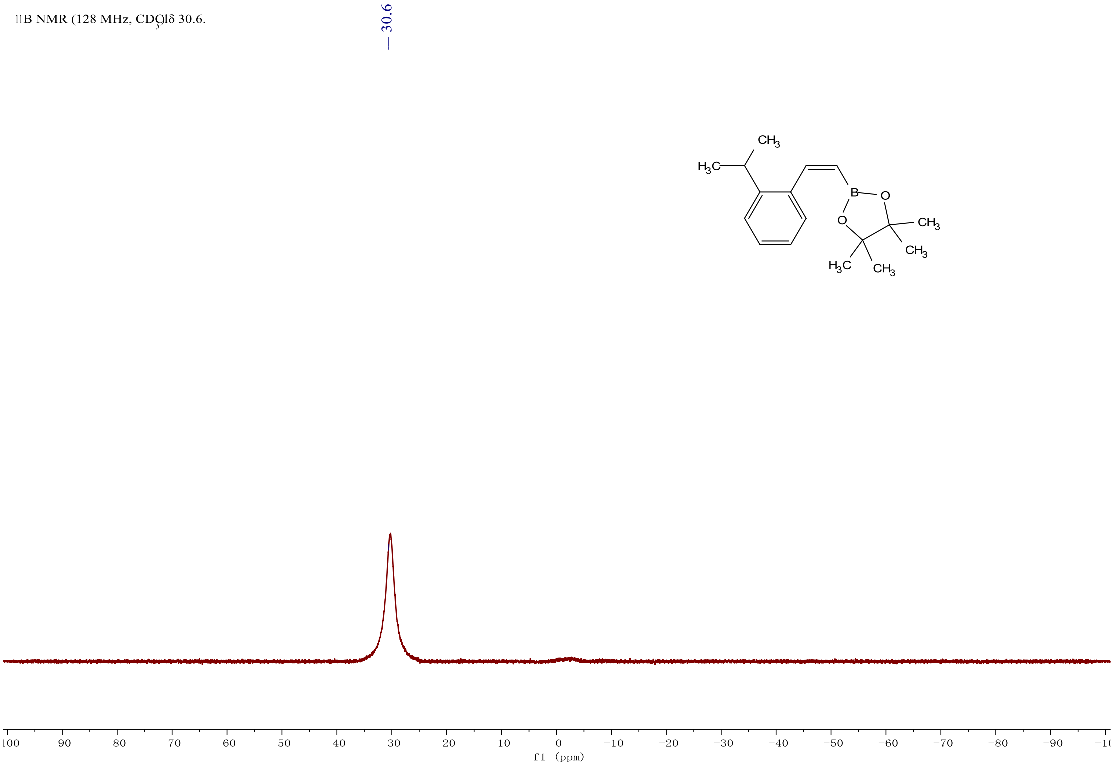

^{1}H NMR (600 MHz, Chloroform-*d*) of 23**b** ([*See procedure*]())

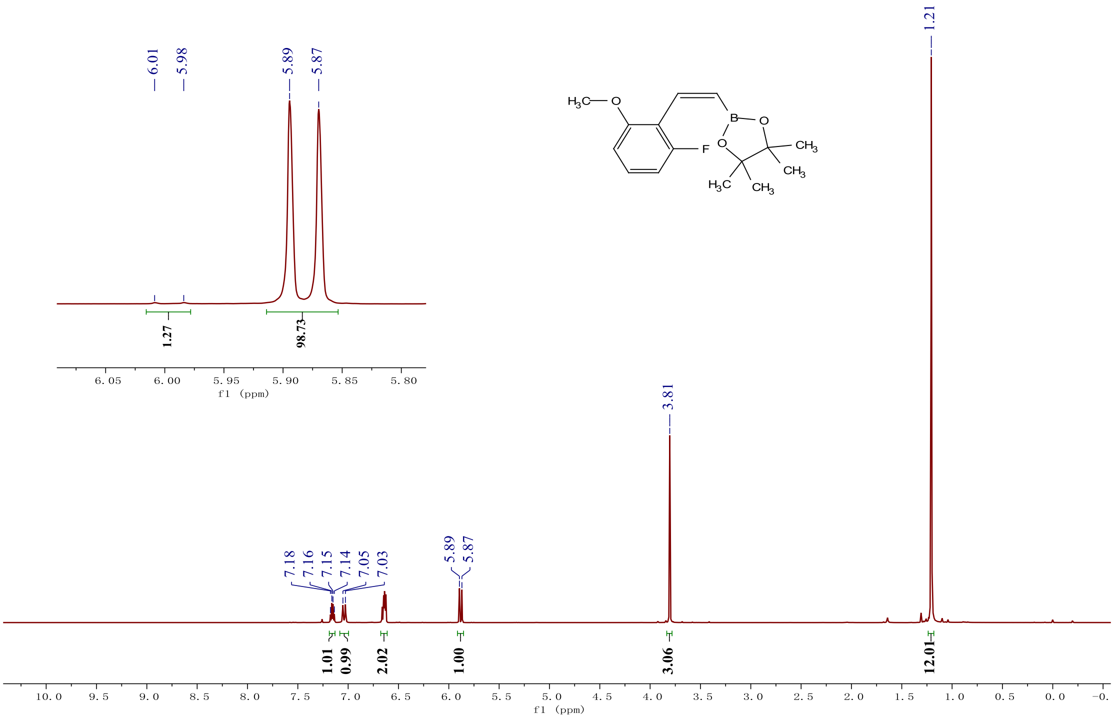

^{13}C NMR (151 MHz, Chloroform-*d*) of 23**b** ([*See procedure*]())

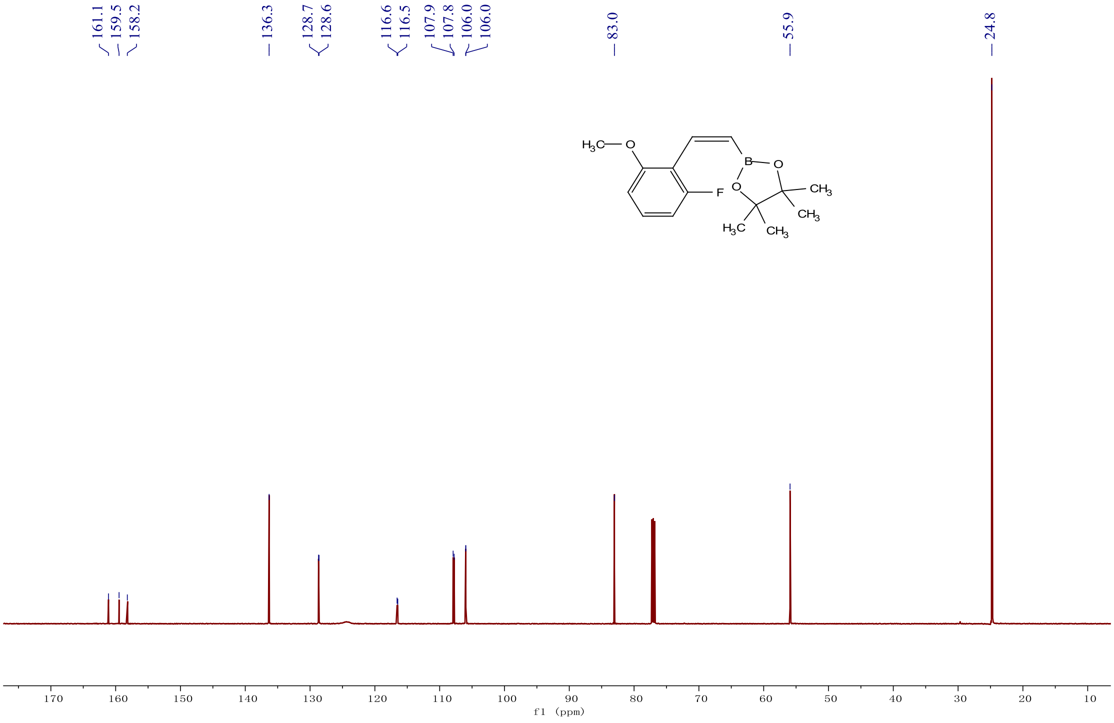

^{11}B NMR (193 MHz, Chloroform-*d*) of 23**b** (*See procedure*)

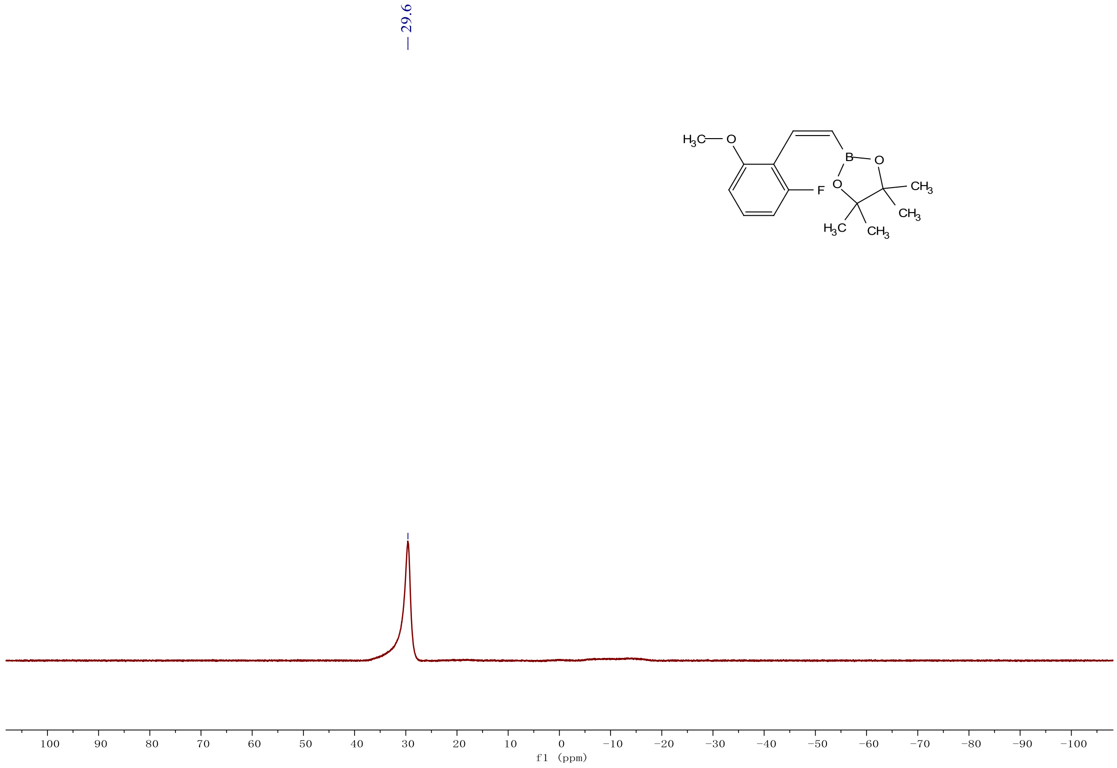

^{19}F NMR (565 MHz, Chloroform-*d*) of 23**b** (*See procedure*)

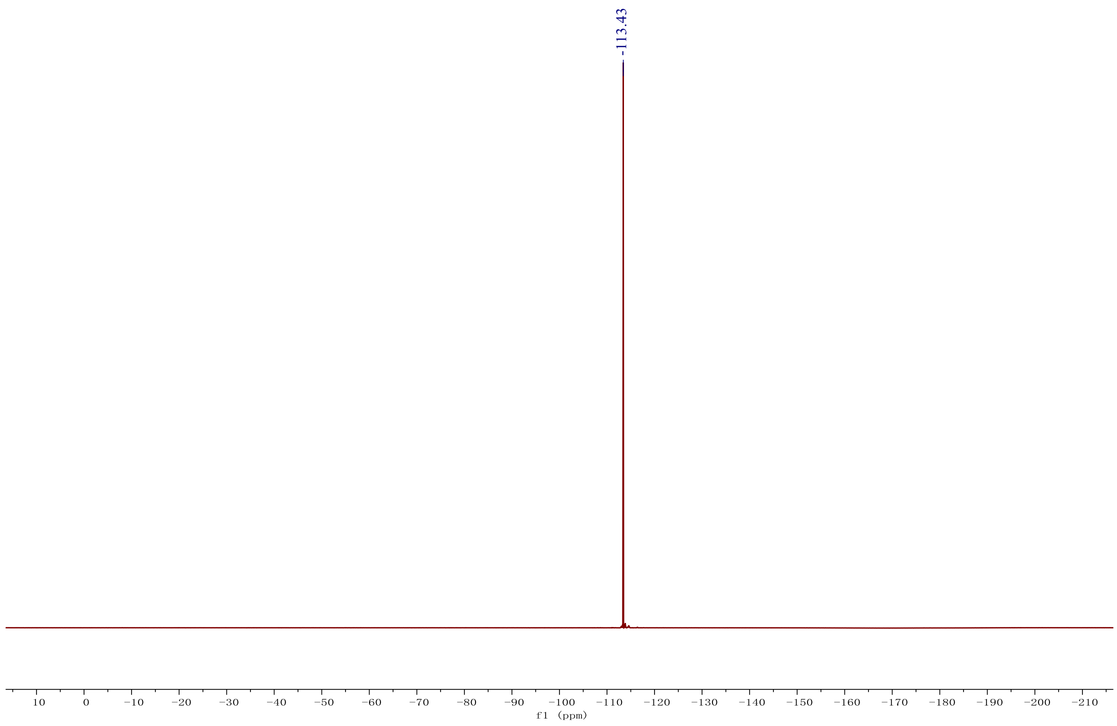

^{1}H NMR (400 MHz, Chloroform-*d*) of 24**b** (*See procedure*)

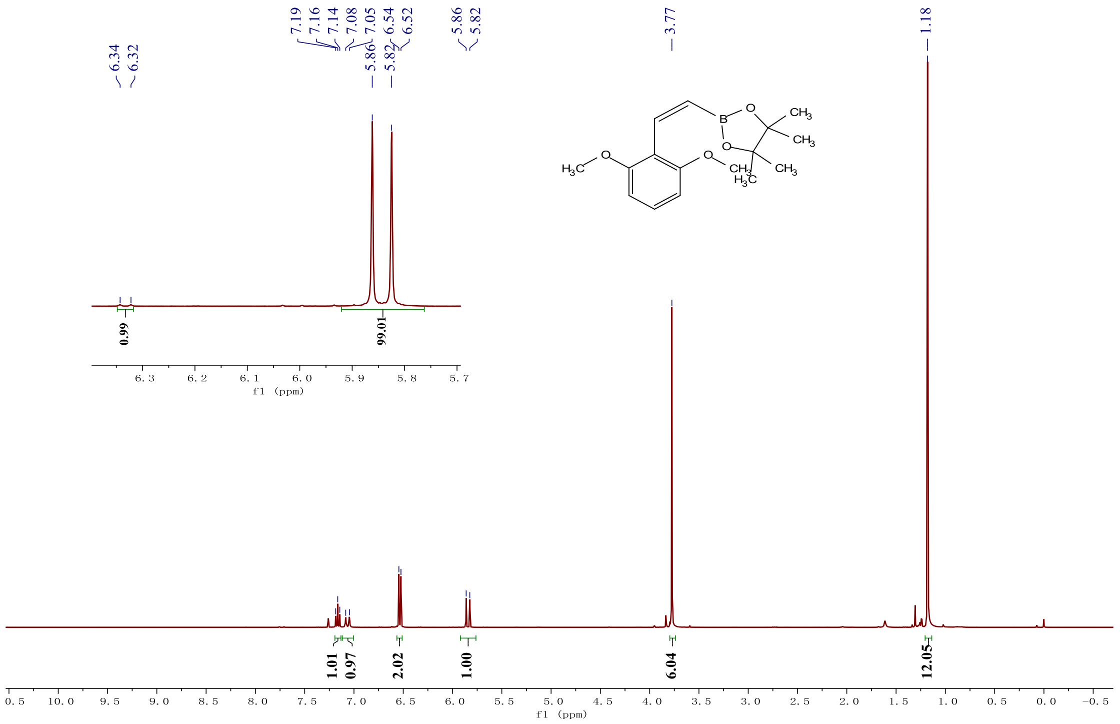

^{13}C NMR (101 MHz, Chloroform-*d*) of 24**b** (*See procedure*)

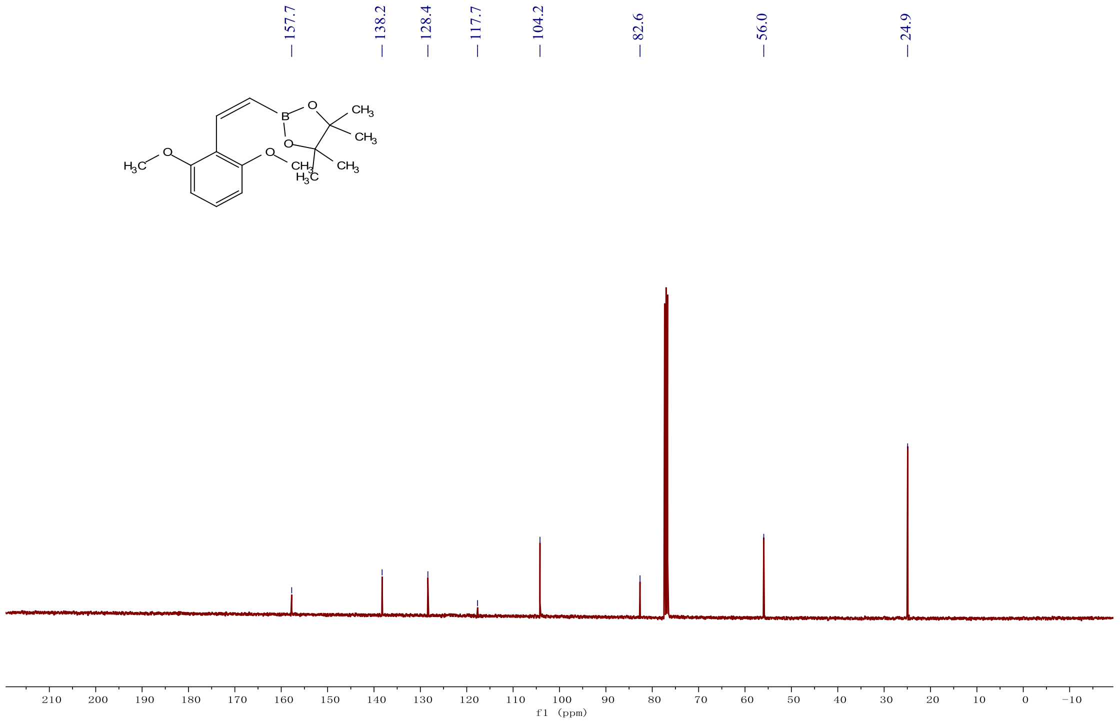

^{11}B NMR (128 MHz, Chloroform-*d*) of 24**b** (*See procedure*)

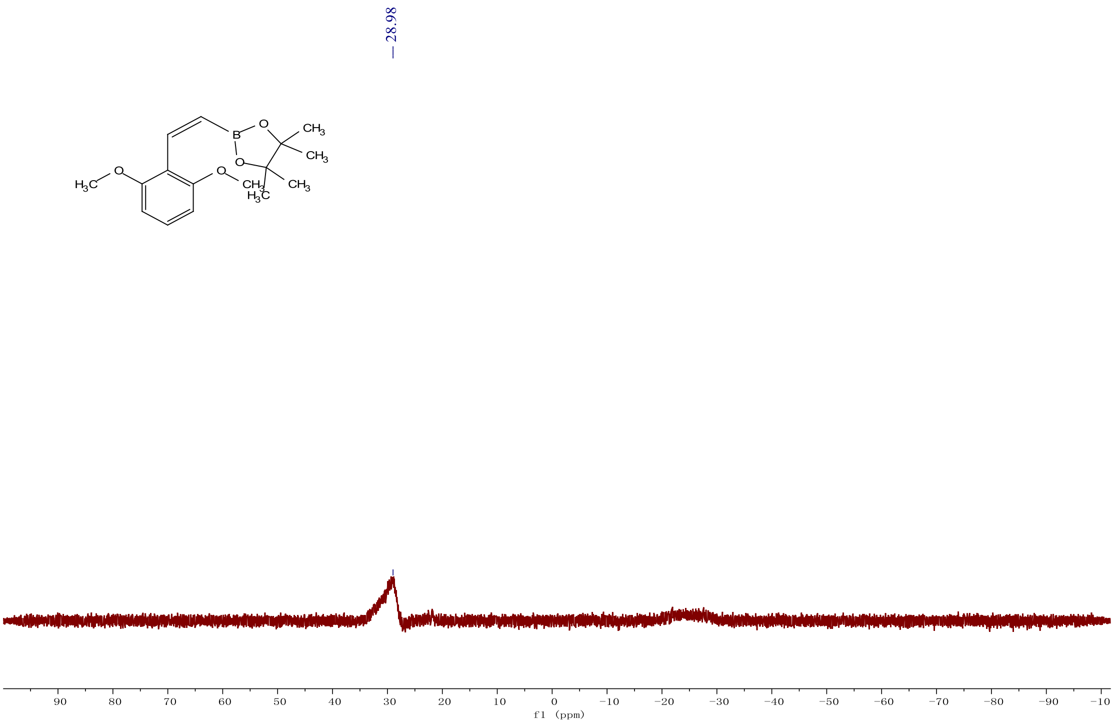

^{1}H NMR (600 MHz, Chloroform-*d*) of 25**b** (*See procedure*)

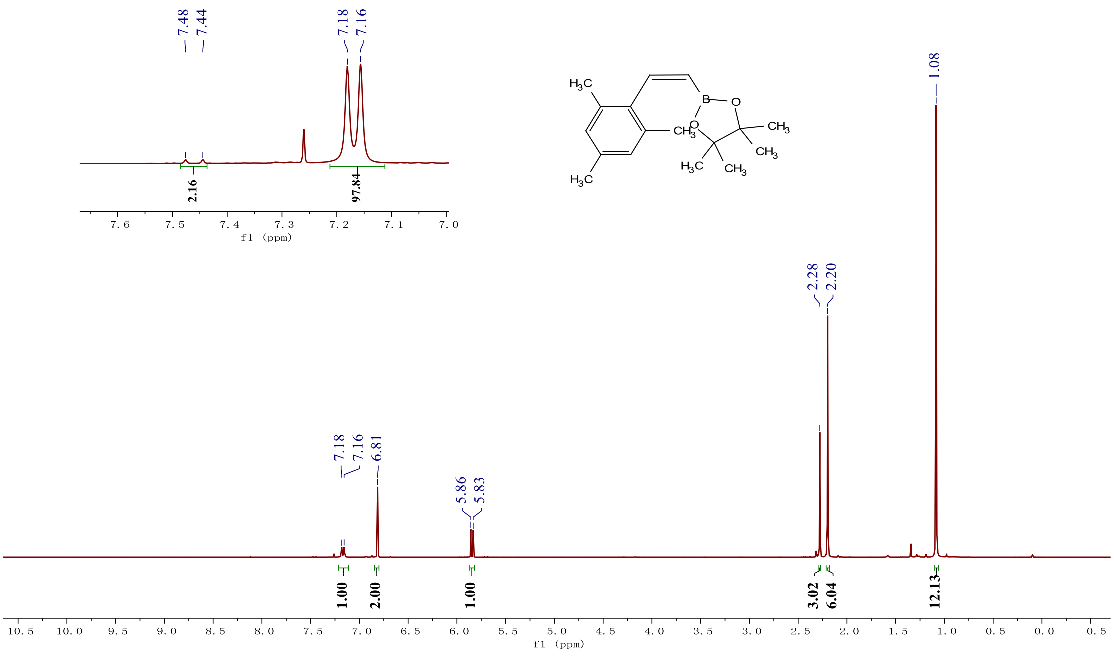

^{13}C NMR (151 MHz, Chloroform-*d*) of 25**b** (See procedure)

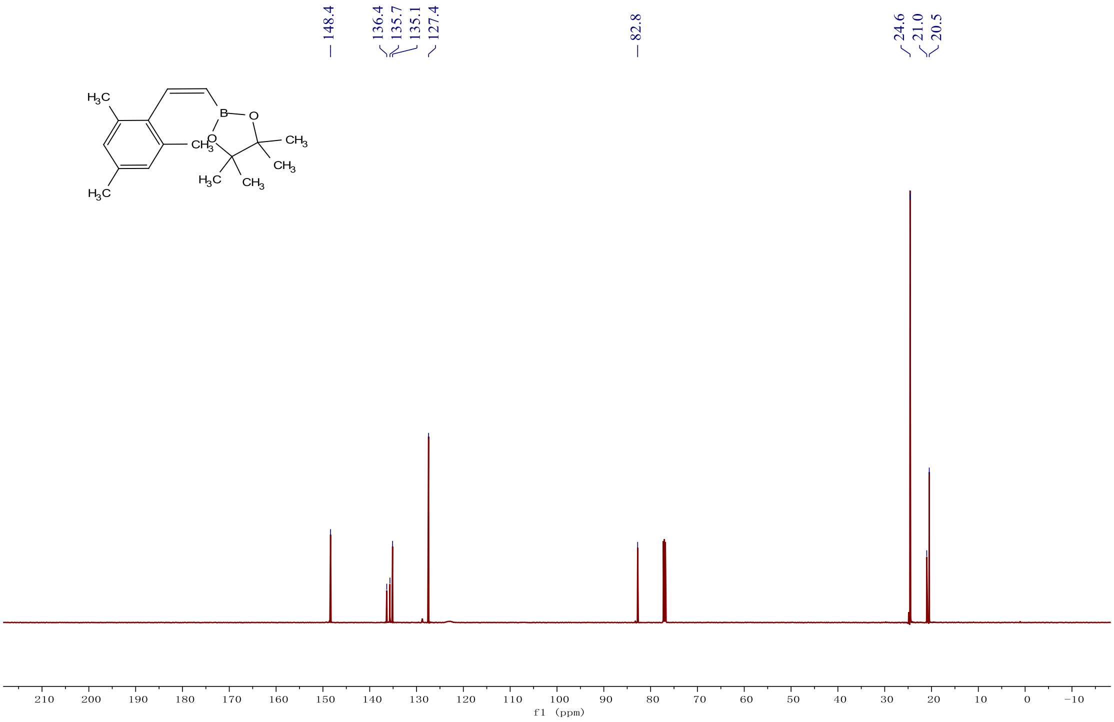

^{11}B NMR (128 MHz, Chloroform-*d*) of 25**b** (See procedure)

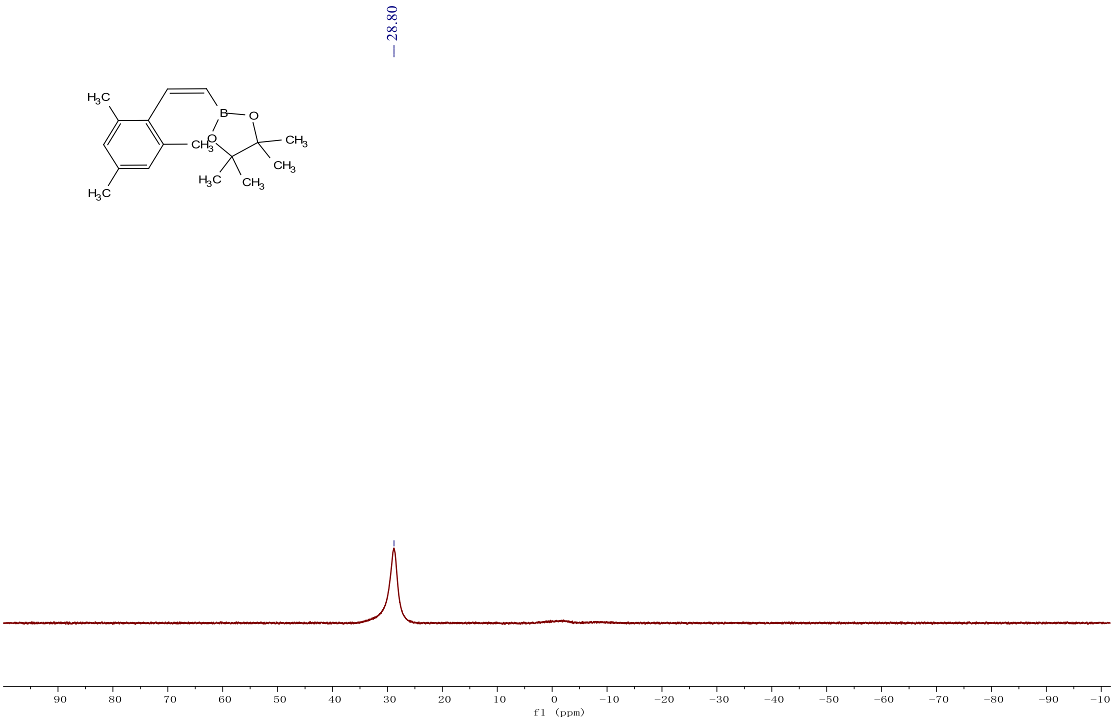

^{1}H NMR (400 MHz, Chloroform-*d*) of 26**b** (See procedure)

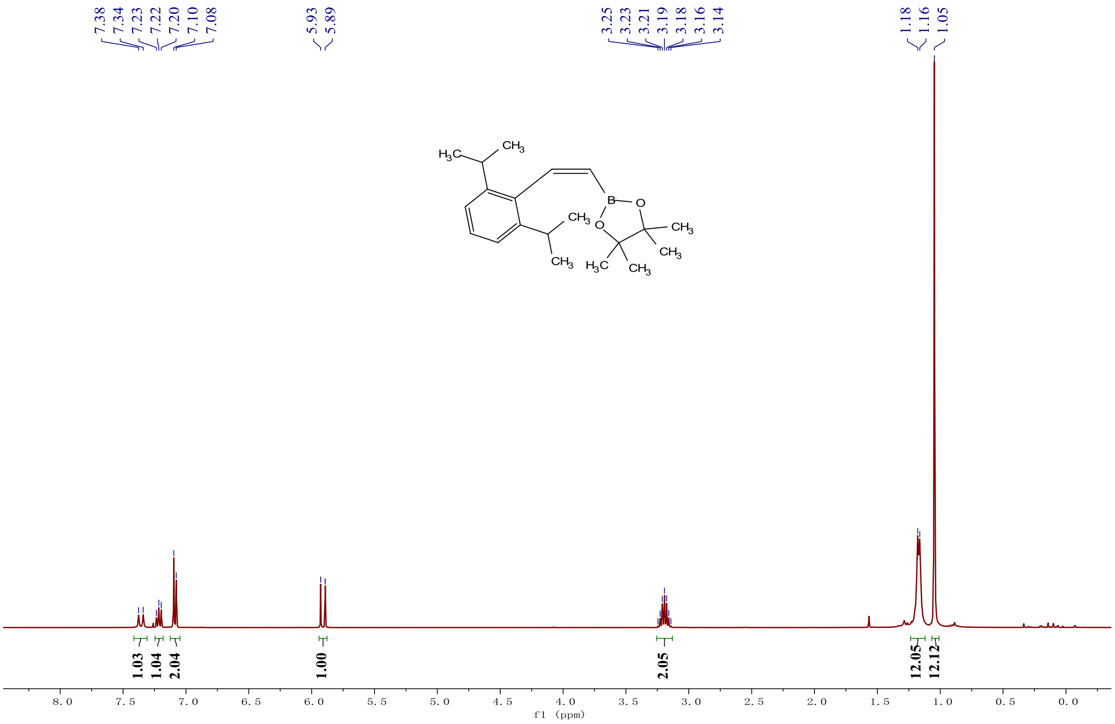

^{13}C NMR (101 MHz, Chloroform-*d*) of 26**b** (See procedure)

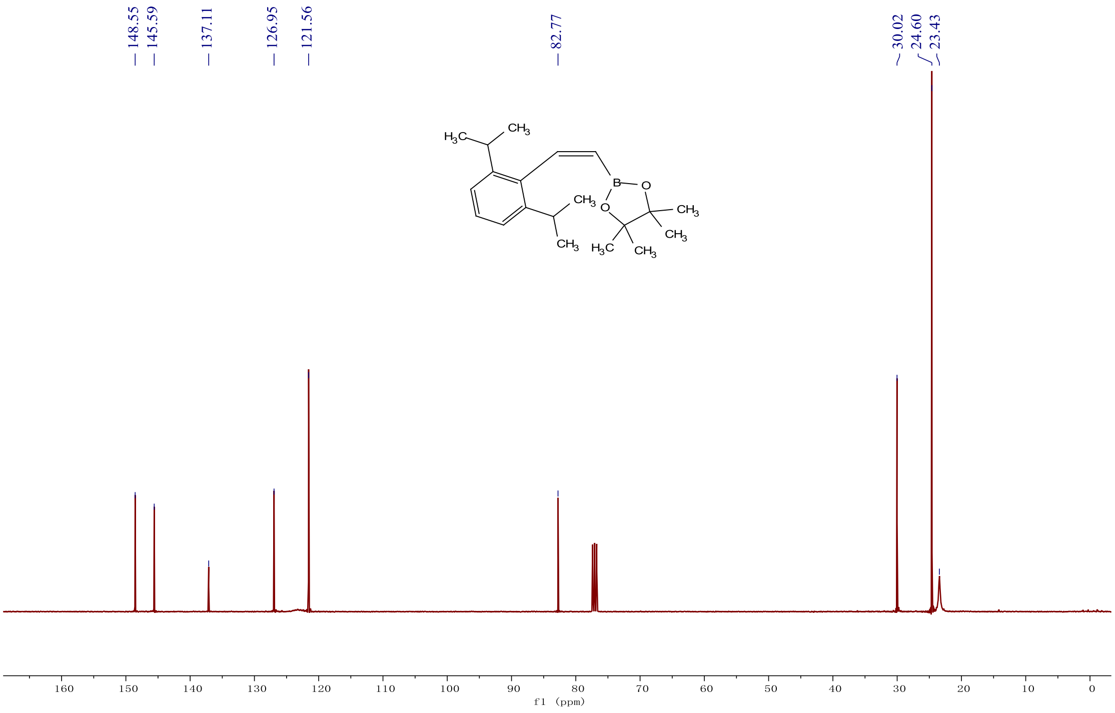

^{11}B NMR (128 MHz, Chloroform-*d*) of 26**b** (*See procedure*)

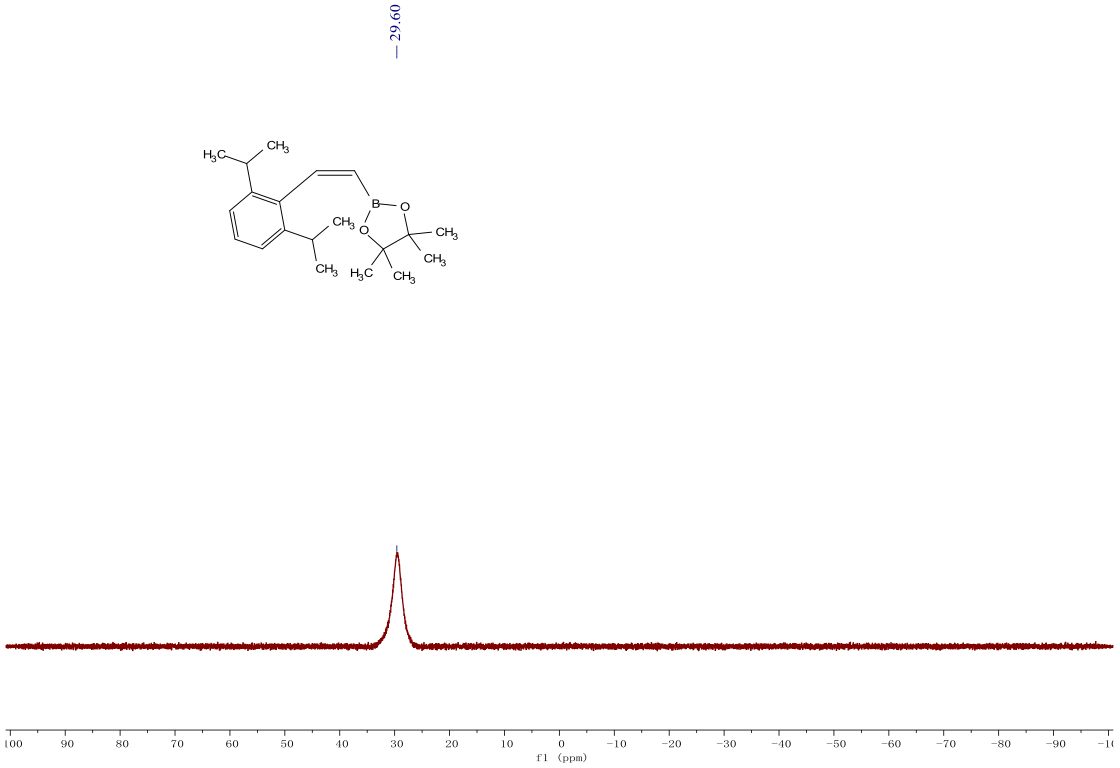

^{1}H NMR (400 MHz, Chloroform-*d*) of 27**b** (*See procedure*)

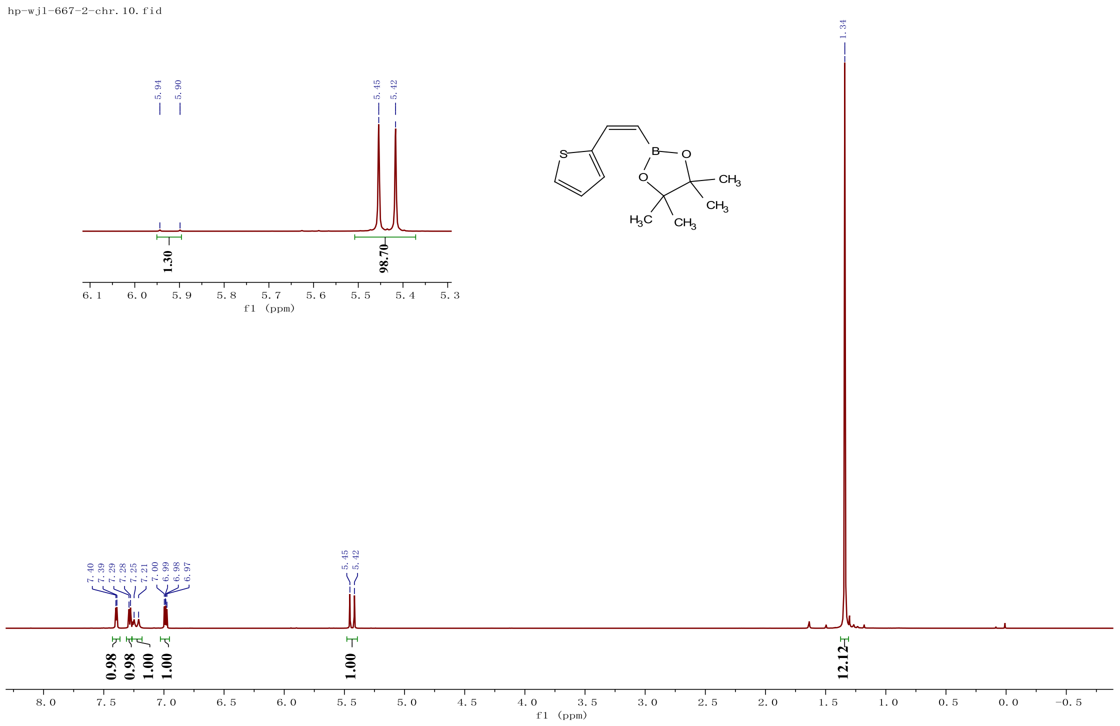

^{1}H NMR (400 MHz, Chloroform-*d*) of 28**b** (<u>*See procedure*</u>)

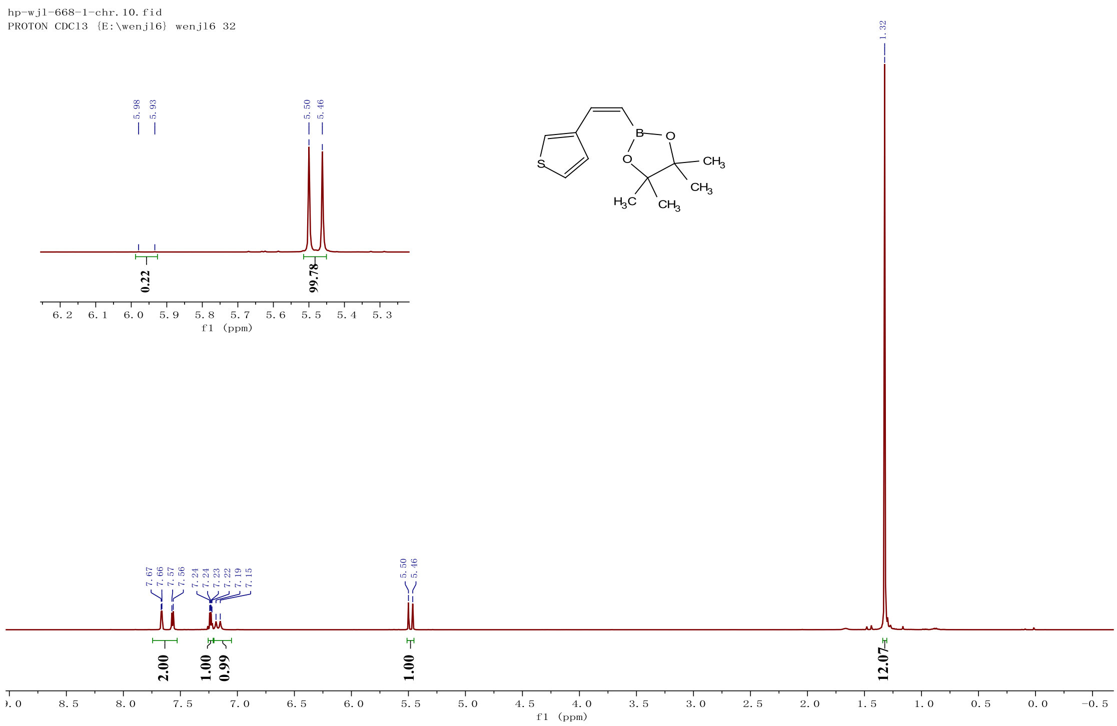

^{13}C NMR (151 MHz, Chloroform-*d*) of 28**b** (<u>*See procedure*</u>)

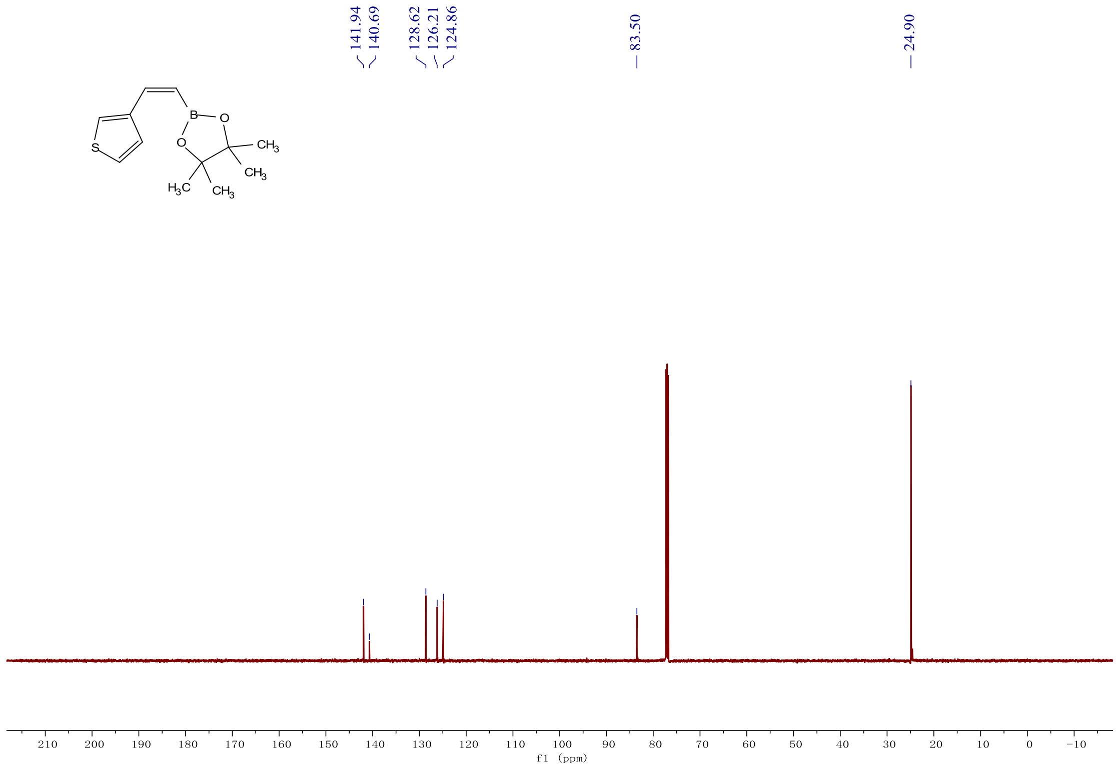

^{11}B NMR (193 MHz, Chloroform-*d*) of 28**b** (*See procedure*)

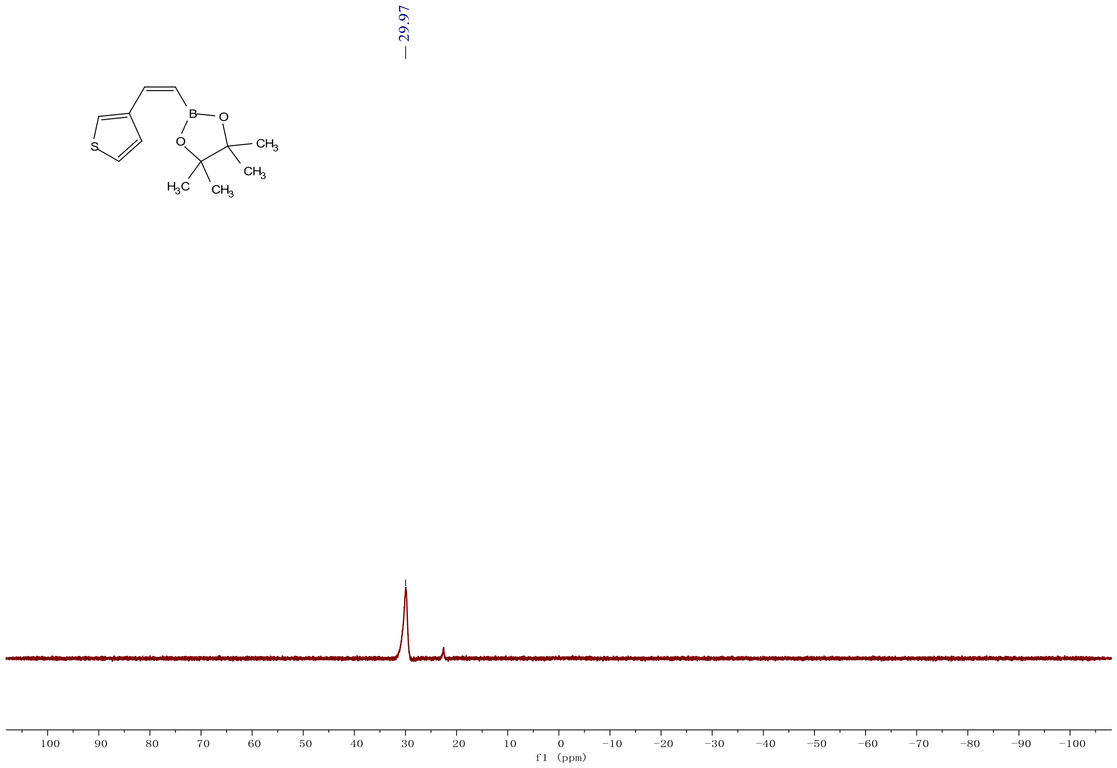

^{1}H NMR (600 MHz, Chloroform-*d*) of 29**b** (*See procedure*)

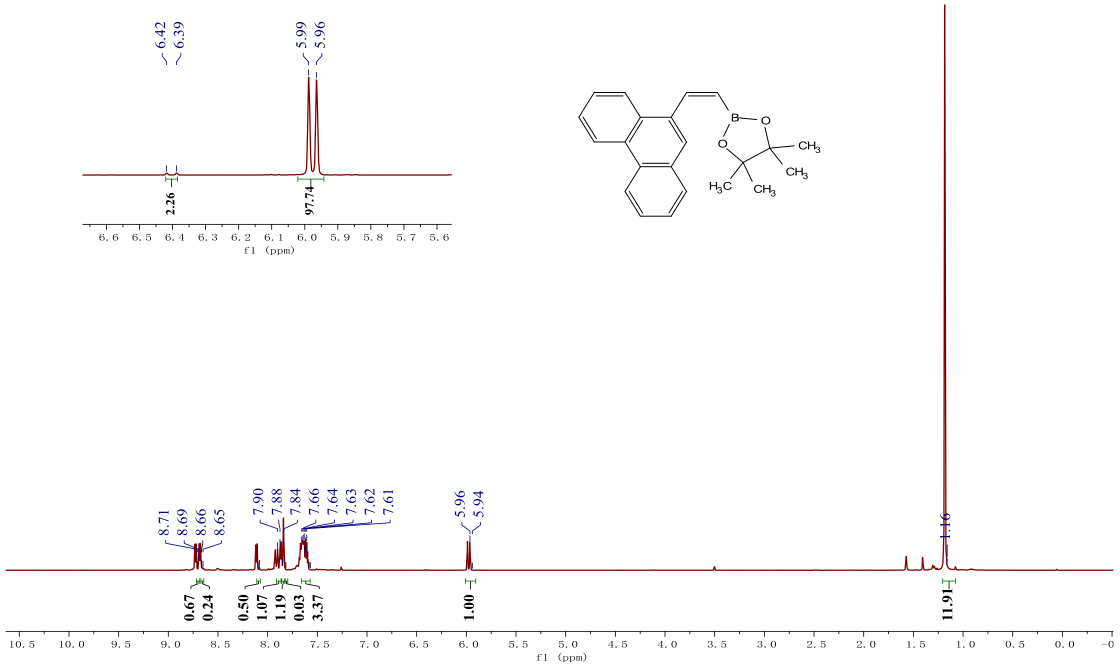

^{13}C NMR (151 MHz, Chloroform-*d*) of 29**b** (*See procedure*)

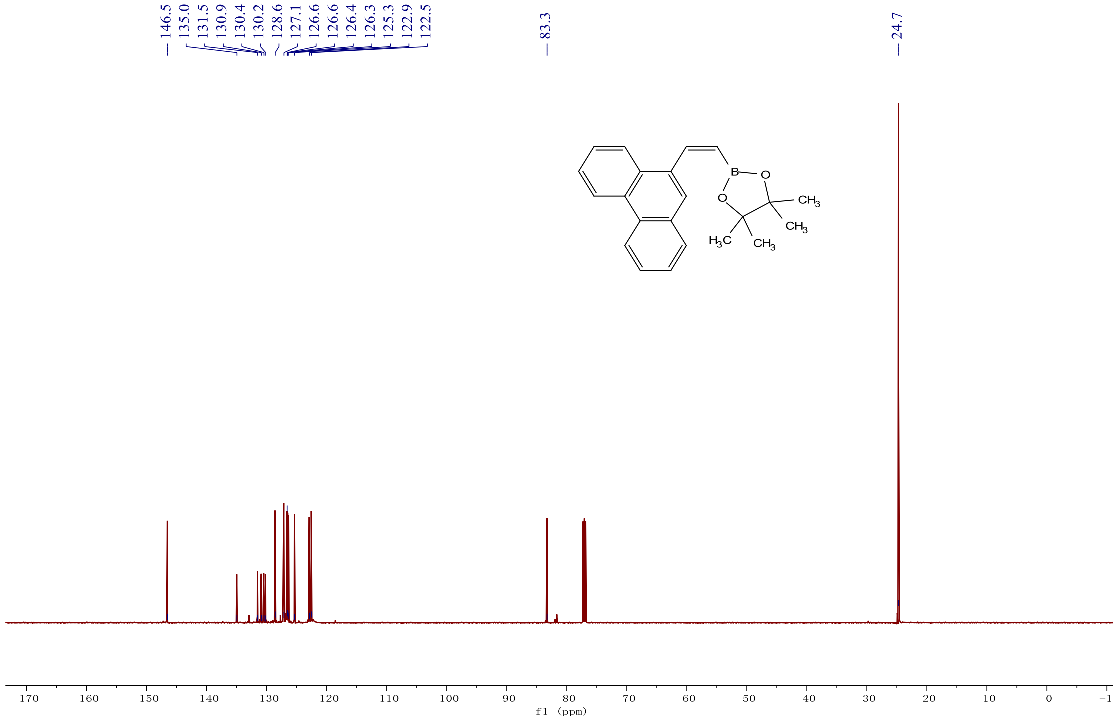

^{11}B NMR (193 MHz, Chloroform-*d*) of 29**b** (*See procedure*)

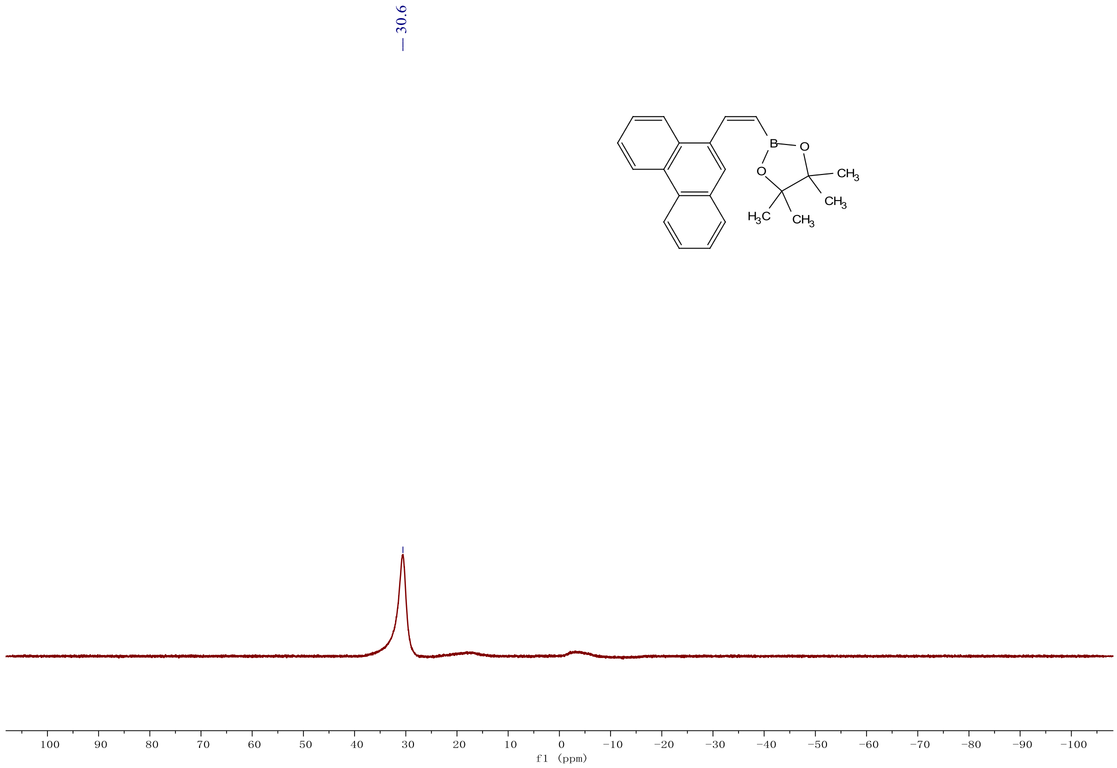

^{1}H NMR (400 MHz, Chloroform-*d*) of 30**b** (*See procedure*)

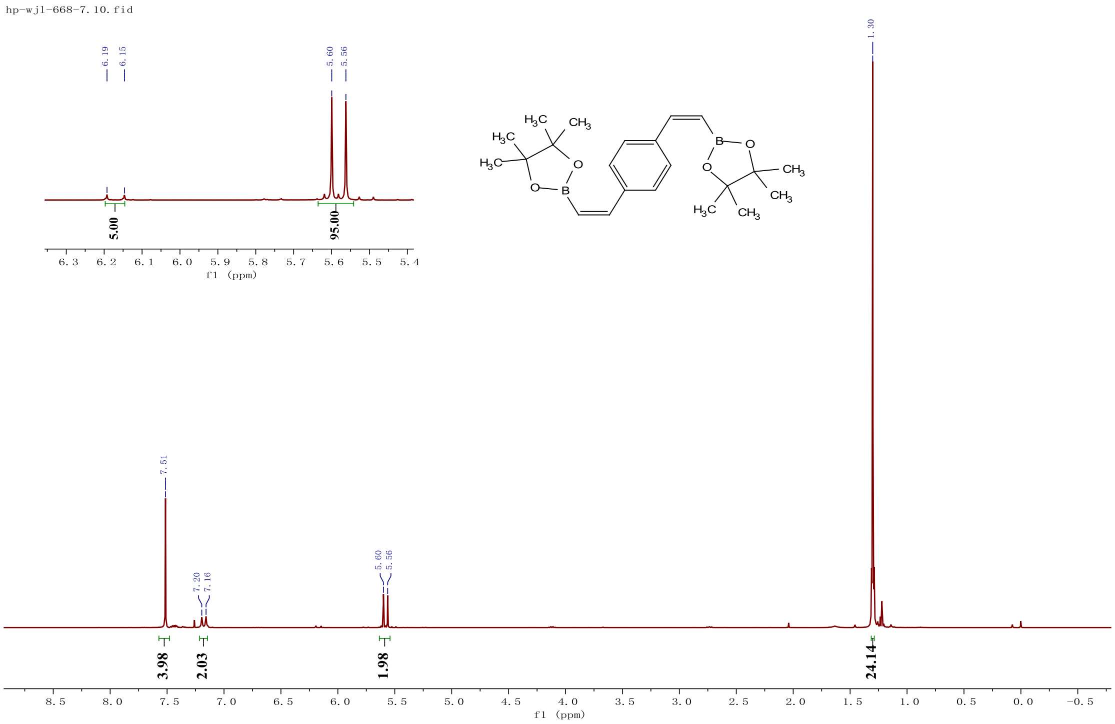

^{1}H NMR (400 MHz, Chloroform-*d*) of 31**b** (*See procedure*)

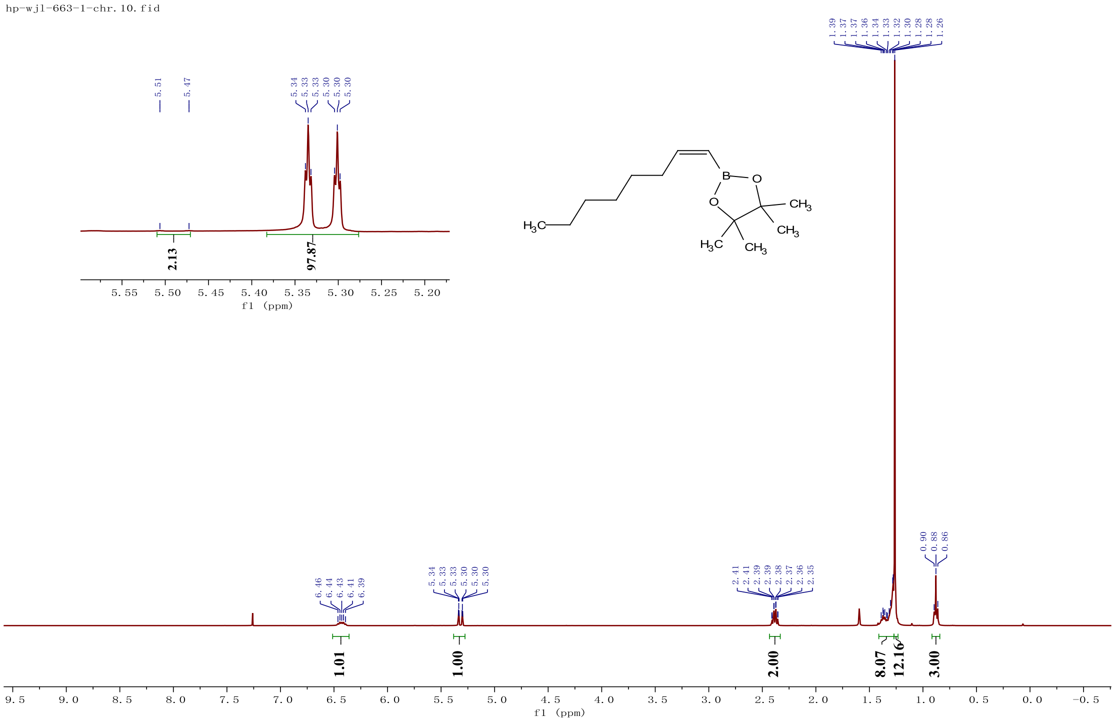

^{11}B NMR (193 MHz, Chloroform-*d*) of 31**b** (*See procedure*)

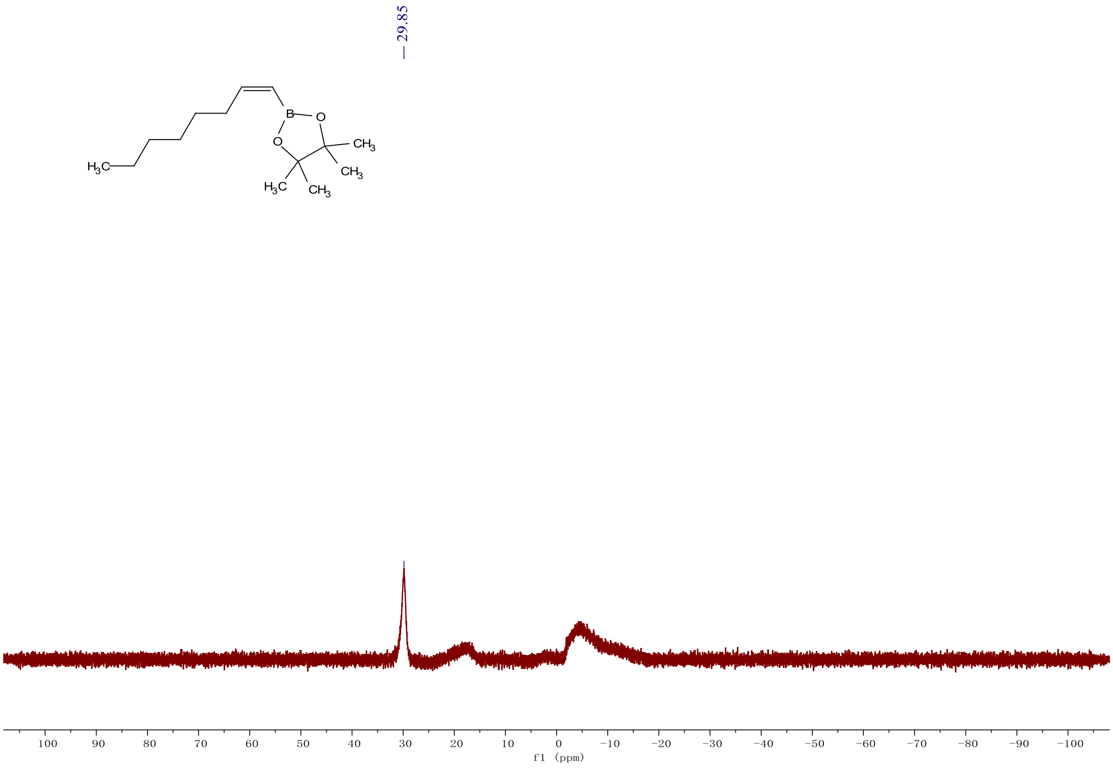

^{1}H NMR (400 MHz, Chloroform-*d*) of 32**b** (*See procedure*)

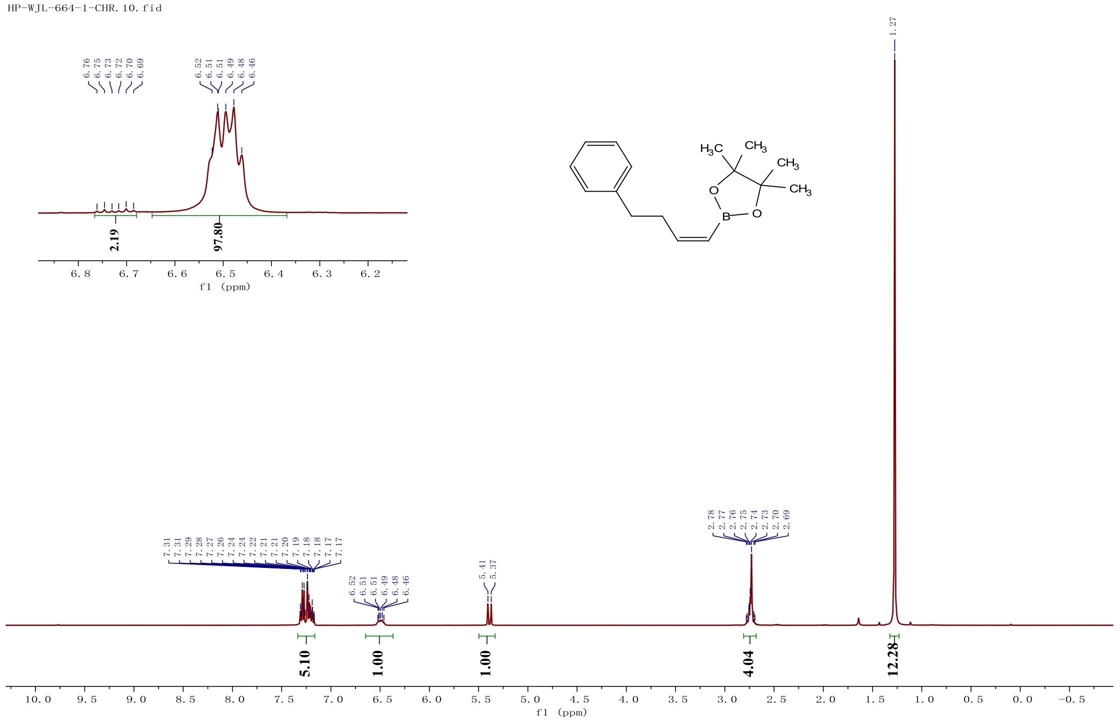

^{1}H NMR (400 MHz, Chloroform-*d*) of 33**b** (*See procedure*)

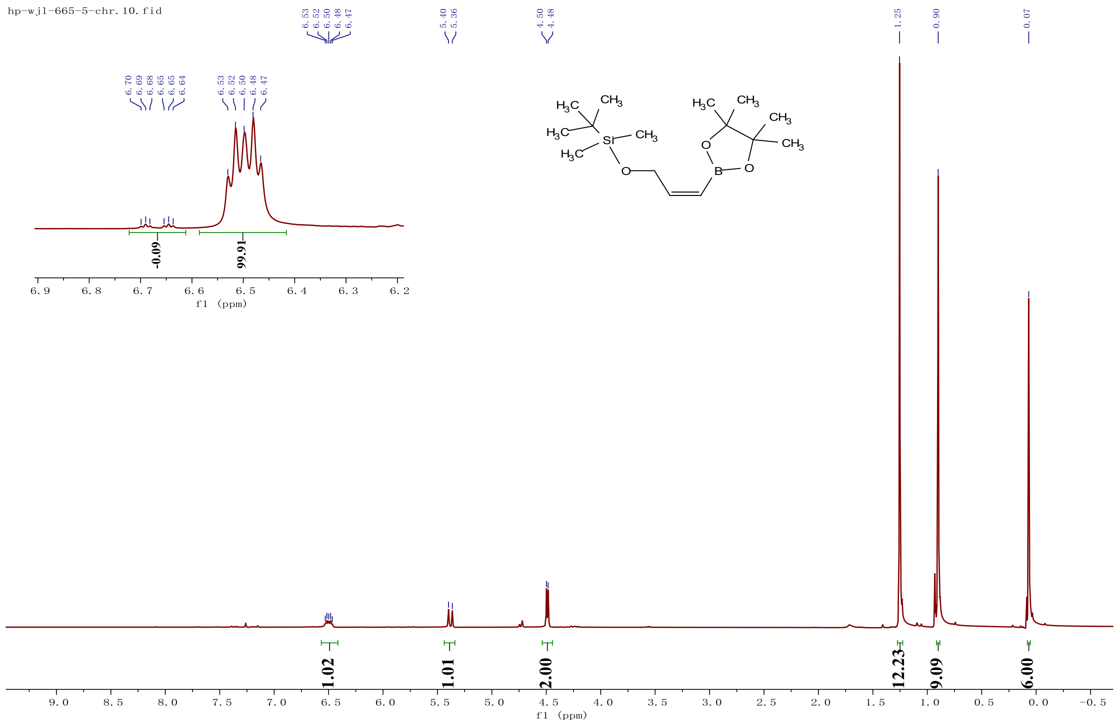

^{1}H NMR (400 MHz, Chloroform-*d*) of 34**b** (*See procedure*)

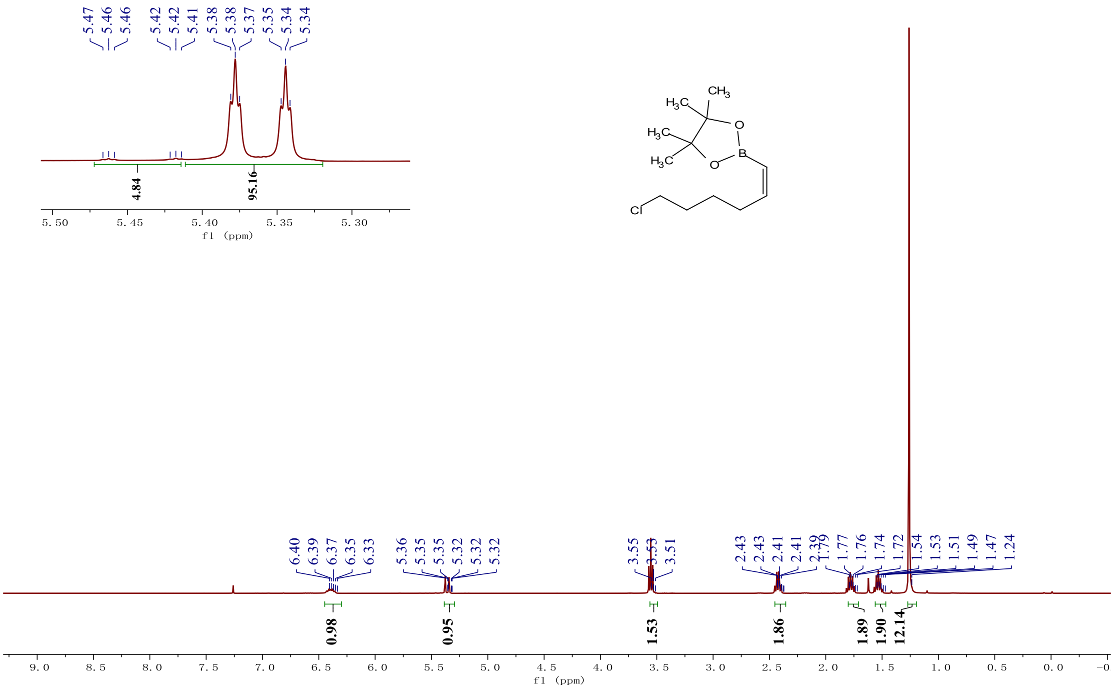

^{13}C NMR (101 MHz, Chloroform-*d*) of 34**b** (*See procedure*)

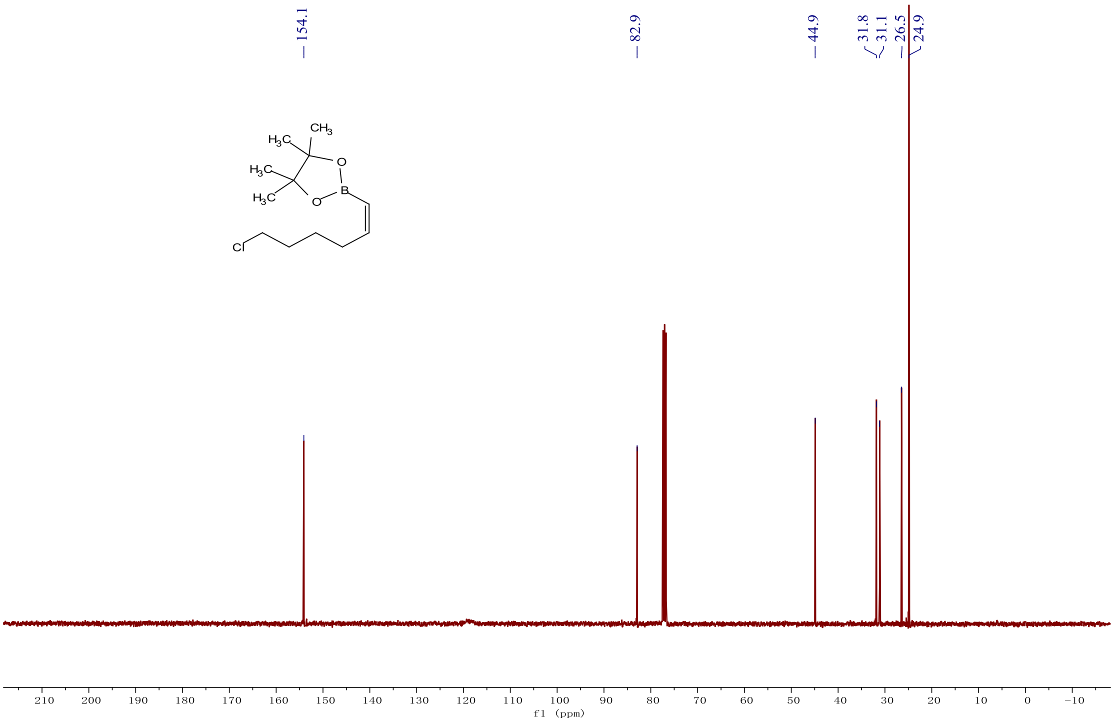

^{11}B NMR (128 MHz, Chloroform-*d*) of 34**b** (See procedure)

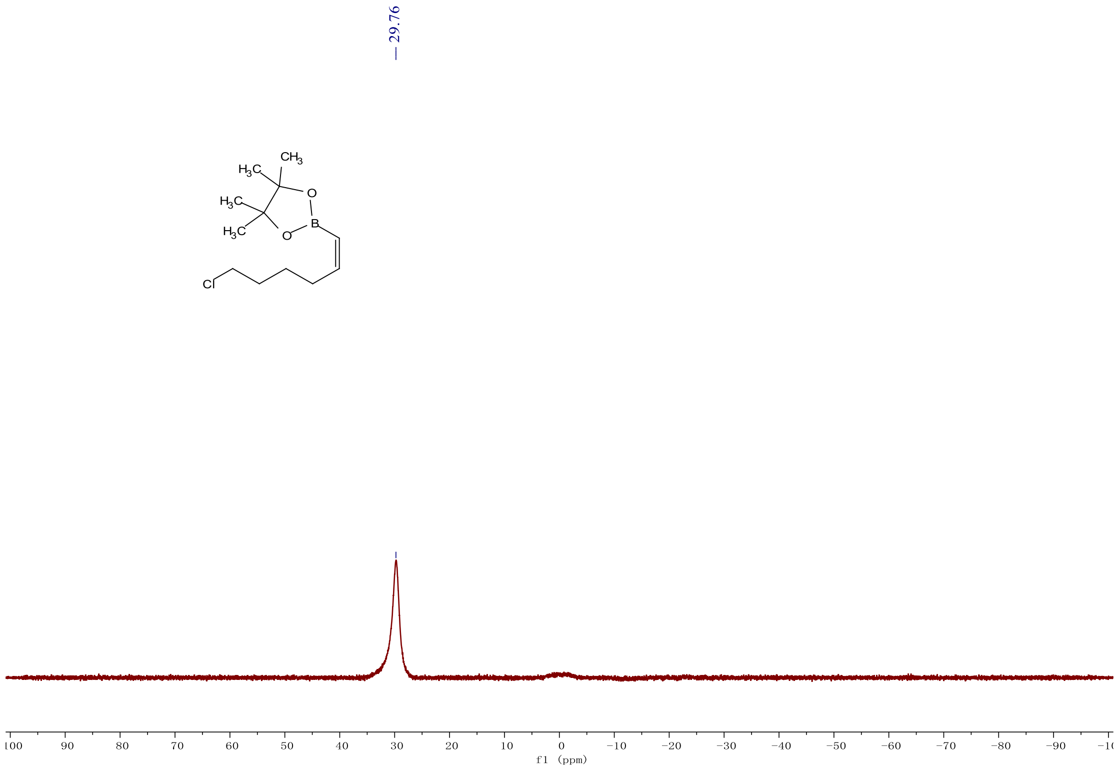

^{1}H NMR (400 MHz, Chloroform-*d*) of 35**b** (See procedure)

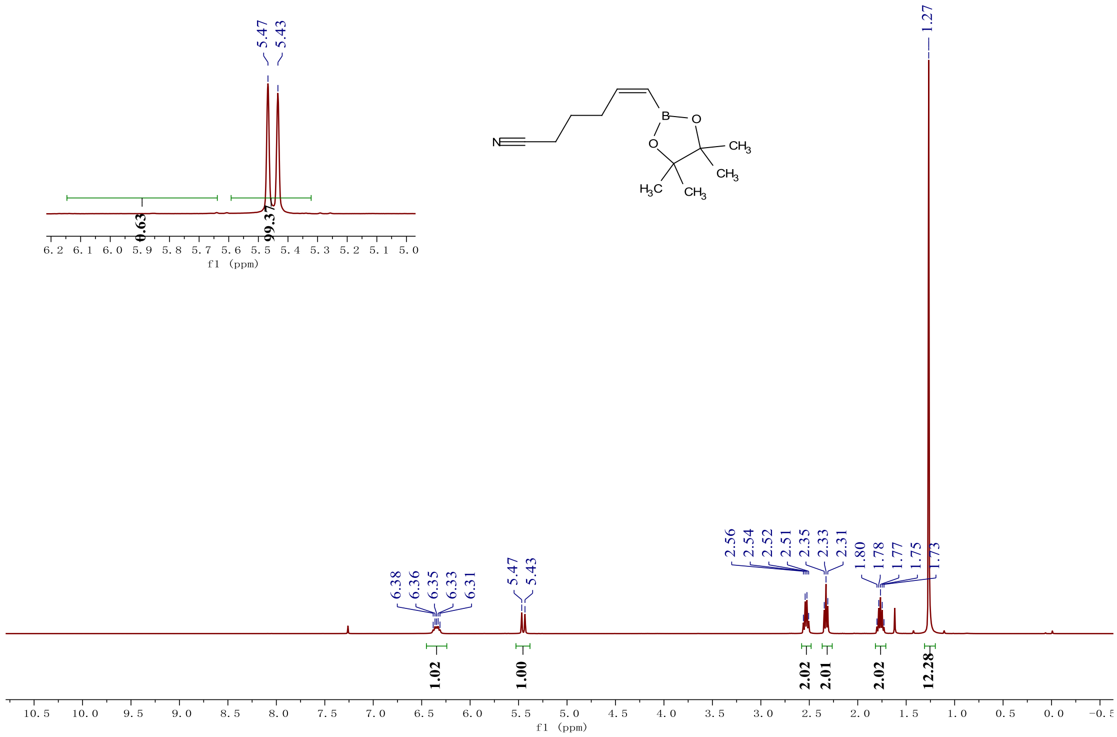

^{13}C NMR (101 MHz, Chloroform-*d*) of 35**b** (See procedure)

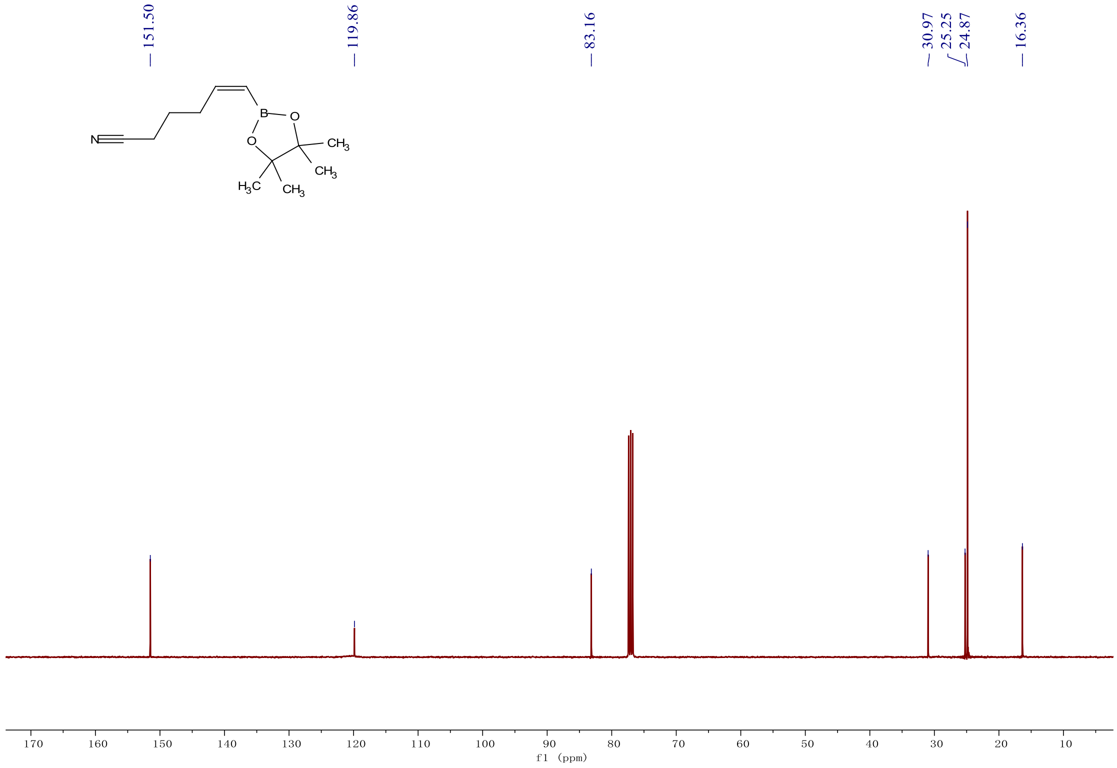

^{11}B NMR (193 MHz, Chloroform-*d*) of 35**b** (See procedure)

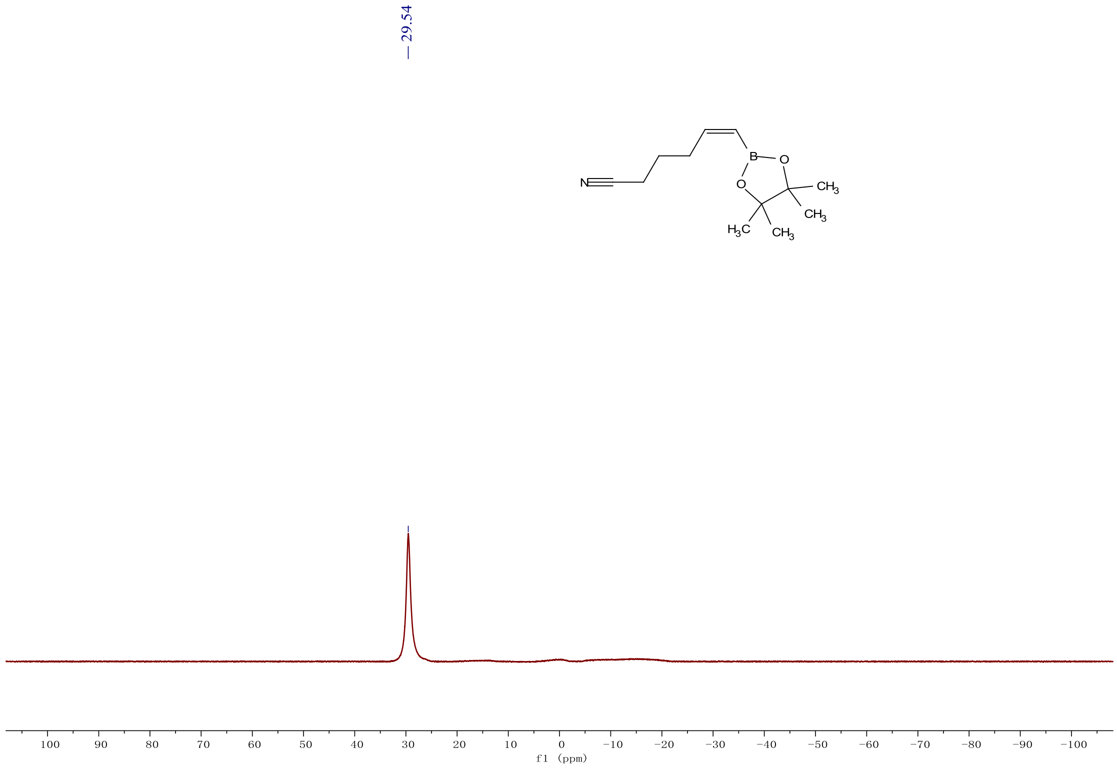

^{1}H NMR (600 MHz, Chloroform-*d*) of 36**b** (*See procedure*)

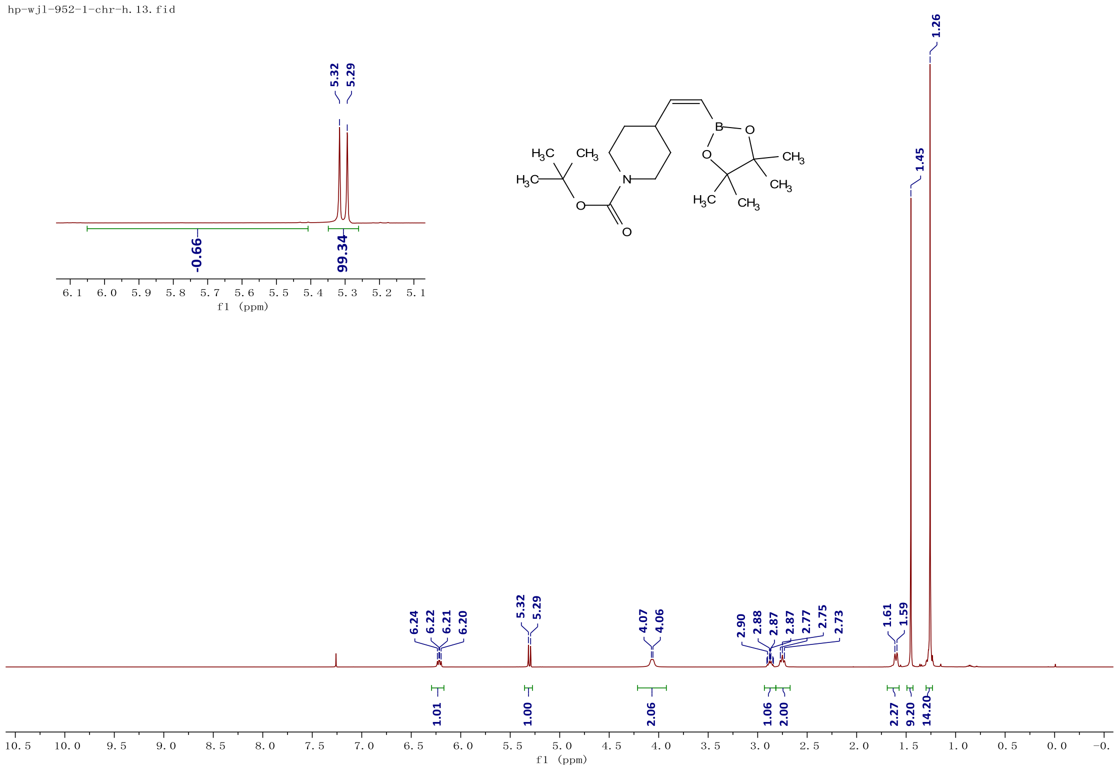

^{13}C NMR (151 MHz, Chloroform-*d*) of 36**b** (*See procedure*)

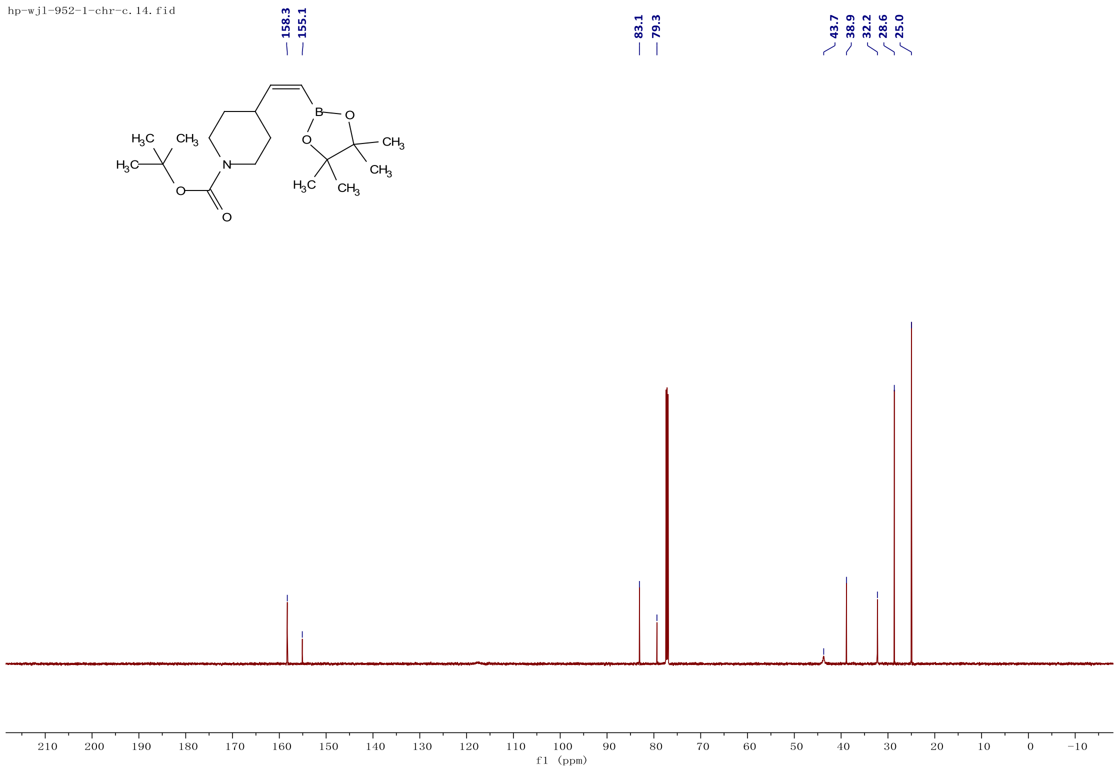

^{11}B NMR (193 MHz, Chloroform-*d*) of 36**b** (*See procedure*)

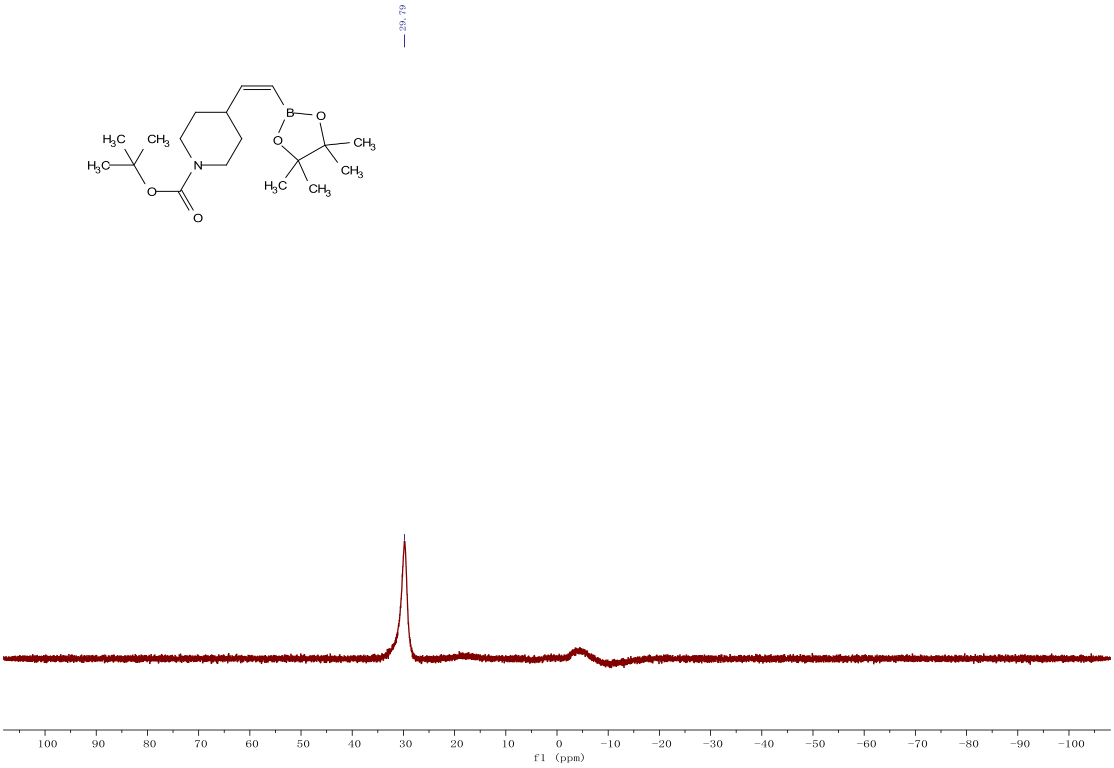

^{1}H NMR (400 MHz, Chloroform-*d*) of 37**b** (*See procedure*)

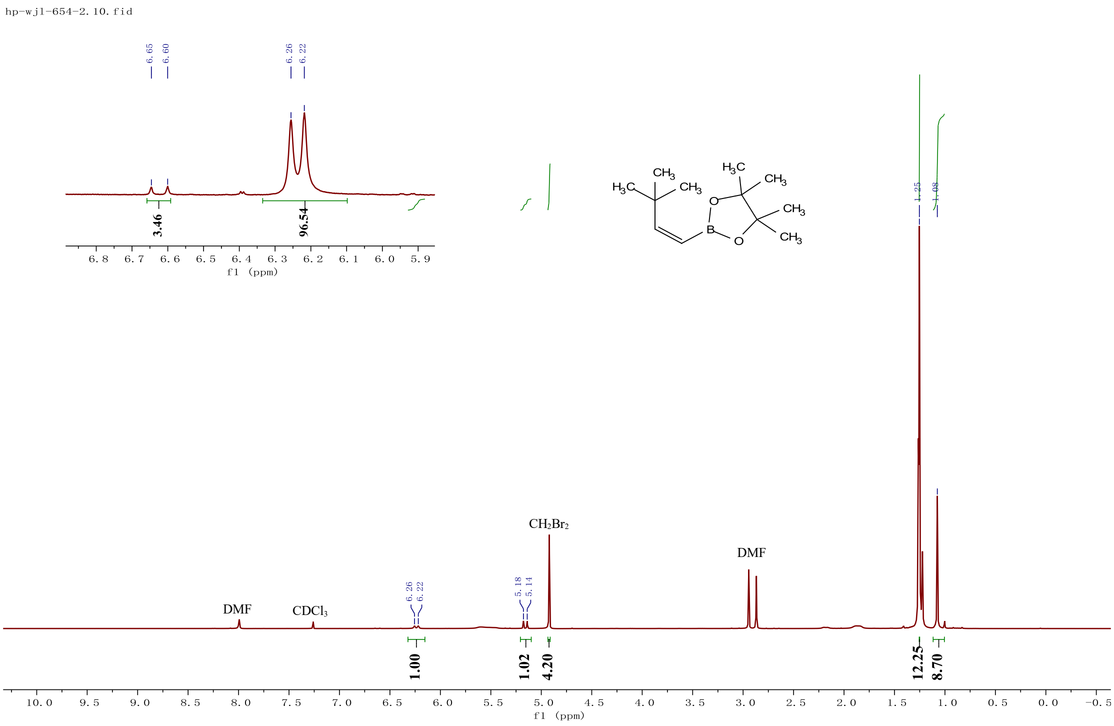

^{1}H NMR (400 MHz, Chloroform-*d*) of 38**b** (*See procedure*)

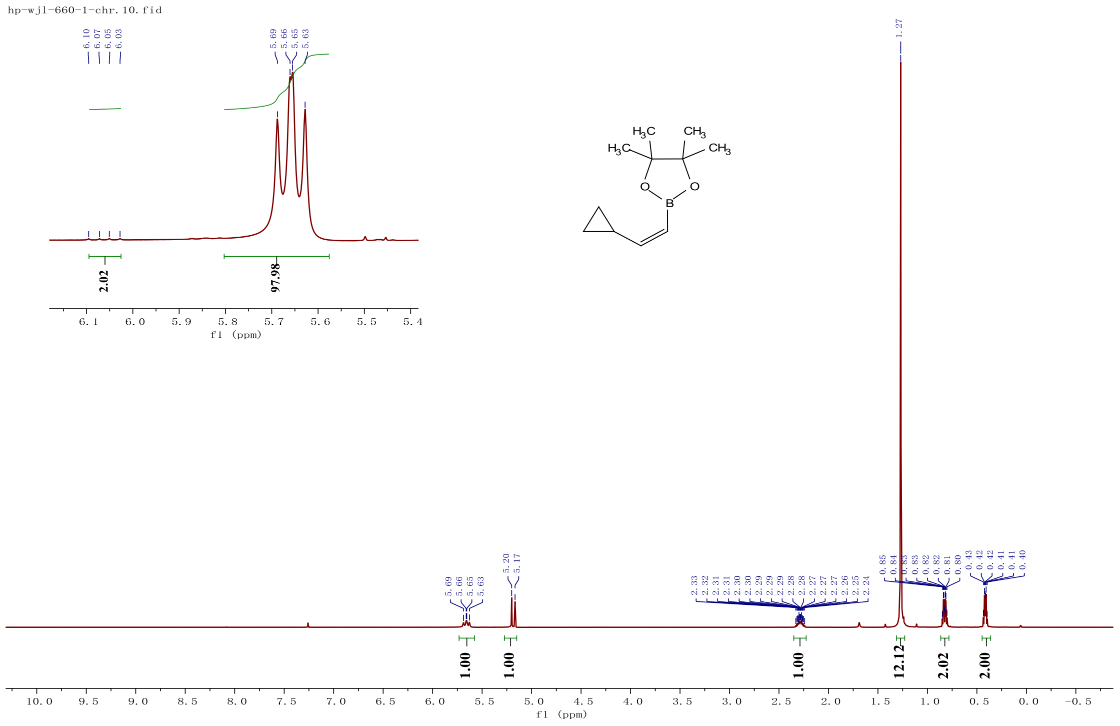

^{1}H NMR (400 MHz, Chloroform-*d*) of 39**b** (*See procedure*)

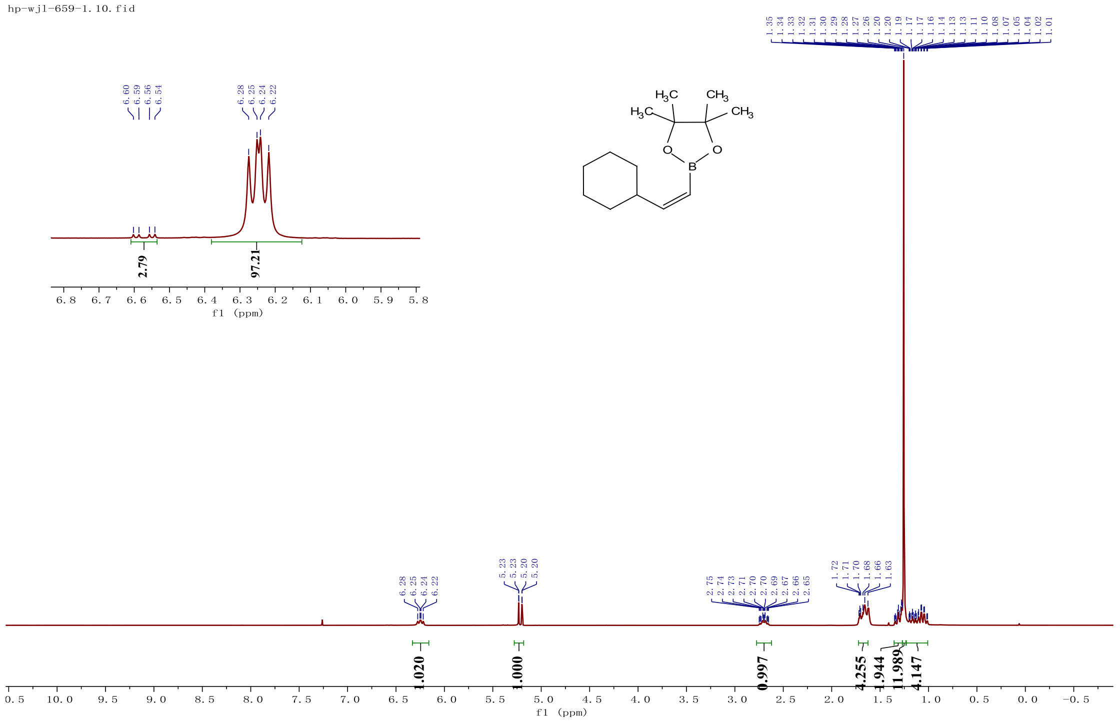

^{1}H NMR (400 MHz, Chloroform-*d*) of 40**b** (*See procedure*)

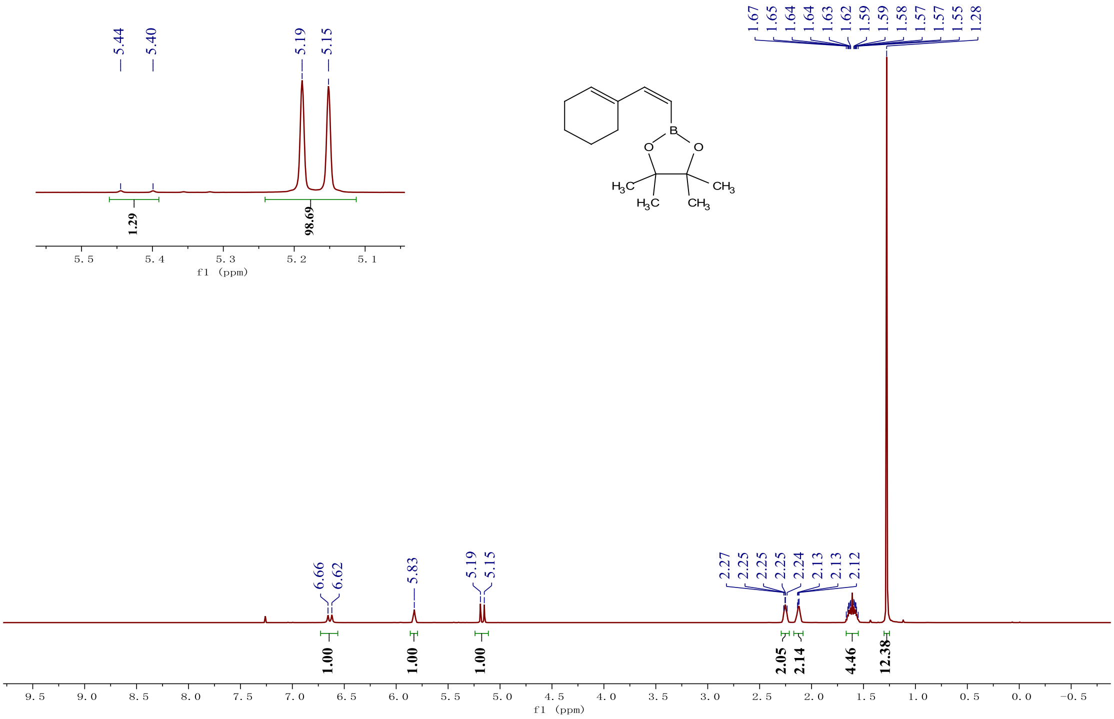

^{13}C NMR (101 MHz, Chloroform-*d*) of 40**b** (*See procedure*)

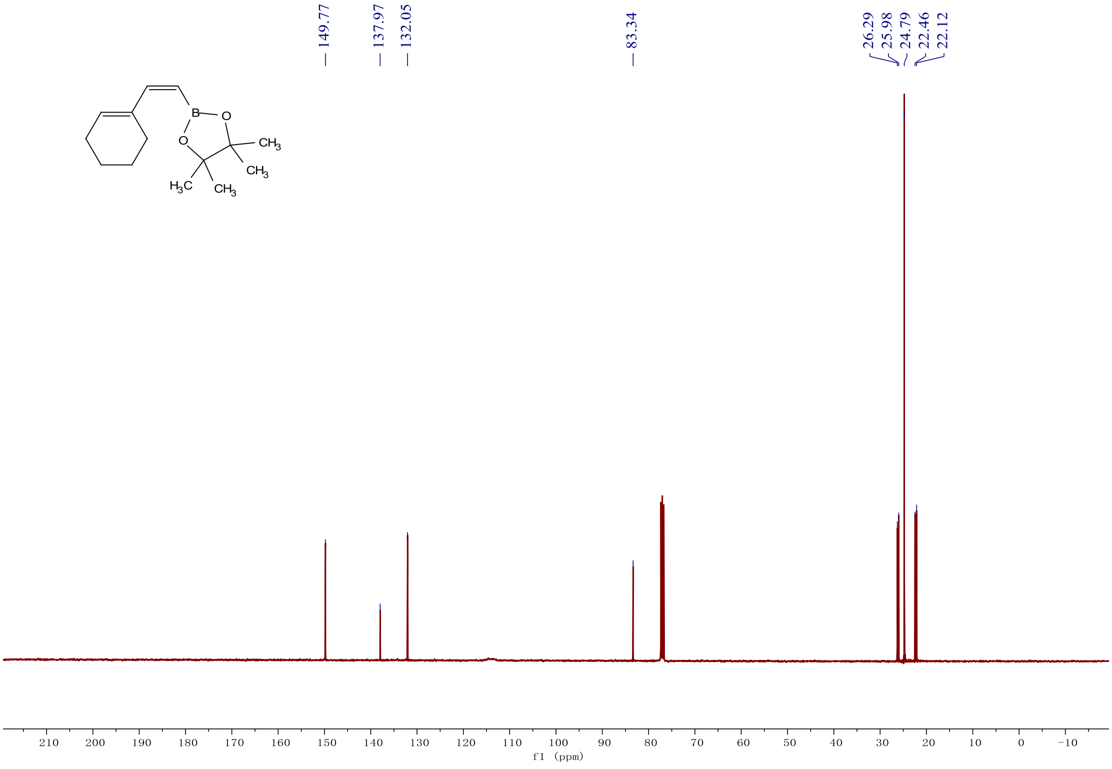

^{11}B NMR (128 MHz, Chloroform-*d*) of 40**b** (<u>*See procedure*</u>)

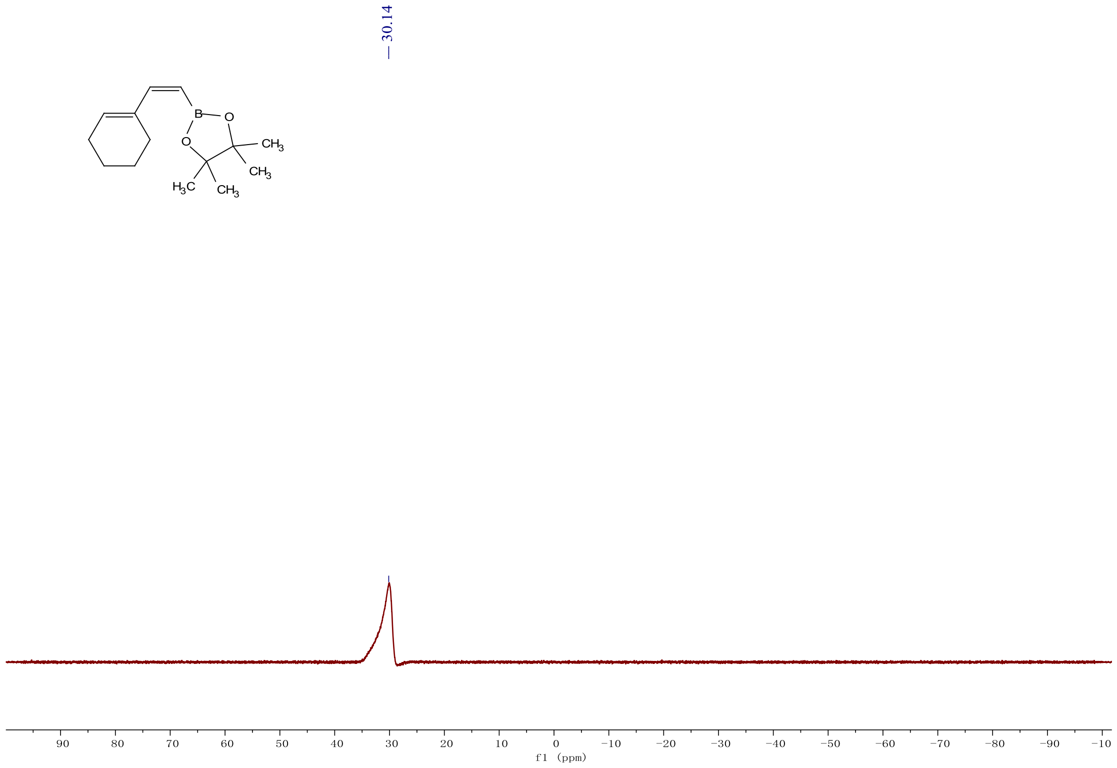

^{1}H NMR (600 MHz, Chloroform-*d*) of 41**b** (<u>*See procedure*</u>)

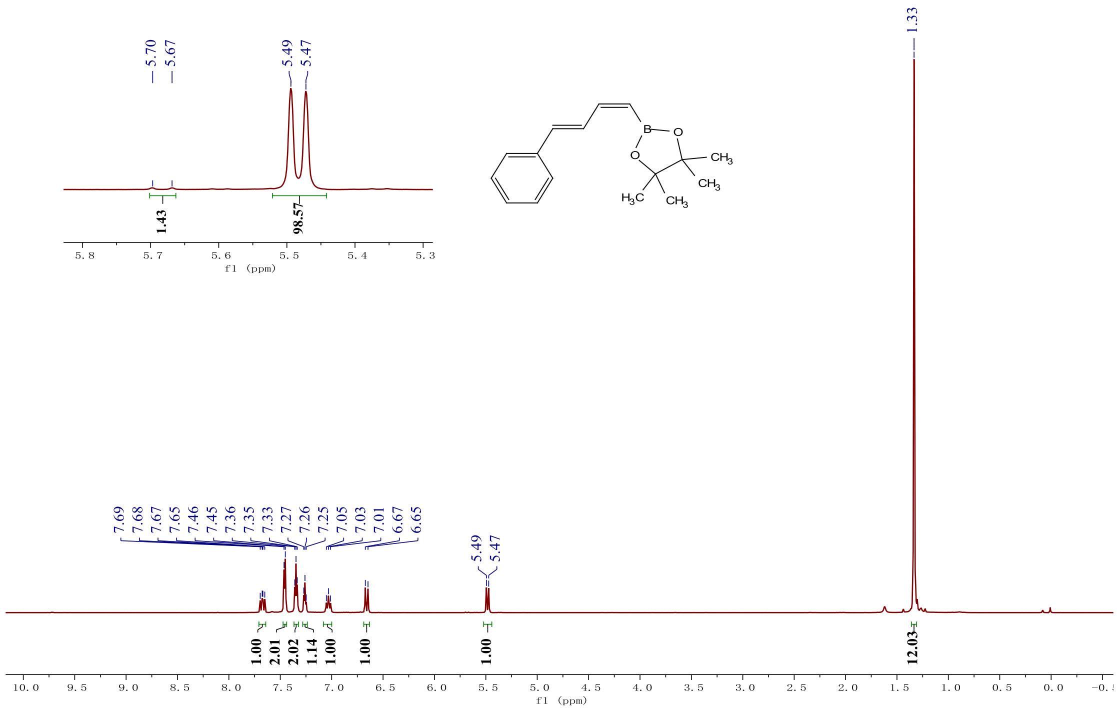

^{13}C NMR (151 MHz, Chloroform-*d*) of 41**b** (*See procedure*)

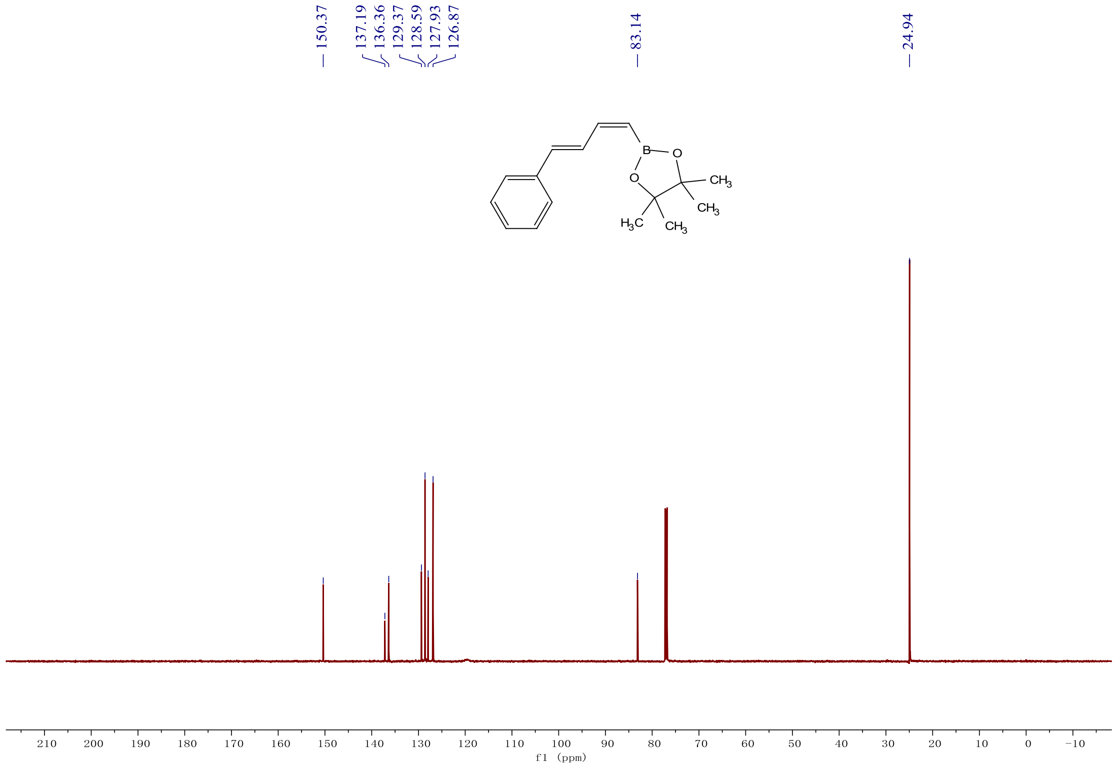

^{11}B NMR (193 MHz, Chloroform-*d*) of 41**b** (*See procedure*)

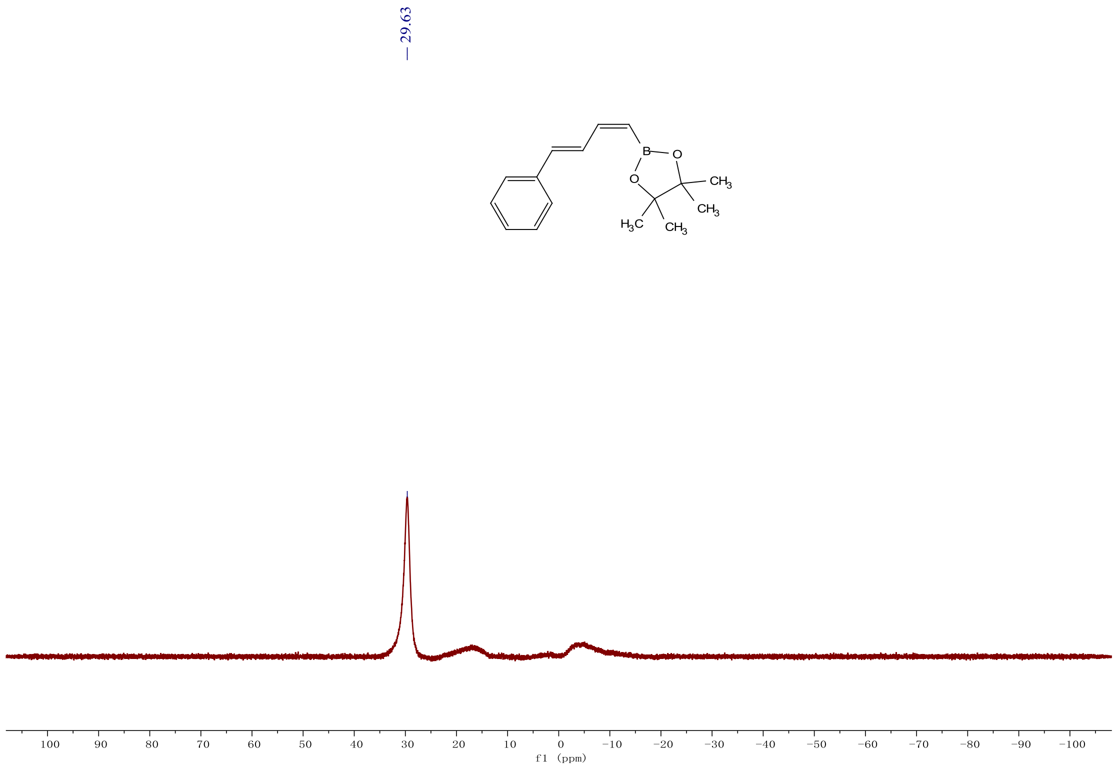

^{1}H NMR (600 MHz, Chloroform-*d*) of 42**b** (*See procedure*)

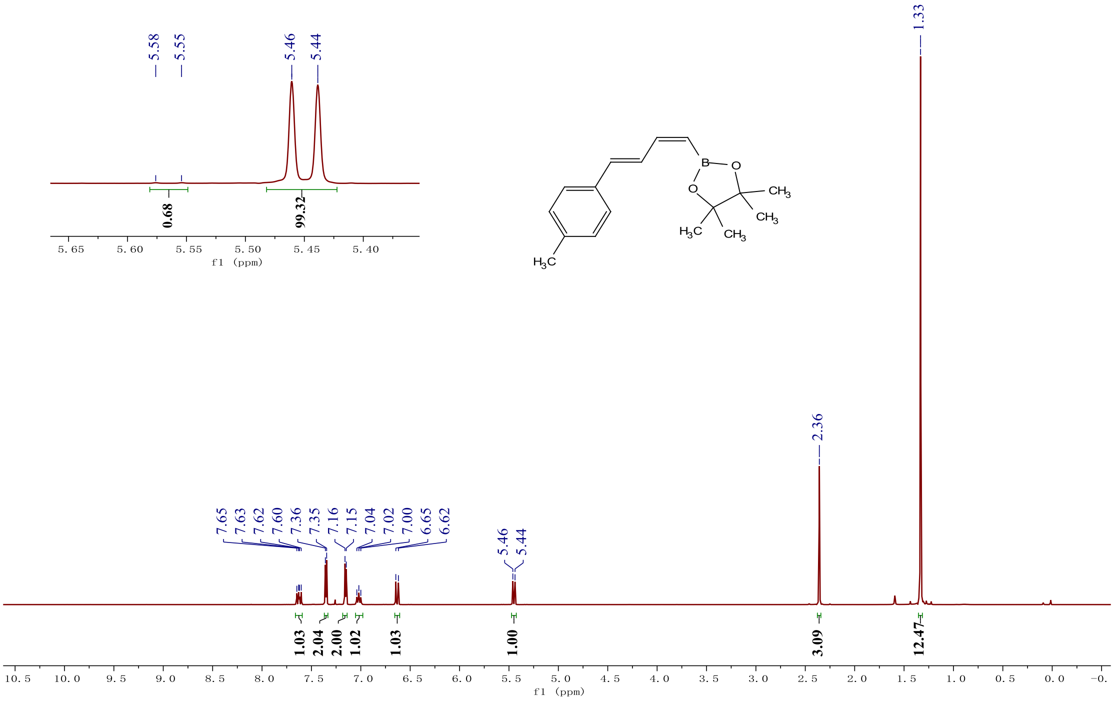

^{13}C NMR (151 MHz, Chloroform-*d*) of 42**b** (*See procedure*)

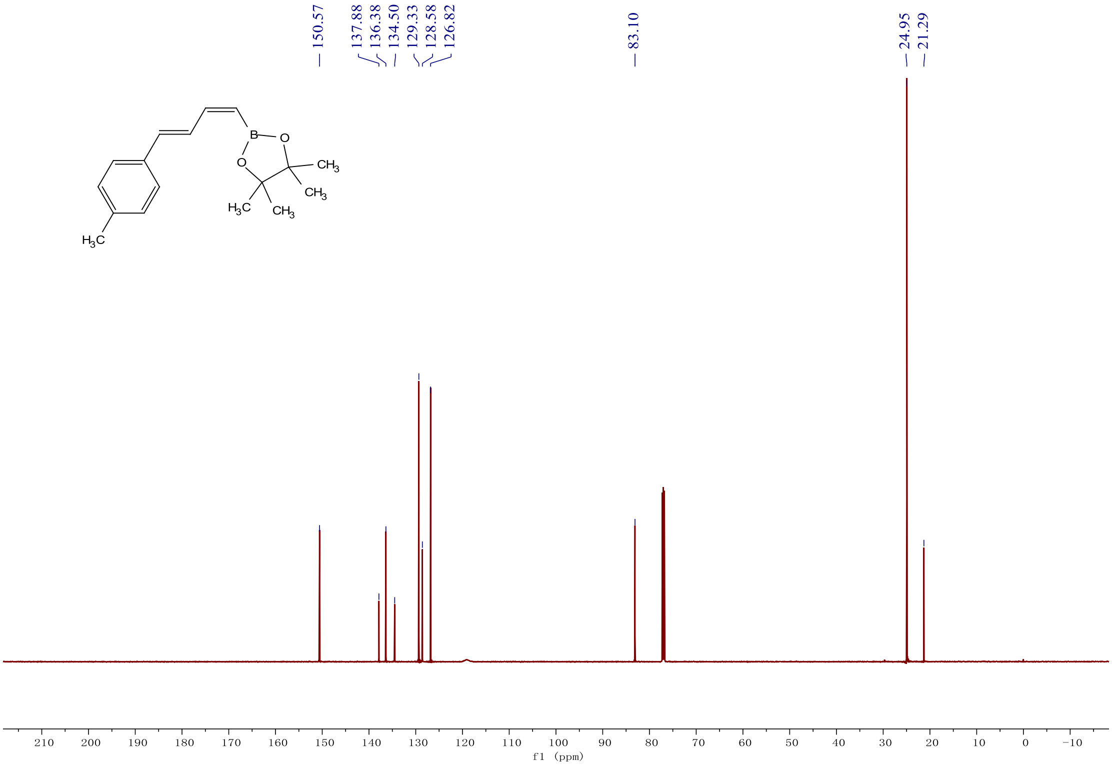

^{11}B NMR (193 MHz, Chloroform-*d*) of 42**b** (*See procedure*)

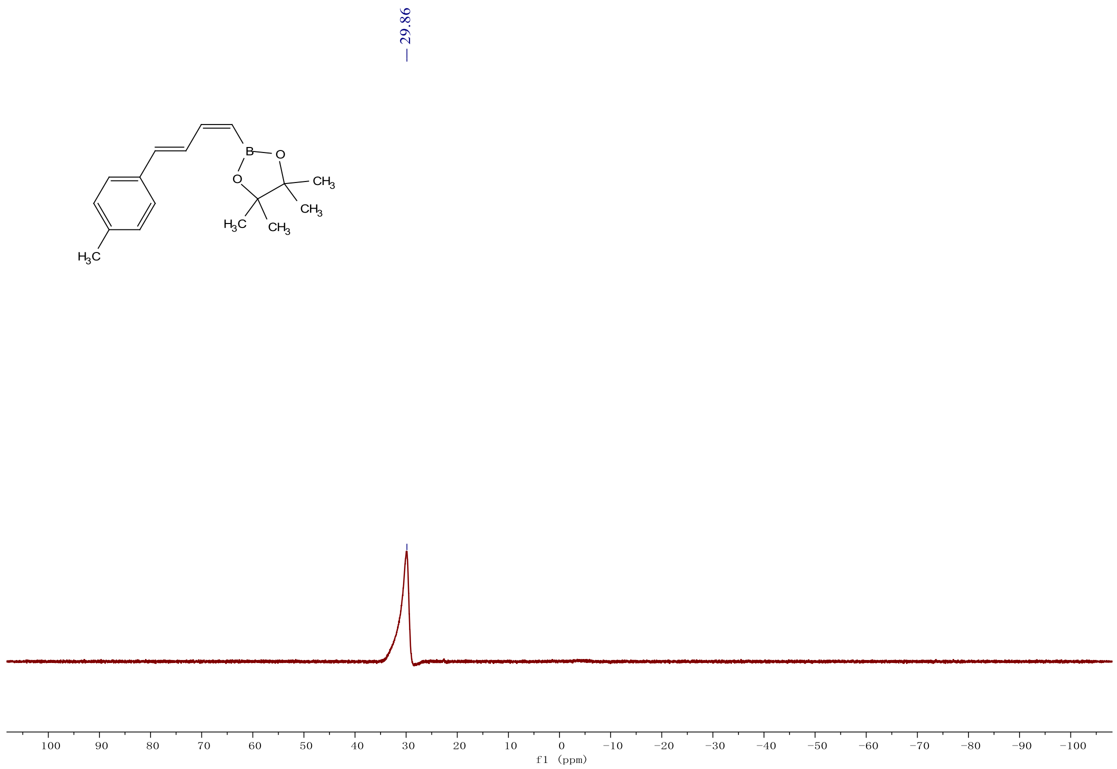

^{1}H NMR (400 MHz, Chloroform-*d*) of E-1**b** (*See procedure*)

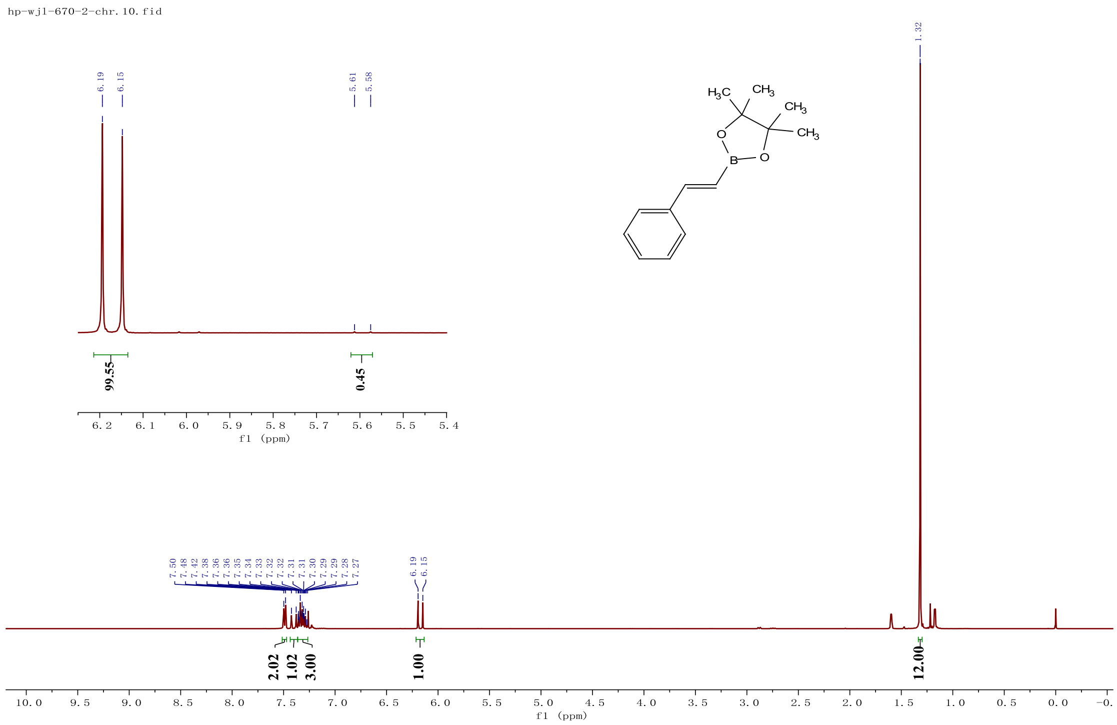

^{1}H NMR (400 MHz, Chloroform-*d*) of E-**2b** (<u>*See procedure*</u>)

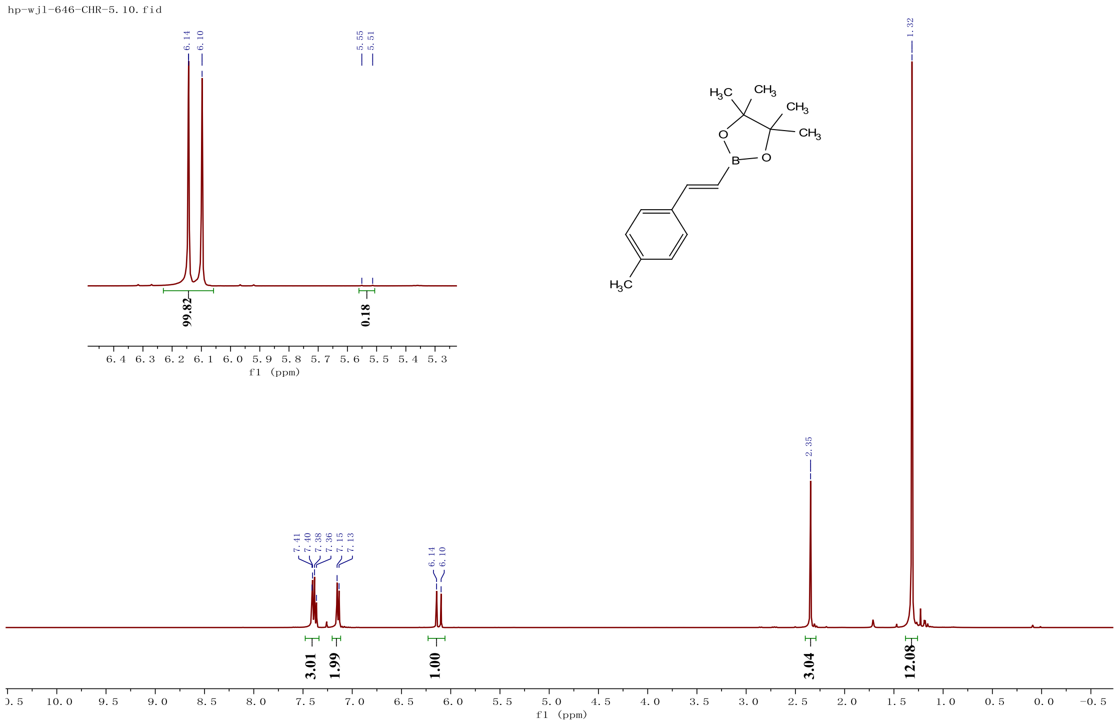

^{1}H NMR (400 MHz, Chloroform-*d*) of E-**3b** (<u>*See procedure*</u>)

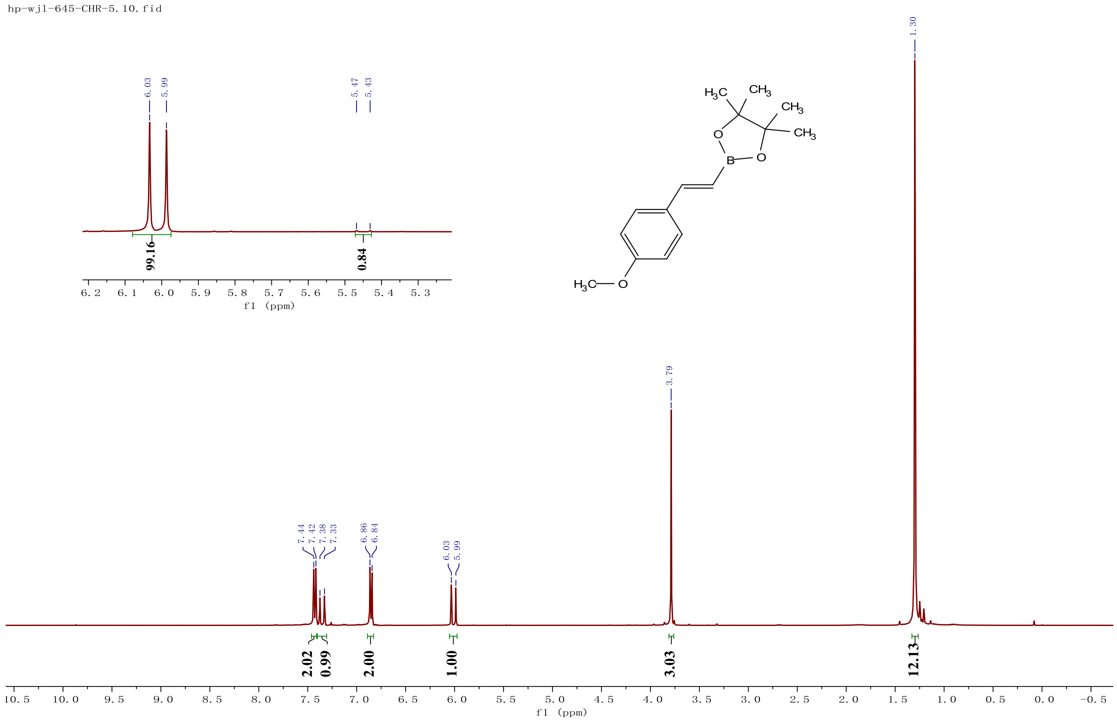

^{1}H NMR (400 MHz, Chloroform-*d*) of E-**13b** (*See procedure*)

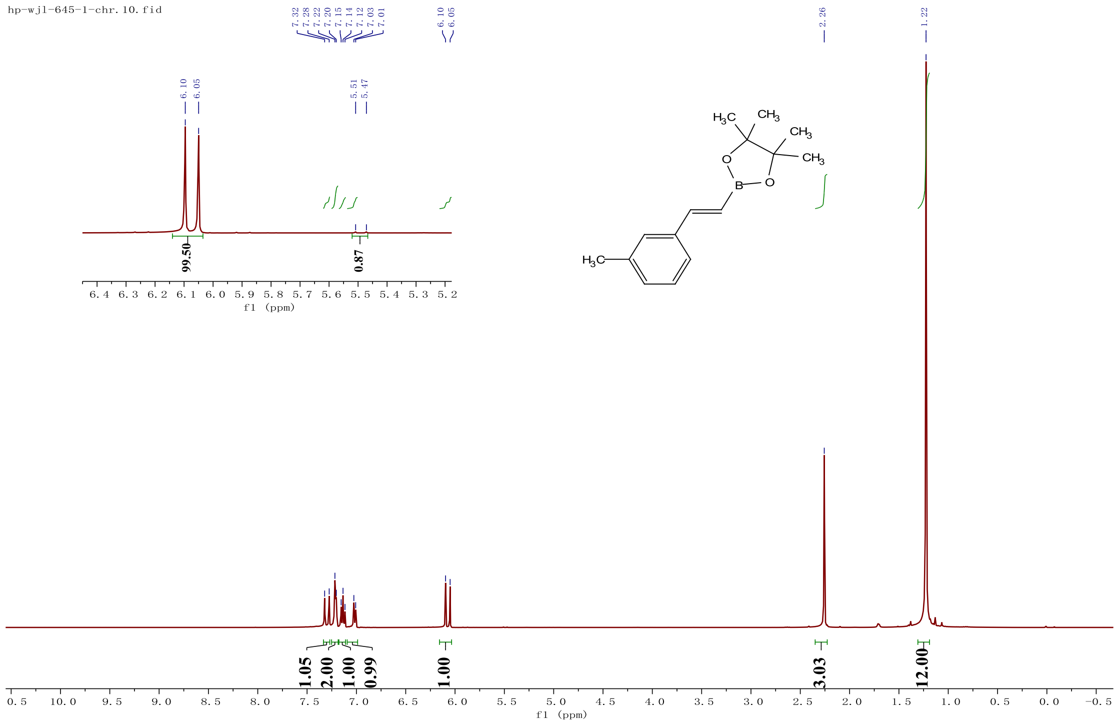

^{1}H NMR (400 MHz, Chloroform-*d*) of E-**4b** (*See procedure*)

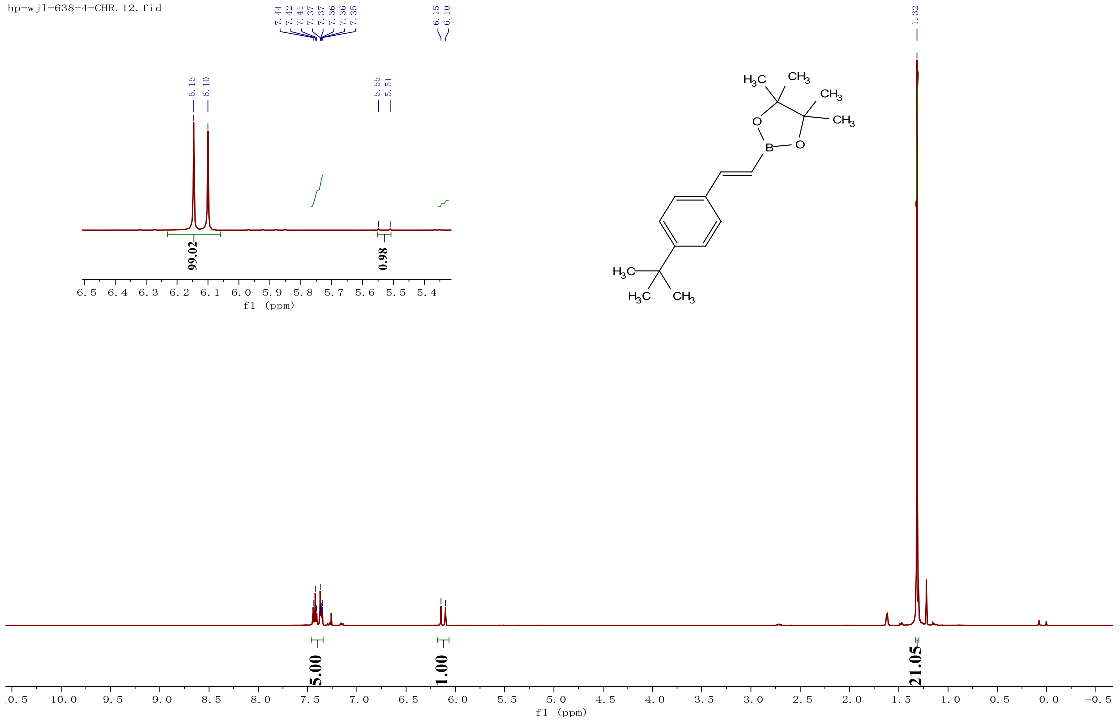

^{1}H NMR (400 MHz, Chloroform-*d*) of E-**14b** (*See procedure*)

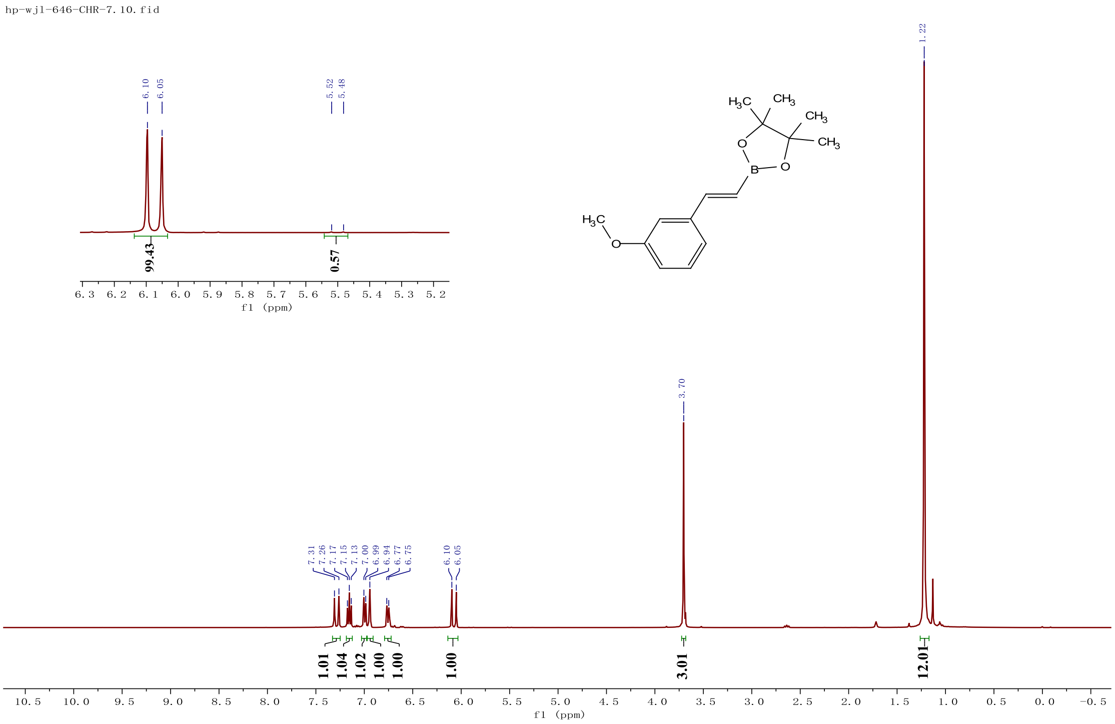

^{1}H NMR (400 MHz, Chloroform-*d*) of E-**30b** (*See procedure*)

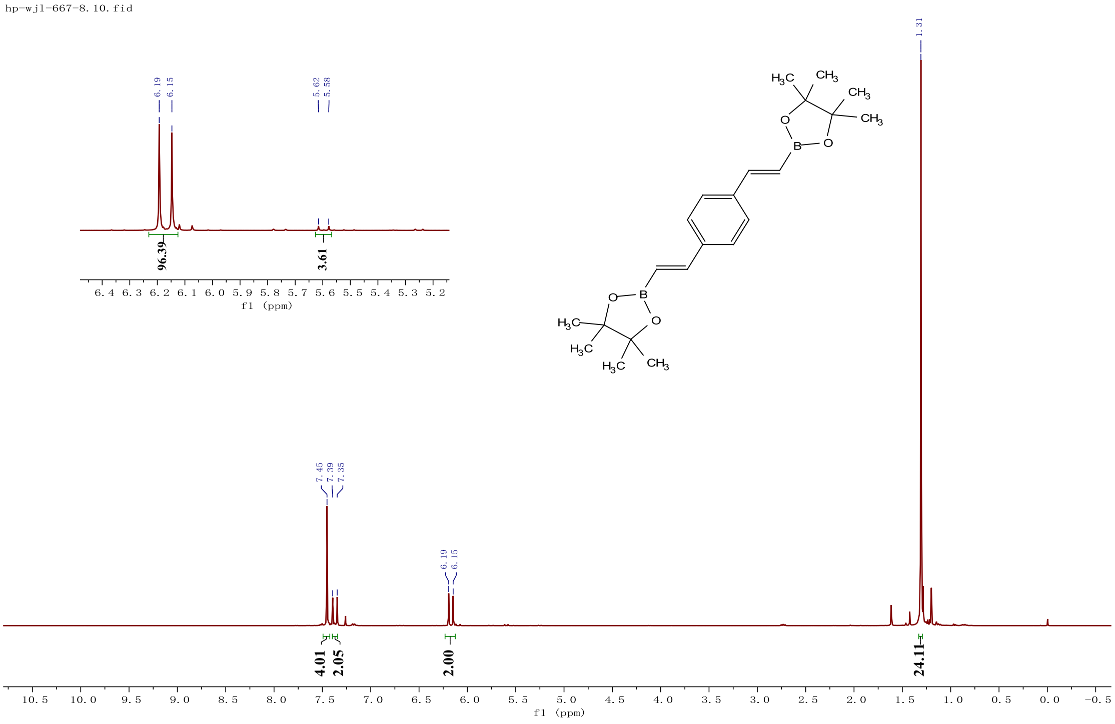

^{1}H NMR (400 MHz, Chloroform-*d*) of E-**39b** (See procedure)

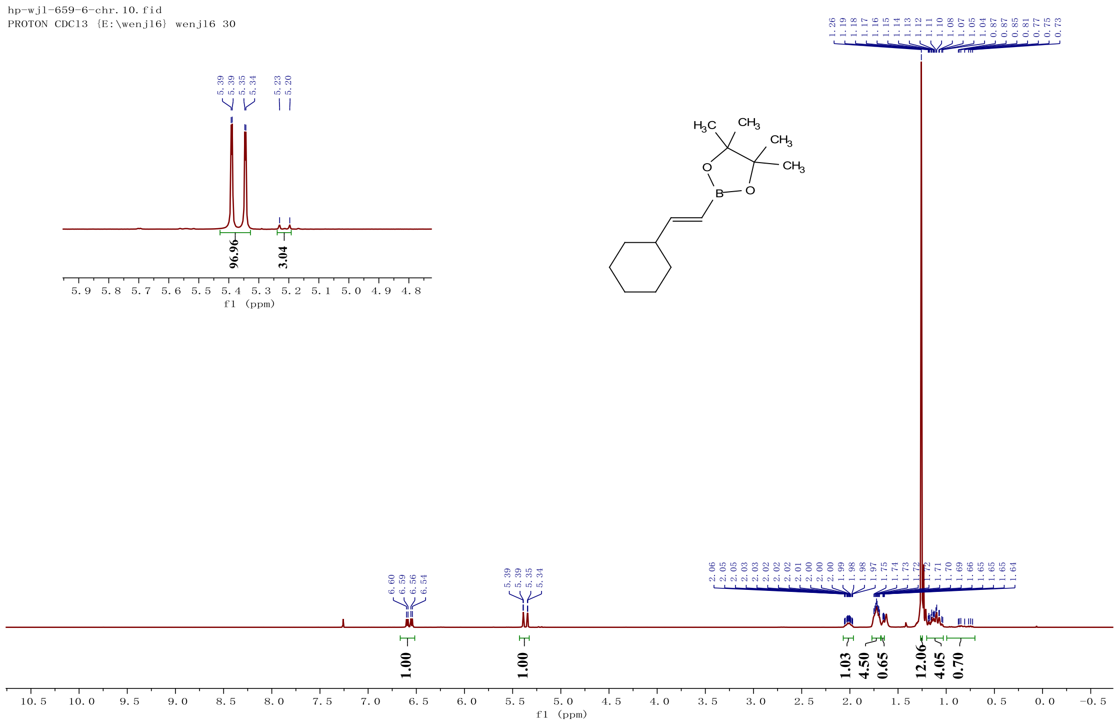

8. References

1. Scherg, T., Schneider, S. K., Frey, G. D., Schwarz, J., Herdtweck, E., Herrmann, W. A. Bridged Imidazolium Salts Used as Precursors for Chelating Carbene Complexes of Palladium in the Mizoroki-Heck Reaction. *Synlett* **18**, 2894–2907 (2006).

2. Gong, H. Y., Rambo, B., Karnas, E., Lynch, V. M., Sessler, J. L. A 'Texas-sized' molecular box that forms an anion-induced supramolecular necklace. *Nature Chem* **2**, 406–409 (2010).

3. Lan, X., Ye, Z., Liu, J., Huang, M., Shao, Y., Cai, X., Liu, Y., Ke, Z. Sustainable and Selective Alkylation of Deactivated Secondary Alcohols to Ketones by Non-bifunctional Pincer N-heterocyclic Carbene Manganese. *ChemSusChem* **13**, 2557–2563 (2020).

4. Andrade, G. A., DiMeglio, J. L., Guardino, E. T., Yap, G. P. A., Rosenthal, J. Synthesis and structure of palladium(II) complexes supported by bis-NHC pincer ligands for the electrochemical activation of CO2 *Polyhedron* **135**, 134–143 (2017).

5. Koch, A., Krieck, S., Görls, H. & Westerhausen, M. Alkaline Earth Metal–Carbene Complexes with the Versatile Tridentate 2,6-Bis(3-mesitylimidazol-2-ylidene)pyridine Ligand. *Organometallics* **36**, 994−1000 (2017).

6. Chan, W., Ho, C., Wong, M. & Che, C. Oxidative Amide Synthesis and N-Terminal α-Amino Group Ligation of Peptides in Aqueous Medium. *J. Am. Chem. Soc.* **46**, 14796–14797 (2006).

7. Xu, C., Du, W., Zeng, Y., Dai, B. & Guo, H. Reactivity Switch Enabled by Counterion: Highly Chemoselective Dimerization and Hydration of Terminal Alkynes. *Org. Lett.* **16**, 948−951(2014).

8. Santandrea, J., Minozzi, C., Cruche, C. & Collins, S. K. Photochemical Dual-Catalytic Synthesis of Alkynyl Sulfides. *Angew. Chem. Int. Ed.* **56**, 12255–12259 (2017).

9. Cassar, D. J., Nagaradja, E., Butler, David. C. D., Villemin, D. & Richards, C. J. Regioselective, Stereoselective, and Conformationally Controlled Synthesis of (η4-Tetraarylcyclobutadiene)(η5-carbomethoxycyclopentadienyl)cobalt Metallocenes. *Org. Lett.* **14**, 894-897 (2012).

10. Shu, C., Liu, R., Liu, S., Li, j., Yu, Y., He, Q., Lu, X. & Ye, L. Practical, Modular, and General Synthesis of 3-Coumaranones through Gold-Catalyzed Intermolecular Alkyne Oxidation Strategy. *Chem. Asian J.* **10**,91–95 (2015).
11. Bens, T., Boden, P., Di Martino-Fumo, P., Beerhues, J., Albold, U., Sobottka, S., Neuman, N. I., Gerhards, M. & Sarkar, B. Chromium(0) and Molydenum(0) Complexes with a Pyridyl-Mesoionic Carbene Ligand: Structural, (Spectro)electrochemical, Photochemical, and Theoretical Investigations. *Inorg. Chem.* **59**, 15504–15513 (2020).
12. Kolb, B., dos Santos, D. S., Krause, S., Zens, A., Laschat, S. Sequential hydrozirconation/Pd-catalyzed cross coupling of acyl chlorides towards conjugated (2E,4E)-dienones *Beilstein J. Org. Chem.* 19, 176–185 (2023).
13. Polášek, J., Paciorek, J., Stošek, J., Semrád, H., Munzarová, M. & Mazal C. Stereoselective Bromoboration of Acetylene with Boron Tribromide: Preparation and Cross-Coupling Reactions of (Z)-Bromovinylboronates. *J. Org. Chem.* **85**, 6992−7000 (2020).
14. Garhwal, S., Fridman, N. & de Ruiter, G. Z-Selective Alkyne Functionalization Catalyzed by a trans-Dihydride N-Heterocyclic Carbene (NHC) Iron Complex. *Inorg. Chem.***59**, 13817-13821 (2020).
15. Nishihara, Y., Kinoshita, M., Hyodo, K., Okuda, Y., Eguchi, R., Goto, H., Hamao, S., Takabayashi, Y. & Kubozono, Y. Phenanthro[1,2-b : 8,7-b']dithiophene: a new picene-type molecule for transistor applications. *RSC Adv*. **3**, 19341-19347 (2013).
16. Gunanathan, C., Hölscher, M., Pan, F. & Leitner, W. Ruthenium Catalyzed Hydroboration of Terminal Alkynes to Z-Vinylboronates. *J. Am. Chem. Soc.***134**, 14349-14352 (2012).
17. Obligacion, J. V., Neely, J. M., Yazdani, A. N., Pappas, I. & Chirik, P. J. Cobalt Catalyzed Z-Selective Hydroboration of Terminal Alkynes and Elucidation of the Origin of Selectivity. *J. Am. Chem. Soc.* **137**, 5855-5858 (2015).
18. Ohmura, T., Yamamoto, Y. & Miyaura, N. Rhodium- or Iridium-Catalyzed trans-Hydroboration of Terminal Alkynes, Giving (Z)-1-Alkenylboron Compounds. *J. Am. Chem. Soc.* **122**, 4990-4991 (2000).
19. Lyu, Y., Toriumi, N. & Iwasawa, N. (Z)-Selective Hydroboration of Terminal Alkynes Catalyzed by a PSP–Pincer Rhodium Complex. *Org. Lett.* **23**, 9262-9266 (2021).
20. Xu, S. Zhang, Y., Li, B. & Liu, S. Site-Selective and Stereoselective trans-Hydroboration of 1,3-Enynes Catalyzed by 1,4-Azaborine-Based Phosphine–Pd Complex. *J. Am. Chem. Soc.* **138**, 14566-14569 (2016)
21. Magre, M., Maity, B., Falconnet, A., Cavallo, L. & Rueping, M. Magnesium-Catalyzed Hydroboration of Terminal and Internal Alkynes. *Angew. Chem. Int. Ed.* **58**, 7025–7029 (2019).
22. Liu, J., Wu, C., Hu, T., Yang, W., Xie, Y., Shi, Y., Liu, Q., Shao, Y. & Zhang, F. Hexamethyldisilazane Lithium (LiHMDS)-Promoted Hydroboration of Alkynes and Alkenes with Pinacolborane. *J. Org. Chem.* **87**, 3442−3452 (2022).
23. Wannagat, U., Niederprüm, H., *Chem. Ber.* **94**, 1540–1547 (1961).
